# Associations of known and newly identified human milk oligosaccharides with infections in early childhood: the Ulm SPATZ health study

**DOI:** 10.3389/fimmu.2025.1703579

**Published:** 2025-12-05

**Authors:** Linda P. Siziba, Zhuoxin Peng, Marko Mank, Bernd Stahl, John Gonsalves, Deborah Wernecke, Dietrich Rothenbacher, Jon Genuneit

**Affiliations:** 1 Pediatric Epidemiology, Department of Pediatrics, Medical Faculty, Leipzig University, Leipzig, Germany; 2 Danone Research and Innovation, Utrecht, Netherlands; 3 Department of Chemical Biology and Drug Discovery, Faculty of Science, Utrecht Institute for Pharmaceutical Sciences, Utrecht University, Utrecht, Netherlands; 4 Institute of Epidemiology and Medical Biometry, Ulm University, Ulm, Germany; 5 German Center for Child and Adolescent Health (DZKJ), Partner Site Ulm, Ulm, Germany

**Keywords:** human milk oligosaccharides (HMOs), lower respiratory tract infections (LRTI), otitis media (OM), secretor milk, infant infections, human milk, immune development

## Abstract

**Introduction:**

Human milk oligosaccharides (HMOs) are bioactive components of breast milk that play a key role in shaping infant immune development and susceptibility to infections. This study investigated associations between 71 known and novel HMOs measured at 6 weeks and 6 months postpartum with infant infections during the first 2 years of life.

**Methods:**

A total of 73 HMOs were analyzed in human milk sampled at 6 weeks (n = 144) and 6 months (n = 133) using LC-ESI-IM-qTOF-MS. Infections in infants were assessed using physician-reported questionnaires at 1 and 2 years. Modified Poisson regression was used to assess associations, adjusted for relevant covariates and corrected for multiple testing (FDR < 0.010).

**Results:**

Higher levels of fucosyl(1-3)-iso-lacto-N-octaose, lacto-N-tetraose (LNT), and sialyllacto-N-tetraose b (LSTb) at 6 weeks were associated with higher likelihood of lower respiratory tract infection (LRTI) at 1 year of age. In contrast, elevated difucosyldisialyllacto-N-hexaose-X2 and difucosyl-lacto-N-hexaose II in non-secretor milk were linked to reduced likelihood of otitis media (OM) in the cumulative 2-year period. Higher levels of LNT2 and LSTa in secretor milk at 6 months were associated with a higher likelihood of LRTI in the first and cumulatively up to the second year of life, respectively.

**Conclusion:**

These findings suggest that specific HMOs may influence early-life infection susceptibility. However, the associations likely reflect a complex, dynamic balance in human milk composition, potentially driven by early microbial exposures or maternal responses. Further research is needed to clarify whether HMOs directly modulate susceptibility to infection or act through broader immunological pathways.

## 1 Introduction

Human milk oligosaccharides (HMOs) are conjugated glycans found in human milk, and they play a multifunctional role in infant health (1, 2). Their relative abundance and concentrations differ significantly among various populations of women and can also fluctuate within individual milk samples throughout the lactation period (3–5). These variations in HMOs are primarily influenced by genetic factors, particularly the maternal Secretor (FUT2) and Lewis (FUT3) genes (6, 7). The active Secretor gene leads to the expression of the α1-2-fucosyltransferase enzyme, which regulates the levels of α1,2-fucosylated HMOs such as 2′-fucosyllactose (2′-FL) and lacto-N-fucopentaose I (LNFP I) in human milk. In contrast, the Lewis gene affects the secretion of α1,3- and α1,4-fucosylated HMOs, including lacto-N-fucopentaose II (LNFP II) (8). To obtain, for example the more complex difucosylated HMO lacto-N-difucohexaose I (LNDFH I), both FUT 2 and FUT3 need to be active.

To date, more than 200 distinct HMOs (9) have been identified. Their structural diversity and abundance confer a range of biological functions, including providing protection against diarrhea, infections, and overall morbidity (1, 10–13). For example, some associations between HMOs and otitis media (OM) (14), acute respiratory infections (ARIs) (15, 16), lower respiratory tract infections (LRTI) (17), and childhood respiratory health (18) have been reported. However, the results remain inconsistent and often statistically insignificant following correction for multiple testing. This highlights the complexity of the relationship between HMOs and infant health, suggesting the need for further investigation into their biological roles.

Despite the growing interest, previous studies focused on one specific HMO (14), more or less than 15 (17) and sometimes up to 51 (15) individual HMO structures, in addition to structure-specific groups of HMOs (15) and secretor status and milk groups (17). While these approaches have provided valuable insights, they may not give a full picture of the structural diversity and interrelated nature of HMO profiles. In addition, this area of research could have important implications for the development of targeted interventions, such as HMO supplementation, to reduce the burden of common infections in infancy. Our study therefore aimed to expand the number of HMOs analyzed, and to improve the resolution of structure-dependent associations within this complex biochemical network of glycans. Thus, by quantifying up to 71 individual HMOs, including new isomers (19). We sought to better understand structure-dependent associations of HMOs, which have been hard to pinpoint due to the limited characterization of most HMOs (11). We therefore investigated the associations between these 71 individual HMOs and infections—specifically upper respiratory tract infections (URTI), OM, and LRTI—during the first 2 years of life in the context of an observational birth cohort study.

## 2 Methods

### 2.1 Study design and population

Data used in the current study are from the Ulm SPATZ Health Study, an ongoing birth cohort study. This study recruited a total of 970 mothers (representing 49% of all eligible families) and their 1,006 newborns shortly after delivery, during their hospital stay at the University Medical Centre Ulm, southern Germany, between April 2012 and May 2013 (20). Notably, the University Medical Centre was the only hospital serving Ulm and its surrounding areas; our data therefore provide a representative sample of the general population of women who gave birth in the region around Ulm. Exclusion criteria included inadequate German language skills, outpatient childbirth, maternal age under 18 years, postpartum transfer of mother or child to intensive care, or stillbirth. All participants provided written informed consent prior to the study, and participation was entirely voluntary. Ethical approval was obtained from the Ethics board of Ulm University (No. 311/11).

### 2.2 Data collection and measurements

Demographic, lifestyle, and birth-related data were collected through self-administered questionnaires, electronic hospital records, and routine screening exams. This included information on child sex, delivery mode, maternal age, education level, parity, and pre-pregnancy body mass index (BMI, calculated as mass(kg)/height(m)²). Standardized questionnaires documented further information on social demographic details, living conditions, and lifestyle factors among other information. Additional clinical data concerning the child’s birth and the mother’s pregnancy were obtained from routine paper records.

Reported medical diagnoses of OM, LRTI, and URTI were evaluated respectively at 1 and 2 years of age through standardized self-administered questionnaires completed by the children’s primary care pediatricians. These questionnaires captured doctor-confirmed diagnosis and not parent symptom reports and were uniformly structured for all pediatricians. Parent reports of OM were defined as a positive response of “otitis media” to the question “Has a doctor diagnosed your child with one of the following in the past 12 months?” asked in the follow-ups at ages 1 and 2 years. Separately assessed pediatrician reports of OM, LRTI, and URTI were defined as a positive response of “otitis media” to the question “Has one of the following bacterial diseases been found/diagnosed by a doctor until now?”; “lower respiratory tract infections (i.e., pneumonia, bronchitis, pertussis, tracheobronchitis, croup, bronchiolitis, and influenza)”; or “upper respiratory tract infections (i.e., rhinitis, pharyngitis, tonsillitis, and epiglottitis)” to the question “Has one of the following diseases been diagnosed from/by a doctor until now?” These reports were for a doctor’s diagnosis although not necessarily made by the caring and responding pediatricians themselves. For the purposes of the current analysis, children with a positive report of a doctor’s diagnosis of OM, LRTI, or URTI by either the parents (OM only) or the caring pediatrician were defined as cases. At 2 years of age, the positive reports of 1 year were also included (i.e., cumulative frequencies of OM, LRTI, and UTRI reports). There were no additional diagnostic criteria or validations through individual medical record reviews. Several other health outcomes were assessed concurrently using these questionnaires. Additional data were collected at 6 weeks, 6 months, and 12 months postpartum via telephone interviews or via mailed self-administered questionnaires if participants were unreachable by phone or had previously reported stopping breastfeeding. Follow-up assessments have been conducted on a yearly basis and are still ongoing.

### 2.3 Human milk sample selection

**Figure 1:**
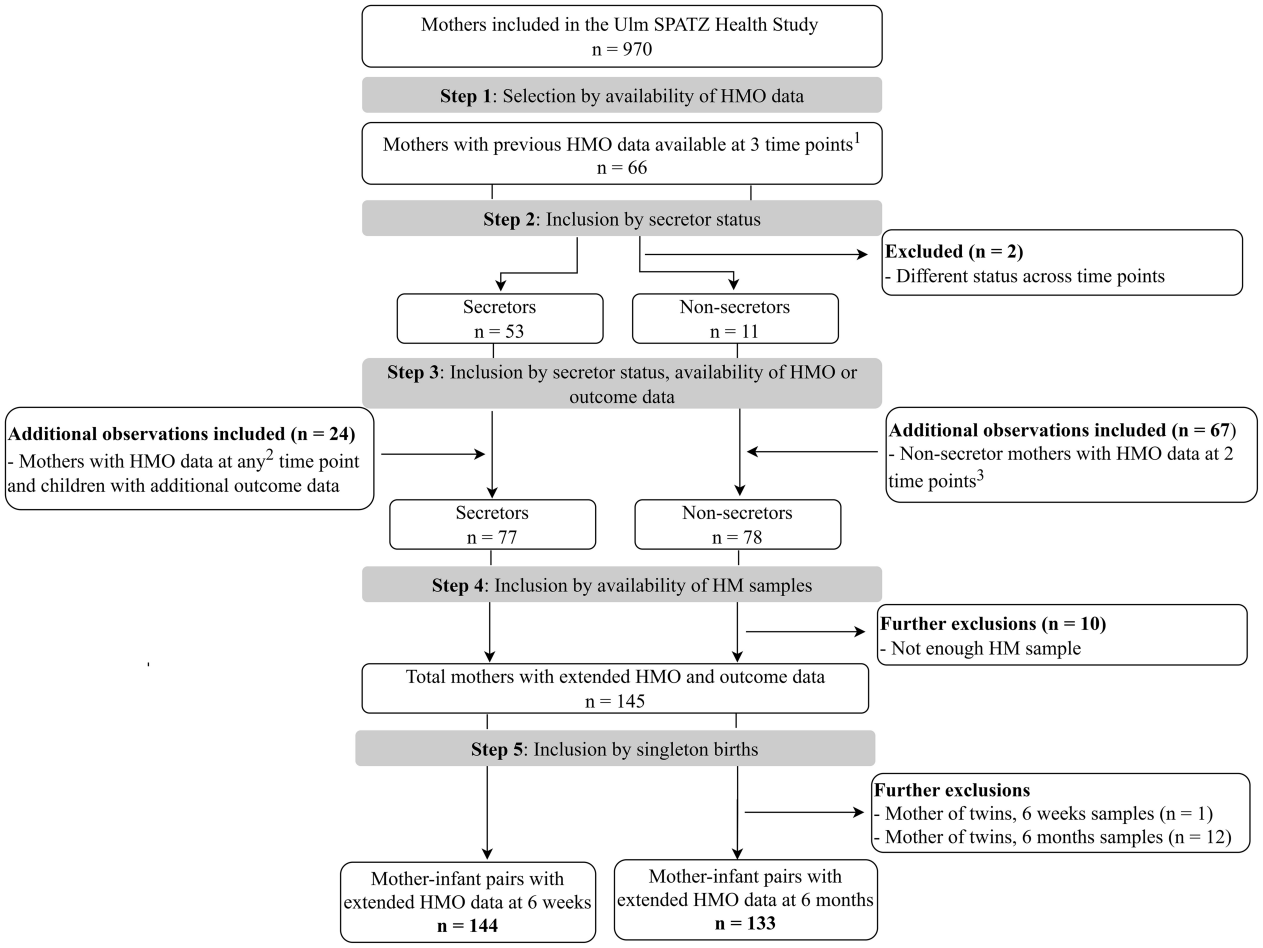
Flowchart showing the selection process of mother–infant pairs with human milk samples for advanced human milk oligosaccharide (HMO) analysis included in this study. Only mothers with HMO data and consistent secretor status were eligible for the extended HMO analysis. The upper division of the diagram shows separation by maternal secretor status (left = secretors; right = non-secretors). The lower division represents the availability of HMO data at different time points (6 weeks and/or 6 months). All data are from the Ulm SPATZ Health Study, no additional participants were included from other sources. The final sample size included in the study comprised 145 mothers (144 mother–infant pairs with HMO data at 6 weeks, and 133 pairs with HMO data at 6 months. ^1^All three time points, i.e., 6 weeks, 6 months, and 12 months; ^2^at any one of the time points; ^3^at both 6 weeks and 6 months. HM, human milk; HMO, human milk oligosaccharide.

Human milk samples were collected from lactating mothers at approximately 6 weeks, 6 months, and 12 months after delivery, as previously described (5). Briefly, mothers expressed milk following a standardized procedure between 9 am and 12 pm, with trained nurses assisting when needed, and stored the samples in their refrigerators until collection. Samples were collected on the same day if milk was expressed between 9 am and 12 pm. If milk was expressed after 12 pm, in the evening, or before 9 am, the samples were collected the next day. The milk was transported under refrigeration to the study center, where it was aliquoted and frozen at −80°C within 48 h of collection and stored for later analysis. Sample selection for the advanced HMO analysis (19) was based on the availability of previous HMO data (5), maternal secretor status, and relevant outcome data, including infections (**Figure 1**). All participants selected and included in this study were from the Ulm SPATZ Health Study; no external datasets were combined or used. The flow diagram shows how the 6-week and 6-month subsets were selected according to the HMO data that were already available at all three (6 weeks, 6 months, and 12 months) or two (6 weeks and/or 6 months) timepoints, as well as maternal secretor status.

### 2.4 Analysis of HMOs

A total of 73 individual HMO structures were measured in a blinded fashion using liquid chromatography coupled with electrospray-ionization ion mobility quadrupole time-of-flight mass spectrometry (LC-ESI-IM-qTOF-MS) at Danone Research & Innovation (Utrecht, NL), as previously described (19, 21). Briefly, individual HMO structures were identified and quantified by LC-ESI-IM-qTOF-MS in negative ion mode. Quantification of relative HMO levels was done for 73 HMOs comprising neutral, acidic, and sulphated HMOs. The four common human milk types were determined for each sample based on the quantified levels of specific HMOs as follows (19): (i) samples with LNFP I and lacto-N-difucohexaose I (LNDFH I) below the limit of quantification (<LLOQ) were classified as type II; (ii) those with LNFP II and LNDFH I <LLOQ were classified as type III; (iii) those with LNFP I, LNFP II, and LNDFH I all <LLOQ were classified as type IV; (iv) all remaining samples were categorized as type I. Secretor milk comprised type I and III milk, whereas non-secretor milk comprised type II and type IV milk. A total of n=144 and n=133 mother–infant pairs with HMO data at 6 weeks and 6 months, respectively, were included in the current analysis and thus had a complete set of HMO and outcome data (**Figure 1**).

### 2.5 Statistical analysis

Data for blood group B-pentasaccharide and lacto-N-difucohexaose II were excluded because ≥90% and ≥30% of the values, respectively, were <LLOQ or >ULOQ. For the remaining 71 HMOs, data <LLOQ were replaced by LLOQ/√2, whereas values >ULOQ were extrapolated based on the HMO-specific calibration line. Due to the selection criteria for HMO analysis, the sample size had an approximately even distribution of secretors and non-secretors (**Figure 1**). Analytical weights were applied to account for the overrepresentation of secretors and non-secretors, respectively, in the two human milk subsets to reflect the expected distribution in the general population 80% secretors to 20% non-secretors) (22). Weights were calculated based on the ratio of the target to observed proportions and incorporated into the overall non-stratified analyses. HMO levels at 6 weeks and 6 months of lactation were reported as mean values. Differences in HMO levels between groups were assessed using the Wilcoxon rank-sum test. Blom’s normal ranks transformation (23) was applied to individual HMO levels before and after stratification by secretor status, prior to inclusion into models. This transformation provides an accurate approximation of expected normal scores, allowing results to be interpreted as the effect of a one-unit standardized increase in HMO levels. Normalization post-stratification was conducted as a sensitivity analysis to determine whether group-specific distributions influenced effect sizes and confidence intervals.

Modified Poisson regression (24) was used to assess associations between individual HMOs and OM, LRTI, or URTI. Analyses were conducted in both crude and adjusted models, with the weighted secretor variable included in non-stratified models. Risk ratios (RR) and 95% confidence intervals (95% CI) were derived by exponentiating the estimated coefficients, as a logarithmic link function was used to model HMO levels as continuous independent variables. The models were adjusted for infant sex, duration of exclusive breastfeeding (weeks), birth weight, gestational age (weeks), and batch effects. These covariates were selected based on prior reports of their associations with infection risk in early childhood (25–29). When delivery mode was included in the adjusted model, convergence issues arose, likely due to the sparse data in the caesarean subgroups. Of note, caesarean section accounted for ~20% of the deliveries among infants with HMO data (7% elective, and 13% emergency). Thus, given the small numbers and model instability, delivery mode was therefore not included in the final adjusted models. A stringent false discovery rate (FDR) threshold of α = 0.010 was chosen to minimize false positives while maintaining sufficient power (30). We modelled each HMO individually, adjusting for relevant confounders and FDR correction to account for multiple testing because the primary aim of this study was to identify specific HMOs associated with infection outcomes. We chose this targeted and hypothesis-generating approach to maximize interpretability and to avoid overfitting, given the small subgroup sample sizes relative to the number of HMOs measured. Additionally, the biological structure of the HMO profiles was partly addressed in stratified analyses, which accounts for the major differences in HMO composition. All statistical analyses were conducted using SAS version 9.4 (The SAS Institute, Cary, NC, USA) and R (version 3.5.1; R Foundation for Statistical Computing).

## 3 Results

A total of n = 144 and n = 133 mother–infant pairs had extended HMO data at 6 weeks and 6 months as well as outcome data, respectively (**Table 1**). The majority of infants (82%, n = 112) were receiving exclusive human milk at 6 weeks. Only a fifth (20%) of the infants with HMO data available at 6 weeks (n = 29, 20.3%) or 6 months (n = 26, 19.5%) were born through elective or emergency caesarean section. Overall, more than half (50%) of the children experienced at least one repeated infection within the first 2 years. Among infants with HMO data available at 6 weeks and/or 6 months, the following prevalences of repeated infections were reported with the first 2 years of life, respectively: OM in 11.8% (n = 17) and 12.0% (n = 16), LRTI in 25.7% (n = 37) and 27.8% (n = 37), and URTI in 59.0% (n = 85) and 60.2% (n = 80). For the subsequent analyses, cumulated reports of infections up to 2 years were used. A doctor’s diagnosis of URTI was most prevalent at 1 and 2 years, whereas OM was the least commonly reported infection diagnosis, for infants with HMO data at either time point. Secretors comprised a little more than half (53%) of the mothers with HMO data at 6 weeks, whereas non-secretors were predominant (54.1%) in the group with HMO data at 6 months. After applying weights, the weighted distribution of secretor to non-secretor mothers was 80% *vs*. 20%.

**Table 1:** Characteristics of mother–infant pairs included in the current analysis.

	6-week samples (n=144)		6-month samples (n=133)	
Category	n	(%) or mean	n	(%) or mean
Child sex				
Female	60	41.7%	56	42.1%
Male	84	58.3%	77	57.9%
Duration of EBF (weeks)	144	18.1 (11.2)	133	18.8 (11.0)
EBF at time of sampling				
Yes	112	82.4%	33	25.8%
No	24	17.6%	95	74.2%
Frequency of HM feeds				
1 to 8	110	76.4%	117	88.0%
9 or more	34	23.6%	16	12.0%
Gestational age at delivery				
≤ 36 weeks	7	4.9%	7	5.3%
≥ 41 weeks	25	17.5%	22	16.5%
Between 36 and 41 weeks	111	77.6%	104	78.2%
Delivery mode^1^				
Elective caesarean	11	7.7%	9	6.8%
Emergency caesarean	18	12.6%	17	12.8%
Vaginal assisted	10	7.0%	8	6.0%
Vaginal spontaneous	104	72.7%	99	74.4%
Infections at 1 year				
Otitis media (OM)				
Yes	17	14.0%	16	14.2%
No	104	86.0%	97	85.8%
Lower respiratory tract infections (LRTI)				
Yes	37	29.8%	37	32.2%
No	87	70.2%	78	67.8%
Upper respiratory tract infections (URTI)				
Yes	85	66.9%	80	67.8%
No	42	33.1%	38	32.2%
Total infections at 1 year				
Single or none	107	74.3%	97	72.9%
2	31	21.5%	30	22.6%
3	6	4.2%	6	4.5%
Infections at 2 years (cumulative)				
OM				
Yes	38	30.6%	35	30.2%
No	86	69.4%	81	69.8%
LRTI				
Yes	62	47.7%	57	47.1%
No	68	52.3%	64	52.9%
URTI				
Yes	111	84.1%	104	84.6%
No	21	15.9%	19	15.4%
Any repeated infection by 2 years				
Yes	96	66.7%	91	68.4%
No	48	33.3%	42	31.6%
Total infections at 2 years				
Single or None	71	49.3%	67	50.4%
2	55	38.2%	49	36.9%
3	18	12.5%	17	12.8%
Maternal age (years)	143	34.2	132	34.3
Maternal BMI at time of sampling				
Normal (18.50 ≤ BMI < 25.0)	84	60.4%	79	63.7%
Obese (BMI > 30.0)	13	9.4%	11	8.9%
Overweight (25.0 ≤ BMI <30.0)	40	28.8%	28	22.6%
Underweight (BMI < 18.50)	2	1.4%	6	4.8%
Parity (n births of foetus ≥ 24 weeks)				
0 births	68	47.6%	62	46.6%
≥1 birth	75	52.4%	71	53.4%
Maternal secretor status				
Yes	76	52.8%	61	45.9%
No	68	47.2%	72	54.1%
Milk type				
I	71	49.3%	56	42.1%
II	65	45.1%	68	51.1%
III	5	3.5%	5	3.8%
IV	3	2.1%	4	3.0%

^1^Delivery mode: spontaneous or assisted vaginal delivery; or elective/emergency caesarean section spontaneous or assisted. HM, human milk; EBF, exclusive breastfeeding; BMI, body mass index.

### 3.1 Mean differences in HMO levels

**Figure 2:**
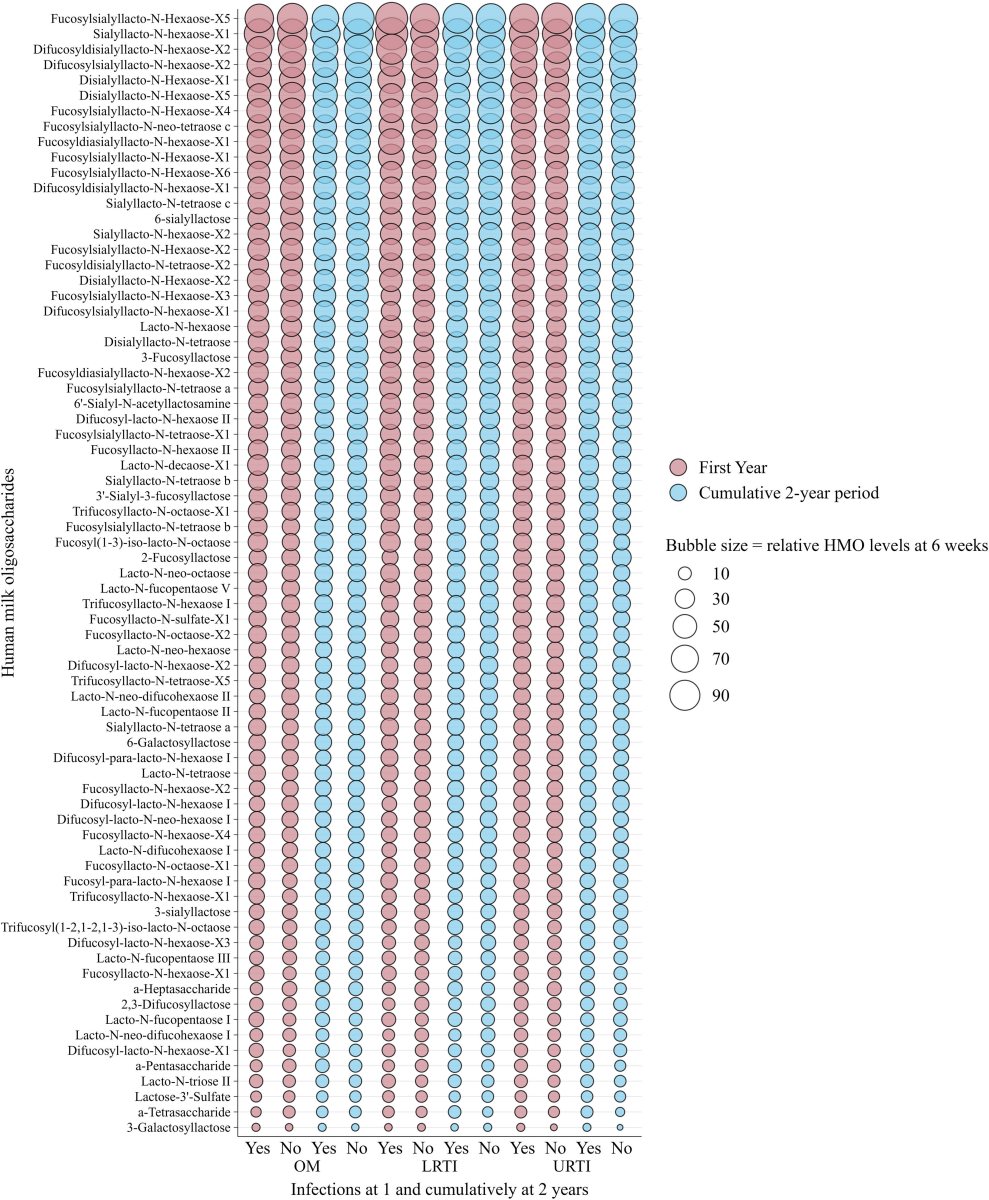
Bubble plot showing the weighted relative means of 71 human milk oligosaccharide (HMO) levels quantified from human milk sampled at 6 weeks of lactation, stratified by infections in infants in the first and cumulatively up to the second year of life in the Ulm SPATZ Health Study. Bubble size reflects relative levels of HMO structures calculated from HMO-specific calibration functions and serves as a descriptive visualisation of the dataset and does not depict statistical differences. HMO, human milk oligosaccharides; OM, otitis media; LRTI, lower respiratory tract infections; URTI, upper respiratory tract infections.

**Figure 3:**
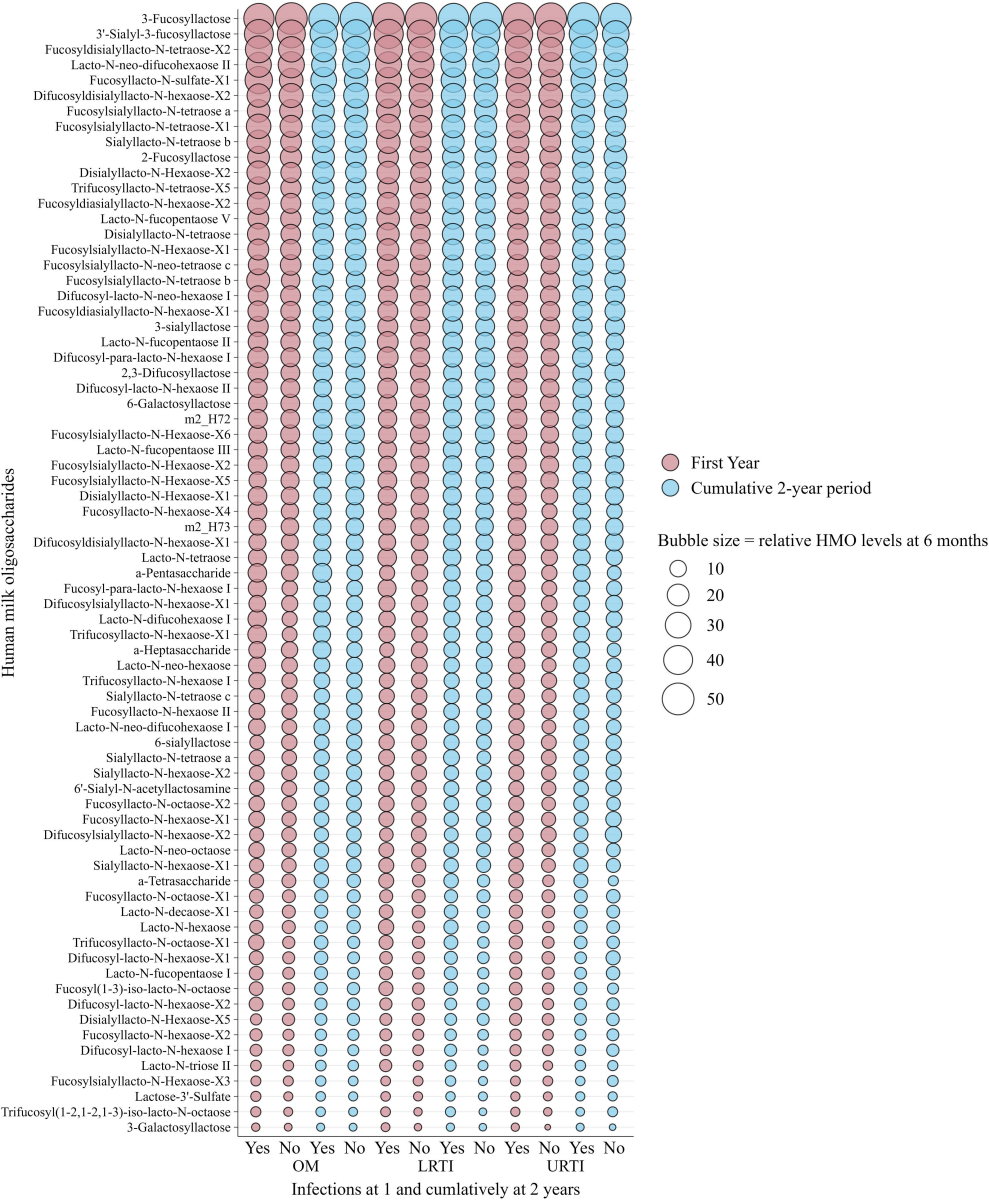
Bubble plot showing weighted relative means of 71 human milk oligosaccharide levels quantified from human milk sampled at 6 months of lactation and infections in infants in the first and cumulatively up to the second year of life in the Ulm SPATZ Health Study. Bubble size reflects relative levels of HMO structures calculated from HMO-specific calibration functions and serves as a descriptive visualisation of the dataset and does not depict statistical differences. HMO, human milk oligosaccharides; OM, otitis media; LRTI, lower respiratory tract infections; URTI, upper respiratory tract infections.

**Figure 2** and **Figure 3** show the relative abundance of the 71 individual HMOs at 6 weeks and 6 months in relation to infection groups during the first year and cumulatively up to 2 years. These are intended as descriptive visualizations of the dataset and not inferential statistics; thus, corresponding numerical values and statistical differences are shown in **Supplementary Tables S1**–**S4**. Overall, in the non-stratified analysis, and after correction for multiple testing (FDR < 0.010), there were no statistically significant differences between the mean levels of individual HMO structures measured at 6 weeks (**Figure 2**) and 6 months (**Figure 3**) in the milk of children with and without infections in the first or cumulatively up to the second year of life.

Although some differences were observed in both known and newly identified HMO structures at 6 weeks (**Supplementary Tables S1**, **S2**) and 6 months (**Supplementary Tables S3**, **S4**), these differences were only statistically significant at the nominal level (p < 0.050) and did not remain significant after FDR correction (FDR < 0.010). Similarly, the mean levels of HMOs at 6 weeks (**Supplementary Tables S5**–**S7**) and 6 months (**Supplementary Tables S8**–**S10**) in secretor and non-secretor milk showed no statistically significant differences between children with and without infections after correction for multiple testing (FDR <0.010).

### 3.2 Associations between HMO levels measured at 6 weeks and infections

**Figure 4:**
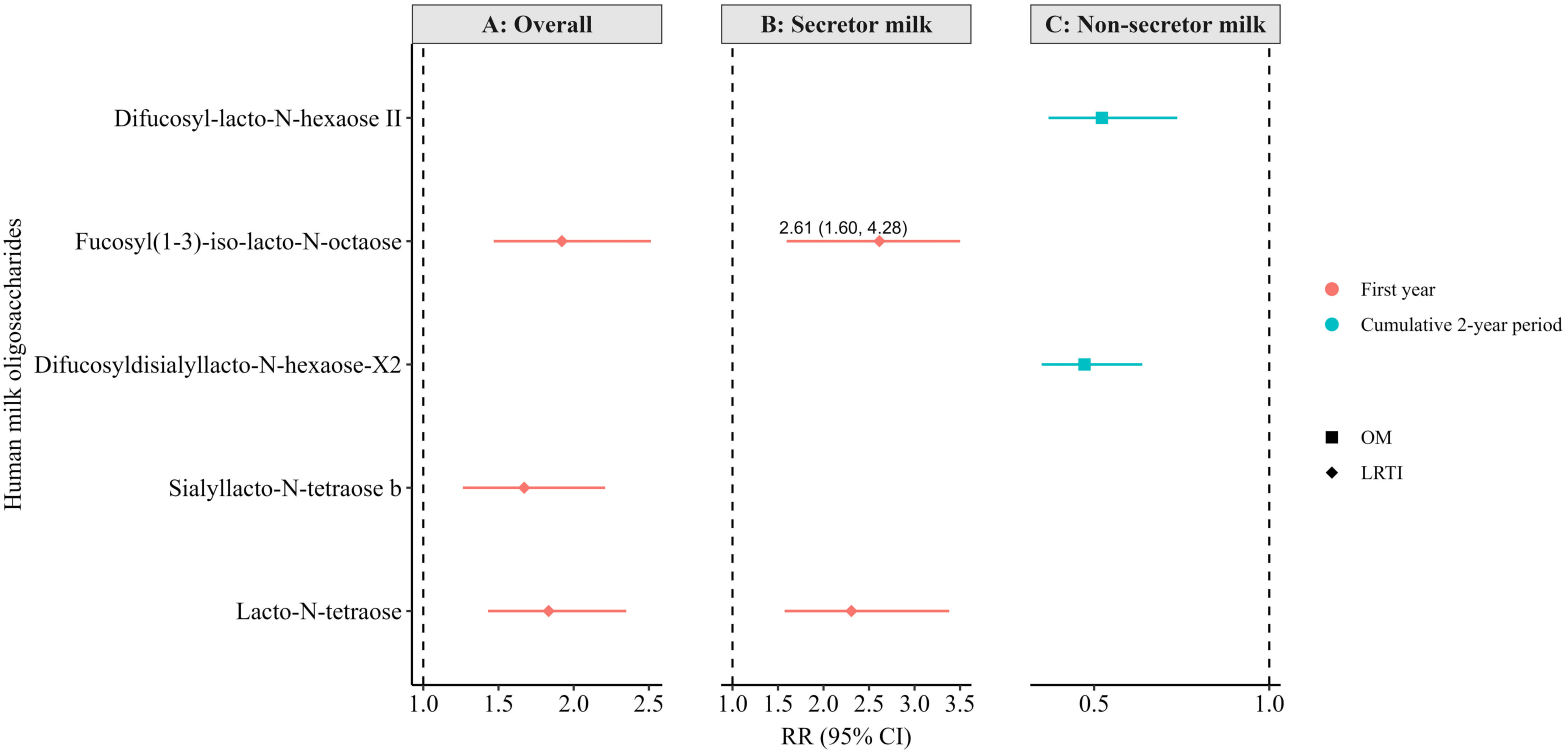
Adjusted associations between human milk oligosaccharides in overall (non-stratified) **(A)**, secretor **(B)**, and non-secretor milk **(C)** measured at 6 weeks of lactation and infections in the first or cumulatively up to the second year of life in the Ulm SPATZ Health Study. RR estimates for panel A were weighted to account for the overrepresentation of non-secretors in the human milk subset, ensuring results reflected the intended 80:20 secretor: non-secretor distribution. All associations shown here were statistically significant following correction for multiple testing (FDR <0.010). Associations determined by modified Poisson regression. Models adjusted for batch, child sex, duration of exclusive breastfeeding (weeks), and gestation age (weeks). OM, otitis media; LRTI, lower respiratory tract infections; RR, risk ratio; CI, confidence intervals.

Following correction for multiple testing (FDR < 0.010), very few statistically significant associations (RR [95% CI]) were observed between the 6-week HMO levels and infection outcomes in both crude (**Supplementary Figure S1**) and adjusted (**Figure 4**) models. For instance, higher levels of sialyllacto-N-tetraose b (LSTb), fucosyl (1-3)-iso-lacto-N-octaose, and lacto-N-tetraose (LNT)—overall and in secretor milk (fucosyl (1-3)-iso-lacto-N-octaose and LNT only)—were associated with a higher likelihood of LRTI in the first year in the crude models (**Supplementary Figures S1A, B**). These associations also remained statistically significant in the adjusted models (**Figures 4A, B**).

Based on the crude models (**Supplementary Figure S1C**), higher levels of 6-galactosyllactose (6′-GL) and fucosyl-para-lacto-N-hexaose I in non-secretor milk were associated with a higher likelihood of OM cumulatively up to the second year of life. On the contrary, higher levels of 3-fucosyllactose (3′-FL), difucosyl-lacto-N-hexaose II, difucosyldisialyllacto-N-hexaose-X2, fucosylsialyllacto-N-tetraose a, lacto-N-fucopentaose II (LNFPI II), and lacto-neo-difucohexaose II in non-secretor milk were associated with a lower likelihood of OM cumulatively up to the second year. Only the associations with difucosyl-lacto-N-hexaose II and difucosyldisialyllacto-N-hexaose-X2 remained statically significant in the adjusted models and following correction for multiple testing (**Figure 4C**). Notably, several other associations were statistically significant at the conventional level (p < 0.05) but did not reach the level of statistical significance after correction for multiple testing in the adjusted models (FDR < 0.010; **Supplementary Tables S11**–**S16**).

### 3.3 Associations between HMO levels measured at 6 months and infections

**Figure 5:**
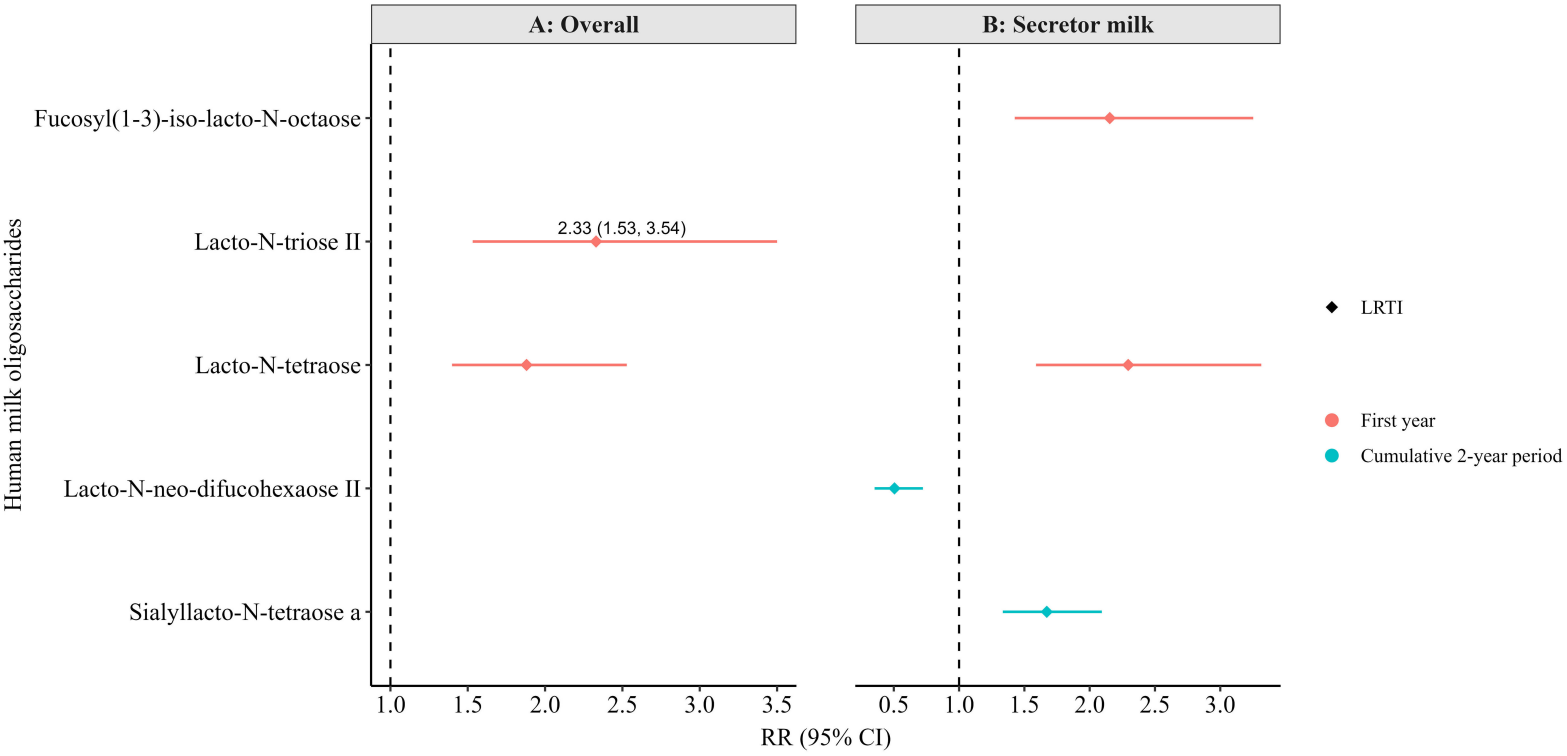
Adjusted associations between human milk oligosaccharides in overall (non-stratified) **(A)** and secretor milk **(B)** measured at 6 months of lactation with infections in the first or cumulatively up to the second year of life in the Ulm SPATZ Health Study. All associations shown were statistically significant after correction for multiple testing (FDR<0.010). RR estimates for panel A were weighted to account for the overrepresentation of non-secretors in the human milk subset, ensuring that results reflected the intended 80:20 secretor: non-secretor distribution. Associations determined by modified Poisson regression. Models adjusted for batch, child sex, duration of exclusive breastfeeding (weeks), and gestation age (weeks). OM, otitis media; LRTI, lower respiratory tract infections; weighted RR, risk ratio determined using weighted secretor status; CI, confidence intervals.

In the 6-month subset, no associations in the crude model reached the statistical level of significance after correction for multiple testing (FDR <0.010). However, a few associations became statistically significant in the adjusted models, ever after correction for multiple testing (FDR <0.010, **Figure 5**). Specifically, higher levels of LNT2 and LNT overall (**Figure 5A**), and higher levels of fucosyl (1-3)-iso-lacto-N-octaose and LNT in secretor milk (**Figure 5B**), were associated with a greater likelihood of LRTI in the first year of life.

Additionally, in secretor milk, higher levels of lacto-N-neo-difucohexaose II (LNnDFH II) were associated with a lower likelihood, and higher levels of sialyllacto-N-tetraose a (LSTa) with a higher likelihood, of LRTI up to the second year of life (**Figure 5B**). Notably, while these associations were statistically significant after FDR correction in the adjusted models, they only reached the conventional level of statistical significance (p<0.05) in the crude models (not shown) and did not survive FDR correction. Still, several other associations were statistically significant at the conventional level (p < 0.050) in the adjusted models (**Supplementary Tables S16**-**S22**), although they also did not pass FDR correction.

## 4 Discussion

This study investigated associations between 71 known and novel HMOs measured at 6 weeks and/or 6 months and the occurrence of OM, LRTI, and URTI during the first 2 years of life in a birth cohort study. Our approach in this study was to capture the diversity of HMO structures, in order to better understand the complex interdependent linkages within human milk, rather than assuming that the analysis of a greater number of HMOs would yield inherently stronger evidence. On one hand, higher levels of fucosyl(1-3)-iso-lacto-N-octaose, LSTb, and LNT at 6 weeks overall—and in secretor milk (only LNT)—were linked to a higher likelihood to LRTI in the first year. On the other hand, elevated levels of difucosyl-lacto-N-hexaose II and difucosyldisiallacto-N-hexaose-X2 in non-secretor milk were associated with a lower likelihood of OM over the cumulative period up to the second year of life. At 6 months, higher LNT2 (overall) and LSTa (in secretor milk) were associated with higher likelihood for LRTI in the first and across the cumulative 2-year period, respectively, whereas lacto-N-neo-difucohexaose II l (LNnDFH II) in secretor milk was linked to a lower likelihood of LRTI over the same cumulative period. These associations were statistically significant after adjustment for confounders and correction for multiple testing (FDR < 0.010), highlighting the relevance for further research into the role of these HMOs in infections and immune development. It is important to note that the direction and the effect sizes of these associations were moderate, which suggests that while some HMOs may influence infection susceptibility, these associations are likely multifactorial or context-dependent and not directly causal.

Firstly, the findings that some HMOs were linked with higher likelihood of infections is in contrast with preclinical evidence, suggesting that HMOs promote beneficial gut microbiota and enhance mucosal defense (31–34). This contrast in results suggests that the relationship between HMOs and susceptibility to infections may be context-dependent rather than strictly protective. Given that gut microbiota can influence immune responses in the respiratory system through the gut–lung axis (35), the observed associations may reflect a more complex, context-dependent role of HMOs in systemic immune modulation and respiratory health. However, as we do not have data on immune parameters or intestinal and respiratory microbiota in this study, our mechanistic explanations are speculative and should be interpreted with caution. Thus, future studies should integrate immune markers and microbiota profiling to better understand the role of these HMOs in respiratory health.

Secondly, previous studies have linked higher levels of LNT to neurodevelopment (36) and cognition (37, 38), but emerging evidence emphasizes the importance of human milk as a complex system rather than focusing on individual components like specific individual HMOs (39). A recent study (18) also highlighted that variations in HMO profiles, influenced by genes like *FUT2*, may impact respiratory health, underscoring the need to consider both maternal and infant genetics in immune development. Our findings therefore support this comprehensive view, suggesting that the overall composition of HMOs, and their overall balance, may better explain the observed patterns of infections. Thirdly, prior hypotheses suggest that LNT2 plays a protective role in promoting bacterial adhesion (40), reducing inflammation (41), and improving gut barrier function (42). While LNT2 potentially supports the adhesion of beneficial bacteria such as *Lactobacillus plantarum* in the gut, these effects may not directly translate into similar benefits in other tissues, i.e., lung tissue. Furthermore, LNT2 is present in human milk in low levels, but it can be produced, e.g., by acid hydrolysis in the stomach or in the infant gut through fermentation of LNT and lacto-N-neotetraose (LNnT) (9). Links between gut microbiota composition and respiratory infections including bronchitis and LRTIs (43) further highlight the complexity of gut–lung interactions. Given these interlinked processes, the associations we observed in this study for LNT2 may likely reflect indirect immunomodulatory or microbial effects rather than a direct causal pathway. Still, the systemic immunomodulatory roles of LNT2 remain unclear, hence the need for more targeted research on its role across different mucosal sites and its impact on susceptibility to infection.

Our findings further suggest that sialylated HMOs (e.g., LSTb and LSTa) (1, 44, 45) may influence susceptibility to primary respiratory infections during early life, potentially shaping the trajectory of immune development. However, the observed associations with a higher likelihood of LRTI cumulatively up to the second year of life may instead reflect early microbial exposures and immune responses triggered by initial infections, rather than a direct effect of HMOs. To avoid overinterpretation, our results should be viewed as associations that simply identify candidate HMOs for further mechanistic studies, and not as evidence of direct causation. Therefore, further studies that explicitly and accurately document multiple infections over time are needed to determine whether HMOs influence an infant’s susceptibility of acquiring infections or instead modulate immune responses following early infections. Also, reverse causality may be involved with elevated HMO levels potentially reflecting maternal responses to early or subclinical infections, similar to the shifts observed in antibody profiles.

Moreover, very few studies have directly linked HMOs to OM, where prior research has reported non-statistically significant associations between other HMOs and OM (14, 17). Granted that OM is often a secondary complication of viral URTIs (46, 47), it is plausible that HMOs may indirectly influence OM susceptibility by modulating primary susceptibility to respiratory infections. However, the infection variables in our study do not explicitly show the sequence of events, i.e., if OM was first or not, and we do not have data for when exactly the infection occurred. Still, the result that higher levels of difucosyl-lacto-N-hexaose II and difucosyldisiallacto-N-hexaose-X2 in non-secretor milk were associated with a lower likelihood of OM within the first two years suggests that despite the absence of certain fucosylated oligosaccharides like 2′-FL in the milk (48, 49), non-secretor milk may still confer protection through alternative HMOs and overall human milk provision (50–52). Still, our findings, although preliminary, may have potential clinical relevance by identifying non-secretor HMO profiles that are potentially linked to reduced susceptibility to OM in early childhood.

These findings also align with broader evidence supporting the protective associations of breastfeeding against infections in childhood (53). Thus, while our data highlight the potential immunological relevance of specific HMOs, it is also essential to recognize the overall benefit of exclusive breastfeeding (EBF), which provides consistent exposure to a full range of bioactive components. In contrast, mixed milk feeding (MMF) introduces variability in HMO exposure, potentially attenuating the protective associations observed in infants receiving exclusive human milk. With increasing age, the number of MMF-fed infants typically increases, which may partly explain why the HMO-related associations detected with milk samples at 6 weeks were not similar to the associations with milk samples collected at 6 months. Clinically, this observation reinforces existing recommendations supporting EBF during early infancy, as consistent exposure to the full HMO profile may be important for immune development of the infant. Understanding these interactions is crucial for elucidating how both individual HMOs and overall breastfeeding practices contribute to infant immunity and infection susceptibility.

Ultimately, the associations of specific HMOs with higher likelihood of LRTI do not necessarily suggest that certain HMOs are inherently harmful but rather reflect a complex interplay, as HMOs share common biosynthetic pathways (54). A decrease in one may correspond to an increase in another, indicating a natural balance rather than an adverse effect (54). This extends to infection susceptibility, where an HMO linked to high likelihood of infection may be part of a broader composition that influences immune function and microbiota development. Furthermore, while several other associations reached the conventional level of statistical significance (p < 0.050), these results should be interpreted with caution. Also, several crude associations lost statistical significance in adjusted models following correction for multiple testing (FDR <0.010), suggesting that the findings may reflect shifts in immune development rather than direct effect on infections. We also acknowledge that HMOs function within a network of complex glycans and that synergistic or interacting effects could be likely. However, in this study, we analyzed individual HMOs to identify specific candidates linked to infection outcomes and adjusted for confounders and multiple testing to limit spurious conclusions. This exploratory and hypothesis-generating approach was used to maintain statistical stability and interpretability, given the small subgroup sample sizes, and the high number of HMOs measured in this study. Additionally, factors like infection timing and secretor status further influence these interactions, emphasizing the need for a holistic approach in interpreting HMO–infection relationships. Consequently, these associations should be interpreted as initial signals within a complex compositional framework rather than as evidence of isolated effects. As part of a secondary analysis, normalizing HMOs post-stratification showed similar associations with narrower confidence intervals **Supplementary Tables S23**–**S28**, and **Supplementary Figures S2**, **S3**) but showed an additional association between higher 3′-galactosyllactose (3′-GL) in non-secretor milk and a greater likelihood of OM (**Supplementary Figure S4**). Together, these preliminary findings highlight candidate HMOs and structural patterns that can be taken forward for further investigation in larger and targeted studies that can formerly model HMO networks and synergistic effects.

Several limitations should be acknowledged. The small sample size and observational design limit causal inference. While HMO levels were measured at 6 weeks and 6 months, adding evidence to previous studies, the exact timing of infections is unknown, and some may have occurred before or at the time at which human milk was collected. Additionally, infection types were recorded retrospectively, but this only provides a rough estimate of the infection burden. Furthermore, the definition of infections in our study were based on physician-reported reports without clinical verification or review of medical records. Although the questionnaires used in our study were standardized, captured doctor diagnoses of infections and potentially reduce bias from parental self-reporting, we still cannot rule out the possibility of some diagnostic misclassification. This is particularly important to note given the challenges of distinguishing between LRTI and URTI in primary care settings. Nevertheless, it is likely to result in non-differential misclassification, which would bias estimates toward the null rather than creating spurious associations. These factors caution against drawing definitive conclusions on cause and effect or timing of immune protection. The selective sampling of short and long-term breastfeeding mothers as well as infants with specific disease outcomes may have introduced biases, limiting the generalizability to all breastfeeding mothers and infants. Future studies should include a more diverse breastfeeding population. Other factors, such as infant microbiome, genetics, and environmental exposures, may also influence these associations. Mode of delivery was excluded from the adjusted models because of the small subgroup sample sizes. However, we acknowledge that antibiotic use is strongly associated with delivery mode, but given the low numbers of caesarean section deliveries in this study, substantial residual confounding is unlikely. We adjusted for key confounders including breastfeeding duration, gestational age, and child sex, to minimize known confounding effects. Still, while we adjusted for these key confounders, we cannot rule out some residual confounding. Larger studies are needed to confirm these findings and consider additional parameters not accounted for here. We report relative HMO levels, which may not reflect absolute concentrations. However, this study represents the largest quantification of HMOs using advanced LC-ESI-IM-QTOF-MS techniques (19). While our study adds to the evidence on HMOs and infection susceptibility, their biological mechanisms are still not fully understood.

## 5 Conclusion

This study offers new insights into how specific HMOs may be linked to susceptibility to infections in early childhood, despite no overall differences in HMO levels between infected and non-infected groups. These associations should be interpreted as potential indicators of underlying biological processes rather than direct causal effects, as they likely reflect a dynamic, adaptive balance in human milk composition rather than suggesting certain HMOs are inherently harmful. Reverse causality may also be involved, with elevated HMO levels potentially representing maternal responses to early or subclinical infections, similar to antibody shifts. From a clinical perspective, the findings highlight the need for future studies to investigate absolute concentrations, in combination with immune biomarkers and infant microbiota to provide more light on the mechanisms linking HMOs with infection susceptibility. Factors like infant secretor status, infection timing, and cascading infection patterns (e.g., viral triggers for OM) underscore the need to interpret these findings within a complex, multifactorial non-causal framework. Further research is needed to clarify how HMOs influence infection susceptibility. Future research from larger cohorts and longitudinal microbiome profiling is needed to confirm these findings and define their clinical implications.

## Data Availability

The original contributions presented in the study are included in the article/**Supplementary Material**. Further inquiries can be directed to the corresponding author.
