# Supplementary material for: Associations of known and newly identified human milk oligosaccharides with infections in early childhood: the Ulm SPATZ health study
Source: Front Immunol. 2025 Dec 5;16:1703579. doi: 10.3389/fimmu.2025.1703579 (PMC12714623; doi:10.3389/fimmu.2025.1703579)
Supplement: Supplementary file 1 [file Supplementaryfile1.docx]

**Associations of known and newly identified human milk oligosaccharides with infections in early childhood: The Ulm SPATZ Health Study**

Linda P. Siziba^1,*^, Zhuoxin Peng^1^, Marko Mank^2^, Bernd Stahl^2,3^, John Gonsalves^2^, Deborah Wernecke^4,5^, Dietrich Rothenbacher^4,5^, Jon Genuneit^1,4^

^1^Pediatric Epidemiology, Department of Paediatrics, Medical Faculty, Leipzig University, 04103 Leipzig, Germany

^2^Danone Research & Innovation, 3584 CT Utrecht, The Netherlands

^3^Department of Chemical Biology & Drug Discovery, Faculty of Science, Utrecht Institute for Pharmaceutical Sciences, Utrecht University, 3584 CG Utrecht, The Netherlands

^4^Institute of Epidemiology and Medical Biometry, Ulm University, 89075 Ulm, Germany

^5^German Center for Child and Adolescent Health (DZKJ), partner site Ulm, Ulm, Germany.

***Supplementary Material***

**Table S1**: Weighted mean relative levels of human milk oligosaccharides measured at 6 weeks stratified by otitis media (OM)

|  | **OM at 1 year** | | | **OM at 2 years** | | |
| --- | --- | --- | --- | --- | --- | --- |
| **HMO Structure** | Yes (n=16) | No (n=97) | p | Yes (n=35) | No (n=98) | p |
| 2,3 Difucosyllactose | 11.3 | 11.6 | 0.57 | 11.4 | 11.4 | 0.57 |
| 2-Fucosyllactose | 21.3 | 24.1 | 0.94 | 23.9 | 23.2 | 0.36 |
| 3'-Sialyl-3-fucosyllactose | 23.2 | 28.0 | 0.17 | 23.8 | 29.1 | **0.02** |
| **3-Fucosyllactose** | 30.4 | 34.5 | 0.27 | **28.2** | **36.9** | **0.005** |
| 3-Galactosyllactose | 3.1 | 2.8 | 0.68 | 3.0 | 2.7 | 0.48 |
| 3-sialyllactose | 15.0 | 14.6 | 0.49 | 14.9 | 14.7 | 0.68 |
| 6'-Sialyl-N-acetyllactosamine | 27.0 | 33.6 | 0.37 | 30.5 | 33.1 | 0.66 |
| 6-Galactosyllactose | 19.5 | 19.2 | 0.39 | 19.7 | 18.9 | 0.67 |
| 6-sialyllactose | 39.0 | 43.0 | 0.61 | 40.2 | 43.2 | 0.41 |
| Difucosyl-lacto-N-hexaose I | 16.0 | 18.8 | 0.52 | 19.4 | 17.5 | 0.16 |
| **Difucosyl-lacto-N-hexaose II** | 27.6 | 31.7 | 0.64 | **25.5** | **33.8** | **0.02** |
| Difucosyl-lacto-N-hexaose-X1 | 13.0 | 8.9 | 0.20 | 11.8 | 8.8 | 0.08 |
| Difucosyl-lacto-N-hexaose-X2 | 18.9 | 21.3 | 0.48 | 18.0 | 21.9 | 0.45 |
| Difucosyl-lacto-N-hexaose-X3 | 11.8 | 13.0 | 0.38 | 11.6 | 13.6 | 0.25 |
| Difucosyl-lacto-N-neo-hexaose I | 16.9 | 18.3 | 0.55 | 17.1 | 18.5 | 0.19 |
| Difucosyl-para-lacto-N-hexaose I | 17.3 | 18.5 | 0.87 | 15.9 | 19.9 | 0.10 |
| Difucosyldisialyllacto-N-hexaose-X1 | 39.9 | 46.4 | 0.87 | 44.4 | 45.2 | 0.36 |
| **Difucosyldisialyllacto-N-hexaose-X2** | 58.6 | 73.8 | 0.42 | **57.0** | **78.4** | **0.02** |
| Difucosylsialyllacto-N-hexaose-X1 | 32.0 | 37.8 | 0.75 | 34.4 | 37.6 | 0.40 |
| Difucosylsialyllacto-N-hexaose-X2 | 53.4 | 64.8 | 0.62 | 58.6 | 63.9 | 0.39 |
| Disialyllacto-N-Hexaose-X1 | 55.0 | 61.1 | 0.67 | 53.8 | 62.2 | 0.23 |
| Disialyllacto-N-Hexaose-X2 | 43.1 | 35.8 | 0.62 | 38.2 | 37.4 | 0.38 |
| Disialyllacto-N-Hexaose-X5 | 48.2 | 63.5 | 0.57 | 52.4 | 64.6 | 0.70 |
| Disialyllacto-N-tetraose | 33.0 | 31.2 | 0.94 | 33.0 | 32.0 | 0.91 |
| Fucosyl(1-3)-iso-lacto-N-octaose | 29.3 | 21.7 | 0.14 | 26.1 | 21.5 | 0.08 |
| **Fucosyl-para-lacto-N-hexaose I** | 19.3 | 13.9 | 0.09 | **17.8** | **13.9** | **0.03** |
| Fucosyldiasialyllacto-N-hexaose-X1 | 49.2 | 53.9 | 0.33 | 51.3 | 53.6 | 0.51 |
| Fucosyldiasialyllacto-N-hexaose-X2 | 32.8 | 31.7 | 0.68 | 32.7 | 32.2 | 0.90 |
| **Fucosyldisialyllacto-N-tetraose-X2** | 32.0 | 41.0 | 0.28 | **32.0** | **43.6** | **0.03** |
| Fucosyllacto-N-hexaose II | 30.2 | 28.7 | 0.77 | 29.3 | 28.7 | 0.76 |
| Fucosyllacto-N-hexaose-X1 | 15.8 | 10.8 | 0.37 | 12.8 | 11.3 | 0.25 |
| Fucosyllacto-N-hexaose-X2 | 16.0 | 18.7 | 0.63 | 17.4 | 18.4 | 0.24 |
| Fucosyllacto-N-hexaose-X4 | 18.2 | 17.0 | 0.23 | 15.5 | 18.0 | 0.54 |
| Fucosyllacto-N-octaose-X1 | 16.1 | 16.6 | 0.48 | 15.6 | 16.8 | 0.22 |
| Fucosyllacto-N-octaose-X2 | 23.4 | 21.3 | 0.13 | 20.6 | 22.1 | 0.53 |
| Fucosyllacto-N-sulfate-X1 | 21.6 | 23.5 | 0.57 | 22.0 | 23.5 | 0.20 |
| Fucosylsialyllacto-N-Hexaose-X1 | 49.7 | 51.2 | 0.71 | 48.6 | 51.9 | 0.48 |
| Fucosylsialyllacto-N-Hexaose-X2 | 39.5 | 41.2 | 0.55 | 44.2 | 38.8 | 0.11 |
| Fucosylsialyllacto-N-Hexaose-X3 | 32.9 | 40.3 | 0.64 | 42.4 | 36.8 | 0.27 |
| Fucosylsialyllacto-N-Hexaose-X4 | 53.1 | 59.5 | 0.69 | 52.9 | 60.8 | 0.49 |
| **Fucosylsialyllacto-N-Hexaose-X5** | 79.5 | 89.9 | 0.66 | **66.6** | **98.5** | **0.01** |
| Fucosylsialyllacto-N-Hexaose-X6 | 43.0 | 47.8 | 0.67 | 42.0 | 49.5 | 0.83 |
| **Fucosylsialyllacto-N-neo-tetraose c** | 47.4 | 58.2 | 0.26 | **44.9** | **62.2** | **0.01** |
| Fucosylsialyllacto-N-tetraose a | 29.9 | 32.6 | 0.83 | 27.9 | 34.6 | 0.09 |
| Fucosylsialyllacto-N-tetraose b | 26.4 | 22.4 | 0.19 | 24.8 | 22.3 | 0.10 |
| Fucosylsialyllacto-N-tetraose-X1 | 29.0 | 30.0 | 0.48 | 27.6 | 31.2 | 0.06 |
| Lacto-N-decaose-X1 | 35.4 | 27.7 | 0.17 | 30.7 | 27.5 | 0.11 |
| Lacto-N-difucohexaose I | 15.8 | 15.9 | 0.42 | 16.1 | 15.7 | 0.30 |
| Lacto-N-fucopentaose I | 14.5 | 10.1 | 0.24 | 12.9 | 9.9 | 0.09 |
| **Lacto-N-fucopentaose II** | 18.9 | 19.5 | 0.79 | **16.6** | **21.1** | **0.04** |
| Lacto-N-fucopentaose III | 12.1 | 12.4 | 0.55 | 12.0 | 12.6 | 0.39 |
| Lacto-N-fucopentaose V | 23.1 | 22.8 | 0.96 | 20.6 | 24.4 | 0.19 |
| Lacto-N-hexaose | 37.9 | 33.6 | 0.26 | 37.6 | 31.8 | 0.22 |
| Lacto-N-neo-difucohexaose I | 10.1 | 10.8 | 0.65 | 9.9 | 11.0 | 0.42 |
| **Lacto-N-neo-difucohexaose II** | 16.4 | 21.4 | 0.28 | **15.6** | **23.3** | **0.01** |
| Lacto-N-neo-hexaose | 20.5 | 22.1 | 0.25 | 18.4 | 23.2 | 0.34 |
| Lacto-N-neo-octaose | 26.9 | 22.8 | 0.22 | 24.1 | 23.0 | 0.07 |
| Lacto-N-tetraose | 20.6 | 16.5 | 0.10 | 18.8 | 16.7 | 0.26 |
| Lacto-N-triose II | 10.9 | 9.1 | 0.25 | 10.0 | 9.0 | 0.50 |
| Lactose-3'-Sulfate | 6.4 | 7.4 | 0.22 | 7.2 | 7.2 | 0.61 |
| Sialyllacto-N-hexaose-X1 | 88.9 | 85.6 | 0.62 | 85.1 | 85.6 | 0.76 |
| Sialyllacto-N-hexaose-X2 | 38.0 | 43.5 | 0.40 | 36.6 | 45.1 | 0.37 |
| Sialyllacto-N-tetraose a | 21.8 | 17.4 | 0.20 | 21.4 | 17.0 | 0.05 |
| Sialyllacto-N-tetraose b | 28.9 | 26.0 | 0.65 | 27.3 | 26.7 | 0.93 |
| Sialyllacto-N-tetraose c | 38.8 | 43.7 | 0.88 | 39.6 | 44.4 | 0.74 |
| Trifucosyl(1-2,1-2,1-3)-iso-lacto-N-octaose | 15.4 | 13.5 | 0.43 | 16.0 | 12.7 | 0.14 |
| Trifucosyllacto-N-hexaose I | 22.5 | 23.4 | 0.45 | 23.8 | 22.4 | 0.23 |
| Trifucosyllacto-N-hexaose-X1 | 16.4 | 14.3 | 0.18 | 15.4 | 14.4 | 0.16 |
| Trifucosyllacto-N-octaose-X1 | 28.9 | 24.3 | 0.23 | 26.8 | 23.9 | 0.17 |
| Trifucosyllacto-N-tetraose-X5 | 18.7 | 19.9 | 0.31 | 20.2 | 19.3 | 0.31 |
| a-Heptasaccharide | 8.9 | 14.2 | 0.55 | 14.4 | 12.5 | 0.55 |
| a-Pentasaccharide | 8.3 | 11.2 | 0.38 | 11.7 | 9.9 | 0.51 |
| a-Tetrasaccharide | 5.4 | 8.3 | 0.62 | 7.5 | 7.9 | 0.75 |

p values derived from Wilcoxon sum-rank test comparing HMO levels between infants with and without otitis media at 1 and/or at 2 years. **Bold** denotes statistically significant values at nominal level (p <0.05). None of the difference shown were significant following correction for multiple testing (False discovery rate, FDR <0.010). OM: Otitis media; HMO: human milk oligosaccharides

.

**Table S2**: Weighted mean relative levels of human milk oligosaccharides measured at 6 weeks stratified by lower and upper respiratory tract infections at one and two years

|  | **LRTI at 1 year** | | | **LRTI at 2 years** | | | **URTI at 1 year** | | | **URTI at 2 years** | | |
| --- | --- | --- | --- | --- | --- | --- | --- | --- | --- | --- | --- | --- |
| **HMO Structure** | Yes (n=37) | No (n=78) | p | Yes (n=57) | No (n=64) | p | Yes (n=80) | No (n=38) | p | Yes (n=104) | No (n=19) | p |
| 2,3 Difucosyllactose | 9.1 | 12.8 | 0.06 | 9.4 | 13.7 | 0.09 | 12.2 | 10.9 | 0.73 | 11.7 | 11.5 | 0.87 |
| **2-Fucosyllactose** | **18.2** | **24.9** | **0.02** | 22.4 | 24.8 | 0.28 | 23.2 | 24.5 | 0.72 | 22.7 | 28.0 | 0.30 |
| 3'-Sialyl-3-fucosyllactose | 31.1 | 27.1 | 0.23 | 27.7 | 27.1 | 0.72 | 28.4 | 25.8 | 0.47 | 28.5 | 21.6 | 0.16 |
| 3-Fucosyllactose | 34.6 | 33.9 | 0.82 | 31.4 | 34.8 | 0.47 | 32.9 | 34.3 | 0.72 | 33.9 | 31.3 | 0.80 |
| **3-Galactosyllactose** | 2.8 | 3.0 | 0.58 | 2.7 | 3.1 | 0.93 | **3.1** | **2.4** | **0.01** | 3.1 | 2.3 | 0.06 |
| 3-sialyllactose | 15.9 | 14.5 | 0.28 | 15.1 | 14.7 | 0.28 | 15.4 | 14.0 | 0.34 | 15.0 | 13.9 | 0.45 |
| 6'-Sialyl-N-acetyllactosamine | 32.3 | 32.4 | 0.82 | 31.4 | 32.9 | 0.76 | 33.4 | 30.3 | 0.44 | 32.4 | 30.9 | 0.64 |
| 6-Galactosyllactose | 17.7 | 20.0 | 0.99 | 17.0 | 21.2 | 0.31 | 19.6 | 18.1 | 0.35 | 19.3 | 18.5 | 0.30 |
| 6-sialyllactose | 40.4 | 42.4 | 0.69 | 40.5 | 42.6 | 0.67 | 42.6 | 40.9 | 0.75 | 41.6 | 42.4 | 0.87 |
| Difucosyl-lacto-N-hexaose I | 14.1 | 18.0 | 0.10 | 18.3 | 17.6 | 0.62 | 18.2 | 17.5 | 0.91 | 17.5 | 18.9 | 0.71 |
| Difucosyl-lacto-N-hexaose II | 37.1 | 29.7 | 0.25 | 30.0 | 31.2 | 0.61 | 29.6 | 32.8 | 0.55 | 31.5 | 26.3 | 0.55 |
| Difucosyl-lacto-N-hexaose-X1 | 10.4 | 10.0 | 0.17 | 10.2 | 10.3 | 0.69 | 10.8 | 8.9 | 0.66 | 10.1 | 10.0 | 0.81 |
| Difucosyl-lacto-N-hexaose-X2 | 18.4 | 21.5 | 0.14 | 17.7 | 23.2 | 0.27 | 21.2 | 19.5 | 0.67 | 20.0 | 22.8 | 0.95 |
| **Difucosyl-lacto-N-hexaose-X3** | 11.0 | 14.0 | 0.05 | **10.9** | **14.9** | **0.02** | 13.7 | 12.2 | 0.10 | 13.7 | 10.1 | 0.01 |
| **Difucosyl-lacto-N-neo-hexaose I** | 16.4 | 18.4 | 0.43 | **15.7** | **20.0** | **0.03** | 18.0 | 18.1 | 0.20 | 18.5 | 15.0 | 0.49 |
| Difucosyl-para-lacto-N-hexaose I | 21.0 | 18.4 | 0.26 | 17.9 | 19.1 | 1.00 | 19.2 | 17.5 | 0.27 | 19.4 | 14.9 | 0.13 |
| Difucosyldisialyllacto-N-hexaose-X1 | 39.8 | 45.4 | 0.08 | 41.1 | 47.4 | 0.33 | 46.5 | 41.7 | 0.76 | 43.8 | 45.3 | 0.68 |
| Difucosyldisialyllacto-N-hexaose-X2 | 90.4 | 65.7 | 0.06 | 70.0 | 70.0 | 0.94 | 65.6 | 77.0 | 0.50 | 70.3 | 64.8 | 0.51 |
| Difucosylsialyllacto-N-hexaose-X1 | 28.6 | 38.8 | 0.05 | 30.9 | 40.9 | 0.19 | 38.6 | 33.0 | 0.68 | 36.3 | 34.7 | 0.81 |
| Difucosylsialyllacto-N-hexaose-X2 | 47.1 | 67.1 | 0.05 | 49.1 | 72.1 | 0.12 | 62.9 | 60.8 | 0.83 | 59.2 | 71.7 | 0.74 |
| Disialyllacto-N-Hexaose-X1 | 70.2 | 54.8 | 0.55 | 60.5 | 57.4 | 0.76 | 64.1 | 48.8 | 0.63 | 59.3 | 53.4 | 1.00 |
| Disialyllacto-N-Hexaose-X2 | 44.9 | 36.7 | 0.95 | 37.2 | 39.3 | 0.58 | 41.5 | 32.5 | 0.18 | 39.1 | 32.4 | 0.22 |
| Disialyllacto-N-Hexaose-X5 | 50.0 | 63.0 | 0.54 | 53.0 | 66.0 | 0.20 | 61.3 | 58.1 | 0.89 | 60.4 | 56.7 | 0.52 |
| Disialyllacto-N-tetraose | 42.6 | 30.8 | 0.06 | 34.1 | 32.7 | 0.83 | 34.0 | 32.6 | 0.63 | 33.3 | 31.8 | 0.24 |
| **Fucosyl(1-3)-iso-lacto-N-octaose** | **33.3** | **19.5** | **0.01** | 26.9 | 19.9 | 0.13 | 24.2 | 21.3 | 0.51 | 24.1 | 17.6 | 0.11 |
| Fucosyl-para-lacto-N-hexaose I | 19.6 | 14.1 | 0.16 | 16.4 | 14.6 | 0.47 | 16.2 | 14.0 | 0.16 | 15.8 | 13.1 | 0.02 |
| Fucosyldiasialyllacto-N-hexaose-X1 | 60.4 | 49.6 | 0.09 | 52.9 | 52.0 | 0.96 | 52.0 | 53.6 | 0.43 | 53.0 | 46.7 | 0.71 |
| Fucosyldiasialyllacto-N-hexaose-X2 | 36.1 | 33.7 | 0.65 | 31.2 | 36.2 | 0.57 | 35.6 | 30.9 | 0.21 | 34.7 | 27.9 | 0.02 |
| Fucosyldisialyllacto-N-tetraose-X2 | 50.0 | 38.3 | 0.06 | 40.0 | 40.2 | 0.67 | 40.8 | 38.9 | 0.44 | 40.8 | 35.6 | 0.14 |
| **Fucosyllacto-N-hexaose II** | **35.3** | **26.4** | **0.02** | 30.5 | 27.3 | 0.38 | 28.5 | 29.4 | 0.81 | 29.4 | 24.8 | 0.30 |
| Fucosyllacto-N-hexaose-X1 | 14.4 | 11.3 | 0.31 | 12.2 | 11.8 | 0.86 | 13.0 | 9.9 | 0.87 | 12.1 | 10.6 | 0.63 |
| Fucosyllacto-N-hexaose-X2 | 16.6 | 17.8 | 0.18 | 18.3 | 17.7 | 0.74 | 18.3 | 17.1 | 0.83 | 17.6 | 18.8 | 0.65 |
| **Fucosyllacto-N-hexaose-X4** | 17.2 | 17.7 | 0.71 | **15.0** | **19.3** | **0.05** | 18.4 | 15.2 | 0.08 | 17.9 | 14.1 | 0.05 |
| Fucosyllacto-N-octaose-X1 | 15.7 | 16.1 | 0.40 | 15.9 | 16.6 | 0.93 | 17.5 | 13.7 | 0.75 | 16.5 | 14.0 | 1.00 |
| Fucosyllacto-N-octaose-X2 | 24.2 | 20.9 | 0.27 | 21.0 | 22.5 | 0.56 | 23.3 | 18.5 | 0.21 | 22.5 | 16.5 | 0.15 |
| Fucosyllacto-N-sulfate-X1 | 23.9 | 23.5 | 0.10 | 23.3 | 23.3 | 0.45 | 25.2 | 20.3 | 0.68 | 24.0 | 19.0 | 0.90 |
| **Fucosylsialyllacto-N-Hexaose-X1** | **62.0** | **46.8** | **0.04** | 52.8 | 48.5 | 0.65 | 50.9 | 49.2 | 0.80 | 52.0 | 41.4 | 0.26 |
| Fucosylsialyllacto-N-Hexaose-X2 | 41.1 | 39.1 | 0.35 | 40.7 | 40.5 | 0.87 | 40.0 | 42.1 | 0.16 | 40.8 | 37.3 | 0.81 |
| **Fucosylsialyllacto-N-Hexaose-X3** | **30.4** | **38.9** | **0.05** | 38.6 | 36.9 | 0.40 | 38.4 | 37.2 | 0.92 | 36.2 | 43.3 | 0.59 |
| Fucosylsialyllacto-N-Hexaose-X4 | 49.4 | 60.8 | 0.31 | 52.1 | 62.8 | 0.08 | 58.5 | 57.5 | 0.82 | 59.0 | 52.1 | 0.19 |
| Fucosylsialyllacto-N-Hexaose-X5 | 104.0 | 83.0 | 0.12 | 87.6 | 85.5 | 0.86 | 81.3 | 94.6 | 0.54 | 87.7 | 79.7 | 0.77 |
| Fucosylsialyllacto-N-Hexaose-X6 | 41.4 | 50.0 | 0.24 | 40.2 | 53.2 | 0.09 | 50.6 | 41.4 | 0.08 | 49.0 | 37.7 | 0.04 |
| Fucosylsialyllacto-N-neo-tetraose c | 54.8 | 57.9 | 0.51 | 51.6 | 59.9 | 0.36 | 56.5 | 55.3 | 0.13 | 57.8 | 48.6 | 0.03 |
| Fucosylsialyllacto-N-tetraose a | 40.6 | 31.0 | 0.06 | 31.9 | 33.3 | 0.89 | 31.9 | 34.4 | 0.46 | 33.4 | 28.0 | 0.58 |
| Fucosylsialyllacto-N-tetraose b | 25.2 | 24.1 | 0.36 | 23.3 | 25.1 | 0.72 | 25.8 | 21.6 | 0.57 | 24.4 | 22.4 | 0.94 |
| **Fucosylsialyllacto-N-tetraose-X1** | 32.3 | 30.0 | 0.36 | 29.1 | 31.1 | 0.63 | **31.7** | **27.3** | **0.05** | **31.2** | **24.3** | **0.01** |
| Lacto-N-decaose-X1 | 36.5 | 25.1 | 0.06 | 31.3 | 26.4 | 0.54 | 31.1 | 23.7 | 0.41 | 29.5 | 22.8 | 0.35 |
| Lacto-N-difucohexaose I | 14.2 | 17.2 | 0.13 | 14.1 | 18.1 | 0.23 | 16.9 | 15.3 | 0.64 | 16.1 | 16.8 | 0.93 |
| Lacto-N-fucopentaose I | 12.6 | 10.4 | 0.30 | 12.2 | 10.2 | 1.00 | 11.6 | 10.0 | 0.81 | 10.9 | 11.6 | 0.63 |
| Lacto-N-fucopentaose II | 23.6 | 18.7 | 0.11 | 19.7 | 19.1 | 0.75 | 19.9 | 18.5 | 0.54 | 20.0 | 16.5 | 0.39 |
| **Lacto-N-fucopentaose III** | 11.7 | 12.7 | 0.09 | **11.4** | **13.4** | **0.01** | 12.7 | 12.1 | 0.48 | 12.7 | 10.9 | 0.07 |
| Lacto-N-fucopentaose V | 30.0 | 21.6 | 0.07 | 24.0 | 22.3 | 0.66 | 23.3 | 23.0 | 0.77 | 23.9 | 19.3 | 0.34 |
| **Lacto-N-hexaose** | **42.8** | **30.0** | **0.04** | 38.0 | 30.5 | 0.18 | 36.1 | 29.1 | 0.41 | 34.1 | 31.7 | 0.57 |
| **Lacto-N-neo-difucohexaose I** | **8.1** | **11.6** | **0.05** | 8.7 | 12.5 | 0.13 | 11.2 | 10.0 | 0.64 | 10.9 | 9.6 | 0.96 |
| Lacto-N-neo-difucohexaose II | 20.9 | 20.9 | 0.83 | 18.5 | 21.8 | 0.33 | 20.3 | 20.3 | 0.83 | 21.2 | 16.3 | 0.22 |
| Lacto-N-neo-hexaose | 20.1 | 21.9 | 0.70 | 19.7 | 23.7 | 0.64 | 23.4 | 18.2 | 0.41 | 22.6 | 16.9 | 0.63 |
| Lacto-N-neo-octaose | 27.2 | 21.9 | 0.53 | 24.1 | 22.9 | 0.92 | 25.4 | 19.6 | 0.66 | 24.2 | 18.8 | 0.81 |
| **Lacto-N-tetraose** | **22.5** | **16.1** | **0.01** | 19.2 | 16.1 | 0.14 | 18.0 | 16.7 | 0.30 | 17.8 | 16.0 | 0.28 |
| **Lacto-N-triose II** | **12.4** | **8.7** | **0.01** | 10.3 | 8.9 | 0.17 | 10.2 | 8.3 | 0.07 | 9.9 | 7.6 | 0.25 |
| Lactose-3'-Sulfate | 7.7 | 7.3 | 0.72 | 6.9 | 7.7 | 0.29 | 7.7 | 6.8 | 0.51 | 7.4 | 6.6 | 0.52 |
| **Sialyllacto-N-hexaose-X1** | **106.7** | **76.9** | **0.03** | 90.0 | 80.3 | 0.37 | 84.9 | 84.4 | 0.78 | 86.9 | 71.9 | 0.54 |
| Sialyllacto-N-hexaose-X2 | 38.1 | 43.0 | 0.27 | 37.5 | 45.7 | 0.38 | 44.1 | 37.6 | 0.57 | 41.8 | 41.0 | 0.94 |
| Sialyllacto-N-tetraose a | 23.7 | 17.0 | 0.23 | 20.6 | 17.2 | 0.66 | 18.8 | 18.8 | 0.81 | 18.8 | 17.8 | 0.43 |
| **Sialyllacto-N-tetraose b** | **33.9** | **25.6** | **0.02** | 29.0 | 25.9 | 0.28 | **28.4** | **25.3** | **0.04** | 27.7 | 25.1 | **0.11** |
| Sialyllacto-N-tetraose c | 39.3 | 43.8 | 0.49 | 39.0 | 45.2 | 0.38 | 44.5 | 39.2 | 0.18 | 42.8 | 40.2 | 0.47 |
| Trifucosyl(1-2,1-2,1-3)-iso-lacto-N-octaose | 13.7 | 12.9 | 0.28 | 15.5 | 12.1 | 0.95 | 14.6 | 11.7 | 0.87 | 13.5 | 13.6 | 0.66 |
| Trifucosyllacto-N-hexaose I | 19.4 | 24.3 | 0.06 | 19.6 | 26.0 | 0.13 | 23.6 | 22.6 | 0.89 | 22.8 | 23.3 | 0.86 |
| Trifucosyllacto-N-hexaose-X1 | 13.7 | 16.6 | 0.11 | 12.9 | 17.9 | 0.14 | 16.6 | 14.0 | 0.58 | 16.0 | 13.2 | 0.75 |
| Trifucosyllacto-N-octaose-X1 | 27.0 | 24.4 | 0.39 | 25.3 | 24.9 | 0.56 | 27.0 | 22.0 | 0.49 | 25.3 | 23.6 | 0.86 |
| Trifucosyllacto-N-tetraose-X5 | 19.8 | 20.9 | 0.27 | 18.9 | 21.6 | 0.36 | 21.1 | 19.4 | 0.64 | 20.4 | 20.1 | 0.73 |
| a-Heptasaccharide | 10.7 | 12.3 | 0.42 | 13.8 | 10.2 | 0.99 | 13.1 | 12.8 | 0.31 | 13.7 | 7.6 | 0.21 |
| a-Pentasaccharide | 8.1 | 9.8 | 0.64 | 11.1 | 8.5 | 0.76 | 10.4 | 10.3 | 0.18 | 10.8 | 6.6 | 0.14 |
| a-Tetrasaccharide | 7.6 | 6.7 | 0.56 | 9.4 | 5.6 | 0.74 | 8.3 | 6.7 | 0.17 | 8.2 | 4.0 | 0.15 |

p values derived from Wilcoxon sum-rank test comparing HMO levels between infants with and without LRTI or URTI at 1 and/or at 2 years. **Bold** denotes statistically significant values at nominal level (p <0.05). None of the p values shown were significant following correction for multiple testing (false discovery rate, FDR <0.010). LRTI: Lower respiratory tract infections; URTI: Upper respiratory tract infections; HMO: human milk oligosaccharides.

**Table S3:** Weighted mean relative levels of human milk oligosaccharides measured at 6 months stratified by otitis media

|  | **OM at 1 year** | | | **OM at 2 years** | | |
| --- | --- | --- | --- | --- | --- | --- |
| **HMO Structure** | Yes (n=16) | No (n=97) | p | Yes (n=35) | No (n=98) | p |
| 2,3 Difucosyllactose | 16.0 | 13.9 | 0.20 | 15.1 | 13.5 | 0.08 |
| 2-Fucosyllactose | 20.0 | 18.9 | 0.21 | 20.5 | 18.1 | 0.06 |
| 3'-Sialyl-3-fucosyllactose | 40.1 | 36.9 | 0.90 | 35.3 | 38.5 | 0.11 |
| **3-Fucosyllactose** | 44.6 | 49.7 | 0.28 | **43.5** | **51.8** | **0.01** |
| 3-Galactosyllactose | 2.2 | 2.0 | 0.19 | 2.1 | 2.0 | 0.08 |
| 3-sialyllactose | 17.0 | 14.5 | 0.18 | 15.3 | 14.7 | 0.67 |
| 6'-Sialyl-N-acetyllactosamine | 7.1 | 7.9 | 0.63 | 7.2 | 8.0 | 0.62 |
| 6-Galactosyllactose | 13.2 | 13.7 | 0.45 | 13.3 | 13.7 | 0.85 |
| 6-sialyllactose | 6.6 | 8.3 | 0.47 | 7.4 | 8.3 | 0.77 |
| Difucosyl-lacto-N-hexaose I | 4.3 | 3.8 | 0.13 | 4.2 | 3.6 | 0.09 |
| **Difucosyl-lacto-N-hexaose II** | 13.6 | 13.7 | 0.75 | **11.4** | **14.9** | **0.01** |
| **Difucosyl-lacto-N-hexaose-X1** | 6.4 | 4.5 | 0.06 | **5.8** | **4.4** | **0.02** |
| **Difucosyl-lacto-N-hexaose-X2** | **6.4** | **4.4** | **0.05** | 4.6 | 4.7 | 0.15 |
| Difucosyl-lacto-N-hexaose-X3 | 14.2 | 13.6 | 0.47 | 13.6 | 13.9 | 1.00 |
| Difucosyl-lacto-N-neo-hexaose I | 15.7 | 15.9 | 0.70 | 15.3 | 15.9 | 0.39 |
| Difucosyl-para-lacto-N-hexaose I | 16.7 | 14.3 | 0.84 | 13.7 | 15.6 | 0.13 |
| Difucosyldisialyllacto-N-hexaose-X1 | 10.9 | 10.6 | 0.31 | 10.9 | 10.4 | 0.70 |
| **Difucosyldisialyllacto-N-hexaose-X2** | 23.9 | 25.8 | 0.48 | **21.1** | **27.6** | **0.01** |
| Difucosylsialyllacto-N-hexaose-X1 | 10.3 | 9.4 | 0.23 | 10.0 | 9.2 | 0.30 |
| Difucosylsialyllacto-N-hexaose-X2 | 6.5 | 7.2 | 0.61 | 6.7 | 7.2 | 0.81 |
| Disialyllacto-N-Hexaose-X1 | 13.4 | 11.7 | 0.57 | 11.4 | 12.2 | 0.69 |
| Disialyllacto-N-Hexaose-X2 | 23.2 | 16.6 | 0.15 | 19.6 | 17.2 | 0.61 |
| Disialyllacto-N-Hexaose-X5 | 3.8 | 4.8 | 0.73 | 4.3 | 4.7 | 0.86 |
| Disialyllacto-N-tetraose | 19.9 | 15.4 | 0.75 | 17.8 | 16.0 | 0.70 |
| Fucosyl(1-3)-iso-lacto-N-octaose | 6.2 | 4.6 | 0.53 | 5.1 | 4.7 | 0.57 |
| Fucosyl-para-lacto-N-hexaose I | 12.5 | 8.6 | 0.28 | 10.8 | 8.8 | 0.11 |
| Fucosyldiasialyllacto-N-hexaose-X1 | 16.8 | 15.0 | 0.92 | 15.4 | 15.1 | 0.47 |
| Fucosyldiasialyllacto-N-hexaose-X2 | 20.9 | 16.1 | 0.44 | 19.0 | 16.3 | 0.65 |
| Fucosyldisialyllacto-N-tetraose-X2 | 33.6 | 29.4 | 0.83 | 28.1 | 31.6 | 0.14 |
| Fucosyllacto-N-hexaose II | 9.0 | 7.7 | 0.96 | 8.0 | 7.8 | 0.92 |
| Fucosyllacto-N-hexaose-X1 | 8.8 | 6.9 | 0.08 | 7.4 | 7.1 | 0.09 |
| Fucosyllacto-N-hexaose-X2 | 4.7 | 3.8 | 0.38 | 3.9 | 3.9 | 0.53 |
| Fucosyllacto-N-hexaose-X4 | 13.6 | 11.2 | 0.20 | 11.3 | 11.6 | 0.49 |
| Fucosyllacto-N-octaose-X1 | 6.4 | 5.4 | 0.68 | 5.7 | 5.5 | 0.78 |
| Fucosyllacto-N-octaose-X2 | 8.4 | 7.3 | 0.62 | 7.1 | 7.6 | 0.99 |
| **Fucosyllacto-N-sulfate-X1** | 35.2 | 24.6 | 0.09 | **30.0** | **23.8** | **0.03** |
| Fucosylsialyllacto-N-Hexaose-X1 | 18.2 | 16.6 | 0.90 | 16.0 | 17.3 | 0.37 |
| Fucosylsialyllacto-N-Hexaose-X2 | 13.8 | 12.1 | 0.23 | 13.0 | 11.9 | 0.30 |
| **Fucosylsialyllacto-N-Hexaose-X3** | 2.9 | 3.0 | 0.50 | **3.2** | **2.8** | **0.04** |
| Fucosylsialyllacto-N-Hexaose-X4 | 10.4 | 11.6 | 0.98 | 11.1 | 11.4 | 0.96 |
| **Fucosylsialyllacto-N-Hexaose-X5** | 11.0 | 12.5 | 0.25 | **10.8** | **13.1** | **0.02** |
| Fucosylsialyllacto-N-Hexaose-X6 | 12.2 | 14.1 | 0.84 | 14.0 | 13.6 | 0.43 |
| Fucosylsialyllacto-N-neo-tetraose c | 15.8 | 17.4 | 0.69 | 15.3 | 18.1 | 0.11 |
| Fucosylsialyllacto-N-tetraose a | 22.0 | 22.7 | 0.74 | 19.4 | 24.6 | 0.06 |
| **Fucosylsialyllacto-N-tetraose b** | 22.8 | 13.2 | 0.06 | **17.6** | **13.3** | **0.04** |
| Fucosylsialyllacto-N-tetraose-X1 | 26.9 | 20.6 | 0.32 | 22.1 | 21.5 | 0.66 |
| Lacto-N-decaose-X1 | 6.0 | 5.5 | 0.09 | 5.6 | 5.5 | 0.33 |
| **Lacto-N-difucohexaose I** | **13.2** | **8.0** | **0.03** | 10.5 | 8.1 | 0.06 |
| **Lacto-N-fucopentaose I** | 6.2 | 4.3 | 0.06 | **5.4** | **4.4** | **0.03** |
| **Lacto-N-fucopentaose II** | 15.4 | 14.6 | 0.73 | **13.1** | **15.9** | **0.04** |
| Lacto-N-fucopentaose III | 13.7 | 12.8 | 0.44 | 13.0 | 13.0 | 0.94 |
| Lacto-N-fucopentaose V | 17.8 | 16.7 | 0.75 | 15.3 | 18.2 | 0.15 |
| Lacto-N-hexaose | 5.8 | 5.5 | 0.38 | 4.9 | 5.8 | 0.24 |
| Lacto-N-neo-difucohexaose I | 10.2 | 7.5 | 0.09 | 8.8 | 7.4 | 0.06 |
| **Lacto-N-neo-difucohexaose II** | 28.2 | 31.5 | 0.69 | **25.8** | **33.8** | **0.03** |
| Lacto-N-neo-hexaose | 10.9 | 8.9 | 0.13 | 8.5 | 9.4 | 0.08 |
| Lacto-N-neo-octaose | 8.3 | 6.7 | 0.10 | 7.1 | 6.9 | 0.27 |
| Lacto-N-tetraose | 12.4 | 9.9 | 0.52 | 10.9 | 10.2 | 0.67 |
| Lacto-N-triose II | 3.4 | 3.3 | 0.49 | 3.4 | 3.4 | 0.15 |
| Lactose-3'-Sulfate | 3.1 | 2.5 | 1.00 | 2.7 | 2.5 | 0.81 |
| Sialyllacto-N-hexaose-X1 | 6.7 | 7.1 | 0.80 | 6.6 | 7.2 | 0.95 |
| Sialyllacto-N-hexaose-X2 | 7.5 | 7.9 | 0.63 | 7.3 | 7.9 | 0.42 |
| Sialyllacto-N-tetraose a | 8.2 | 7.3 | 0.68 | 8.4 | 7.2 | 0.17 |
| Sialyllacto-N-tetraose b | 23.9 | 17.8 | 0.32 | 19.9 | 18.7 | 0.91 |
| Sialyllacto-N-tetraose c | 8.0 | 8.7 | 0.75 | 8.3 | 8.7 | 0.57 |
| Trifucosyl(1-2,1-2,1-3)-iso-lacto-N-octaose | 3.1 | 2.4 | 0.09 | 2.7 | 2.4 | 0.25 |
| **Trifucosyllacto-N-hexaose I** | **9.9** | **8.0** | **0.05** | 8.8 | 7.8 | 0.11 |
| **Trifucosyllacto-N-hexaose-X1** | **13.6** | **7.6** | **0.02** | **10.6** | **7.7** | **0.04** |
| Trifucosyllacto-N-octaose-X1 | 8.3 | 4.7 | 0.08 | 6.1 | 4.8 | 0.08 |
| Trifucosyllacto-N-tetraose-X5 | 20.8 | 16.6 | 0.05 | 19.2 | 16.3 | 0.07 |
| a-Heptasaccharide | 10.2 | 9.7 | 0.40 | 12.1 | 8.2 | 0.21 |
| a-Pentasaccharide | 13.4 | 10.2 | 0.26 | 14.2 | 8.5 | 0.23 |
| a-Tetrasaccharide | 6.9 | 6.4 | 0.50 | 7.2 | 5.9 | 0.39 |

p values derived from Wilcoxon sum-rank test comparing HMO levels between infants with and without otitis media at 1 and/or at 2 years. **Bold** denotes statistically significant values at nominal level (p <0.05). None of the difference shown were significant following correction for multiple testing (False discovery rate, FDR <0.010). OM: Otitis media; HMO: human milk oligosaccharides

**Table S4**: Weighted mean relative levels of human milk oligosaccharides measured at 6 months stratified by lower and upper respiratory tract infections

|  | **LRTI at 1 year** | | | **LRTI at 2 years** | | | **URTI at 1 year** | | | | **URTI at 2 years** | | | |
| --- | --- | --- | --- | --- | --- | --- | --- | --- | --- | --- | --- | --- | --- | --- |
| **HMO Structure** | Yes (n=37) | No (n=78) | p | Yes (n=57) | No (n=64) | p | Yes (n=80) | No (n=38) | p | Yes (n=104) | | No (n=19) | p |  |
| 2,3 Difucosyllactose | 11.8 | 15.2 | 0.15 | 12.4 | 15.9 | 0.22 | 14.2 | 13.9 | 0.78 | 14.1 | | 15.4 | 0.49 |  |
| 2-Fucosyllactose | 16.8 | 19.4 | 0.15 | 19.3 | 18.9 | 0.48 | 18.7 | 19.4 | 1.00 | 18.2 | | 22.3 | 0.30 |  |
| 3'-Sialyl-3-fucosyllactose | 41.0 | 36.4 | 0.21 | 36.3 | 37.1 | 0.60 | 38.3 | 33.4 | 0.29 | 37.6 | | 32.6 | 0.18 |  |
| 3-Fucosyllactose | 48.9 | 49.6 | 0.96 | 45.1 | 51.1 | 0.50 | 48.6 | 48.3 | 0.97 | 49.2 | | 45.6 | 0.56 |  |
| 3-Galactosyllactose | 2.4 | 1.9 | 0.28 | 2.1 | 1.9 | 0.83 | 2.1 | 1.7 | 0.12 | 2.0 | | 1.7 | 0.44 |  |
| 3-sialyllactose | 16.2 | 14.3 | 0.11 | 15.7 | 14.1 | 0.07 | 15.1 | 14.0 | 0.16 | 14.8 | | 14.6 | 0.31 |  |
| 6'-Sialyl-N-acetyllactosamine | 8.5 | 7.3 | 0.14 | 7.8 | 7.1 | 0.20 | 7.7 | 7.0 | 0.06 | 7.3 | | 8.2 | 0.41 |  |
| 6-Galactosyllactose | 12.6 | 14.0 | 0.32 | 12.4 | 14.0 | 0.34 | 13.0 | 14.1 | 0.93 | 13.1 | | 14.1 | 0.98 |  |
| 6-sialyllactose | 8.2 | 7.8 | 0.88 | 8.1 | 7.5 | 0.72 | 7.9 | 7.6 | 0.12 | 7.7 | | 8.3 | 0.13 |  |
| Difucosyl-lacto-N-hexaose I | 3.6 | 3.5 | 0.71 | 4.5 | 3.0 | 0.48 | 3.8 | 3.6 | 0.93 | 3.4 | | 4.8 | 0.37 |  |
| Difucosyl-lacto-N-hexaose II | 15.9 | 13.1 | 0.16 | 14.3 | 12.9 | 0.38 | 14.0 | 12.8 | 0.96 | 14.0 | | 11.5 | 0.43 |  |
| Difucosyl-lacto-N-hexaose-X1 | 5.1 | 4.5 | 0.74 | 5.4 | 4.2 | 0.63 | 4.7 | 4.9 | 0.61 | 4.3 | | 6.7 | 0.81 |  |
| Difucosyl-lacto-N-hexaose-X2 | 4.7 | 4.8 | 0.54 | 4.4 | 4.7 | 0.47 | 5.0 | 3.7 | 0.41 | 4.4 | | 4.9 | 0.84 |  |
| **Difucosyl-lacto-N-hexaose-X3** | 13.1 | 13.7 | 0.78 | 12.6 | 13.9 | 1.00 | **14.6** | **11.2** | **0.03** | 13.9 | | 10.7 | 0.06 |  |
| Difucosyl-lacto-N-neo-hexaose I | 14.4 | 16.0 | 0.80 | 14.9 | 16.0 | 0.98 | 16.0 | 15.0 | 0.52 | 15.9 | | 14.0 | 0.23 |  |
| Difucosyl-para-lacto-N-hexaose I | 17.7 | 14.0 | 0.25 | 15.1 | 14.0 | 0.53 | 15.9 | 11.6 | 0.15 | 15.5 | | 10.3 | 0.08 |  |
| Difucosyldisialyllacto-N-hexaose-X1 | 10.6 | 10.6 | 0.90 | 10.8 | 10.3 | 0.95 | 10.5 | 10.6 | 0.45 | 10.2 | | 12.0 | 0.28 |  |
| Difucosyldisialyllacto-N-hexaose-X2 | 27.9 | 24.6 | 0.13 | 26.0 | 23.9 | 0.24 | 25.2 | 24.9 | 0.96 | 24.7 | | 25.6 | 0.77 |  |
| Difucosylsialyllacto-N-hexaose-X1 | 9.1 | 9.6 | 0.23 | 9.3 | 9.3 | 0.41 | 9.6 | 8.7 | 0.29 | 9.0 | | 10.3 | 0.43 |  |
| Difucosylsialyllacto-N-hexaose-X2 | 6.1 | 7.6 | 0.26 | 6.4 | 7.5 | 0.38 | 6.6 | 8.2 | 0.76 | 6.4 | | 9.8 | 0.27 |  |
| Disialyllacto-N-Hexaose-X1 | 13.1 | 11.2 | 0.84 | 12.6 | 10.7 | 0.53 | 12.6 | 9.8 | 0.29 | 11.3 | | 12.3 | 0.96 |  |
| **Disialyllacto-N-Hexaose-X2** | 21.9 | 16.3 | 0.44 | 19.5 | 15.6 | 0.58 | **19.1** | **14.5** | **0.02** | 17.3 | | 17.2 | 0.53 |  |
| **Disialyllacto-N-Hexaose-X5** | 4.4 | 4.6 | 0.21 | 4.4 | 4.5 | 0.11 | **4.6** | **4.2** | **0.02** | 4.3 | | 4.9 | 0.11 |  |
| Disialyllacto-N-tetraose | 21.2 | 14.7 | 0.07 | 19.0 | 14.0 | 0.16 | 16.8 | 15.3 | 0.34 | 16.2 | | 16.5 | 0.39 |  |
| **Fucosyl(1-3)-iso-lacto-N-octaose** | 6.7 | 4.0 | 0.07 | **5.9** | **3.8** | **0.04** | 5.2 | 4.0 | 0.71 | 4.9 | | 3.7 | 0.31 |  |
| **Fucosyl-para-lacto-N-hexaose I** | **12.4** | **8.2** | **0.05** | 10.5 | 8.1 | 0.08 | 10.0 | 7.8 | 0.10 | 9.4 | | 8.0 | 0.14 |  |
| Fucosyldiasialyllacto-N-hexaose-X1 | 16.0 | 14.3 | 0.19 | 16.6 | 13.3 | 0.14 | 15.1 | 14.7 | 0.90 | 14.6 | | 16.0 | 0.93 |  |
| Fucosyldiasialyllacto-N-hexaose-X2 | 20.1 | 15.8 | 0.17 | 18.5 | 15.3 | 0.24 | 17.8 | 14.9 | 0.08 | 16.9 | | 15.8 | 0.13 |  |
| Fucosyldisialyllacto-N-tetraose-X2 | 36.3 | 28.4 | 0.12 | 30.9 | 28.5 | 0.51 | 31.6 | 25.2 | 0.31 | 30.5 | | 25.6 | 0.28 |  |
| Fucosyllacto-N-hexaose II | 9.2 | 7.1 | 0.08 | 8.8 | 6.8 | 0.08 | 8.0 | 7.5 | 0.94 | 7.9 | | 7.0 | 0.46 |  |
| Fucosyllacto-N-hexaose-X1 | 7.9 | 6.9 | 0.79 | 7.0 | 6.9 | 0.87 | 7.2 | 6.7 | 0.49 | 6.6 | | 8.4 | 0.49 |  |
| Fucosyllacto-N-hexaose-X2 | 4.4 | 3.7 | 0.88 | 4.1 | 3.5 | 0.57 | 4.0 | 3.4 | 0.38 | 3.6 | | 4.4 | 0.37 |  |
| **Fucosyllacto-N-hexaose-X4** | 11.6 | 11.3 | 0.73 | 10.6 | 11.5 | 0.68 | **12.7** | **8.0** | **0.004** | 11.5 | | 8.4 | 0.29 |  |
| Fucosyllacto-N-octaose-X1 | 5.5 | 5.6 | 0.59 | 5.5 | 5.5 | 0.87 | 5.6 | 5.3 | 0.60 | 5.3 | | 6.0 | 0.32 |  |
| Fucosyllacto-N-octaose-X2 | 8.3 | 7.1 | 0.52 | 7.4 | 7.1 | 0.50 | 7.9 | 6.1 | 0.32 | 7.5 | | 6.1 | 0.72 |  |
| Fucosyllacto-N-sulfate-X1 | 30.0 | 23.8 | 0.58 | 26.8 | 24.6 | 0.73 | 28.3 | 19.0 | 0.46 | 26.5 | | 19.8 | 0.77 |  |
| **Fucosylsialyllacto-N-Hexaose-X1** | **19.7** | **15.6** | **0.03** | 18.0 | 15.4 | 0.10 | 17.0 | 16.0 | 0.34 | 16.6 | | 16.3 | 0.31 |  |
| Fucosylsialyllacto-N-Hexaose-X2 | 12.1 | 12.1 | 0.97 | 13.4 | 11.2 | 0.59 | 12.2 | 12.3 | 0.14 | 11.9 | | 13.4 | 0.16 |  |
| Fucosylsialyllacto-N-Hexaose-X3 | 2.8 | 2.9 | 0.39 | 3.3 | 2.5 | 0.78 | 2.9 | 2.9 | 0.80 | 2.6 | | 3.6 | 0.55 |  |
| **Fucosylsialyllacto-N-Hexaose-X4** | 9.8 | 11.8 | 0.70 | 10.3 | 11.6 | 0.61 | **11.6** | **10.4** | **0.02** | 11.0 | | 11.1 | 0.07 |  |
| **Fucosylsialyllacto-N-Hexaose-X5** | **13.7** | **11.7** | **0.02** | 12.8 | 11.5 | 0.09 | 12.4 | 11.5 | 0.47 | 12.4 | | 10.9 | 0.18 |  |
| Fucosylsialyllacto-N-Hexaose-X6 | 12.2 | 14.1 | 0.76 | 12.5 | 13.8 | 0.77 | **14.2** | **12.0** | **0.02** | 13.7 | | 11.6 | 0.10 |  |
| **Fucosylsialyllacto-N-neo-tetraose c** | 18.5 | 16.3 | 0.10 | 15.8 | 16.8 | 0.71 | **18.3** | **12.8** | **0.04** | **17.4** | | **12.1** | **0.05** |  |
| Fucosylsialyllacto-N-tetraose a | 24.8 | 22.0 | 0.29 | 23.0 | 21.3 | 0.61 | 22.6 | 21.6 | 0.92 | 22.9 | | 19.8 | 0.51 |  |
| Fucosylsialyllacto-N-tetraose b | 17.2 | 13.9 | 0.66 | 16.2 | 13.6 | 0.96 | 15.0 | 13.8 | 0.55 | 14.2 | | 16.8 | 0.43 |  |
| **Fucosylsialyllacto-N-tetraose-X1** | 26.0 | 20.1 | 0.08 | 22.8 | 19.9 | 0.38 | **23.3** | **16.5** | **0.02** | **22.2** | | **15.9** | **0.04** |  |
| Lacto-N-decaose-X1 | 6.3 | 5.3 | 0.24 | 6.0 | 5.0 | 0.36 | 5.7 | 5.0 | 0.89 | 5.5 | | 5.3 | 0.41 |  |
| Lacto-N-difucohexaose I | 9.8 | 8.8 | 0.52 | 9.5 | 8.8 | 0.56 | 9.3 | 8.3 | 0.57 | 9.1 | | 9.0 | 0.83 |  |
| Lacto-N-fucopentaose I | 5.6 | 4.2 | 0.67 | 5.6 | 3.9 | 0.81 | 4.6 | 4.6 | 0.74 | 4.3 | | 5.9 | 0.41 |  |
| Lacto-N-fucopentaose II | 17.6 | 14.1 | 0.24 | 15.1 | 14.3 | 0.64 | 15.5 | 12.8 | 0.31 | 15.4 | | 11.5 | 0.12 |  |
| Lacto-N-fucopentaose III | 12.7 | 12.8 | 0.59 | 12.4 | 12.9 | 0.77 | 13.3 | 11.7 | 0.08 | 13.0 | | 11.5 | 0.14 |  |
| Lacto-N-fucopentaose V | 21.8 | 15.7 | 0.07 | 18.4 | 15.6 | 0.35 | 17.7 | 15.1 | 0.43 | 17.7 | | 13.2 | 0.11 |  |
| Lacto-N-hexaose | 8.2 | 4.5 | 0.27 | 6.8 | 4.1 | 0.34 | 6.1 | 4.0 | 0.23 | 5.3 | | 5.2 | 0.84 |  |
| Lacto-N-neo-difucohexaose I | 7.0 | 8.1 | 0.30 | 7.4 | 8.0 | 0.59 | 8.3 | 6.4 | 0.38 | 7.7 | | 7.3 | 0.68 |  |
| Lacto-N-neo-difucohexaose II | 31.2 | 31.0 | 0.57 | 27.7 | 32.1 | 0.88 | 32.1 | 26.6 | 0.33 | 31.6 | | 24.4 | 0.22 |  |
| Lacto-N-neo-hexaose | 9.5 | 9.1 | 0.94 | 8.4 | 9.1 | 0.89 | 10.0 | 6.5 | 0.19 | 9.0 | | 7.5 | 0.87 |  |
| Lacto-N-neo-octaose | 7.6 | 6.7 | 0.54 | 7.3 | 6.4 | 0.75 | 7.4 | 5.7 | 0.16 | 6.9 | | 6.2 | 0.33 |  |
| **Lacto-N-tetraose** | **13.6** | **9.3** | **0.03** | 11.8 | 9.1 | 0.14 | 10.8 | 9.4 | 0.26 | 10.5 | | 9.4 | 0.11 |  |
| **Lacto-N-triose II** | **4.6** | **2.8** | **<0.001** | 3.8 | 2.9 | 0.05 | **3.6** | **2.7** | **0.03** | 3.3 | | 3.1 | **0.45** |  |
| Lactose-3'-Sulfate | 2.8 | 2.5 | 0.83 | 2.7 | 2.4 | 0.84 | 2.6 | 2.4 | 0.32 | 2.5 | | 2.5 | 0.49 |  |
| Sialyllacto-N-hexaose-X1 | 7.8 | 6.7 | 0.16 | 7.1 | 6.7 | 0.36 | 7.2 | 6.4 | 0.39 | 6.9 | | 6.6 | 0.19 |  |
| Sialyllacto-N-hexaose-X2 | 7.1 | 8.1 | 0.28 | 7.1 | 7.9 | 0.49 | 7.8 | 7.1 | 0.46 | 7.4 | | 8.0 | 0.82 |  |
| Sialyllacto-N-tetraose a | 9.1 | 6.9 | 0.20 | 9.1 | 6.2 | 0.14 | 7.5 | 7.4 | 0.68 | 7.5 | | 7.5 | 0.77 |  |
| **Sialyllacto-N-tetraose b** | **24.0** | **17.3** | **0.04** | 20.6 | 17.2 | 0.26 | 19.7 | 16.6 | 0.08 | 19.0 | | 17.0 | 0.08 |  |
| **Sialyllacto-N-tetraose c** | 8.2 | 8.4 | 0.51 | 8.1 | 8.2 | 0.56 | **9.0** | **6.7** | **0.01** | 8.4 | | 7.2 | 0.09 |  |
| Trifucosyl(1-2,1-2,1-3)-iso-lacto-N-octaose | 2.9 | 2.2 | 0.85 | 3.0 | 1.9 | 0.20 | 2.6 | 2.2 | 0.54 | 2.3 | | 2.9 | 0.19 |  |
| Trifucosyllacto-N-hexaose I | 7.8 | 8.3 | 0.39 | 8.2 | 8.1 | 0.39 | 8.2 | 8.2 | 0.57 | 7.8 | | 9.8 | 0.86 |  |
| Trifucosyllacto-N-hexaose-X1 | 9.4 | 8.5 | 0.50 | 8.8 | 8.6 | 0.45 | 9.3 | 7.2 | 0.43 | 8.9 | | 7.5 | 0.94 |  |
| Trifucosyllacto-N-octaose-X1 | 6.6 | 4.6 | 0.30 | 6.3 | 4.1 | 0.38 | 5.7 | 4.2 | 0.55 | 5.2 | | 5.1 | 0.93 |  |
| Trifucosyllacto-N-tetraose-X5 | 17.1 | 17.7 | 0.64 | 17.4 | 17.4 | 0.75 | 17.9 | 15.9 | 0.77 | 17.5 | | 17.2 | 0.55 |  |
| a-Heptasaccharide | 7.1 | 9.0 | 0.76 | 9.0 | 8.5 | 0.69 | 9.9 | 8.0 | 0.16 | 9.7 | | 6.0 | 0.25 |  |
| a-Pentasaccharide | 8.0 | 9.7 | 1.00 | 9.6 | 9.5 | 0.89 | 11.0 | 8.3 | 0.12 | 10.5 | | 6.4 | 0.32 |  |
| a-Tetrasaccharide | 7.7 | 4.8 | 0.98 | 7.2 | 4.8 | 0.98 | 7.0 | 4.1 | 0.16 | 6.5 | | 3.1 | 0.36 |  |

p values derived from Wilcoxon sum-rank test comparing HMO levels between infants with and without LRTI or URTI at 1 and/or at 2 years. Bold denotes statistically significant values at nominal level (p <0.05). None of the p values shown were significant following correction for multiple testing (false discovery rate, FDR <0.010). LRTI: Lower respiratory tract infections; URTI: Upper respiratory tract infections; HMO: human milk oligosaccharides.

**Table S5**: Mean relative levels of human milk oligosaccharides measured at 6 weeks stratified by secretor milk and otitis media

|  | **OM at 1 year** | | | | | | **OM at 2 years** | | | | | |
| --- | --- | --- | --- | --- | --- | --- | --- | --- | --- | --- | --- | --- |
|  | **Secretor milk** | | | **Non-secretor milk** | | | **Secretor milk** | | | **Non-secretor milk** | | |
| **HMO Structure** | OM Yes (n=10) | OM No (n=43) | p | OM Yes (n=6) | OM No (n=54) | p | OM Yes (n=21) | OM No (n=41) | p | OM Yes (n=14) | OM No (n=57) | p |
| 2,3 Difucosyllactose | 12.8 | 14.4 | 0.27 | 1.1 | 1.1 | 1.00 | 13.1 | 14.6 | 0.10 | 1.1 | 1.1 | 1.00 |
| **2-Fucosyllactose** | **24.2** | **30.0** | **0.03** | 2.1 | 2.1 | 1.00 | 27.4 | 29.7 | 0.12 | 2.1 | 2.1 | 1.00 |
| 3'-Sialyl-3-fucosyllactose | 20.8 | 21.8 | 0.87 | 38.8 | 51.3 | 0.15 | 20.8 | 21.9 | 0.72 | 41.9 | 52.5 | 0.08 |
| **3-Fucosyllactose** | 25.8 | 25.7 | 0.97 | 60.5 | 67.1 | 0.36 | 24.0 | 26.5 | 0.61 | **54.0** | **70.8** | **0.02** |
| **3-Galactosyllactose** | 2.3 | 2.7 | 0.51 | 8.4 | 2.9 | 0.62 | 2.6 | 2.7 | 0.80 | **5.8** | **2.7** | **0.04** |
| 3-sialyllactose | 15.5 | 14.9 | 0.93 | 11.9 | 13.7 | 0.23 | 15.2 | 15.0 | 0.86 | 13.0 | 13.6 | 0.37 |
| 6'-Sialyl-N-acetyllactosamine | 26.9 | 35.2 | 0.09 | 27.8 | 27.6 | 0.89 | 30.9 | 35.0 | 0.26 | 28.3 | 27.0 | 0.95 |
| **6-Galactosyllactose** | 15.2 | 18.8 | 0.10 | 47.8 | 20.3 | 0.30 | 17.2 | 18.7 | 0.47 | **35.1** | **19.6** | **0.02** |
| 6-sialyllactose | 38.7 | 43.9 | 0.29 | 41.2 | 39.4 | 0.66 | 40.4 | 44.3 | 0.26 | 39.1 | 39.6 | 0.94 |
| Difucosyl-lacto-N-hexaose I | 18.3 | 23.6 | 0.42 | 1.1 | 1.1 | 1.00 | 22.4 | 22.6 | 0.79 | 1.1 | 1.1 | 1.00 |
| **Difucosyl-lacto-N-hexaose II** | 20.5 | 20.8 | 0.65 | 74.2 | 72.1 | 0.72 | 20.6 | 20.5 | 0.87 | **55.4** | **77.2** | **0.03** |
| Difucosyl-lacto-N-hexaose-X1 | 14.9 | 11.1 | 0.57 | 0.5 | 0.5 | 1.00 | 13.6 | 11.4 | 0.72 | 0.5 | 0.5 | 1.00 |
| Difucosyl-lacto-N-hexaose-X2 | 21.7 | 26.9 | 0.34 | 0.5 | 0.5 | 1.00 | 20.9 | 28.5 | 0.12 | 0.5 | 0.5 | 1.00 |
| Difucosyl-lacto-N-hexaose-X3 | 11.9 | 12.5 | 0.82 | 10.7 | 14.7 | 0.15 | 11.3 | 13.3 | 0.35 | 13.5 | 14.5 | 0.73 |
| Difucosyl-lacto-N-neo-hexaose I | 16.3 | 17.2 | 0.84 | 20.8 | 22.5 | 0.97 | 16.6 | 17.2 | 0.74 | 19.8 | 23.0 | 0.27 |
| Difucosyl-para-lacto-N-hexaose I | 14.3 | 12.8 | 0.18 | 37.2 | 39.6 | 0.76 | 13.0 | 13.4 | 0.82 | 33.7 | 41.0 | 0.45 |
| Difucosyldisialyllacto-N-hexaose-X1 | 44.6 | 56.3 | 0.19 | 9.2 | 9.5 | 0.57 | 50.1 | 56.2 | 0.36 | 9.2 | 9.5 | 0.34 |
| **Difucosyldisialyllacto-N-hexaose-X2** | 50.2 | 50.8 | 0.77 | 113.8 | 159.3 | 0.28 | 50.9 | 50.2 | 0.98 | **94.8** | **170.0** | **0.01** |
| Difucosylsialyllacto-N-hexaose-X1 | 36.2 | 46.8 | 0.28 | 4.5 | 4.5 | 1.00 | 39.2 | 47.7 | 0.18 | 4.5 | 4.5 | 1.00 |
| Difucosylsialyllacto-N-hexaose-X2 | 60.9 | 81.0 | 0.16 | 4.5 | 4.5 | 1.00 | 67.5 | 82.1 | 0.19 | 4.5 | 4.5 | 1.00 |
| Disialyllacto-N-Hexaose-X1 | 59.5 | 71.0 | 0.61 | 25.5 | 24.2 | 0.91 | 58.2 | 74.4 | 0.72 | 27.3 | 22.6 | 0.68 |
| Disialyllacto-N-Hexaose-X2 | 47.2 | 39.7 | 0.40 | 16.2 | 21.2 | 0.53 | 41.1 | 42.6 | 0.90 | 20.2 | 20.3 | 0.91 |
| Disialyllacto-N-Hexaose-X5 | 48.5 | 69.0 | 0.12 | 45.8 | 43.3 | 0.81 | 53.9 | 71.2 | 0.09 | 43.6 | 42.8 | 0.71 |
| Disialyllacto-N-tetraose | 33.8 | 29.4 | 0.31 | 27.7 | 37.6 | 0.23 | 32.8 | 30.6 | 0.63 | 34.3 | 36.5 | 0.62 |
| **Fucosyl(1-3)-iso-lacto-N-octaose** | 28.5 | 19.9 | 0.17 | 34.7 | 28.6 | 0.40 | 23.7 | 20.4 | 0.25 | **41.2** | **25.1** | **0.02** |
| **Fucosyl-para-lacto-N-hexaose I** | 20.3 | 13.8 | **0.04** | 12.7 | 14.1 | 0.76 | 17.7 | 14.4 | 0.33 | **18.9** | **12.2** | **0.02** |
| Fucosyldiasialyllacto-N-hexaose-X1 | 49.4 | 51.9 | 0.76 | 48.2 | 61.1 | 0.30 | 51.3 | 51.2 | 0.85 | 51.3 | 61.3 | 0.31 |
| Fucosyldiasialyllacto-N-hexaose-X2 | 34.3 | 31.3 | 0.50 | 23.0 | 33.2 | 0.13 | 32.7 | 32.5 | 0.98 | 33.2 | 31.4 | 0.96 |
| Fucosyldisialyllacto-N-tetraose-X2 | 27.0 | 27.2 | 0.58 | 64.8 | 92.5 | 0.23 | 26.5 | 27.7 | 0.99 | 65.9 | 95.8 | 0.11 |
| Fucosyllacto-N-hexaose II | 27.1 | 25.2 | 0.38 | 50.8 | 41.7 | 0.44 | 26.0 | 25.1 | 0.22 | 49.3 | 40.3 | 0.15 |
| Fucosyllacto-N-hexaose-X1 | 17.9 | 13.1 | 0.64 | 2.1 | 2.1 | 1.00 | 14.5 | 14.1 | 0.81 | 2.1 | 2.1 | 1.00 |
| Fucosyllacto-N-hexaose-X2 | 18.2 | 23.1 | 0.41 | 2.1 | 2.1 | 1.00 | 19.9 | 23.3 | 0.44 | 2.1 | 2.1 | 1.00 |
| Fucosyllacto-N-hexaose-X4 | 18.4 | 18.2 | 0.62 | 17.0 | 12.7 | 0.42 | 16.1 | 19.6 | 0.18 | 12.4 | 13.0 | 0.49 |
| Fucosyllacto-N-octaose-X1 | 17.9 | 19.8 | 0.81 | 4.5 | 4.5 | 1.00 | 17.5 | 20.5 | 0.57 | 4.5 | 4.5 | 1.00 |
| Fucosyllacto-N-octaose-X2 | 23.8 | 22.5 | 0.60 | 20.5 | 16.6 | 0.21 | 20.9 | 24.0 | 0.53 | 18.7 | 16.1 | 0.33 |
| Fucosyllacto-N-sulfate-X1 | 24.6 | 29.2 | 0.34 | 2.1 | 2.1 | 1.00 | 25.2 | 30.0 | 0.57 | 2.1 | 2.1 | 1.00 |
| Fucosylsialyllacto-N-Hexaose-X1 | 45.9 | 45.3 | 0.74 | 74.8 | 73.4 | 0.74 | 45.0 | 45.1 | 0.75 | 70.5 | 73.9 | 0.97 |
| Fucosylsialyllacto-N-Hexaose-X2 | 39.4 | 42.1 | 0.94 | 40.7 | 37.7 | 0.46 | 44.9 | 39.5 | 0.30 | 40.2 | 36.4 | 0.34 |
| Fucosylsialyllacto-N-Hexaose-X3 | 37.7 | 50.7 | 0.37 | 1.4 | 1.7 | 0.59 | 49.1 | 47.5 | 0.48 | 1.5 | 1.8 | 0.69 |
| Fucosylsialyllacto-N-Hexaose-X4 | 52.7 | 60.7 | 0.35 | 55.7 | 55.3 | 0.75 | 52.7 | 62.6 | 0.14 | 54.4 | 55.3 | 0.57 |
| Fucosylsialyllacto-N-Hexaose-X5 | 54.6 | 61.5 | 0.98 | 243.3 | 195.4 | 0.29 | 52.5 | 63.9 | 0.44 | 153.0 | 211.3 | 0.08 |
| Fucosylsialyllacto-N-Hexaose-X6 | 45.6 | 50.7 | 0.78 | 25.4 | 37.0 | 0.28 | 43.1 | 53.8 | 0.19 | 35.3 | 35.5 | 0.89 |
| Fucosylsialyllacto-N-neo-tetraose c | 43.1 | 49.9 | 0.70 | 75.5 | 89.0 | 0.65 | 40.8 | 53.0 | 0.14 | 70.2 | 92.3 | 0.18 |
| Fucosylsialyllacto-N-tetraose a | 24.8 | 22.7 | 0.45 | 63.0 | 69.6 | 0.80 | 23.9 | 23.0 | 0.62 | 52.4 | 72.6 | 0.23 |
| Fucosylsialyllacto-N-tetraose b | 30.1 | 27.8 | 0.45 | 2.1 | 2.1 | 1.00 | 28.6 | 28.5 | 0.68 | 2.1 | 2.1 | 1.00 |
| Fucosylsialyllacto-N-tetraose-X1 | 27.7 | 26.4 | 0.62 | 37.3 | 43.2 | 0.43 | 25.9 | 27.3 | 0.49 | 37.9 | 43.7 | 0.37 |
| Lacto-N-decaose-X1 | 35.2 | 29.3 | 0.58 | 36.7 | 21.9 | 0.22 | 29.5 | 30.3 | 0.97 | 37.8 | 18.4 | 0.12 |
| Lacto-N-difucohexaose I | 18.2 | 20.0 | 0.49 | 0.5 | 0.5 | 1.00 | 18.7 | 20.4 | 0.43 | 0.5 | 0.5 | 1.00 |
| Lacto-N-fucopentaose I | 16.6 | 12.6 | 0.71 | 0.5 | 0.5 | 1.00 | 14.9 | 12.8 | 0.68 | 0.5 | 0.5 | 1.00 |
| Lacto-N-fucopentaose II | 14.4 | 12.6 | 0.37 | 48.7 | 45.4 | 0.85 | 12.9 | 13.0 | 0.82 | 39.1 | 47.5 | 0.17 |
| Lacto-N-fucopentaose III | 12.1 | 12.1 | 0.91 | 12.0 | 13.3 | 0.35 | 11.8 | 12.5 | 0.53 | 13.0 | 13.1 | 0.87 |
| Lacto-N-fucopentaose V | 17.6 | 13.4 | 0.11 | 59.0 | 57.6 | 0.87 | 15.1 | 14.0 | 0.50 | 54.5 | 58.5 | 0.73 |
| Lacto-N-hexaose | 35.8 | 34.2 | 1.00 | 51.8 | 31.3 | 0.21 | 34.7 | 33.8 | 0.97 | 55.3 | 25.4 | 0.33 |
| Lacto-N-neo-difucohexaose I | 11.5 | 13.4 | 0.39 | 1.1 | 1.1 | 1.00 | 11.3 | 14.0 | 0.12 | 1.1 | 1.1 | 1.00 |
| Lacto-N-neo-difucohexaose II | 12.9 | 14.0 | 0.72 | 39.2 | 48.9 | 0.27 | 12.0 | 14.8 | 0.55 | 38.1 | 51.3 | 0.07 |
| Lacto-N-neo-hexaose | 22.0 | 26.2 | 0.74 | 10.5 | 6.9 | 0.25 | 19.7 | 28.5 | 0.22 | 10.3 | 6.1 | 0.75 |
| Lacto-N-neo-octaose | 28.8 | 25.7 | 0.78 | 15.0 | 12.1 | 0.21 | 25.2 | 26.9 | 0.82 | 17.8 | 10.4 | 0.18 |
| **Lacto-N-tetraose** | 19.9 | 14.6 | **0.04** | 25.3 | 23.6 | 0.54 | 17.4 | 14.9 | 0.32 | 27.0 | 22.6 | 0.12 |
| **Lacto-N-triose II** | 10.2 | 7.9 | 0.39 | 15.2 | 13.2 | 0.18 | 8.8 | 8.1 | 0.96 | **16.9** | **12.1** | **0.02** |
| Lactose-3'-Sulfate | 6.8 | 7.8 | 0.39 | 4.1 | 6.0 | 0.10 | 7.6 | 7.6 | 0.84 | 4.7 | 6.0 | 0.14 |
| Sialyllacto-N-hexaose-X1 | 76.3 | 78.5 | 0.76 | 171.8 | 112.0 | 0.32 | 73.0 | 79.9 | 0.90 | 159.6 | 103.9 | 0.15 |
| Sialyllacto-N-hexaose-X2 | 41.0 | 51.7 | 0.41 | 18.5 | 12.9 | 0.25 | 39.8 | 55.2 | 0.11 | 16.8 | 12.1 | 0.72 |
| Sialyllacto-N-tetraose a | 22.8 | 17.6 | 0.22 | 15.5 | 16.4 | 0.66 | 22.0 | 17.5 | 0.14 | 17.5 | 15.6 | 0.36 |
| Sialyllacto-N-tetraose b | 28.4 | 23.0 | 0.13 | 32.3 | 37.2 | 0.63 | 25.6 | 23.8 | 0.59 | 37.6 | 36.2 | 0.62 |
| Sialyllacto-N-tetraose c | 40.1 | 47.1 | 0.54 | 30.0 | 31.0 | 0.96 | 40.8 | 48.9 | 0.20 | 32.4 | 29.9 | 0.62 |
| Trifucosyl(1-2,1-2,1-3)-iso-lacto-N-octa | 17.6 | 16.9 | 0.98 | 1.1 | 1.1 | 1.00 | 18.4 | 16.2 | 0.91 | 1.1 | 1.1 | 1.00 |
| Trifucosyllacto-N-hexaose I | 25.6 | 29.1 | 0.39 | 2.1 | 2.1 | 1.00 | 27.3 | 28.7 | 0.60 | 2.1 | 2.1 | 1.00 |
| Trifucosyllacto-N-hexaose-X1 | 18.5 | 17.6 | 0.66 | 2.1 | 2.1 | 1.00 | 17.5 | 18.2 | 0.95 | 2.1 | 2.1 | 1.00 |
| Trifucosyllacto-N-octaose-X1 | 33.0 | 30.3 | 0.91 | 2.1 | 2.1 | 1.00 | 30.8 | 30.6 | 0.93 | 2.1 | 2.1 | 1.00 |
| Trifucosyllacto-N-tetraose-X5 | 20.2 | 22.7 | 0.94 | 9.2 | 9.2 | 1.00 | 22.0 | 22.3 | 0.79 | 9.2 | 9.2 | 1.00 |
| a-Heptasaccharide | 10.0 | 17.5 | 0.77 | 2.1 | 2.1 | 1.00 | 16.4 | 15.7 | 0.75 | 2.1 | 2.1 | 1.00 |
| a-Pentasaccharide | 9.4 | 14.0 | 0.94 | 0.5 | 0.5 | 1.00 | 13.6 | 12.8 | 0.78 | 0.5 | 0.5 | 1.00 |
| a-Tetrasaccharide | 6.2 | 10.4 | 0.72 | 0.5 | 0.5 | 1.00 | 8.6 | 10.1 | 0.50 | 0.5 | 0.5 | 1.00 |

p values derived from Wilcoxon sum-rank test comparing HMO levels between infants with and without otitis media at 1 and/or at 2 years. Bold denotes significant at nominal level (p <0.05). None of the differences were statistically significant following correction for multiple testing (FDR<0.010). OM: Otitis media; HMO: human milk oligosaccharides.

**Table S6**: Mean relative levels of human milk oligosaccharides measured at 6 weeks stratified by secretor milk and lower respiratory tract infections

|  | **Secretor milk** | | | **Non-secretor milk** | | | **Secretor milk** | | | **Non-secretor milk** | | |
| --- | --- | --- | --- | --- | --- | --- | --- | --- | --- | --- | --- | --- |
| **HMO Structure** | LRTI Yes (n=14) | LRTI No (n=39) | p | LRTI Yes (n=23) | LRTI No (n=39) | p | LRTI Yes (n=25) | LRTI No (n=33) | p | LRTI Yes (n=32) | LRTI No (n=31) | p |
| 2,3 Difucosyllactose | 12.8 | 14.4 | 0.30 | 1.1 | 1.1 | 1.00 | **13.1** | **14.6** | **0.01** | 1.1 | 1.1 | 1.00 |
| **2-Fucosyllactose** | **24.2** | **30.0** | **0.03** | 2.1 | 2.1 | 1.00 | 27.4 | 29.7 | 0.19 | 2.1 | 2.1 | 1.00 |
| 3'-Sialyl-3-fucosyllactose | 20.8 | 21.8 | 0.44 | 38.8 | 51.3 | 0.81 | 20.8 | 21.9 | 0.56 | 41.9 | 52.5 | 0.42 |
| 3-Fucosyllactose | 25.8 | 25.7 | 0.51 | 60.5 | 67.1 | 0.14 | 24.0 | 26.5 | 0.15 | 54.0 | 70.8 | 0.18 |
| 3-Galactosyllactose | 2.3 | 2.7 | 0.97 | 8.4 | 2.9 | 0.59 | 2.6 | 2.7 | 0.70 | 5.8 | 2.7 | 0.65 |
| 3-sialyllactose | 15.5 | 14.9 | 0.09 | 11.9 | 13.7 | 0.68 | 15.2 | 15.0 | 0.64 | 13.0 | 13.6 | 0.22 |
| 6'-Sialyl-N-acetyllactosamine | 26.9 | 35.2 | 0.50 | 27.8 | 27.6 | 0.12 | 30.9 | 35.0 | 0.53 | 28.3 | 27.0 | 0.23 |
| **6-Galactosyllactose** | 15.2 | 18.8 | 0.23 | 47.8 | 20.3 | 0.43 | **17.2** | **18.7** | **0.01** | 35.1 | 19.6 | 0.25 |
| 6-sialyllactose | 38.7 | 43.9 | 0.24 | 41.2 | 39.4 | 0.53 | 40.4 | 44.3 | 0.23 | 39.1 | 39.6 | 0.54 |
| Difucosyl-lacto-N-hexaose I | 18.3 | 23.6 | 0.65 | 1.1 | 1.1 | 1.00 | 22.4 | 22.6 | 0.91 | 1.1 | 1.1 | 1.00 |
| Difucosyl-lacto-N-hexaose II | 20.5 | 20.8 | 0.72 | 74.2 | 72.1 | 0.41 | 20.6 | 20.5 | 0.22 | 55.4 | 77.2 | 0.15 |
| Difucosyl-lacto-N-hexaose-X1 | 14.9 | 11.1 | 0.60 | 0.5 | 0.5 | 1.00 | 13.6 | 11.4 | 0.78 | 0.5 | 0.5 | 1.00 |
| Difucosyl-lacto-N-hexaose-X2 | 21.7 | 26.9 | 0.80 | 0.5 | 0.5 | 1.00 | 20.9 | 28.5 | 0.30 | 0.5 | 0.5 | 1.00 |
| **Difucosyl-lacto-N-hexaose-X3** | 11.9 | 12.5 | 0.30 | 10.7 | 14.7 | 0.10 | **11.3** | **13.3** | **0.02** | 13.5 | 14.5 | 0.50 |
| **Difucosyl-lacto-N-neo-hexaose I** | 16.3 | 17.2 | 0.23 | 20.8 | 22.5 | 0.31 | **16.6** | **17.2** | **0.01** | 19.8 | 23.0 | 0.24 |
| Difucosyl-para-lacto-N-hexaose I | 14.3 | 12.8 | 0.13 | 37.2 | 39.6 | 0.22 | 13.0 | 13.4 | 0.91 | 33.7 | 41.0 | 0.42 |
| Difucosyldisialyllacto-N-hexaose-X1 | 44.6 | 56.3 | 0.93 | 9.2 | 9.5 | 0.18 | 50.1 | 56.2 | 0.27 | 9.2 | 9.5 | 0.60 |
| Difucosyldisialyllacto-N-hexaose-X2 | 50.2 | 50.8 | 0.50 | 113.8 | 159.3 | 0.61 | 50.9 | 50.2 | 0.15 | 94.8 | 170.0 | 0.98 |
| **Difucosylsialyllacto-N-hexaose-X1** | 36.2 | 46.8 | 0.32 | 4.5 | 4.5 | 1.00 | **39.2** | **47.7** | **0.03** | 4.5 | 4.5 | 1.00 |
| **Difucosylsialyllacto-N-hexaose-X2** | 60.9 | 81.0 | 0.33 | 4.5 | 4.5 | 1.00 | **67.5** | **82.1** | **0.04** | 4.5 | 4.5 | 1.00 |
| **Disialyllacto-N-Hexaose-X1** | 59.5 | 71.0 | 0.48 | **25.5** | **24.2** | **0.03** | 58.2 | 74.4 | 0.99 | 27.3 | 22.6 | 0.27 |
| Disialyllacto-N-Hexaose-X2 | 47.2 | 39.7 | 0.28 | 16.2 | 21.2 | 0.74 | 41.1 | 42.6 | 0.55 | 20.2 | 20.3 | 0.83 |
| **Disialyllacto-N-Hexaose-X5** | 48.5 | 69.0 | 0.10 | 45.8 | 43.3 | 0.16 | **53.9** | **71.2** | **0.02** | 43.6 | 42.8 | 0.34 |
| **Disialyllacto-N-tetraose** | **33.8** | **29.4** | **0.03** | 27.7 | 37.6 | 0.77 | 32.8 | 30.6 | 1.00 | 34.3 | 36.5 | 0.83 |
| **Fucosyl(1-3)-iso-lacto-N-octaose** | **28.5** | **19.9** | **0.002** | 34.7 | 28.6 | 0.75 | 23.7 | 20.4 | 0.11 | 41.2 | 25.1 | 0.72 |
| Fucosyl-para-lacto-N-hexaose I | 20.3 | 13.8 | 0.05 | 12.7 | 14.1 | 0.80 | 17.7 | 14.4 | 0.60 | 18.9 | 12.2 | 0.66 |
| Fucosyldiasialyllacto-N-hexaose-X1 | 49.4 | 51.9 | 0.49 | 48.2 | 61.1 | 0.27 | 51.3 | 51.2 | 0.51 | 51.3 | 61.3 | 0.76 |
| Fucosyldiasialyllacto-N-hexaose-X2 | 34.3 | 31.3 | 0.40 | 23.0 | 33.2 | 0.88 | 32.7 | 32.5 | 0.27 | 33.2 | 31.4 | 0.82 |
| Fucosyldisialyllacto-N-tetraose-X2 | 27.0 | 27.2 | 0.15 | 64.8 | 92.5 | 0.84 | 26.5 | 27.7 | 0.79 | 65.9 | 95.8 | 0.52 |
| Fucosyllacto-N-hexaose II | 27.1 | 25.2 | 0.11 | 50.8 | 41.7 | 0.56 | 26.0 | 25.1 | 0.56 | 49.3 | 40.3 | 1.00 |
| Fucosyllacto-N-hexaose-X1 | 17.9 | 13.1 | 0.42 | 2.1 | 2.1 | 1.00 | 14.5 | 14.1 | 0.91 | 2.1 | 2.1 | 1.00 |
| Fucosyllacto-N-hexaose-X2 | 18.2 | 23.1 | 0.79 | 2.1 | 2.1 | 1.00 | 19.9 | 23.3 | 0.66 | 2.1 | 2.1 | 1.00 |
| **Fucosyllacto-N-hexaose-X4** | 18.4 | 18.2 | 0.88 | 17.0 | 12.7 | 0.81 | **16.1** | **19.6** | **0.03** | 12.4 | 13.0 | 0.48 |
| Fucosyllacto-N-octaose-X1 | 17.9 | 19.8 | 0.53 | 4.5 | 4.5 | 1.00 | 17.5 | 20.5 | 0.97 | 4.5 | 4.5 | 1.00 |
| Fucosyllacto-N-octaose-X2 | 23.8 | 22.5 | 0.11 | 20.5 | 16.6 | 0.53 | 20.9 | 24.0 | 0.56 | 18.7 | 16.1 | 0.82 |
| Fucosyllacto-N-sulfate-X1 | 24.6 | 29.2 | 1.00 | 2.1 | 2.1 | 1.00 | 25.2 | 30.0 | 0.46 | 2.1 | 2.1 | 1.00 |
| Fucosylsialyllacto-N-Hexaose-X1 | 45.9 | 45.3 | 0.07 | 74.8 | 73.4 | 0.64 | 45.0 | 45.1 | 0.71 | 70.5 | 73.9 | 0.58 |
| Fucosylsialyllacto-N-Hexaose-X2 | 39.4 | 42.1 | 0.50 | 40.7 | 37.7 | 0.43 | 44.9 | 39.5 | 1.00 | 40.2 | 36.4 | 0.95 |
| **Fucosylsialyllacto-N-Hexaose-X3** | 37.7 | 50.7 | 1.00 | **1.4** | **1.7** | **0.04** | 49.1 | 47.5 | 0.83 | 1.5 | 1.8 | 0.06 |
| **Fucosylsialyllacto-N-Hexaose-X4** | **52.7** | **60.7** | **0.04** | 55.7 | 55.3 | 0.41 | 52.7 | 62.6 | **0.00** | 54.4 | 55.3 | 0.51 |
| Fucosylsialyllacto-N-Hexaose-X5 | 54.6 | 61.5 | 0.81 | 243.3 | 195.4 | 0.74 | 52.5 | 63.9 | 0.18 | 153.0 | 211.3 | 0.86 |
| Fucosylsialyllacto-N-Hexaose-X6 | 45.6 | 50.7 | 0.15 | 25.4 | 37.0 | 0.78 | 43.1 | 53.8 | **0.01** | 35.3 | 35.5 | 0.64 |
| Fucosylsialyllacto-N-neo-tetraose c | 43.1 | 49.9 | 0.29 | 75.5 | 89.0 | 0.67 | 40.8 | 53.0 | **0.01** | 70.2 | 92.3 | 0.71 |
| Fucosylsialyllacto-N-tetraose a | 24.8 | 22.7 | 0.14 | 63.0 | 69.6 | 1.00 | 23.9 | 23.0 | 0.69 | 52.4 | 72.6 | 0.45 |
| Fucosylsialyllacto-N-tetraose b | 30.1 | 27.8 | 0.11 | 2.1 | 2.1 | 1.00 | 28.6 | 28.5 | 1.00 | 2.1 | 2.1 | 1.00 |
| Fucosylsialyllacto-N-tetraose-X1 | 27.7 | 26.4 | 0.38 | 37.3 | 43.2 | 0.44 | 25.9 | 27.3 | 0.40 | 37.9 | 43.7 | 0.77 |
| Lacto-N-decaose-X1 | 35.2 | 29.3 | 0.07 | 36.7 | 21.9 | 0.27 | 29.5 | 30.3 | 0.48 | 37.8 | 18.4 | 0.86 |
| Lacto-N-difucohexaose I | 18.2 | 20.0 | 0.78 | 0.5 | 0.5 | 1.00 | 18.7 | 20.4 | 0.29 | 0.5 | 0.5 | 1.00 |
| Lacto-N-fucopentaose I | 16.6 | 12.6 | 0.27 | 0.5 | 0.5 | 1.00 | 14.9 | 12.8 | 0.28 | 0.5 | 0.5 | 1.00 |
| **Lacto-N-fucopentaose II** | **14.4** | **12.6** | **0.04** | 48.7 | 45.4 | 0.37 | 12.9 | 13.0 | 0.40 | 39.1 | 47.5 | 0.35 |
| Lacto-N-fucopentaose III | 12.1 | 12.1 | 0.36 | 12.0 | 13.3 | 0.09 | **11.8** | **12.5** | **0.01** | 13.0 | 13.1 | 0.43 |
| **Lacto-N-fucopentaose V** | **17.6** | **13.4** | **0.02** | 59.0 | 57.6 | 0.31 | 15.1 | 14.0 | 0.33 | 54.5 | 58.5 | 0.47 |
| Lacto-N-hexaose | 35.8 | 34.2 | 0.10 | 51.8 | 31.3 | 0.13 | 34.7 | 33.8 | 0.10 | 55.3 | 25.4 | 0.76 |
| Lacto-N-neo-difucohexaose I | 11.5 | 13.4 | 0.20 | 1.1 | 1.1 | 1.00 | **11.3** | **14.0** | **0.01** | 1.1 | 1.1 | 1.00 |
| Lacto-N-neo-difucohexaose II | 12.9 | 14.0 | 0.54 | 39.2 | 48.9 | 0.14 | **12.0** | **14.8** | **0.03** | 38.1 | 51.3 | 0.35 |
| Lacto-N-neo-hexaose | 22.0 | 26.2 | 1.00 | 10.5 | 6.9 | 0.31 | 19.7 | 28.5 | 0.54 | 10.3 | 6.1 | 0.94 |
| Lacto-N-neo-octaose | 28.8 | 25.7 | 0.16 | 15.0 | 12.1 | 0.36 | 25.2 | 26.9 | 0.86 | 17.8 | 10.4 | 0.96 |
| **Lacto-N-tetraose** | **19.9** | **14.6** | **0.004** | 25.3 | 23.6 | 0.97 | 17.4 | 14.9 | 0.07 | 27.0 | 22.6 | 0.92 |
| Lacto-N-triose II | 10.2 | 7.9 | 0.18 | 15.2 | 13.2 | 0.13 | 8.8 | 8.1 | 0.44 | 16.9 | 12.1 | 0.23 |
| Lactose-3'-Sulfate | 6.8 | 7.8 | 0.20 | 4.1 | 6.0 | 0.46 | 7.6 | 7.6 | 0.41 | 4.7 | 6.0 | 0.85 |
| Sialyllacto-N-hexaose-X1 | 76.3 | 78.5 | 0.28 | 171.8 | 112.0 | 0.17 | 73.0 | 79.9 | 0.80 | 159.6 | 103.9 | 0.44 |
| Sialyllacto-N-hexaose-X2 | 41.0 | 51.7 | 0.44 | 18.5 | 12.9 | 0.24 | 39.8 | 55.2 | 0.19 | 16.8 | 12.1 | 0.99 |
| Sialyllacto-N-tetraose a | 22.8 | 17.6 | 0.09 | 15.5 | 16.4 | 0.88 | 22.0 | 17.5 | 0.38 | 17.5 | 15.6 | 0.67 |
| **Sialyllacto-N-tetraose b** | **28.4** | **23.0** | **0.002** | 32.3 | 37.2 | 0.65 | 25.6 | 23.8 | 0.19 | 37.6 | 36.2 | 0.91 |
| Sialyllacto-N-tetraose c | 40.1 | 47.1 | 0.28 | 30.0 | 31.0 | 0.28 | 40.8 | 48.9 | 0.07 | 32.4 | 29.9 | 0.39 |
| Trifucosyl(1-2,1-2,1-3)-iso-lacto-N-octa | 17.6 | 16.9 | 0.39 | 1.1 | 1.1 | 1.00 | 18.4 | 16.2 | 0.47 | 1.1 | 1.1 | 1.00 |
| **Trifucosyllacto-N-hexaose I** | 25.6 | 29.1 | 0.45 | 2.1 | 2.1 | 1.00 | **27.3** | **28.7** | **0.04** | 2.1 | 2.1 | 1.00 |
| Trifucosyllacto-N-hexaose-X1 | 18.5 | 17.6 | 0.95 | 2.1 | 2.1 | 1.00 | 17.5 | 18.2 | 0.06 | 2.1 | 2.1 | 1.00 |
| Trifucosyllacto-N-octaose-X1 | 33.0 | 30.3 | 0.07 | 2.1 | 2.1 | 1.00 | 30.8 | 30.6 | 0.82 | 2.1 | 2.1 | 1.00 |
| Trifucosyllacto-N-tetraose-X5 | 20.2 | 22.7 | 0.66 | 9.2 | 9.2 | 1.00 | 22.0 | 22.3 | 0.50 | 9.2 | 9.2 | 1.00 |
| a-Heptasaccharide | 10.0 | 17.5 | 0.84 | 2.1 | 2.1 | 1.00 | 16.4 | 15.7 | 0.67 | 2.1 | 2.1 | 1.00 |
| a-Pentasaccharide | 9.4 | 14.0 | 0.63 | 0.5 | 0.5 | 1.00 | 13.6 | 12.8 | 0.51 | 0.5 | 0.5 | 1.00 |
| a-Tetrasaccharide | 6.2 | 10.4 | 0.59 | 0.5 | 0.5 | 1.00 | 8.6 | 10.1 | 0.38 | 0.5 | 0.5 | 1.00 |

p values derived from Wilcoxon sum-rank test comparing HMO levels between infants with and without otitis media at 1 and/or at 2 years. **Bold** denotes significant at nominal level (p <0.05). None of the differences were statistically significant following correction for multiple testing (FDR<0.010). LRTI: Lower respiratory tract infection; HMO: human milk oligosaccharides.

**Table S7**: Mean relative levels of human milk oligosaccharides measured at 6 weeks stratified by secretor milk and upper respiratory tract infections

|  | **URTI at 1 year** | | | | | | **URTI at 2 years** | | | | | |
| --- | --- | --- | --- | --- | --- | --- | --- | --- | --- | --- | --- | --- |
|  | **Secretor milk** | | | **Non-secretor milk** | | | **Secretor milk** | | | **Non-secretor milk** | | |
| **HMO Structure** | URTI Yes (n=14) | URTI No (n=39) | p | URTI Yes (n=23) | URTI No (n=39) | p | URTI Yes (n=25) | URTI No (n=33) | p | URTI Yes (n=32) | URTI No (n=31) | p |
| 2,3 Difucosyllactose | 12.8 | 14.4 | 0.73 | 1.1 | 1.1 | 1.00 | 13.1 | 14.6 | 0.75 | 1.1 | 1.1 | 1.00 |
| 2-Fucosyllactose | 24.2 | 30.0 | 0.28 | 2.1 | 2.1 | 1.00 | 27.4 | 29.7 | 0.14 | 2.1 | 2.1 | 1.00 |
| 3'-Sialyl-3-fucosyllactose | 20.8 | 21.8 | 0.32 | 38.8 | 51.3 | 0.48 | 20.8 | 21.9 | 0.19 | 41.9 | 52.5 | 0.53 |
| 3-Fucosyllactose | 25.8 | 25.7 | 0.68 | 60.5 | 67.1 | 0.89 | 24.0 | 26.5 | 0.85 | 54.0 | 70.8 | 0.76 |
| 3-Galactosyllactose | 2.3 | 2.7 | 0.09 | 8.4 | 2.9 | 0.08 | 2.6 | 2.7 | 0.12 | 5.8 | 2.7 | 0.31 |
| 3-sialyllactose | 15.5 | 14.9 | 0.23 | 11.9 | 13.7 | 1.00 | 15.2 | 15.0 | 0.37 | 13.0 | 13.6 | 0.94 |
| 6'-Sialyl-N-acetyllactosamine | 26.9 | 35.2 | 0.43 | 27.8 | 27.6 | 0.69 | 30.9 | 35.0 | 0.99 | 28.3 | 27.0 | 0.30 |
| 6-Galactosyllactose | 15.2 | 18.8 | 0.56 | 47.8 | 20.3 | 0.47 | 17.2 | 18.7 | 1.00 | 35.1 | 19.6 | 0.17 |
| 6-sialyllactose | 38.7 | 43.9 | 0.75 | 41.2 | 39.4 | 0.80 | 40.4 | 44.3 | 0.53 | 39.1 | 39.6 | 0.22 |
| Difucosyl-lacto-N-hexaose I | 18.3 | 23.6 | 0.78 | 1.1 | 1.1 | 1.00 | 22.4 | 22.6 | 1.00 | 1.1 | 1.1 | 1.00 |
| Difucosyl-lacto-N-hexaose II | 20.5 | 20.8 | 0.58 | 74.2 | 72.1 | 0.13 | 20.6 | 20.5 | 0.25 | 55.4 | 77.2 | 0.71 |
| Difucosyl-lacto-N-hexaose-X1 | 14.9 | 11.1 | 0.43 | 0.5 | 0.5 | 1.00 | 13.6 | 11.4 | 0.72 | 0.5 | 0.5 | 1.00 |
| Difucosyl-lacto-N-hexaose-X2 | 21.7 | 26.9 | 0.38 | 0.5 | 0.5 | 1.00 | 20.9 | 28.5 | 0.93 | 0.5 | 0.5 | 1.00 |
| **Difucosyl-lacto-N-hexaose-X3** | 11.9 | 12.5 | 0.16 | 10.7 | 14.7 | 0.31 | **11.3** | **13.3** | **0.02** | 13.5 | 14.5 | 0.13 |
| **Difucosyl-lacto-N-neo-hexaose I** | 16.3 | 17.2 | 0.69 | **20.8** | **22.5** | **0.01** | 16.6 | 17.2 | 0.09 | 19.8 | 23.0 | 0.27 |
| Difucosyl-para-lacto-N-hexaose I | 14.3 | 12.8 | 0.22 | 37.2 | 39.6 | 0.26 | 13.0 | 13.4 | 0.12 | 33.7 | 41.0 | 0.10 |
| Difucosyldisialyllacto-N-hexaose-X1 | 44.6 | 56.3 | 0.52 | 9.2 | 9.5 | 0.97 | 50.1 | 56.2 | 0.86 | 9.2 | 9.5 | 0.48 |
| Difucosyldisialyllacto-N-hexaose-X2 | 50.2 | 50.8 | 0.85 | 113.8 | 159.3 | 0.46 | 50.9 | 50.2 | 0.48 | 94.8 | 170.0 | 0.89 |
| Difucosylsialyllacto-N-hexaose-X1 | 36.2 | 46.8 | 0.27 | 4.5 | 4.5 | 1.00 | 39.2 | 47.7 | 0.72 | 4.5 | 4.5 | 1.00 |
| Difucosylsialyllacto-N-hexaose-X2 | 60.9 | 81.0 | 0.70 | 4.5 | 4.5 | 1.00 | 67.5 | 82.1 | 0.61 | 4.5 | 4.5 | 1.00 |
| Disialyllacto-N-Hexaose-X1 | 59.5 | 71.0 | 0.31 | 25.5 | 24.2 | 0.95 | 58.2 | 74.4 | 0.76 | 27.3 | 22.6 | 0.89 |
| Disialyllacto-N-Hexaose-X2 | 47.2 | 39.7 | 0.17 | 16.2 | 21.2 | 0.25 | 41.1 | 42.6 | 0.26 | 20.2 | 20.3 | 0.27 |
| Disialyllacto-N-Hexaose-X5 | 48.5 | 69.0 | 0.64 | 45.8 | 43.3 | 0.65 | 53.9 | 71.2 | 0.73 | 43.6 | 42.8 | 0.42 |
| Disialyllacto-N-tetraose | 33.8 | 29.4 | 0.64 | 27.7 | 37.6 | 0.89 | 32.8 | 30.6 | 0.49 | 34.3 | 36.5 | 0.35 |
| Fucosyl(1-3)-iso-lacto-N-octaose | 28.5 | 19.9 | 0.16 | 34.7 | 28.6 | 0.64 | 23.7 | 20.4 | 0.06 | 41.2 | 25.1 | 0.71 |
| **Fucosyl-para-lacto-N-hexaose I** | 20.3 | 13.8 | 0.16 | 12.7 | 14.1 | 0.62 | **17.7** | **14.4** | **0.02** | 18.9 | 12.2 | 0.40 |
| **Fucosyldiasialyllacto-N-hexaose-X1** | 49.4 | 51.9 | 0.39 | **48.2** | **61.1** | **0.04** | 51.3 | 51.2 | 0.28 | 51.3 | 61.3 | 0.43 |
| Fucosyldiasialyllacto-N-hexaose-X2 | 34.3 | 31.3 | 0.18 | 23.0 | 33.2 | 0.72 | 32.7 | 32.5 | 0.08 | 33.2 | 31.4 | 0.14 |
| Fucosyldisialyllacto-N-tetraose-X2 | 27.0 | 27.2 | 0.43 | 64.8 | 92.5 | 0.34 | 26.5 | 27.7 | 0.26 | 65.9 | 95.8 | 0.22 |
| Fucosyllacto-N-hexaose II | 27.1 | 25.2 | 0.25 | 50.8 | 41.7 | 0.13 | 26.0 | 25.1 | 0.07 | 49.3 | 40.3 | 0.57 |
| Fucosyllacto-N-hexaose-X1 | 17.9 | 13.1 | 0.53 | 2.1 | 2.1 | 1.00 | 14.5 | 14.1 | 0.94 | 2.1 | 2.1 | 1.00 |
| Fucosyllacto-N-hexaose-X2 | 18.2 | 23.1 | 0.61 | 2.1 | 2.1 | 1.00 | 19.9 | 23.3 | 1.00 | 2.1 | 2.1 | 1.00 |
| Fucosyllacto-N-hexaose-X4 | 18.4 | 18.2 | 0.10 | 17.0 | 12.7 | 0.17 | 16.1 | 19.6 | 0.10 | 12.4 | 13.0 | 0.09 |
| Fucosyllacto-N-octaose-X1 | 17.9 | 19.8 | 0.39 | 4.5 | 4.5 | 1.00 | 17.5 | 20.5 | 0.54 | 4.5 | 4.5 | 1.00 |
| **Fucosyllacto-N-octaose-X2** | 23.8 | 22.5 | 0.09 | 20.5 | 16.6 | 0.84 | **20.9** | **24.0** | **0.04** | 18.7 | 16.1 | 0.88 |
| Fucosyllacto-N-sulfate-X1 | 24.6 | 29.2 | 0.33 | 2.1 | 2.1 | 1.00 | 25.2 | 30.0 | 0.59 | 2.1 | 2.1 | 1.00 |
| **Fucosylsialyllacto-N-Hexaose-X1** | 45.9 | 45.3 | 0.21 | 74.8 | 73.4 | 0.33 | **45.0** | **45.1** | **0.05** | 70.5 | 73.9 | 0.34 |
| **Fucosylsialyllacto-N-Hexaose-X2** | 39.4 | 42.1 | 0.67 | **40.7** | **37.7** | **0.02** | 44.9 | 39.5 | 0.72 | 40.2 | 36.4 | 0.55 |
| Fucosylsialyllacto-N-Hexaose-X3 | 37.7 | 50.7 | 0.77 | 1.4 | 1.7 | 0.37 | 49.1 | 47.5 | 0.53 | 1.5 | 1.8 | 0.75 |
| Fucosylsialyllacto-N-Hexaose-X4 | 52.7 | 60.7 | 0.53 | 55.7 | 55.3 | 0.65 | 52.7 | 62.6 | 0.37 | 54.4 | 55.3 | 0.39 |
| Fucosylsialyllacto-N-Hexaose-X5 | 54.6 | 61.5 | 0.79 | 243.3 | 195.4 | 0.26 | 52.5 | 63.9 | 0.79 | 153.0 | 211.3 | 0.92 |
| **Fucosylsialyllacto-N-Hexaose-X6** | 45.6 | 50.7 | 0.11 | 25.4 | 37.0 | 0.23 | 43.1 | 53.8 | 0.12 | **35.3** | **35.5** | **0.03** |
| Fucosylsialyllacto-N-neo-tetraose c | 43.1 | 49.9 | 0.19 | 75.5 | 89.0 | 0.31 | 40.8 | 53.0 | 0.20 | 70.2 | 92.3 | 0.07 |
| Fucosylsialyllacto-N-tetraose a | 24.8 | 22.7 | 0.82 | 63.0 | 69.6 | 0.34 | 23.9 | 23.0 | 0.52 | 52.4 | 72.6 | 0.65 |
| Fucosylsialyllacto-N-tetraose b | 30.1 | 27.8 | 0.10 | 2.1 | 2.1 | 1.00 | 28.6 | 28.5 | 0.34 | 2.1 | 2.1 | 1.00 |
| **Fucosylsialyllacto-N-tetraose-X1** | 27.7 | 26.4 | 0.11 | 37.3 | 43.2 | 0.23 | **25.9** | **27.3** | **0.01** | 37.9 | 43.7 | 0.19 |
| Lacto-N-decaose-X1 | 35.2 | 29.3 | 0.13 | 36.7 | 21.9 | 0.60 | 29.5 | 30.3 | 0.15 | 37.8 | 18.4 | 0.99 |
| Lacto-N-difucohexaose I | 18.2 | 20.0 | 0.40 | 0.5 | 0.5 | 1.00 | 18.7 | 20.4 | 0.98 | 0.5 | 0.5 | 1.00 |
| Lacto-N-fucopentaose I | 16.6 | 12.6 | 0.56 | 0.5 | 0.5 | 1.00 | 14.9 | 12.8 | 0.79 | 0.5 | 0.5 | 1.00 |
| Lacto-N-fucopentaose II | 14.4 | 12.6 | 0.52 | 48.7 | 45.4 | 0.23 | 12.9 | 13.0 | 0.45 | 39.1 | 47.5 | 0.33 |
| **Lacto-N-fucopentaose III** | 12.1 | 12.1 | 0.41 | 12.0 | 13.3 | 0.83 | **11.8** | **12.5** | **0.05** | 13.0 | 13.1 | 0.66 |
| Lacto-N-fucopentaose V | 17.6 | 13.4 | 0.53 | 59.0 | 57.6 | 0.80 | 15.1 | 14.0 | 0.27 | 54.5 | 58.5 | 0.54 |
| Lacto-N-hexaose | 35.8 | 34.2 | 0.25 | 51.8 | 31.3 | 0.87 | 34.7 | 33.8 | 0.41 | 55.3 | 25.4 | 0.86 |
| Lacto-N-neo-difucohexaose I | 11.5 | 13.4 | 0.32 | 1.1 | 1.1 | 1.00 | 11.3 | 14.0 | 0.27 | 1.1 | 1.1 | 1.00 |
| Lacto-N-neo-difucohexaose II | 12.9 | 14.0 | 0.70 | 39.2 | 48.9 | 0.42 | 12.0 | 14.8 | 0.17 | 38.1 | 51.3 | 0.25 |
| Lacto-N-neo-hexaose | 22.0 | 26.2 | 0.20 | 10.5 | 6.9 | 1.00 | 19.7 | 28.5 | 0.22 | 10.3 | 6.1 | 0.81 |
| Lacto-N-neo-octaose | 28.8 | 25.7 | 0.23 | 15.0 | 12.1 | 0.63 | 25.2 | 26.9 | 0.29 | 17.8 | 10.4 | 0.79 |
| Lacto-N-tetraose | 19.9 | 14.6 | 0.31 | 25.3 | 23.6 | 0.62 | 17.4 | 14.9 | 0.27 | 27.0 | 22.6 | 0.75 |
| Lacto-N-triose II | 10.2 | 7.9 | 0.18 | 15.2 | 13.2 | 0.16 | 8.8 | 8.1 | 0.22 | 16.9 | 12.1 | 0.63 |
| Lactose-3'-Sulfate | 6.8 | 7.8 | 0.45 | 4.1 | 6.0 | 0.56 | 7.6 | 7.6 | 0.60 | 4.7 | 6.0 | 0.33 |
| Sialyllacto-N-hexaose-X1 | 76.3 | 78.5 | 0.37 | 171.8 | 112.0 | 0.25 | 73.0 | 79.9 | 0.49 | 159.6 | 103.9 | 0.95 |
| Sialyllacto-N-hexaose-X2 | 41.0 | 51.7 | 0.55 | 18.5 | 12.9 | 0.72 | 39.8 | 55.2 | 0.90 | 16.8 | 12.1 | 0.46 |
| Sialyllacto-N-tetraose a | 22.8 | 17.6 | 0.75 | 15.5 | 16.4 | 0.32 | 22.0 | 17.5 | 0.66 | 17.5 | 15.6 | 0.59 |
| Sialyllacto-N-tetraose b | 28.4 | 23.0 | 0.16 | 32.3 | 37.2 | 0.07 | 25.6 | 23.8 | 0.13 | 37.6 | 36.2 | 0.34 |
| Sialyllacto-N-tetraose c | 40.1 | 47.1 | 0.31 | 30.0 | 31.0 | 0.22 | 40.8 | 48.9 | 0.94 | 32.4 | 29.9 | 0.17 |
| Trifucosyl(1-2,1-2,1-3)-iso-lacto-N-octa | 17.6 | 16.9 | 0.59 | 1.1 | 1.1 | 1.00 | 18.4 | 16.2 | 0.93 | 1.1 | 1.1 | 1.00 |
| Trifucosyllacto-N-hexaose I | 25.6 | 29.1 | 0.83 | 2.1 | 2.1 | 1.00 | 27.3 | 28.7 | 0.89 | 2.1 | 2.1 | 1.00 |
| Trifucosyllacto-N-hexaose-X1 | 18.5 | 17.6 | 0.24 | 2.1 | 2.1 | 1.00 | 17.5 | 18.2 | 0.32 | 2.1 | 2.1 | 1.00 |
| Trifucosyllacto-N-octaose-X1 | 33.0 | 30.3 | 0.13 | 2.1 | 2.1 | 1.00 | 30.8 | 30.6 | 0.51 | 2.1 | 2.1 | 1.00 |
| Trifucosyllacto-N-tetraose-X5 | 20.2 | 22.7 | 0.57 | 9.2 | 9.2 | 1.00 | 22.0 | 22.3 | 0.99 | 9.2 | 9.2 | 1.00 |
| a-Heptasaccharide | 10.0 | 17.5 | 0.27 | 2.1 | 2.1 | 1.00 | 16.4 | 15.7 | 0.11 | 2.1 | 2.1 | 1.00 |
| a-Pentasaccharide | 9.4 | 14.0 | 0.19 | 0.5 | 0.5 | 1.00 | 13.6 | 12.8 | 0.07 | 0.5 | 0.5 | 1.00 |
| a-Tetrasaccharide | 6.2 | 10.4 | 0.13 | 0.5 | 0.5 | 1.00 | 8.6 | 10.1 | 0.07 | 0.5 | 0.5 | 1.00 |

p values derived from Wilcoxon sum-rank test comparing HMO levels between infants with and without otitis media at 1 and/or at 2 years. None of the differences were statistically significant following correction for multiple testing (FDR <0.010). Bold denotes significant at nominal level (p <0.05). None of the differences were statistically significant following correction for multiple testing (FDR<0.010). URTI: Upper respiratory tract infection; HMO: human milk oligosaccharides.

**Table S8**: Mean relative levels of human milk oligosaccharides measured at 6 months stratified by secretor milk and otitis media

|  | **OM at 1 year** | | | | | | **OM at 2 years** | | | | | |
| --- | --- | --- | --- | --- | --- | --- | --- | --- | --- | --- | --- | --- |
|  | **Secretor milk** | | | **Non-secretor milk** | | | **Secretor milk** | | | **Non-secretor milk** | | |
| **HMO Structure** | OM Yes (n=10) | OM No (n=43) | p | OM Yes (n=6) | OM No (n=54) | p | OM Yes (n=21) | OM No (n=41) | p | OM Yes (n=14) | OM No (n=57) | p |
| 2,3 Difucosyllactose | 17.9 | 17.4 | 0.90 | 1.1 | 1.1 | 1.00 | 17.0 | 17.6 | 0.66 | 1.1 | 1.1 | 1.00 |
| 2-Fucosyllactose | 22.3 | 23.6 | 0.81 | 2.1 | 2.1 | 1.00 | 23.1 | 23.3 | 0.90 | 2.1 | 2.1 | 1.00 |
| 3'-Sialyl-3-fucosyllactose | 33.9 | 28.2 | 0.58 | 89.0 | 68.0 | 0.25 | 30.4 | 28.4 | 0.78 | 70.2 | 69.7 | 0.79 |
| 3-Fucosyllactose | 39.6 | 41.3 | 0.83 | 84.0 | 80.2 | 0.82 | 39.8 | 41.5 | 0.74 | 69.6 | 83.6 | 0.06 |
| **3-Galactosyllactose** | 1.9 | 1.9 | 0.98 | **4.1** | **2.4** | **0.01** | 1.8 | 1.9 | 0.69 | **3.6** | **2.3** | **0.001** |
| 3-sialyllactose | 16.6 | 13.8 | 0.30 | 19.5 | 17.0 | 0.15 | 14.9 | 14.0 | 0.69 | 18.4 | 16.8 | 0.20 |
| 6'-Sialyl-N-acetyllactosamine | 6.9 | 7.9 | 0.83 | 8.7 | 7.9 | 0.38 | 6.9 | 8.1 | 0.64 | 9.3 | 7.5 | 0.23 |
| **6-Galactosyllactose** | 12.2 | 13.3 | 0.78 | **21.3** | **15.4** | **0.03** | 12.7 | 13.2 | 0.68 | 17.9 | 15.2 | 0.11 |
| 6-sialyllactose | 6.6 | 8.5 | 0.40 | 7.4 | 7.8 | 0.80 | 7.3 | 8.5 | 0.54 | 8.1 | 7.7 | 0.73 |
| Difucosyl-lacto-N-hexaose I | 4.7 | 4.6 | 0.71 | 1.1 | 1.1 | 1.00 | 4.6 | 4.5 | 0.89 | 1.1 | 1.1 | 1.00 |
| Difucosyl-lacto-N-hexaose II | 11.8 | 9.3 | 0.22 | 28.2 | 29.6 | 0.99 | 9.9 | 9.5 | 0.98 | 22.3 | 31.6 | 0.07 |
| Difucosyl-lacto-N-hexaose-X1 | 7.2 | 5.6 | 0.15 | 0.5 | 0.5 | 1.00 | 6.6 | 5.6 | 0.30 | 0.5 | 0.5 | 1.00 |
| Difucosyl-lacto-N-hexaose-X2 | 7.2 | 5.4 | 0.25 | 0.5 | 0.5 | 1.00 | 5.2 | 6.0 | 0.58 | 0.5 | 0.5 | 1.00 |
| Difucosyl-lacto-N-hexaose-X3 | 13.5 | 12.5 | 0.58 | 20.0 | 18.0 | 0.29 | 12.6 | 12.7 | 0.97 | 20.1 | 17.6 | 0.42 |
| Difucosyl-lacto-N-neo-hexaose I | 15.2 | 14.8 | 0.76 | 20.0 | 19.5 | 0.79 | 14.7 | 14.8 | 0.90 | 19.8 | 19.5 | 0.95 |
| Difucosyl-para-lacto-N-hexaose I | 14.0 | 10.0 | 0.15 | 38.2 | 30.0 | 0.66 | 11.1 | 10.7 | 0.74 | 31.8 | 30.6 | 0.88 |
| Difucosyldisialyllacto-N-hexaose-X1 | 11.1 | 10.9 | 0.70 | 9.2 | 9.2 | 1.00 | 11.1 | 10.8 | 0.80 | 9.2 | 9.2 | 1.00 |
| Difucosyldisialyllacto-N-hexaose-X2 | 20.6 | 18.2 | 0.35 | 49.2 | 52.9 | 0.85 | 18.5 | 18.5 | 0.99 | 39.3 | 55.6 | 0.06 |
| Difucosylsialyllacto-N-hexaose-X1 | 11.0 | 10.8 | 0.59 | 4.5 | 4.5 | 1.00 | 10.8 | 10.7 | 1.00 | 4.5 | 4.5 | 1.00 |
| Difucosylsialyllacto-N-hexaose-X2 | 6.8 | 8.0 | 0.62 | 4.5 | 4.5 | 1.00 | 7.0 | 8.2 | 0.28 | 4.5 | 4.5 | 1.00 |
| Disialyllacto-N-Hexaose-X1 | 14.5 | 13.2 | 0.46 | 4.5 | 6.4 | 0.17 | 12.1 | 14.1 | 0.85 | 6.3 | 6.1 | 0.16 |
| Disialyllacto-N-Hexaose-X2 | 24.1 | 17.3 | 0.19 | 16.1 | 14.1 | 0.70 | 20.3 | 18.3 | 0.91 | 14.7 | 13.8 | 0.89 |
| Disialyllacto-N-Hexaose-X5 | 3.8 | 4.9 | 0.73 | 3.6 | 4.6 | 0.59 | 4.0 | 5.0 | 0.52 | 6.6 | 3.9 | 0.47 |
| Disialyllacto-N-tetraose | 18.8 | 12.6 | 0.25 | 28.5 | 25.7 | 0.83 | 16.5 | 12.9 | 0.73 | 27.2 | 25.4 | 0.89 |
| Fucosyl(1-3)-iso-lacto-N-octaose | 6.2 | 4.0 | 0.29 | 6.7 | 6.7 | 0.90 | 4.6 | 4.3 | 0.96 | 8.7 | 6.1 | 0.15 |
| Fucosyl-para-lacto-N-hexaose I | 12.7 | 8.2 | 0.16 | 10.9 | 9.8 | 0.96 | 10.3 | 8.7 | 0.82 | 13.8 | 8.8 | 0.05 |
| Fucosyldiasialyllacto-N-hexaose-X1 | 16.4 | 13.2 | 0.33 | 20.5 | 21.7 | 0.90 | 14.7 | 12.9 | 0.75 | 20.4 | 21.8 | 0.77 |
| Fucosyldiasialyllacto-N-hexaose-X2 | 20.1 | 13.6 | 0.20 | 27.8 | 25.1 | 0.73 | 17.5 | 13.8 | 0.57 | 29.4 | 24.1 | 0.34 |
| Fucosyldisialyllacto-N-tetraose-X2 | 27.7 | 18.7 | 0.19 | 80.7 | 68.0 | 0.20 | 22.7 | 19.3 | 0.60 | 66.3 | 69.6 | 0.89 |
| Fucosyllacto-N-hexaose II | 8.7 | 6.6 | 0.31 | 11.2 | 11.7 | 0.67 | 7.3 | 6.7 | 0.85 | 12.8 | 11.4 | 0.49 |
| Fucosyllacto-N-hexaose-X1 | 9.6 | 8.2 | 0.48 | 2.1 | 2.1 | 1.00 | 8.2 | 8.7 | 0.99 | 2.1 | 2.1 | 1.00 |
| Fucosyllacto-N-hexaose-X2 | 5.0 | 4.2 | 0.99 | 2.1 | 2.1 | 1.00 | 4.2 | 4.4 | 0.52 | 2.1 | 2.1 | 1.00 |
| Fucosyllacto-N-hexaose-X4 | 14.6 | 12.4 | 0.37 | 6.3 | 6.8 | 0.95 | 11.9 | 13.2 | 0.81 | 6.9 | 6.6 | 0.99 |
| Fucosyllacto-N-octaose-X1 | 6.7 | 5.6 | 0.96 | 4.5 | 4.5 | 1.00 | 5.9 | 5.8 | 0.68 | 4.5 | 4.5 | 1.00 |
| Fucosyllacto-N-octaose-X2 | 8.8 | 7.6 | 0.51 | 5.0 | 6.2 | 0.56 | 7.2 | 8.2 | 0.80 | 6.2 | 6.0 | 0.75 |
| Fucosyllacto-N-sulfate-X1 | 39.4 | 30.8 | 0.44 | 2.1 | 2.1 | 1.00 | 34.0 | 30.9 | 0.47 | 2.1 | 2.1 | 1.00 |
| **Fucosylsialyllacto-N-Hexaose-X1** | **17.7** | **13.9** | **0.03** | 21.7 | 26.4 | 0.34 | 14.8 | 14.4 | 1.00 | 24.7 | 26.2 | 0.97 |
| Fucosylsialyllacto-N-Hexaose-X2 | 13.9 | 12.1 | 0.19 | 13.2 | 11.8 | 0.99 | 12.9 | 12.1 | 0.34 | 13.2 | 11.5 | 0.85 |
| **Fucosylsialyllacto-N-Hexaose-X3** | 3.1 | 3.4 | 0.83 | 1.8 | 1.5 | 0.44 | 3.4 | 3.3 | 0.91 | **2.1** | **1.4** | **0.002** |
| Fucosylsialyllacto-N-Hexaose-X4 | 10.2 | 11.5 | 0.94 | 11.5 | 12.1 | 0.74 | 10.6 | 11.4 | 0.70 | 14.7 | 11.4 | 0.53 |
| Fucosylsialyllacto-N-Hexaose-X5 | 10.4 | 10.6 | 0.84 | 15.9 | 19.2 | 0.53 | 10.1 | 10.9 | 0.28 | 16.1 | 19.9 | 0.24 |
| Fucosylsialyllacto-N-Hexaose-X6 | 11.8 | 14.2 | 0.59 | 14.8 | 13.8 | 0.37 | 13.3 | 13.8 | 0.87 | 18.5 | 12.7 | 0.20 |
| Fucosylsialyllacto-N-neo-tetraose c | 13.7 | 14.1 | 0.74 | 32.3 | 29.3 | 0.46 | 12.9 | 14.6 | 0.64 | 32.2 | 29.0 | 0.88 |
| Fucosylsialyllacto-N-tetraose a | 16.5 | 15.8 | 0.61 | 65.0 | 47.7 | 0.40 | 15.4 | 16.4 | 0.85 | 48.0 | 50.0 | 0.60 |
| Fucosylsialyllacto-N-tetraose b | 25.4 | 16.2 | 0.22 | 2.1 | 2.1 | 1.00 | 19.8 | 17.0 | 0.88 | 2.1 | 2.1 | 1.00 |
| Fucosylsialyllacto-N-tetraose-X1 | 25.1 | 16.6 | 0.22 | 40.7 | 34.9 | 0.24 | 19.8 | 17.2 | 0.98 | 38.2 | 34.6 | 0.35 |
| Lacto-N-decaose-X1 | 6.1 | 5.6 | 0.27 | 4.8 | 5.1 | 0.28 | 5.6 | 5.7 | 0.77 | 5.5 | 4.9 | 0.38 |
| Lacto-N-difucohexaose I | 14.9 | 10.1 | 0.09 | 0.5 | 0.5 | 1.00 | 11.9 | 10.6 | 0.81 | 0.5 | 0.5 | 1.00 |
| Lacto-N-fucopentaose I | 7.0 | 5.4 | 0.19 | 0.5 | 0.5 | 1.00 | 6.1 | 5.6 | 0.56 | 0.5 | 0.5 | 1.00 |
| Lacto-N-fucopentaose II | 12.8 | 9.9 | 0.26 | 35.8 | 31.4 | 0.82 | 10.8 | 10.5 | 0.80 | 29.4 | 32.6 | 0.47 |
| Lacto-N-fucopentaose III | 13.4 | 12.2 | 0.43 | 16.3 | 15.0 | 0.36 | 12.5 | 12.4 | 0.97 | 16.2 | 14.8 | 0.30 |
| Lacto-N-fucopentaose V | 14.0 | 10.2 | 0.17 | 47.7 | 40.2 | 0.51 | 11.4 | 11.0 | 0.98 | 43.0 | 40.6 | 0.64 |
| Lacto-N-hexaose | 6.3 | 6.2 | 0.58 | 2.1 | 3.1 | 0.82 | 5.0 | 6.9 | 0.91 | 4.3 | 2.5 | 0.34 |
| Lacto-N-neo-difucohexaose I | 11.3 | 9.3 | 0.44 | 1.1 | 1.1 | 1.00 | 9.9 | 9.5 | 0.81 | 1.1 | 1.1 | 1.00 |
| Lacto-N-neo-difucohexaose II | 22.2 | 22.5 | 0.74 | 75.8 | 63.9 | 0.15 | 21.1 | 23.2 | 0.96 | 59.1 | 66.6 | 0.67 |
| Lacto-N-neo-hexaose | 12.0 | 10.6 | 0.70 | 1.5 | 2.6 | 0.59 | 9.2 | 11.8 | 0.52 | 3.6 | 2.2 | 0.22 |
| Lacto-N-neo-octaose | 8.8 | 7.2 | 0.29 | 4.9 | 5.0 | 0.76 | 7.3 | 7.5 | 0.93 | 5.8 | 4.8 | 0.66 |
| Lacto-N-tetraose | 11.9 | 8.7 | 0.08 | 15.8 | 13.9 | 0.80 | 10.1 | 9.2 | 0.82 | 16.7 | 13.4 | 0.06 |
| **Lacto-N-triose II** | 3.5 | 3.3 | 0.44 | 2.9 | 3.4 | 0.77 | 3.2 | 3.5 | 0.81 | **4.8** | **3.0** | **0.02** |
| Lactose-3'-Sulfate | 3.0 | 2.2 | 0.76 | 4.0 | 3.6 | 0.86 | 2.6 | 2.2 | 0.78 | 3.7 | 3.6 | 0.89 |
| Sialyllacto-N-hexaose-X1 | 6.6 | 6.7 | 0.79 | 6.8 | 8.3 | 0.70 | 6.1 | 7.0 | 0.66 | 9.9 | 7.7 | 0.44 |
| Sialyllacto-N-hexaose-X2 | 7.8 | 8.7 | 0.65 | 4.5 | 4.8 | 0.52 | 7.6 | 9.0 | 0.44 | 5.6 | 4.6 | 0.86 |
| Sialyllacto-N-tetraose a | 7.9 | 7.0 | 0.62 | 10.9 | 8.4 | 0.66 | 8.0 | 6.9 | 0.30 | 10.8 | 8.1 | 0.15 |
| Sialyllacto-N-tetraose b | 22.9 | 14.5 | 0.05 | 31.7 | 29.9 | 0.78 | 18.1 | 15.3 | 0.78 | 32.7 | 29.2 | 0.37 |
| Sialyllacto-N-tetraose c | 8.3 | 9.3 | 0.84 | 5.7 | 6.7 | 0.81 | 8.4 | 9.4 | 0.76 | 8.0 | 6.2 | 0.76 |
| Trifucosyl(1-2,1-2,1-3)-iso-lacto-N-octa | 3.4 | 2.8 | 0.46 | 1.1 | 1.1 | 1.00 | 3.0 | 2.8 | 0.98 | 1.1 | 1.1 | 1.00 |
| Trifucosyllacto-N-hexaose I | 10.9 | 9.6 | 0.28 | 2.1 | 2.1 | 1.00 | 9.8 | 9.7 | 0.91 | 2.1 | 2.1 | 1.00 |
| Trifucosyllacto-N-hexaose-X1 | 15.1 | 9.2 | 0.06 | 2.1 | 2.1 | 1.00 | 11.8 | 9.5 | 0.55 | 2.1 | 2.1 | 1.00 |
| Trifucosyllacto-N-octaose-X1 | 9.1 | 5.4 | 0.16 | 2.1 | 2.1 | 1.00 | 6.7 | 5.6 | 0.82 | 2.1 | 2.1 | 1.00 |
| Trifucosyllacto-N-tetraose-X5 | 22.2 | 18.6 | 0.36 | 9.2 | 9.2 | 1.00 | 20.6 | 18.6 | 0.64 | 9.2 | 9.2 | 1.00 |
| a-Heptasaccharide | 11.2 | 11.8 | 0.97 | 2.1 | 2.1 | 1.00 | 13.5 | 10.2 | 0.87 | 2.1 | 2.1 | 1.00 |
| a-Pentasaccharide | 15.0 | 12.9 | 0.82 | 0.5 | 0.5 | 1.00 | 16.1 | 11.0 | 0.86 | 0.5 | 0.5 | 1.00 |
| a-Tetrasaccharide | 7.7 | 8.0 | 0.88 | 0.5 | 0.5 | 1.00 | 8.1 | 7.7 | 0.83 | 0.5 | 0.5 | 1.00 |

p values derived from Wilcoxon sum-rank test comparing HMO levels between infants with and without otitis media at 1 and/or at 2 years. Bold denotes significant at nominal level (p <0.05). None of the differences were statistically significant following correction for multiple testing (FDR<0.010). OM: Otitis media; HMO: human milk oligosaccharides

**Table S9:** Mean relative levels of human milk oligosaccharides measured at 6 months stratified by secretor milk and lower respiratory tract infections

|  | **LRTI at 1 year** | | | | | | **LRTI at 2 years** | | | | | |
| --- | --- | --- | --- | --- | --- | --- | --- | --- | --- | --- | --- | --- |
|  | **Secretor milk** | | | **Non-secretor milk** | | | **Secretor milk** | | | **Non-secretor milk** | | |
| **HMO Structure** | LRTI Yes (n=14) | LRTI No (n=39) | p | LRTI Yes (n=23) | LRTI No (n=39) | p | LRTI Yes (n=25) | LRTI No (n=33) | p | LRTI Yes (n=32) | LRTI No (n=31) | p |
| 2,3 Difucosyllactose | 17.9 | 17.4 | 0.20 | 1.1 | 1.1 | 1.00 | 17.0 | 17.6 | 0.09 | 1.1 | 1.1 | 1.00 |
| 2-Fucosyllactose | 22.3 | 23.6 | 0.13 | 2.1 | 2.1 | 1.00 | 23.1 | 23.3 | 0.64 | 2.1 | 2.1 | 1.00 |
| 3'-Sialyl-3-fucosyllactose | 33.9 | 28.2 | 0.89 | 89.0 | 68.0 | 0.27 | 30.4 | 28.4 | 0.44 | 70.2 | 69.7 | 0.32 |
| 3-Fucosyllactose | 39.6 | 41.3 | 0.42 | 84.0 | 80.2 | 0.45 | 39.8 | 41.5 | 0.15 | 69.6 | 83.6 | 0.25 |
| 3-Galactosyllactose | 1.9 | 1.9 | 0.31 | 4.1 | 2.4 | 0.98 | 1.8 | 1.9 | 0.66 | 3.6 | 2.3 | 0.71 |
| 3-sialyllactose | 16.6 | 13.8 | 0.68 | 19.5 | 17.0 | 0.24 | 14.9 | 14.0 | 0.33 | 18.4 | 16.8 | 0.20 |
| 6'-Sialyl-N-acetyllactosamine | 6.9 | 7.9 | 0.70 | 8.7 | 7.9 | 0.09 | 6.9 | 8.1 | 0.46 | 9.3 | 7.5 | 0.30 |
| 6-Galactosyllactose | 12.2 | 13.3 | 0.41 | 21.3 | 15.4 | 0.27 | 12.7 | 13.2 | 0.37 | 17.9 | 15.2 | 0.42 |
| 6-sialyllactose | 6.6 | 8.5 | 0.56 | 7.4 | 7.8 | 0.38 | 7.3 | 8.5 | 0.66 | 8.1 | 7.7 | 0.98 |
| Difucosyl-lacto-N-hexaose I | 4.7 | 4.6 | 0.55 | 1.1 | 1.1 | 1.00 | 4.6 | 4.5 | 0.06 | 1.1 | 1.1 | 1.00 |
| Difucosyl-lacto-N-hexaose II | 11.8 | 9.3 | 0.31 | 28.2 | 29.6 | 0.64 | 9.9 | 9.5 | 0.84 | 22.3 | 31.6 | 0.85 |
| Difucosyl-lacto-N-hexaose-X1 | 7.2 | 5.6 | 0.23 | 0.5 | 0.5 | 1.00 | 6.6 | 5.6 | 0.06 | 0.5 | 0.5 | 1.00 |
| Difucosyl-lacto-N-hexaose-X2 | 7.2 | 5.4 | 0.32 | 0.5 | 0.5 | 1.00 | 5.2 | 6.0 | 0.77 | 0.5 | 0.5 | 1.00 |
| Difucosyl-lacto-N-hexaose-X3 | 13.5 | 12.5 | 0.47 | 20.0 | 18.0 | 0.61 | 12.6 | 12.7 | 0.22 | 20.1 | 17.6 | 0.38 |
| Difucosyl-lacto-N-neo-hexaose I | 15.2 | 14.8 | 0.13 | 20.0 | 19.5 | 0.76 | 14.7 | 14.8 | 0.18 | 19.8 | 19.5 | 0.42 |
| Difucosyl-para-lacto-N-hexaose I | 14.0 | 10.0 | 0.28 | 38.2 | 30.0 | 0.99 | 11.1 | 10.7 | 0.82 | 31.8 | 30.6 | 0.90 |
| Difucosyldisialyllacto-N-hexaose-X1 | 11.1 | 10.9 | 0.84 | 9.2 | 9.2 | 1.00 | 11.1 | 10.8 | 0.87 | 9.2 | 9.2 | 1.00 |
| Difucosyldisialyllacto-N-hexaose-X2 | 20.6 | 18.2 | 0.90 | 49.2 | 52.9 | 0.28 | 18.5 | 18.5 | 0.89 | 39.3 | 55.6 | 0.34 |
| Difucosylsialyllacto-N-hexaose-X1 | 11.0 | 10.8 | 0.78 | 4.5 | 4.5 | 1.00 | 10.8 | 10.7 | 0.92 | 4.5 | 4.5 | 1.00 |
| Difucosylsialyllacto-N-hexaose-X2 | 6.8 | 8.0 | 0.38 | 4.5 | 4.5 | 1.00 | 7.0 | 8.2 | 0.42 | 4.5 | 4.5 | 1.00 |
| Disialyllacto-N-Hexaose-X1 | 14.5 | 13.2 | 0.75 | 4.5 | 6.4 | 0.93 | 12.1 | 14.1 | 0.44 | 6.3 | 6.1 | 0.56 |
| Disialyllacto-N-Hexaose-X2 | 24.1 | 17.3 | 0.28 | 16.1 | 14.1 | 0.47 | 20.3 | 18.3 | 0.36 | 14.7 | 13.8 | 0.81 |
| **Disialyllacto-N-Hexaose-X5** | 3.8 | 4.9 | 0.65 | **3.6** | **4.6** | **0.04** | 4.0 | 5.0 | 0.80 | 6.6 | 3.9 | 0.05 |
| Disialyllacto-N-tetraose | 18.8 | 12.6 | 0.76 | 28.5 | 25.7 | 0.39 | 16.5 | 12.9 | 0.16 | 27.2 | 25.4 | 0.83 |
| **Fucosyl(1-3)-iso-lacto-N-octaose** | **6.2** | **4.0** | **0.04** | 6.7 | 6.7 | 0.65 | **4.6** | **4.3** | **0.02** | 8.7 | 6.1 | 0.66 |
| Fucosyl-para-lacto-N-hexaose I | 12.7 | 8.2 | 0.08 | 10.9 | 9.8 | 0.26 | 10.3 | 8.7 | 0.17 | 13.8 | 8.8 | 0.29 |
| Fucosyldiasialyllacto-N-hexaose-X1 | 16.4 | 13.2 | 0.93 | 20.5 | 21.7 | 0.21 | 14.7 | 12.9 | 0.18 | 20.4 | 21.8 | 0.62 |
| Fucosyldiasialyllacto-N-hexaose-X2 | 20.1 | 13.6 | 0.86 | 27.8 | 25.1 | 0.47 | 17.5 | 13.8 | 0.35 | 29.4 | 24.1 | 0.80 |
| Fucosyldisialyllacto-N-tetraose-X2 | 27.7 | 18.7 | 0.58 | 80.7 | 68.0 | 0.24 | 22.7 | 19.3 | 0.96 | 66.3 | 69.6 | 0.65 |
| Fucosyllacto-N-hexaose II | 8.7 | 6.6 | 0.15 | 11.2 | 11.7 | 0.49 | 7.3 | 6.7 | 0.11 | 12.8 | 11.4 | 0.56 |
| Fucosyllacto-N-hexaose-X1 | 9.6 | 8.2 | 0.37 | 2.1 | 2.1 | 1.00 | 8.2 | 8.7 | 0.49 | 2.1 | 2.1 | 1.00 |
| Fucosyllacto-N-hexaose-X2 | 5.0 | 4.2 | 0.35 | 2.1 | 2.1 | 1.00 | 4.2 | 4.4 | 0.28 | 2.1 | 2.1 | 1.00 |
| Fucosyllacto-N-hexaose-X4 | 14.6 | 12.4 | 0.84 | 6.3 | 6.8 | 0.95 | 11.9 | 13.2 | 0.69 | 6.9 | 6.6 | 0.91 |
| Fucosyllacto-N-octaose-X1 | 6.7 | 5.6 | 0.91 | 4.5 | 4.5 | 1.00 | 5.9 | 5.8 | 0.71 | 4.5 | 4.5 | 1.00 |
| Fucosyllacto-N-octaose-X2 | 8.8 | 7.6 | 0.29 | 5.0 | 6.2 | 0.90 | 7.2 | 8.2 | 0.71 | 6.2 | 6.0 | 0.66 |
| Fucosyllacto-N-sulfate-X1 | 39.4 | 30.8 | 0.22 | 2.1 | 2.1 | 1.00 | 34.0 | 30.9 | 0.59 | 2.1 | 2.1 | 1.00 |
| Fucosylsialyllacto-N-Hexaose-X1 | 17.7 | 13.9 | 0.12 | 21.7 | 26.4 | 0.14 | 14.8 | 14.4 | 0.29 | 24.7 | 26.2 | 0.21 |
| Fucosylsialyllacto-N-Hexaose-X2 | 13.9 | 12.1 | 0.59 | 13.2 | 11.8 | 0.77 | 12.9 | 12.1 | 0.09 | 13.2 | 11.5 | 0.42 |
| Fucosylsialyllacto-N-Hexaose-X3 | 3.1 | 3.4 | 0.70 | 1.8 | 1.5 | 0.80 | 3.4 | 3.3 | 0.23 | 2.1 | 1.4 | 0.44 |
| Fucosylsialyllacto-N-Hexaose-X4 | 10.2 | 11.5 | 0.19 | 11.5 | 12.1 | 0.11 | 10.6 | 11.4 | 0.72 | 14.7 | 11.4 | 0.23 |
| Fucosylsialyllacto-N-Hexaose-X5 | 10.4 | 10.6 | 0.52 | 15.9 | 19.2 | 0.07 | 10.1 | 10.9 | 0.58 | 16.1 | 19.9 | 0.14 |
| Fucosylsialyllacto-N-Hexaose-X6 | 11.8 | 14.2 | 0.50 | 14.8 | 13.8 | 0.86 | 13.3 | 13.8 | 0.65 | 18.5 | 12.7 | 0.99 |
| Fucosylsialyllacto-N-neo-tetraose c | 13.7 | 14.1 | 0.98 | 32.3 | 29.3 | 0.07 | 12.9 | 14.6 | 0.41 | 32.2 | 29.0 | 0.29 |
| Fucosylsialyllacto-N-tetraose a | 16.5 | 15.8 | 0.65 | 65.0 | 47.7 | 0.66 | 15.4 | 16.4 | 0.51 | 48.0 | 50.0 | 0.43 |
| Fucosylsialyllacto-N-tetraose b | 25.4 | 16.2 | 0.11 | 2.1 | 2.1 | 1.00 | 19.8 | 17.0 | 0.09 | 2.1 | 2.1 | 1.00 |
| Fucosylsialyllacto-N-tetraose-X1 | 25.1 | 16.6 | 0.49 | 40.7 | 34.9 | 0.37 | 19.8 | 17.2 | 0.63 | 38.2 | 34.6 | 0.76 |
| Lacto-N-decaose-X1 | 6.1 | 5.6 | 0.56 | 4.8 | 5.1 | 0.21 | 5.6 | 5.7 | 0.35 | 5.5 | 4.9 | 0.64 |
| Lacto-N-difucohexaose I | 14.9 | 10.1 | 0.34 | 0.5 | 0.5 | 1.00 | 11.9 | 10.6 | 0.35 | 0.5 | 0.5 | 1.00 |
| **Lacto-N-fucopentaose I** | 7.0 | 5.4 | 0.10 | 0.5 | 0.5 | 1.00 | **6.1** | **5.6** | **0.03** | 0.5 | 0.5 | 1.00 |
| Lacto-N-fucopentaose II | 12.8 | 9.9 | 0.08 | 35.8 | 31.4 | 0.55 | 10.8 | 10.5 | 0.38 | 29.4 | 32.6 | 0.27 |
| Lacto-N-fucopentaose III | 13.4 | 12.2 | 0.57 | 16.3 | 15.0 | 0.44 | 12.5 | 12.4 | 0.38 | 16.2 | 14.8 | 0.32 |
| **Lacto-N-fucopentaose V** | **14.0** | **10.2** | **0.05** | 47.7 | 40.2 | 0.63 | 11.4 | 11.0 | 0.28 | 43.0 | 40.6 | 0.93 |
| Lacto-N-hexaose | 6.3 | 6.2 | 0.08 | 2.1 | 3.1 | 0.74 | 5.0 | 6.9 | 0.08 | 4.3 | 2.5 | 0.92 |
| Lacto-N-neo-difucohexaose I | 11.3 | 9.3 | 0.79 | 1.1 | 1.1 | 1.00 | 9.9 | 9.5 | 1.00 | 1.1 | 1.1 | 1.00 |
| Lacto-N-neo-difucohexaose II | 22.2 | 22.5 | 0.62 | 75.8 | 63.9 | 0.75 | 21.1 | 23.2 | 0.07 | 59.1 | 66.6 | 0.73 |
| Lacto-N-neo-hexaose | 12.0 | 10.6 | 0.50 | 1.5 | 2.6 | 0.72 | 9.2 | 11.8 | 0.99 | 3.6 | 2.2 | 0.49 |
| Lacto-N-neo-octaose | 8.8 | 7.2 | 0.65 | 4.9 | 5.0 | 0.39 | 7.3 | 7.5 | 0.55 | 5.8 | 4.8 | 0.37 |
| **Lacto-N-tetraose** | **11.9** | **8.7** | **0.01** | 15.8 | 13.9 | 0.60 | 10.1 | 9.2 | 0.08 | 16.7 | 13.4 | 0.81 |
| **Lacto-N-triose II** | **3.5** | **3.3** | **0.001** | **2.9** | **3.4** | **0.02** | 3.2 | 3.5 | 0.07 | 4.8 | 3.0 | 0.32 |
| Lactose-3'-Sulfate | 3.0 | 2.2 | 0.75 | 4.0 | 3.6 | 0.88 | 2.6 | 2.2 | 0.62 | 3.7 | 3.6 | 0.42 |
| Sialyllacto-N-hexaose-X1 | 6.6 | 6.7 | 0.72 | 6.8 | 8.3 | 0.18 | 6.1 | 7.0 | 0.82 | 9.9 | 7.7 | 0.38 |
| **Sialyllacto-N-hexaose-X2** | 7.8 | 8.7 | 0.82 | 4.5 | 4.8 | 0.12 | 7.6 | 9.0 | 0.89 | **5.6** | **4.6** | **0.04** |
| Sialyllacto-N-tetraose a | 7.9 | 7.0 | 0.31 | 10.9 | 8.4 | 0.52 | **8.0** | **6.9** | **0.01** | 10.8 | 8.1 | 0.55 |
| Sialyllacto-N-tetraose b | 22.9 | 14.5 | 0.08 | 31.7 | 29.9 | 0.69 | 18.1 | 15.3 | 0.27 | 32.7 | 29.2 | 0.89 |
| Sialyllacto-N-tetraose c | 8.3 | 9.3 | 0.90 | 5.7 | 6.7 | 0.18 | 8.4 | 9.4 | 0.92 | 8.0 | 6.2 | 0.45 |
| Trifucosyl(1-2,1-2,1-3)-iso-lacto-N-octa | 3.4 | 2.8 | 0.26 | 1.1 | 1.1 | 1.00 | **3.0** | **2.8** | **0.03** | 1.1 | 1.1 | 1.00 |
| Trifucosyllacto-N-hexaose I | 10.9 | 9.6 | 0.64 | 2.1 | 2.1 | 1.00 | 9.8 | 9.7 | 0.85 | 2.1 | 2.1 | 1.00 |
| Trifucosyllacto-N-hexaose-X1 | 15.1 | 9.2 | 0.54 | 2.1 | 2.1 | 1.00 | 11.8 | 9.5 | 0.83 | 2.1 | 2.1 | 1.00 |
| **Trifucosyllacto-N-octaose-X1** | **9.1** | **5.4** | **0.01** | 2.1 | 2.1 | 1.00 | **6.7** | **5.6** | **0.04** | 2.1 | 2.1 | 1.00 |
| Trifucosyllacto-N-tetraose-X5 | 22.2 | 18.6 | 0.76 | 9.2 | 9.2 | 1.00 | 20.6 | 18.6 | 0.67 | 9.2 | 9.2 | 1.00 |
| a-Heptasaccharide | 11.2 | 11.8 | 0.86 | 2.1 | 2.1 | 1.00 | 13.5 | 10.2 | 0.91 | 2.1 | 2.1 | 1.00 |
| a-Pentasaccharide | 15.0 | 12.9 | 0.55 | 0.5 | 0.5 | 1.00 | 16.1 | 11.0 | 0.86 | 0.5 | 0.5 | 1.00 |
| a-Tetrasaccharide | 7.7 | 8.0 | 0.39 | 0.5 | 0.5 | 1.00 | 8.1 | 7.7 | 0.64 | 0.5 | 0.5 | 1.00 |

p values derived from Wilcoxon sum-rank test comparing HMO levels between infants with and without otitis media at 1 and/or at 2 years. Bold denotes significant at nominal level (p <0.05). None of the differences were statistically significant following correction for multiple testing (FDR<0.010). LRTI: Lower respiratory tract infection; HMO: human milk oligosaccharides.

**Table S10**: Mean relative levels of human milk oligosaccharides measured at 6 months stratified by secretor milk and upper respiratory tract infections

|  | **URTI at 1 year** | | | | | | **URTI at 2 years** | | | | | |
| --- | --- | --- | --- | --- | --- | --- | --- | --- | --- | --- | --- | --- |
|  | **Secretor milk** | | | **Non-secretor milk** | | | **Secretor milk** | | | **Non-secretor milk** | | |
| **HMO Structure** | URTI Yes (n=14) | URTI No (n=39) | p | URTI Yes (n=23) | URTI No (n=39) | p | URTI Yes (n=25) | URTI No (n=33) | p | URTI Yes (n=32) | URTI No (n=31) | p |
| 2,3 Difucosyllactose | 17.9 | 17.4 | 0.80 | 1.1 | 1.1 | 1.00 | 17.0 | 17.6 | 0.58 | 1.1 | 1.1 | 1.00 |
| 2-Fucosyllactose | 22.3 | 23.6 | 0.37 | 2.1 | 2.1 | 1.00 | 23.1 | 23.3 | 0.17 | 2.1 | 2.1 | 1.00 |
| **3'-Sialyl-3-fucosyllactose** | 33.9 | 28.2 | 0.31 | **89.0** | **68.0** | **0.01** | 30.4 | 28.4 | 0.67 | **70.2** | **69.7** | **0.04** |
| 3-Fucosyllactose | 39.6 | 41.3 | 0.86 | 84.0 | 80.2 | 0.30 | 39.8 | 41.5 | 0.71 | 69.6 | 83.6 | 0.56 |
| 3-Galactosyllactose | 1.9 | 1.9 | 0.15 | 4.1 | 2.4 | 0.17 | 1.8 | 1.9 | 0.25 | 3.6 | 2.3 | 0.98 |
| 3-sialyllactose | 16.6 | 13.8 | 0.63 | 19.5 | 17.0 | 0.05 | 14.9 | 14.0 | 0.44 | 18.4 | 16.8 | 0.07 |
| 6'-Sialyl-N-acetyllactosamine | 6.9 | 7.9 | 0.56 | 8.7 | 7.9 | 0.07 | 6.9 | 8.1 | 0.73 | 9.3 | 7.5 | 0.14 |
| 6-Galactosyllactose | 12.2 | 13.3 | 0.99 | 21.3 | 15.4 | 0.55 | 12.7 | 13.2 | 0.48 | 17.9 | 15.2 | 0.68 |
| **6-sialyllactose** | 6.6 | 8.5 | 0.81 | **7.4** | **7.8** | **0.03** | 7.3 | 8.5 | 0.53 | **8.1** | **7.7** | **0.001** |
| Difucosyl-lacto-N-hexaose I | 4.7 | 4.6 | 0.81 | 1.1 | 1.1 | 1.00 | 4.6 | 4.5 | 0.26 | 1.1 | 1.1 | 1.00 |
| Difucosyl-lacto-N-hexaose II | 11.8 | 9.3 | 0.25 | 28.2 | 29.6 | 0.82 | 9.9 | 9.5 | 0.56 | 22.3 | 31.6 | 0.53 |
| Difucosyl-lacto-N-hexaose-X1 | 7.2 | 5.6 | 0.49 | 0.5 | 0.5 | 1.00 | 6.6 | 5.6 | 0.95 | 0.5 | 0.5 | 1.00 |
| Difucosyl-lacto-N-hexaose-X2 | 7.2 | 5.4 | 0.31 | 0.5 | 0.5 | 1.00 | 5.2 | 6.0 | 0.56 | 0.5 | 0.5 | 1.00 |
| Difucosyl-lacto-N-hexaose-X3 | 13.5 | 12.5 | 0.06 | 20.0 | 18.0 | 0.08 | 12.6 | 12.7 | 0.28 | 20.1 | 17.6 | 0.13 |
| Difucosyl-lacto-N-neo-hexaose I | 15.2 | 14.8 | 0.42 | 20.0 | 19.5 | 0.65 | 14.7 | 14.8 | 0.36 | 19.8 | 19.5 | 0.48 |
| **Difucosyl-para-lacto-N-hexaose I** | 14.0 | 10.0 | 0.10 | **38.2** | **30.0** | **0.03** | 11.1 | 10.7 | 0.21 | 31.8 | 30.6 | 0.09 |
| Difucosyldisialyllacto-N-hexaose-X1 | 11.1 | 10.9 | 0.60 | 9.2 | 9.2 | 1.00 | 11.1 | 10.8 | 0.29 | 9.2 | 9.2 | 1.00 |
| Difucosyldisialyllacto-N-hexaose-X2 | 20.6 | 18.2 | 0.63 | 49.2 | 52.9 | 0.46 | 18.5 | 18.5 | 0.45 | 39.3 | 55.6 | 0.25 |
| Difucosylsialyllacto-N-hexaose-X1 | 11.0 | 10.8 | 0.32 | 4.5 | 4.5 | 1.00 | 10.8 | 10.7 | 0.77 | 4.5 | 4.5 | 1.00 |
| Difucosylsialyllacto-N-hexaose-X2 | 6.8 | 8.0 | 0.40 | 4.5 | 4.5 | 1.00 | 7.0 | 8.2 | 0.20 | 4.5 | 4.5 | 1.00 |
| Disialyllacto-N-Hexaose-X1 | 14.5 | 13.2 | 0.29 | 4.5 | 6.4 | 0.52 | 12.1 | 14.1 | 0.85 | 6.3 | 6.1 | 0.45 |
| Disialyllacto-N-Hexaose-X2 | 24.1 | 17.3 | 0.18 | 16.1 | 14.1 | **0.05** | 20.3 | 18.3 | 0.69 | 14.7 | 13.8 | 0.11 |
| **Disialyllacto-N-Hexaose-X5** | 3.8 | 4.9 | 0.26 | 3.6 | 4.6 | 0.06 | 4.0 | 5.0 | 0.95 | **6.6** | **3.9** | **0.03** |
| **Disialyllacto-N-tetraose** | 18.8 | 12.6 | 0.78 | 28.5 | 25.7 | 0.14 | 16.5 | 12.9 | 0.15 | **27.2** | **25.4** | **0.02** |
| Fucosyl(1-3)-iso-lacto-N-octaose | 6.2 | 4.0 | 0.60 | 6.7 | 6.7 | 0.96 | 4.6 | 4.3 | 0.98 | 8.7 | 6.1 | 0.29 |
| Fucosyl-para-lacto-N-hexaose I | 12.7 | 8.2 | 0.22 | 10.9 | 9.8 | 0.29 | 10.3 | 8.7 | 0.48 | 13.8 | 8.8 | 0.18 |
| Fucosyldiasialyllacto-N-hexaose-X1 | 16.4 | 13.2 | 0.61 | 20.5 | 21.7 | 0.91 | 14.7 | 12.9 | 0.32 | 20.4 | 21.8 | 0.36 |
| **Fucosyldiasialyllacto-N-hexaose-X2** | 20.1 | 13.6 | 0.15 | 27.8 | 25.1 | 0.06 | 17.5 | 13.8 | 0.66 | **29.4** | **24.1** | **0.01** |
| **Fucosyldisialyllacto-N-tetraose-X2** | 27.7 | 18.7 | 0.39 | **80.7** | **68.0** | **0.03** | 22.7 | 19.3 | 0.84 | 66.3 | 69.6 | 0.10 |
| Fucosyllacto-N-hexaose II | 8.7 | 6.6 | 0.57 | 11.2 | 11.7 | 0.57 | 7.3 | 6.7 | 0.77 | 12.8 | 11.4 | 0.54 |
| Fucosyllacto-N-hexaose-X1 | 9.6 | 8.2 | 0.56 | 2.1 | 2.1 | 1.00 | 8.2 | 8.7 | 0.72 | 2.1 | 2.1 | 1.00 |
| Fucosyllacto-N-hexaose-X2 | 5.0 | 4.2 | 0.53 | 2.1 | 2.1 | 1.00 | 4.2 | 4.4 | 0.36 | 2.1 | 2.1 | 1.00 |
| **Fucosyllacto-N-hexaose-X4** | **14.6** | **12.4** | **0.03** | **6.3** | **6.8** | **0.01** | 11.9 | 13.2 | 0.23 | 6.9 | 6.6 | 0.28 |
| Fucosyllacto-N-octaose-X1 | 6.7 | 5.6 | 0.71 | 4.5 | 4.5 | 1.00 | 5.9 | 5.8 | 0.40 | 4.5 | 4.5 | 1.00 |
| Fucosyllacto-N-octaose-X2 | 8.8 | 7.6 | 0.31 | 5.0 | 6.2 | 0.79 | 7.2 | 8.2 | 0.36 | 6.2 | 6.0 | 0.69 |
| Fucosyllacto-N-sulfate-X1 | 39.4 | 30.8 | 0.23 | 2.1 | 2.1 | 1.00 | 34.0 | 30.9 | 0.54 | 2.1 | 2.1 | 1.00 |
| Fucosylsialyllacto-N-Hexaose-X1 | 17.7 | 13.9 | 0.39 | 21.7 | 26.4 | 0.24 | 14.8 | 14.4 | 0.57 | 24.7 | 26.2 | 0.06 |
| **Fucosylsialyllacto-N-Hexaose-X2** | 13.9 | 12.1 | 0.83 | **13.2** | **11.8** | **0.02** | 12.9 | 12.1 | 0.46 | 13.2 | 11.5 | 0.25 |
| Fucosylsialyllacto-N-Hexaose-X3 | 3.1 | 3.4 | 0.42 | 1.8 | 1.5 | 0.13 | 3.4 | 3.3 | 0.09 | 2.1 | 1.4 | 0.07 |
| **Fucosylsialyllacto-N-Hexaose-X4** | 10.2 | 11.5 | 0.16 | **11.5** | **12.1** | **0.05** | 10.6 | 11.4 | 0.79 | **14.7** | **11.4** | **0.02** |
| **Fucosylsialyllacto-N-Hexaose-X5** | 10.4 | 10.6 | 0.46 | 15.9 | 19.2 | 0.41 | 10.1 | 10.9 | 0.64 | **16.1** | **19.9** | **0.05** |
| **Fucosylsialyllacto-N-Hexaose-X6** | 11.8 | 14.2 | 0.44 | **14.8** | **13.8** | **0.02** | 13.3 | 13.8 | 0.91 | **18.5** | **12.7** | **0.03** |
| **Fucosylsialyllacto-N-neo-tetraose c** | **13.7** | **14.1** | **0.03** | 32.3 | 29.3 | 0.06 | 12.9 | 14.6 | 0.35 | **32.2** | **29.0** | **0.03** |
| Fucosylsialyllacto-N-tetraose a | 16.5 | 15.8 | 0.89 | 65.0 | 47.7 | 0.83 | 15.4 | 16.4 | 0.67 | 48.0 | 50.0 | 0.36 |
| Fucosylsialyllacto-N-tetraose b | 25.4 | 16.2 | 0.82 | 2.1 | 2.1 | 1.00 | 19.8 | 17.0 | 0.46 | 2.1 | 2.1 | 1.00 |
| **Fucosylsialyllacto-N-tetraose-X1** | **25.1** | **16.6** | **0.01** | **40.7** | **34.9** | **0.01** | 19.8 | 17.2 | 0.18 | 38.2 | 34.6 | 0.07 |
| Lacto-N-decaose-X1 | 6.1 | 5.6 | 0.53 | 4.8 | 5.1 | 0.40 | 5.6 | 5.7 | 0.74 | 5.5 | 4.9 | 0.46 |
| Lacto-N-difucohexaose I | 14.9 | 10.1 | 0.74 | 0.5 | 0.5 | 1.00 | 11.9 | 10.6 | 0.91 | 0.5 | 0.5 | 1.00 |
| Lacto-N-fucopentaose I | 7.0 | 5.4 | 0.95 | 0.5 | 0.5 | 1.00 | 6.1 | 5.6 | 0.41 | 0.5 | 0.5 | 1.00 |
| **Lacto-N-fucopentaose II** | 12.8 | 9.9 | 0.17 | **35.8** | **31.4** | **0.02** | 10.8 | 10.5 | 0.39 | **29.4** | **32.6** | **0.02** |
| Lacto-N-fucopentaose III | 13.4 | 12.2 | 0.09 | 16.3 | 15.0 | 0.19 | 12.5 | 12.4 | 0.36 | 16.2 | 14.8 | 0.23 |
| Lacto-N-fucopentaose V | 14.0 | 10.2 | 0.18 | 47.7 | 40.2 | 0.18 | 11.4 | 11.0 | 0.33 | **43.0** | **40.6** | **0.04** |
| Lacto-N-hexaose | 6.3 | 6.2 | 0.26 | 2.1 | 3.1 | 0.56 | 5.0 | 6.9 | 0.88 | 4.3 | 2.5 | 0.63 |
| Lacto-N-neo-difucohexaose I | 11.3 | 9.3 | 0.11 | 1.1 | 1.1 | 1.00 | 9.9 | 9.5 | 0.78 | 1.1 | 1.1 | 1.00 |
| **Lacto-N-neo-difucohexaose II** | 22.2 | 22.5 | 0.09 | **75.8** | **63.9** | **0.05** | 21.1 | 23.2 | 0.15 | 59.1 | 66.6 | 0.30 |
| Lacto-N-neo-hexaose | 12.0 | 10.6 | 0.22 | 1.5 | 2.6 | 0.11 | 9.2 | 11.8 | 0.46 | 3.6 | 2.2 | 0.51 |
| Lacto-N-neo-octaose | 8.8 | 7.2 | 0.24 | 4.9 | 5.0 | 0.53 | 7.3 | 7.5 | 0.52 | 5.8 | 4.8 | 0.24 |
| Lacto-N-tetraose | 11.9 | 8.7 | 0.30 | 15.8 | 13.9 | 0.39 | 10.1 | 9.2 | 0.59 | 16.7 | 13.4 | 0.16 |
| Lacto-N-triose II | 3.5 | 3.3 | 0.06 | 2.9 | 3.4 | 0.19 | 3.2 | 3.5 | 0.73 | 4.8 | 3.0 | 0.38 |
| Lactose-3'-Sulfate | 3.0 | 2.2 | 0.71 | 4.0 | 3.6 | 0.08 | 2.6 | 2.2 | 0.64 | 3.7 | 3.6 | 0.10 |
| Sialyllacto-N-hexaose-X1 | 6.6 | 6.7 | 0.37 | 6.8 | 8.3 | 0.71 | 6.1 | 7.0 | 0.76 | **9.9** | **7.7** | **0.05** |
| Sialyllacto-N-hexaose-X2 | 7.8 | 8.7 | 0.55 | 4.5 | 4.8 | 0.73 | 7.6 | 9.0 | 0.91 | 5.6 | 4.6 | 0.42 |
| Sialyllacto-N-tetraose a | 7.9 | 7.0 | 0.86 | 10.9 | 8.4 | 0.54 | 8.0 | 6.9 | 0.74 | 10.8 | 8.1 | 0.58 |
| **Sialyllacto-N-tetraose b** | 22.9 | 14.5 | 0.47 | **31.7** | **29.9** | **0.01** | 18.1 | 15.3 | 0.78 | **32.7** | **29.2** | **0.01** |
| Sialyllacto-N-tetraose c | 8.3 | 9.3 | 0.09 | 5.7 | 6.7 | 0.05 | 8.4 | 9.4 | 0.81 | **8.0** | **6.2** | **0.02** |
| Trifucosyl(1-2,1-2,1-3)-iso-lacto-N-octa | 3.4 | 2.8 | 0.66 | 1.1 | 1.1 | 1.00 | 3.0 | 2.8 | 0.28 | 1.1 | 1.1 | 1.00 |
| Trifucosyllacto-N-hexaose I | 10.9 | 9.6 | 0.88 | 2.1 | 2.1 | 1.00 | 9.8 | 9.7 | 0.61 | 2.1 | 2.1 | 1.00 |
| Trifucosyllacto-N-hexaose-X1 | 15.1 | 9.2 | 0.27 | 2.1 | 2.1 | 1.00 | 11.8 | 9.5 | 0.47 | 2.1 | 2.1 | 1.00 |
| Trifucosyllacto-N-octaose-X1 | 9.1 | 5.4 | 0.49 | 2.1 | 2.1 | 1.00 | 6.7 | 5.6 | 0.80 | 2.1 | 2.1 | 1.00 |
| Trifucosyllacto-N-tetraose-X5 | 22.2 | 18.6 | 0.94 | 9.2 | 9.2 | 1.00 | 20.6 | 18.6 | 0.82 | 9.2 | 9.2 | 1.00 |
| a-Heptasaccharide | 11.2 | 11.8 | 0.18 | 2.1 | 2.1 | 1.00 | 13.5 | 10.2 | 0.14 | 2.1 | 2.1 | 1.00 |
| a-Pentasaccharide | 15.0 | 12.9 | 0.14 | 0.5 | 0.5 | 1.00 | 16.1 | 11.0 | 0.15 | 0.5 | 0.5 | 1.00 |
| a-Tetrasaccharide | 7.7 | 8.0 | 0.16 | 0.5 | 0.5 | 1.00 | 8.1 | 7.7 | 0.15 | 0.5 | 0.5 | 1.00 |

p values derived from Wilcoxon sum-rank test comparing HMO levels between infants with and without otitis media at 1 and/or at 2 years. None of the differences were statistically significant following correction for multiple testing (FDR <0.010). **Bold** denotes significant at nominal level (p <0.05). None of the differences were statistically significant following correction for multiple testing (FDR<0.010). URTI: Upper respiratory tract infection; HMO: human milk oligosaccharides


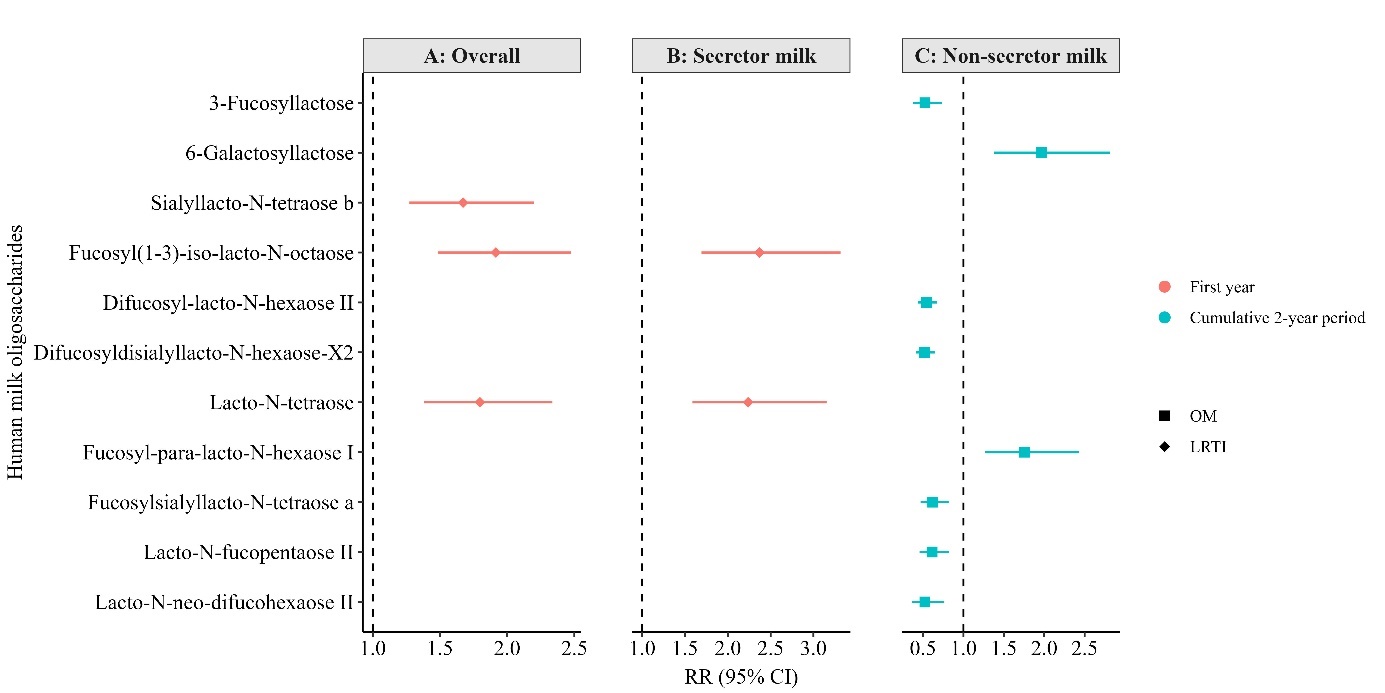


**Figure S1:** Crude associations between human milk oligosaccharides in overall i.e. non-stratified (**A**), secretor (**B**) and non-secretor milk (**C**) measured at 6 weeks of lactation with infections in the first or second year of life in the Ulm SPATZ Health Study. Associations determined by modified Poisson regression. Models adjusted for batch, child sex, duration of exclusive breastfeeding (weeks) and gestation age (weeks). OM: Otitis media; LRTI: Lower respiratory tract infections; URTI: Upper respiratory tract infections; RR: Risk Ratio determined using weighted secretor status for panel A only; CI: Confidence intervals.

**Table S11:** Adjusted associations between human milk oligosaccharides measured at 6 weeks of lactation with otitis media in the first or second year of life in the Ulm SPATZ Health Study.

|  | **OM at 1 year** | | **OM at 2 years** | |
| --- | --- | --- | --- | --- |
| **HMO Structure** | RR (95% CI) | p | RR (95% CI) | p |
| 2,3 Difucosyllactose | 1.09 (0.74, 1.60) | 0.67 | 0.94 (0.69, 1.29) | 0.71 |
| 2-Fucosyllactose | 0.78 (0.54, 1.11) | 0.16 | 1.00 (0.74, 1.35) | 0.99 |
| 3'-Sialyl-3-fucosyllactose | 0.86 (0.60, 1.23) | 0.39 | 0.78 (0.59, 1.04) | 0.09 |
| **3-Fucosyllactose** | 0.80 (0.53, 1.20) | 0.28 | **0.68 (0.52, 0.90)** | **0.01** |
| 3-Galactosyllactose | 0.82 (0.53, 1.27) | 0.37 | 1.02 (0.77, 1.36) | 0.89 |
| 3-sialyllactose | 0.92 (0.51, 1.65) | 0.79 | 1.02 (0.76, 1.37) | 0.89 |
| 6'-Sialyl-N-acetyllactosamine | 0.71 (0.46, 1.09) | 0.11 | 0.88 (0.65, 1.20) | 0.42 |
| 6-Galactosyllactose | 0.71 (0.46, 1.11) | 0.13 | 0.96 (0.72, 1.28) | 0.79 |
| 6-sialyllactose | 0.81 (0.51, 1.30) | 0.39 | 0.85 (0.63, 1.13) | 0.27 |
| Difucosyl-lacto-N-hexaose I | 1.02 (0.65, 1.59) | 0.94 | 1.23 (0.90, 1.67) | 0.19 |
| **Difucosyl-lacto-N-hexaose II** | 0.89 (0.59, 1.33) | 0.57 | **0.74 (0.56, 0.98)** | **0.03** |
| **Difucosyl-lacto-N-hexaose-X1** | 1.70 (0.97, 2.97) | 0.06 | **1.40 (1.02, 1.93)** | **0.04** |
| Difucosyl-lacto-N-hexaose-X2 | 1.09 (0.71, 1.65) | 0.70 | 0.94 (0.69, 1.27) | 0.68 |
| Difucosyl-lacto-N-hexaose-X3 | 0.94 (0.67, 1.32) | 0.71 | 0.84 (0.65, 1.10) | 0.20 |
| Difucosyl-lacto-N-neo-hexaose I | 0.86 (0.60, 1.21) | 0.38 | 0.88 (0.70, 1.11) | 0.29 |
| Difucosyl-para-lacto-N-hexaose I | 1.08 (0.76, 1.54) | 0.66 | 0.80 (0.60, 1.07) | 0.14 |
| Difucosyldisialyllacto-N-hexaose-X1 | 0.78 (0.51, 1.20) | 0.26 | 1.02 (0.71, 1.46) | 0.93 |
| Difucosyldisialyllacto-N-hexaose-X2 | 0.88 (0.60, 1.28) | 0.50 | 0.79 (0.60, 1.03) | 0.08 |
| Difucosylsialyllacto-N-hexaose-X1 | 0.88 (0.56, 1.40) | 0.60 | 1.00 (0.70, 1.44) | 0.99 |
| Difucosylsialyllacto-N-hexaose-X2 | 0.96 (0.67, 1.37) | 0.82 | 0.97 (0.72, 1.30) | 0.83 |
| Disialyllacto-N-Hexaose-X1 | 1.02 (0.62, 1.67) | 0.93 | 1.06 (0.78, 1.44) | 0.70 |
| Disialyllacto-N-Hexaose-X2 | 1.31 (0.70, 2.46) | 0.40 | 1.07 (0.77, 1.48) | 0.69 |
| Disialyllacto-N-Hexaose-X5 | 0.75 (0.48, 1.16) | 0.19 | 0.82 (0.61, 1.10) | 0.18 |
| Disialyllacto-N-tetraose | 1.04 (0.58, 1.87) | 0.90 | 0.99 (0.74, 1.33) | 0.95 |
| Fucosyl(1-3)-iso-lacto-N-octaose | 1.32 (0.66, 2.63) | 0.43 | 1.19 (0.86, 1.64) | 0.29 |
| Fucosyl-para-lacto-N-hexaose I | 1.61 (0.83, 3.16) | 0.16 | 1.23 (0.89, 1.70) | 0.21 |
| Fucosyldiasialyllacto-N-hexaose-X1 | 0.74 (0.44, 1.26) | 0.27 | 0.95 (0.71, 1.28) | 0.73 |
| Fucosyldiasialyllacto-N-hexaose-X2 | 0.95 (0.49, 1.84) | 0.87 | 0.96 (0.69, 1.34) | 0.82 |
| Fucosyldisialyllacto-N-tetraose-X2 | 0.92 (0.62, 1.35) | 0.67 | 0.79 (0.61, 1.03) | 0.08 |
| Fucosyllacto-N-hexaose II | 1.01 (0.57, 1.79) | 0.97 | 1.02 (0.77, 1.34) | 0.90 |
| Fucosyllacto-N-hexaose-X1 | 1.33 (0.78, 2.26) | 0.30 | 1.10 (0.79, 1.54) | 0.57 |
| Fucosyllacto-N-hexaose-X2 | 0.97 (0.60, 1.57) | 0.91 | 1.09 (0.78, 1.51) | 0.62 |
| Fucosyllacto-N-hexaose-X4 | 1.21 (0.80, 1.84) | 0.36 | 0.85 (0.64, 1.13) | 0.27 |
| Fucosyllacto-N-octaose-X1 | 1.06 (0.65, 1.72) | 0.81 | 1.09 (0.78, 1.52) | 0.61 |
| Fucosyllacto-N-octaose-X2 | 1.31 (0.81, 2.12) | 0.27 | 0.96 (0.73, 1.27) | 0.79 |
| Fucosyllacto-N-sulfate-X1 | 1.07 (0.68, 1.67) | 0.78 | 1.12 (0.82, 1.52) | 0.47 |
| Fucosylsialyllacto-N-Hexaose-X1 | 0.88 (0.52, 1.49) | 0.63 | 0.91 (0.69, 1.18) | 0.47 |
| Fucosylsialyllacto-N-Hexaose-X2 | 1.02 (0.64, 1.64) | 0.92 | 1.22 (0.91, 1.65) | 0.19 |
| Fucosylsialyllacto-N-Hexaose-X3 | 0.98 (0.64, 1.52) | 0.94 | 1.12 (0.83, 1.52) | 0.44 |
| Fucosylsialyllacto-N-Hexaose-X4 | 0.83 (0.54, 1.29) | 0.41 | 0.79 (0.59, 1.06) | 0.12 |
| **Fucosylsialyllacto-N-Hexaose-X5** | 0.88 (0.54, 1.42) | 0.59 | **0.68 (0.50, 0.93)** | **0.01** |
| Fucosylsialyllacto-N-Hexaose-X6 | 0.94 (0.63, 1.40) | 0.76 | 0.86 (0.64, 1.16) | 0.32 |
| **Fucosylsialyllacto-N-neo-tetraose c** | 0.83 (0.57, 1.20) | 0.31 | **0.68 (0.52, 0.89)** | **0.004** |
| Fucosylsialyllacto-N-tetraose a | 0.99 (0.68, 1.43) | 0.94 | 0.82 (0.63, 1.07) | 0.15 |
| Fucosylsialyllacto-N-tetraose b | 1.54 (0.91, 2.61) | 0.11 | 1.25 (0.89, 1.75) | 0.19 |
| Fucosylsialyllacto-N-tetraose-X1 | 0.90 (0.56, 1.46) | 0.67 | 0.83 (0.63, 1.10) | 0.19 |
| Lacto-N-decaose-X1 | 1.18 (0.62, 2.26) | 0.61 | 1.15 (0.84, 1.57) | 0.39 |
| Lacto-N-difucohexaose I | 1.04 (0.72, 1.50) | 0.83 | 0.98 (0.72, 1.33) | 0.91 |
| **Lacto-N-fucopentaose I** | **1.53 (1.01, 2.31)** | **0.04** | **1.35 (1.03, 1.77)** | **0.03** |
| Lacto-N-fucopentaose II | 1.03 (0.69, 1.53) | 0.88 | 0.75 (0.56, 1.00) | 0.05 |
| Lacto-N-fucopentaose III | 0.95 (0.66, 1.35) | 0.76 | 0.90 (0.70, 1.16) | 0.41 |
| Lacto-N-fucopentaose V | 1.12 (0.75, 1.67) | 0.57 | 0.85 (0.65, 1.12) | 0.26 |
| Lacto-N-hexaose | 1.17 (0.70, 1.94) | 0.55 | 1.14 (0.84, 1.55) | 0.40 |
| Lacto-N-neo-difucohexaose I | 0.91 (0.60, 1.39) | 0.67 | 0.93 (0.66, 1.31) | 0.69 |
| **Lacto-N-neo-difucohexaose II** | 0.87 (0.61, 1.23) | 0.42 | **0.75 (0.57, 0.98)** | **0.03** |
| Lacto-N-neo-hexaose | 1.09 (0.73, 1.62) | 0.67 | 0.94 (0.71, 1.25) | 0.69 |
| Lacto-N-neo-octaose | 1.28 (0.74, 2.21) | 0.38 | 1.14 (0.84, 1.55) | 0.41 |
| Lacto-N-tetraose | 1.55 (0.80, 3.01) | 0.19 | 1.14 (0.83, 1.55) | 0.41 |
| Lacto-N-triose II | 1.33 (0.76, 2.31) | 0.31 | 1.02 (0.72, 1.46) | 0.90 |
| Lactose-3'-Sulfate | 0.76 (0.47, 1.25) | 0.29 | 1.03 (0.75, 1.41) | 0.85 |
| Sialyllacto-N-hexaose-X1 | 1.07 (0.58, 1.97) | 0.83 | 1.01 (0.74, 1.38) | 0.96 |
| Sialyllacto-N-hexaose-X2 | 1.07 (0.72, 1.58) | 0.75 | 0.93 (0.71, 1.22) | 0.59 |
| Sialyllacto-N-tetraose a | 1.21 (0.58, 2.53) | 0.61 | 1.23 (0.84, 1.81) | 0.28 |
| Sialyllacto-N-tetraose b | 1.24 (0.73, 2.09) | 0.43 | 1.00 (0.76, 1.33) | 0.98 |
| Sialyllacto-N-tetraose c | 0.93 (0.63, 1.35) | 0.69 | 0.89 (0.66, 1.20) | 0.45 |
| Trifucosyl(1-2,1-2,1-3)-iso-lacto-N-octa | 1.17 (0.71, 1.93) | 0.54 | 1.24 (0.90, 1.71) | 0.18 |
| Trifucosyllacto-N-hexaose I | 1.10 (0.75, 1.62) | 0.62 | 1.13 (0.82, 1.57) | 0.45 |
| Trifucosyllacto-N-hexaose-X1 | 1.36 (0.90, 2.07) | 0.15 | 1.11 (0.79, 1.55) | 0.56 |
| Trifucosyllacto-N-octaose-X1 | 1.38 (0.87, 2.18) | 0.18 | 1.13 (0.82, 1.55) | 0.44 |
| Trifucosyllacto-N-tetraose-X5 | 1.06 (0.71, 1.59) | 0.77 | 1.01 (0.75, 1.36) | 0.94 |
| a-Heptasaccharide | 1.08 (0.64, 1.83) | 0.77 | 1.02 (0.75, 1.40) | 0.88 |
| a-Pentasaccharide | 1.20 (0.70, 2.04) | 0.50 | 1.08 (0.79, 1.47) | 0.62 |
| a-Tetrasaccharide | 1.11 (0.61, 2.00) | 0.73 | 1.00 (0.72, 1.40) | 0.99 |

Associations determined by modified Poisson regression. Models adjusted for batch, child sex, duration of exclusive breastfeeding (weeks) and gestation age (weeks). Estimates are weighted to reflect 80% secretors and 20% non-secretors in the full study population Bold denotes statistically significant at nominal level (p<0.05). *Denotes statistically significant after correction for multiple testing (FDR<0.010). OM: Otitis media; HMO: Human milk oligosaccharides; RR: weighted Risk Ratio; CI: Confidence Interval.

**Table S12:** Adjusted associations between human milk oligosaccharides measured at 6 weeks of lactation with lower respiratory tract infections in the first or second year of life in the Ulm SPATZ Health Study.

|  | **LRTI at 1 year** | | **LRTI at 2 years** | |
| --- | --- | --- | --- | --- |
| **HMO Structure** | RR (95% CI) | p | RR (95% CI) | p |
| **2,3 Difucosyllactose** | 0.73 (0.53, 1.03) | 0.07 | **0.75 (0.60, 0.93)** | **0.01** |
| **2-Fucosyllactose** | **0.59 (0.40, 0.88)** | **0.01** | 0.89 (0.69, 1.16) | 0.40 |
| 3'-Sialyl-3-fucosyllactose | 1.20 (0.86, 1.66) | 0.28 | 0.96 (0.77, 1.21) | 0.74 |
| 3-Fucosyllactose | 0.93 (0.65, 1.32) | 0.69 | 0.82 (0.65, 1.04) | 0.10 |
| 3-Galactosyllactose | 1.00 (0.72, 1.39) | 1.00 | 0.97 (0.79, 1.19) | 0.78 |
| 3-sialyllactose | 1.28 (0.87, 1.88) | 0.21 | 1.18 (0.95, 1.47) | 0.13 |
| 6'-Sialyl-N-acetyllactosamine | 0.95 (0.67, 1.36) | 0.79 | 1.00 (0.79, 1.26) | 0.98 |
| 6-Galactosyllactose | 0.81 (0.60, 1.10) | 0.18 | 0.75 (0.62, 0.90) | **0.00** |
| 6-sialyllactose | 0.89 (0.63, 1.25) | 0.49 | 0.94 (0.74, 1.19) | 0.61 |
| Difucosyl-lacto-N-hexaose I | 0.77 (0.52, 1.16) | 0.21 | 1.06 (0.82, 1.38) | 0.66 |
| Difucosyl-lacto-N-hexaose II | 1.13 (0.79, 1.63) | 0.50 | 0.87 (0.69, 1.09) | 0.23 |
| Difucosyl-lacto-N-hexaose-X1 | 0.92 (0.59, 1.42) | 0.69 | 1.07 (0.83, 1.37) | 0.59 |
| Difucosyl-lacto-N-hexaose-X2 | 0.82 (0.56, 1.21) | 0.32 | 0.85 (0.65, 1.10) | 0.21 |
| **Difucosyl-lacto-N-hexaose-X3** | 0.76 (0.56, 1.04) | 0.09 | **0.78 (0.64, 0.96)** | **0.02** |
| **Difucosyl-lacto-N-neo-hexaose I** | 0.81 (0.57, 1.14) | 0.22 | **0.77 (0.65, 0.92)** | **0.004** |
| Difucosyl-para-lacto-N-hexaose I | 1.22 (0.89, 1.69) | 0.22 | 0.93 (0.74, 1.18) | 0.56 |
| Difucosyldisialyllacto-N-hexaose-X1 | 0.80 (0.51, 1.26) | 0.34 | 0.98 (0.74, 1.30) | 0.89 |
| Difucosyldisialyllacto-N-hexaose-X2 | 1.27 (0.88, 1.83) | 0.20 | 0.89 (0.71, 1.11) | 0.29 |
| **Difucosylsialyllacto-N-hexaose-X1** | **0.64 (0.43, 0.97)** | **0.03** | 0.86 (0.64, 1.14) | 0.29 |
| **Difucosylsialyllacto-N-hexaose-X2** | **0.67 (0.45, 0.98)** | **0.04** | **0.75 (0.58, 0.98)** | **0.03** |
| Disialyllacto-N-Hexaose-X1 | 1.23 (0.86, 1.76) | 0.26 | 1.11 (0.88, 1.39) | 0.37 |
| Disialyllacto-N-Hexaose-X2 | 1.17 (0.76, 1.82) | 0.48 | 1.03 (0.81, 1.31) | 0.80 |
| Disialyllacto-N-Hexaose-X5 | 0.78 (0.54, 1.11) | 0.17 | 0.83 (0.64, 1.07) | 0.16 |
| **Disialyllacto-N-tetraose** | **1.50 (1.07, 2.10)** | **0.02** | 1.08 (0.86, 1.35) | 0.50 |
| **Fucosyl(1-3)-iso-lacto-N-octaose** | **1.92 (1.47, 2.51)** | **<0.001*** | **1.30 (1.07, 1.57)** | **0.01** |
| **Fucosyl-para-lacto-N-hexaose I** | **1.45 (1.07, 1.96)** | **0.02** | 1.17 (0.95, 1.44) | 0.13 |
| Fucosyldiasialyllacto-N-hexaose-X1 | 1.36 (0.96, 1.91) | 0.08 | 1.04 (0.84, 1.30) | 0.71 |
| Fucosyldiasialyllacto-N-hexaose-X2 | 1.19 (0.82, 1.72) | 0.37 | 0.98 (0.78, 1.23) | 0.86 |
| Fucosyldisialyllacto-N-tetraose-X2 | 1.35 (0.98, 1.85) | 0.06 | 0.98 (0.78, 1.23) | 0.88 |
| **Fucosyllacto-N-hexaose II** | **1.53 (1.14, 2.05)** | **0.004** | 1.11 (0.91, 1.36) | 0.30 |
| Fucosyllacto-N-hexaose-X1 | 1.09 (0.72, 1.63) | 0.69 | 1.12 (0.89, 1.40) | 0.33 |
| Fucosyllacto-N-hexaose-X2 | 0.88 (0.58, 1.33) | 0.54 | 1.05 (0.82, 1.34) | 0.71 |
| Fucosyllacto-N-hexaose-X4 | 0.91 (0.60, 1.38) | 0.64 | 0.79 (0.61, 1.01) | 0.06 |
| Fucosyllacto-N-octaose-X1 | 1.04 (0.70, 1.53) | 0.86 | 1.13 (0.90, 1.42) | 0.30 |
| Fucosyllacto-N-octaose-X2 | 1.36 (0.93, 1.98) | 0.12 | 1.00 (0.77, 1.30) | 0.99 |
| Fucosyllacto-N-sulfate-X1 | 0.86 (0.55, 1.33) | 0.50 | 1.01 (0.78, 1.29) | 0.97 |
| **Fucosylsialyllacto-N-Hexaose-X1** | **1.50 (1.11, 2.02)** | **0.01** | 1.10 (0.90, 1.34) | 0.37 |
| Fucosylsialyllacto-N-Hexaose-X2 | 1.17 (0.82, 1.67) | 0.40 | 1.03 (0.82, 1.31) | 0.79 |
| Fucosylsialyllacto-N-Hexaose-X3 | 0.80 (0.55, 1.15) | 0.23 | 1.03 (0.81, 1.32) | 0.79 |
| **Fucosylsialyllacto-N-Hexaose-X4** | **0.71 (0.52, 0.97)** | **0.03** | **0.78 (0.62, 0.99)** | **0.04** |
| Fucosylsialyllacto-N-Hexaose-X5 | 1.22 (0.85, 1.75) | 0.28 | 0.90 (0.71, 1.13) | 0.35 |
| Fucosylsialyllacto-N-Hexaose-X6 | 0.75 (0.53, 1.05) | 0.09 | 0.78 (0.62, 0.98) | **0.03** |
| Fucosylsialyllacto-N-neo-tetraose c | 0.94 (0.65, 1.37) | 0.76 | 0.81 (0.65, 1.01) | 0.07 |
| Fucosylsialyllacto-N-tetraose a | 1.32 (0.93, 1.87) | 0.12 | 0.94 (0.74, 1.20) | 0.61 |
| Fucosylsialyllacto-N-tetraose b | 1.07 (0.71, 1.62) | 0.74 | 1.09 (0.84, 1.41) | 0.53 |
| Fucosylsialyllacto-N-tetraose-X1 | 1.11 (0.76, 1.62) | 0.58 | 0.93 (0.74, 1.18) | 0.56 |
| **Lacto-N-decaose-X1** | **1.52 (1.10, 2.11)** | **0.01** | 1.19 (0.96, 1.47) | 0.11 |
| Lacto-N-difucohexaose I | 0.75 (0.53, 1.07) | 0.11 | 0.83 (0.65, 1.07) | 0.16 |
| Lacto-N-fucopentaose I | 1.12 (0.75, 1.67) | 0.57 | 1.19 (0.96, 1.47) | 0.11 |
| Lacto-N-fucopentaose II | 1.36 (0.99, 1.87) | 0.06 | 0.98 (0.77, 1.25) | 0.85 |
| **Lacto-N-fucopentaose III** | 0.80 (0.59, 1.09) | 0.16 | **0.79 (0.64, 0.96)** | **0.02** |
| **Lacto-N-fucopentaose V** | **1.45 (1.10, 1.92)** | **0.01** | 1.01 (0.80, 1.27) | 0.94 |
| **Lacto-N-hexaose** | **1.51 (1.13, 2.01)** | **0.005** | **1.24 (1.01, 1.52)** | **0.04** |
| **Lacto-N-neo-difucohexaose I** | **0.60 (0.42, 0.86)** | **0.005** | **0.76 (0.58, 0.98)** | **0.04** |
| **Lacto-N-neo-difucohexaose II** | 0.92 (0.65, 1.31) | 0.64 | **0.80 (0.64, 1.00)** | **0.05** |
| Lacto-N-neo-hexaose | 0.92 (0.64, 1.34) | 0.67 | 0.95 (0.75, 1.19) | 0.63 |
| Lacto-N-neo-octaose | 1.29 (0.88, 1.89) | 0.20 | 1.14 (0.90, 1.44) | 0.28 |
| **Lacto-N-tetraose** | **1.83 (1.43, 2.35)** | **<0.001*** | **1.26 (1.02, 1.55)** | **0.03** |
| Lacto-N-triose II | **1.56 (1.16, 2.10)** | **0.003** | 1.12 (0.89, 1.40) | 0.34 |
| Lactose-3'-Sulfate | 1.21 (0.85, 1.72) | 0.29 | 1.01 (0.80, 1.28) | 0.93 |
| **Sialyllacto-N-hexaose-X1** | **1.45 (1.04, 2.03)** | **0.03** | 1.10 (0.89, 1.35) | 0.37 |
| Sialyllacto-N-hexaose-X2 | 0.81 (0.52, 1.27) | 0.36 | 0.90 (0.70, 1.16) | 0.42 |
| **Sialyllacto-N-tetraose a** | **1.49 (1.05, 2.12)** | **0.03** | **1.27 (1.02, 1.59)** | **0.04** |
| **Sialyllacto-N-tetraose b** | **1.67 (1.26, 2.21)** | **<0.001*** | 1.17 (0.93, 1.46) | 0.18 |
| Sialyllacto-N-tetraose c | 0.84 (0.58, 1.23) | 0.38 | 0.88 (0.69, 1.13) | 0.32 |
| Trifucosyl(1-2,1-2,1-3)-iso-lacto-N-octa | 1.00 (0.66, 1.51) | 0.99 | 1.20 (0.94, 1.54) | 0.15 |
| Trifucosyllacto-N-hexaose I | 0.78 (0.50, 1.23) | 0.28 | 0.81 (0.60, 1.07) | 0.14 |
| Trifucosyllacto-N-hexaose-X1 | 0.72 (0.49, 1.07) | 0.11 | 0.78 (0.60, 1.01) | 0.06 |
| Trifucosyllacto-N-octaose-X1 | 1.08 (0.69, 1.70) | 0.74 | 1.07 (0.81, 1.41) | 0.64 |
| Trifucosyllacto-N-tetraose-X5 | 0.86 (0.59, 1.26) | 0.43 | 0.91 (0.71, 1.17) | 0.45 |
| a-Heptasaccharide | 0.94 (0.60, 1.46) | 0.77 | 1.03 (0.81, 1.31) | 0.81 |
| a-Pentasaccharide | 1.02 (0.68, 1.53) | 0.92 | 1.08 (0.86, 1.36) | 0.52 |
| a-Tetrasaccharide | 1.09 (0.69, 1.72) | 0.73 | 1.12 (0.88, 1.44) | 0.36 |

Associations determined by modified Poisson regression. Models adjusted for batch, child sex, duration of exclusive breastfeeding (weeks) and gestation age (weeks). Bold denotes statistically significant at nominal level (p<0.05). *Denotes statistically significant after correction for multiple testing (FDR<0.010). LRTI: lower respiratory infections; HMO: Human milk oligosaccharides. RR: weighted Risk Ratio determined using weighted secretor status; CI: Confidence Interval.

**Table S13:** Adjusted associations between human milk oligosaccharides measured at 6 weeks of lactation with upper respiratory tract infections in the first or second year of life in the Ulm SPATZ Health Study.

|  | **URTI at 1 year** | | **URTI at 2 years** | |
| --- | --- | --- | --- | --- |
| **HMO Structure** | RR (95% CI) | p | RR (95% CI) | p |
| 2,3 Difucosyllactose | 1.10 (0.95, 1.27) | 0.19 | 1.02 (0.90, 1.15) | 0.79 |
| 2-Fucosyllactose | 0.96 (0.80, 1.15) | 0.62 | 0.90 (0.79, 1.02) | 0.09 |
| 3'-Sialyl-3-fucosyllactose | 1.06 (0.91, 1.23) | 0.49 | 1.08 (0.99, 1.19) | 0.10 |
| 3-Fucosyllactose | 0.97 (0.83, 1.14) | 0.69 | 1.04 (0.94, 1.15) | 0.47 |
| 3-Galactosyllactose | 1.12 (0.98, 1.29) | 0.10 | 1.09 (1.00, 1.18) | 0.05 |
| 3-sialyllactose | 1.08 (0.93, 1.25) | 0.32 | 1.03 (0.94, 1.14) | 0.47 |
| 6'-Sialyl-N-acetyllactosamine | 1.06 (0.92, 1.22) | 0.44 | 1.00 (0.92, 1.09) | 1.00 |
| 6-Galactosyllactose | 1.01 (0.87, 1.17) | 0.86 | 1.02 (0.93, 1.12) | 0.63 |
| 6-sialyllactose | 1.01 (0.89, 1.16) | 0.84 | 0.98 (0.90, 1.06) | 0.57 |
| Difucosyl-lacto-N-hexaose I | 1.04 (0.88, 1.23) | 0.67 | 0.96 (0.87, 1.07) | 0.49 |
| Difucosyl-lacto-N-hexaose II | 0.97 (0.83, 1.13) | 0.72 | 1.06 (0.96, 1.17) | 0.27 |
| Difucosyl-lacto-N-hexaose-X1 | 1.10 (0.93, 1.28) | 0.26 | 0.98 (0.89, 1.08) | 0.69 |
| Difucosyl-lacto-N-hexaose-X2 | 1.07 (0.92, 1.25) | 0.39 | 0.97 (0.86, 1.10) | 0.65 |
| **Difucosyl-lacto-N-hexaose-X3** | 1.14 (0.98, 1.32) | 0.09 | **1.13 (1.02, 1.26)** | **0.03** |
| Difucosyl-lacto-N-neo-hexaose I | 0.99 (0.85, 1.15) | 0.90 | 1.07 (0.96, 1.20) | 0.20 |
| Difucosyl-para-lacto-N-hexaose I | 1.06 (0.91, 1.23) | 0.44 | 1.09 (0.99, 1.21) | 0.09 |
| Difucosyldisialyllacto-N-hexaose-X1 | 1.11 (0.95, 1.30) | 0.21 | 0.95 (0.84, 1.07) | 0.39 |
| Difucosyldisialyllacto-N-hexaose-X2 | 0.95 (0.82, 1.11) | 0.54 | 1.04 (0.95, 1.14) | 0.37 |
| Difucosylsialyllacto-N-hexaose-X1 | 1.08 (0.91, 1.29) | 0.35 | 0.97 (0.87, 1.09) | 0.65 |
| Difucosylsialyllacto-N-hexaose-X2 | 1.04 (0.89, 1.22) | 0.61 | 0.96 (0.85, 1.09) | 0.52 |
| Disialyllacto-N-Hexaose-X1 | 1.08 (0.95, 1.24) | 0.25 | 1.00 (0.92, 1.09) | 1.00 |
| Disialyllacto-N-Hexaose-X2 | 1.15 (0.99, 1.34) | 0.06 | 1.05 (0.96, 1.15) | 0.26 |
| Disialyllacto-N-Hexaose-X5 | 0.99 (0.85, 1.16) | 0.91 | 1.00 (0.89, 1.11) | 0.97 |
| Disialyllacto-N-tetraose | 1.03 (0.89, 1.19) | 0.70 | 1.04 (0.95, 1.14) | 0.38 |
| **Fucosyl(1-3)-iso-lacto-N-octaose** | 1.07 (0.92, 1.23) | 0.38 | **1.09 (1.00, 1.18)** | **0.05** |
| Fucosyl-para-lacto-N-hexaose I | 1.10 (0.95, 1.27) | 0.20 | 1.10 (0.99, 1.23) | 0.07 |
| Fucosyldiasialyllacto-N-hexaose-X1 | 0.99 (0.84, 1.15) | 0.85 | 1.03 (0.96, 1.12) | 0.41 |
| Fucosyldiasialyllacto-N-hexaose-X2 | 1.08 (0.92, 1.26) | 0.35 | 1.09 (0.98, 1.22) | 0.10 |
| Fucosyldisialyllacto-N-tetraose-X2 | 1.06 (0.92, 1.23) | 0.44 | 1.08 (0.98, 1.19) | 0.11 |
| Fucosyllacto-N-hexaose II | 1.01 (0.86, 1.18) | 0.90 | 1.08 (0.98, 1.18) | 0.12 |
| Fucosyllacto-N-hexaose-X1 | 1.08 (0.93, 1.25) | 0.32 | 0.98 (0.90, 1.07) | 0.69 |
| Fucosyllacto-N-hexaose-X2 | 1.04 (0.88, 1.24) | 0.63 | 0.96 (0.86, 1.07) | 0.42 |
| **Fucosyllacto-N-hexaose-X4** | **1.20 (1.01, 1.42)** | **0.04** | 1.10 (0.98, 1.23) | 0.09 |
| Fucosyllacto-N-octaose-X1 | 1.09 (0.94, 1.28) | 0.26 | 1.01 (0.92, 1.11) | 0.81 |
| Fucosyllacto-N-octaose-X2 | 1.13 (0.97, 1.32) | 0.13 | 1.08 (0.98, 1.18) | 0.11 |
| Fucosyllacto-N-sulfate-X1 | 1.07 (0.95, 1.22) | 0.27 | 1.00 (0.93, 1.08) | 0.93 |
| Fucosylsialyllacto-N-Hexaose-X1 | 1.02 (0.87, 1.19) | 0.83 | 1.07 (0.98, 1.17) | 0.13 |
| Fucosylsialyllacto-N-Hexaose-X2 | 0.98 (0.84, 1.14) | 0.76 | 1.00 (0.93, 1.08) | 0.94 |
| Fucosylsialyllacto-N-Hexaose-X3 | 1.01 (0.86, 1.19) | 0.93 | 0.94 (0.85, 1.05) | 0.27 |
| Fucosylsialyllacto-N-Hexaose-X4 | 1.01 (0.86, 1.19) | 0.86 | 1.05 (0.95, 1.16) | 0.39 |
| Fucosylsialyllacto-N-Hexaose-X5 | 0.96 (0.82, 1.12) | 0.63 | 1.03 (0.94, 1.13) | 0.51 |
| Fucosylsialyllacto-N-Hexaose-X6 | 1.14 (0.98, 1.33) | 0.10 | 1.09 (0.99, 1.21) | 0.09 |
| Fucosylsialyllacto-N-neo-tetraose c | 1.07 (0.92, 1.26) | 0.37 | 1.08 (0.98, 1.20) | 0.12 |
| Fucosylsialyllacto-N-tetraose a | 0.96 (0.83, 1.11) | 0.57 | 1.04 (0.95, 1.14) | 0.37 |
| Fucosylsialyllacto-N-tetraose b | 1.10 (0.93, 1.29) | 0.27 | 1.00 (0.90, 1.11) | 0.98 |
| **Fucosylsialyllacto-N-tetraose-X1** | 1.12 (0.98, 1.29) | 0.11 | **1.12 (1.01, 1.25)** | **0.03** |
| Lacto-N-decaose-X1 | 1.09 (0.95, 1.25) | 0.22 | 1.06 (0.97, 1.16) | 0.21 |
| Lacto-N-difucohexaose I | 1.07 (0.93, 1.23) | 0.34 | 0.99 (0.89, 1.10) | 0.83 |
| Lacto-N-fucopentaose I | 1.06 (0.90, 1.23) | 0.49 | 0.96 (0.87, 1.07) | 0.47 |
| Lacto-N-fucopentaose II | 1.04 (0.89, 1.21) | 0.64 | 1.06 (0.96, 1.18) | 0.26 |
| Lacto-N-fucopentaose III | 1.06 (0.91, 1.24) | 0.45 | 1.11 (0.99, 1.25) | 0.07 |
| Lacto-N-fucopentaose V | 1.03 (0.88, 1.20) | 0.74 | 1.09 (0.97, 1.21) | 0.15 |
| Lacto-N-hexaose | 1.06 (0.92, 1.22) | 0.45 | 1.03 (0.94, 1.12) | 0.55 |
| Lacto-N-neo-difucohexaose I | 1.09 (0.91, 1.32) | 0.35 | 1.01 (0.92, 1.10) | 0.91 |
| Lacto-N-neo-difucohexaose II | 1.01 (0.87, 1.18) | 0.88 | 1.08 (0.98, 1.19) | 0.14 |
| Lacto-N-neo-hexaose | 1.10 (0.94, 1.29) | 0.25 | 1.04 (0.96, 1.12) | 0.40 |
| Lacto-N-neo-octaose | 1.10 (0.97, 1.24) | 0.14 | 1.05 (0.98, 1.12) | 0.20 |
| Lacto-N-tetraose | 1.05 (0.91, 1.22) | 0.50 | 1.06 (0.96, 1.18) | 0.25 |
| Lacto-N-triose II | 1.09 (0.94, 1.26) | 0.25 | 1.08 (0.98, 1.20) | 0.11 |
| Lactose-3'-Sulfate | 1.04 (0.88, 1.22) | 0.67 | 1.01 (0.92, 1.11) | 0.81 |
| Sialyllacto-N-hexaose-X1 | 1.01 (0.87, 1.17) | 0.91 | 1.04 (0.95, 1.13) | 0.40 |
| Sialyllacto-N-hexaose-X2 | 1.10 (0.94, 1.29) | 0.23 | 0.99 (0.90, 1.09) | 0.85 |
| Sialyllacto-N-tetraose a | 1.03 (0.89, 1.19) | 0.69 | 1.04 (0.95, 1.14) | 0.38 |
| Sialyllacto-N-tetraose b | 1.08 (0.93, 1.24) | 0.33 | 1.06 (0.96, 1.17) | 0.28 |
| Sialyllacto-N-tetraose c | 1.10 (0.96, 1.26) | 0.18 | 1.02 (0.93, 1.12) | 0.69 |
| Trifucosyl(1-2,1-2,1-3)-iso-lacto-N-octa | 1.05 (0.89, 1.23) | 0.59 | 0.96 (0.87, 1.07) | 0.46 |
| Trifucosyllacto-N-hexaose I | 1.07 (0.93, 1.23) | 0.34 | 0.99 (0.89, 1.10) | 0.86 |
| Trifucosyllacto-N-hexaose-X1 | 1.10 (0.95, 1.28) | 0.21 | 1.04 (0.95, 1.14) | 0.42 |
| Trifucosyllacto-N-octaose-X1 | 1.13 (0.98, 1.29) | 0.09 | 1.02 (0.92, 1.13) | 0.71 |
| Trifucosyllacto-N-tetraose-X5 | 1.07 (0.92, 1.24) | 0.37 | 0.99 (0.90, 1.09) | 0.85 |
| a-Heptasaccharide | 1.12 (0.95, 1.32) | 0.18 | 1.08 (0.97, 1.21) | 0.14 |
| a-Pentasaccharide | 1.15 (0.96, 1.37) | 0.12 | 1.09 (0.97, 1.22) | 0.15 |
| **a-Tetrasaccharide** | **1.18 (1.01, 1.37)** | **0.04** | 1.09 (0.98, 1.21) | 0.13 |

Associations determined by modified Poisson regression. Models adjusted for batch, child sex, duration of exclusive breastfeeding (weeks) and gestation age (weeks). Bold denotes statistically significant at nominal level (p<0.05). *Denotes statistically significant after correction for multiple testing (FDR<0.010). URTI: upper respiratory infections; HMO: Human milk oligosaccharides. RR: weighted Risk Ratio determined using weighted secretor status; CI: Confidence Interval

**Table S14**: Adjusted associations between human milk oligosaccharides measured at 6 weeks of lactation with otitis media (OM) in the first or second year of life, stratified by secretor status in the Ulm SPATZ Health Study.

|  | **OM at 1 year** | | | | **OM at 2 years** | | | |
| --- | --- | --- | --- | --- | --- | --- | --- | --- |
|  | **Secretor milk** | | **Non-secretor milk** | | **Secretor milk** | | **Non-secretor milk** | |
| **HMO Structure** | RR (95% CI) | p | RR (95% CI) | p | RR (95% CI) | p | RR (95% CI) | p |
| 2,3 Difucosyllactose | 0.93 (0.57, 1.53) | 0.79 | . (., .) | . | 0.77 (0.58, 1.04) | 0.09 | . (., .) | . |
| **2-Fucosyllactose** | **0.26 (0.10, 0.69)** | **0.01** | . (., .) | . | 0.75 (0.44, 1.27) | 0.28 | . (., .) | . |
| **3'-Sialyl-3-fucosyllactose** | 1.17 (0.67, 2.03) | 0.58 | 0.52 (0.25, 1.08) | 0.08 | 0.90 (0.59, 1.36) | 0.61 | **0.60 (0.39, 0.92)** | **0.02** |
| **3-Fucosyllactose** | 1.01 (0.51, 2.00) | 0.98 | 0.72 (0.36, 1.45) | 0.35 | 0.74 (0.50, 1.11) | 0.15 | **0.54 (0.35, 0.83)** | **0.01** |
| **3-Galactosyllactose** | 0.75 (0.46, 1.21) | 0.24 | 1.29 (0.61, 2.74) | 0.5 | 0.95 (0.68, 1.32) | 0.75 | **1.83 (1.14, 2.94)** | **0.01** |
| 3-sialyllactose | 0.97 (0.52, 1.80) | 0.92 | 0.61 (0.25, 1.45) | 0.26 | 1.04 (0.74, 1.47) | 0.81 | 0.79 (0.50, 1.24) | 0.31 |
| **6'-Sialyl-N-acetyllactosamine** | **0.57 (0.33, 0.97)** | **0.04** | 0.87 (0.37, 2.02) | 0.74 | 0.81 (0.55, 1.18) | 0.27 | 1.10 (0.59, 2.04) | 0.77 |
| **6-Galactosyllactose** | **0.55 (0.31, 0.99)** | **0.05** | 1.90 (1.02, 3.55) | 0.04 | 0.90 (0.62, 1.31) | 0.57 | 2.07 (1.34, 3.22) | **<0.001** |
| 6-sialyllactose | 0.73 (0.42, 1.28) | 0.27 | 0.98 (0.47, 2.02) | 0.95 | 0.80 (0.56, 1.13) | 0.21 | 0.95 (0.55, 1.62) | 0.84 |
| Difucosyl-lacto-N-hexaose I | 0.61 (0.27, 1.35) | 0.22 | . (., .) | . | 1.10 (0.67, 1.83) | 0.7 | . (., .) | . |
| **Difucosyl-lacto-N-hexaose II** | 1.21 (0.52, 2.81) | 0.66 | 1.28 (0.53, 3.05) | 0.58 | 0.91 (0.57, 1.46) | 0.69 | **0.52 (0.37, 0.74)** | **<0.001*** |
| Difucosyl-lacto-N-hexaose-X1 | 1.73 (0.68, 4.44) | 0.25 | . (., .) | . | 1.37 (0.82, 2.30) | 0.22 | . (., .) | . |
| Difucosyl-lacto-N-hexaose-X2 | 0.88 (0.48, 1.63) | 0.69 | . (., .) | . | 0.74 (0.49, 1.11) | 0.14 | . (., .) | . |
| Difucosyl-lacto-N-hexaose-X3 | 1.05 (0.68, 1.62) | 0.83 | 0.61 (0.32, 1.17) | 0.14 | 0.83 (0.61, 1.13) | 0.24 | 1.02 (0.62, 1.66) | 0.95 |
| Difucosyl-lacto-N-neo-hexaose I | 0.92 (0.61, 1.38) | 0.67 | 1.20 (0.45, 3.17) | 0.72 | 0.94 (0.71, 1.25) | 0.69 | 0.79 (0.45, 1.40) | 0.43 |
| Difucosyl-para-lacto-N-hexaose I | 1.86 (0.78, 4.41) | 0.16 | 1.09 (0.48, 2.45) | 0.84 | 0.91 (0.58, 1.41) | 0.66 | 0.72 (0.47, 1.11) | 0.14 |
| **Difucosyldisialyllacto-N-hexaose-X1** | **0.35 (0.15, 0.79)** | **0.01** | . (., .) | . | 0.75 (0.39, 1.46) | 0.4 | . (., .) | . |
| **Difucosyldisialyllacto-N-hexaose-X2** | 1.23 (0.61, 2.48) | 0.56 | 0.61 (0.36, 1.05) | 0.07 | 1.00 (0.65, 1.54) | 0.99 | **0.47 (0.35, 0.64)** | **<0.001*** |
| Difucosylsialyllacto-N-hexaose-X1 | 0.44 (0.19, 1.00) | 0.05 | . (., .) | . | 0.70 (0.35, 1.37) | 0.29 | . (., .) | . |
| Difucosylsialyllacto-N-hexaose-X2 | 0.73 (0.43, 1.24) | 0.24 | . (., .) | . | 0.77 (0.52, 1.15) | 0.2 | . (., .) | . |
| Disialyllacto-N-Hexaose-X1 | 0.81 (0.36, 1.82) | 0.62 | 1.09 (0.43, 2.72) | 0.86 | 0.93 (0.60, 1.42) | 0.72 | 1.16 (0.65, 2.09) | 0.62 |
| Disialyllacto-N-Hexaose-X2 | 1.36 (0.58, 3.21) | 0.48 | 0.75 (0.39, 1.43) | 0.38 | 0.95 (0.61, 1.49) | 0.84 | 1.03 (0.63, 1.70) | 0.9 |
| Disialyllacto-N-Hexaose-X5 | 0.55 (0.29, 1.04) | 0.07 | 1.10 (0.43, 2.86) | 0.84 | 0.70 (0.47, 1.04) | 0.08 | 1.15 (0.65, 2.02) | 0.63 |
| Disialyllacto-N-tetraose | 1.22 (0.58, 2.55) | 0.6 | 0.50 (0.18, 1.36) | 0.17 | 1.03 (0.73, 1.45) | 0.88 | 0.89 (0.50, 1.61) | 0.71 |
| Fucosyl(1-3)-iso-lacto-N-octaose | 1.34 (0.63, 2.88) | 0.44 | 1.48 (0.76, 2.90) | 0.25 | 1.20 (0.80, 1.78) | 0.38 | 1.71 (0.89, 3.31) | 0.11 |
| **Fucosyl-para-lacto-N-hexaose I** | 1.67 (0.73, 3.80) | 0.22 | 1.05 (0.74, 1.50) | 0.78 | 1.11 (0.78, 1.60) | 0.56 | **1.77 (1.17, 2.68)** | **0.01** |
| **Fucosyldiasialyllacto-N-hexaose-X1** | 0.83 (0.39, 1.75) | 0.62 | **0.54 (0.30, 0.97)** | **0.04** | 1.06 (0.72, 1.57) | 0.76 | 0.77 (0.48, 1.26) | 0.3 |
| Fucosyldiasialyllacto-N-hexaose-X2 | 1.09 (0.45, 2.62) | 0.85 | 0.46 (0.19, 1.15) | 0.1 | 0.94 (0.63, 1.40) | 0.76 | 1.09 (0.64, 1.87) | 0.74 |
| **Fucosyldisialyllacto-N-tetraose-X2** | 1.40 (0.66, 2.97) | 0.38 | 0.60 (0.28, 1.29) | 0.19 | 0.92 (0.60, 1.39) | 0.68 | **0.62 (0.44, 0.89)** | **0.01** |
| Fucosyllacto-N-hexaose II | 1.09 (0.50, 2.37) | 0.82 | 1.69 (0.51, 5.61) | 0.39 | 1.11 (0.73, 1.71) | 0.62 | 1.37 (0.64, 2.93) | 0.41 |
| Fucosyllacto-N-hexaose-X1 | 1.17 (0.51, 2.70) | 0.71 | . (., .) | . | 0.94 (0.58, 1.51) | 0.79 | . (., .) | . |
| Fucosyllacto-N-hexaose-X2 | 0.62 (0.26, 1.49) | 0.28 | . (., .) | . | 0.91 (0.55, 1.51) | 0.72 | . (., .) | . |
| Fucosyllacto-N-hexaose-X4 | 1.08 (0.65, 1.80) | 0.75 | 1.63 (0.94, 2.83) | 0.08 | 0.75 (0.53, 1.06) | 0.1 | 1.04 (0.58, 1.89) | 0.89 |
| Fucosyllacto-N-octaose-X1 | 0.84 (0.44, 1.59) | 0.58 | . (., .) | . | 0.96 (0.62, 1.46) | 0.84 | . (., .) | . |
| Fucosyllacto-N-octaose-X2 | 1.18 (0.70, 1.99) | 0.54 | 1.83 (0.87, 3.88) | 0.11 | 0.89 (0.68, 1.17) | 0.39 | 1.39 (0.75, 2.57) | 0.29 |
| Fucosyllacto-N-sulfate-X1 | 0.76 (0.33, 1.76) | 0.52 | . (., .) | . | 0.92 (0.55, 1.56) | 0.76 | . (., .) | . |
| Fucosylsialyllacto-N-Hexaose-X1 | 0.98 (0.46, 2.09) | 0.97 | 1.06 (0.38, 2.91) | 0.92 | 1.00 (0.67, 1.50) | 0.99 | 1.02 (0.59, 1.75) | 0.95 |
| Fucosylsialyllacto-N-Hexaose-X2 | 0.94 (0.49, 1.77) | 0.84 | 1.29 (0.76, 2.20) | 0.35 | 1.25 (0.85, 1.83) | 0.25 | 1.23 (0.76, 1.98) | 0.4 |
| Fucosylsialyllacto-N-Hexaose-X3 | 0.64 (0.29, 1.41) | 0.27 | 0.44 (0.03, 5.88) | 0.54 | 0.97 (0.57, 1.62) | 0.9 | 0.95 (0.30, 2.98) | 0.93 |
| Fucosylsialyllacto-N-Hexaose-X4 | 0.78 (0.48, 1.28) | 0.33 | 1.06 (0.51, 2.18) | 0.88 | 0.75 (0.53, 1.04) | 0.08 | 1.13 (0.70, 1.81) | 0.62 |
| Fucosylsialyllacto-N-Hexaose-X5 | 0.96 (0.42, 2.20) | 0.93 | 1.95 (0.65, 5.87) | 0.24 | 0.75 (0.48, 1.16) | 0.2 | 0.57 (0.35, 0.93) | 0.02 |
| Fucosylsialyllacto-N-Hexaose-X6 | 0.94 (0.56, 1.57) | 0.81 | 0.54 (0.26, 1.12) | 0.1 | 0.72 (0.47, 1.10) | 0.13 | 1.12 (0.67, 1.86) | 0.66 |
| Fucosylsialyllacto-N-neo-tetraose c | 0.93 (0.57, 1.52) | 0.77 | 0.79 (0.38, 1.64) | 0.53 | 0.69 (0.49, 0.96) | 0.03 | 0.72 (0.45, 1.16) | 0.17 |
| **Fucosylsialyllacto-N-tetraose a** | 1.46 (0.66, 3.23) | 0.36 | 1.08 (0.55, 2.13) | 0.82 | 0.99 (0.64, 1.54) | 0.97 | **0.61 (0.43, 0.89)** | **0.01** |
| Fucosylsialyllacto-N-tetraose b | 1.47 (0.59, 3.67) | 0.41 | . (., .) | . | 1.08 (0.58, 1.98) | 0.81 | . (., .) | . |
| Fucosylsialyllacto-N-tetraose-X1 | 1.11 (0.50, 2.47) | 0.79 | 0.75 (0.38, 1.49) | 0.41 | 0.89 (0.59, 1.35) | 0.59 | 0.80 (0.51, 1.26) | 0.34 |
| Lacto-N-decaose-X1 | 1.05 (0.49, 2.26) | 0.89 | 1.53 (0.65, 3.57) | 0.33 | 1.04 (0.71, 1.52) | 0.86 | 1.55 (1.05, 2.30) | 0.03 |
| Lacto-N-difucohexaose I | 0.82 (0.47, 1.44) | 0.49 | . (., .) | . | 0.76 (0.49, 1.18) | 0.23 | . (., .) | . |
| Lacto-N-fucopentaose I | 1.51 (0.75, 3.04) | 0.25 | . (., .) | . | 1.31 (0.86, 2.01) | 0.21 | . (., .) | . |
| **Lacto-N-fucopentaose II** | 1.84 (0.64, 5.29) | 0.26 | 1.26 (0.46, 3.48) | 0.65 | 0.88 (0.54, 1.43) | 0.6 | **0.62 (0.44, 0.88)** | **0.01** |
| Lacto-N-fucopentaose III | 1.00 (0.66, 1.53) | 0.98 | 0.78 (0.40, 1.53) | 0.47 | 0.90 (0.68, 1.20) | 0.47 | 1.12 (0.64, 1.98) | 0.69 |
| Lacto-N-fucopentaose V | 2.39 (0.69, 8.29) | 0.17 | 0.98 (0.31, 3.09) | 0.98 | 0.98 (0.60, 1.61) | 0.95 | 0.73 (0.35, 1.51) | 0.39 |
| Lacto-N-hexaose | 1.02 (0.52, 2.01) | 0.94 | 1.53 (0.72, 3.25) | 0.27 | 1.09 (0.74, 1.62) | 0.66 | 1.26 (0.79, 2.02) | 0.33 |
| **Lacto-N-neo-difucohexaose I** | **0.48 (0.24, 0.97)** | **0.04** | . (., .) | . | 0.56 (0.30, 1.03) | 0.06 | . (., .) | . |
| **Lacto-N-neo-difucohexaose II** | 1.17 (0.65, 2.08) | 0.6 | 0.64 (0.27, 1.52) | 0.31 | 0.82 (0.55, 1.21) | 0.32 | **0.56 (0.36, 0.86)** | **0.01** |
| Lacto-N-neo-hexaose | 0.82 (0.44, 1.53) | 0.53 | 1.70 (0.62, 4.62) | 0.3 | 0.73 (0.48, 1.11) | 0.15 | 1.18 (0.55, 2.54) | 0.68 |
| Lacto-N-neo-octaose | 1.09 (0.52, 2.30) | 0.82 | 1.50 (0.62, 3.65) | 0.37 | 0.96 (0.63, 1.45) | 0.85 | 1.56 (0.96, 2.53) | 0.07 |
| Lacto-N-tetraose | 1.81 (0.81, 4.04) | 0.15 | 1.15 (0.67, 1.95) | 0.62 | 1.17 (0.82, 1.68) | 0.38 | 1.57 (0.92, 2.68) | 0.1 |
| Lacto-N-triose II | 1.37 (0.76, 2.49) | 0.29 | 1.43 (0.88, 2.32) | 0.15 | 1.02 (0.67, 1.54) | 0.93 | 1.57 (0.73, 3.38) | 0.25 |
| **Lactose-3'-Sulfate** | 0.74 (0.40, 1.38) | 0.35 | **0.26 (0.12, 0.57)** | **<0.001** | 1.06 (0.72, 1.56) | 0.78 | 0.65 (0.36, 1.17) | 0.15 |
| Sialyllacto-N-hexaose-X1 | 1.03 (0.50, 2.14) | 0.93 | 1.60 (0.59, 4.37) | 0.36 | 0.99 (0.66, 1.48) | 0.96 | 1.44 (0.76, 2.71) | 0.26 |
| Sialyllacto-N-hexaose-X2 | 0.70 (0.34, 1.42) | 0.32 | 2.39 (0.87, 6.53) | 0.09 | 0.65 (0.40, 1.05) | 0.08 | 1.40 (0.65, 3.04) | 0.39 |
| Sialyllacto-N-tetraose a | 1.19 (0.51, 2.79) | 0.69 | 1.17 (0.66, 2.08) | 0.6 | 1.20 (0.77, 1.87) | 0.42 | 1.31 (0.76, 2.27) | 0.33 |
| Sialyllacto-N-tetraose b | 1.52 (0.75, 3.08) | 0.25 | 0.80 (0.38, 1.66) | 0.54 | 1.04 (0.74, 1.47) | 0.82 | 1.15 (0.73, 1.82) | 0.54 |
| Sialyllacto-N-tetraose c | 0.73 (0.42, 1.26) | 0.26 | 0.91 (0.39, 2.09) | 0.82 | 0.67 (0.41, 1.12) | 0.13 | 1.24 (0.69, 2.24) | 0.47 |
| Trifucosyl(1-2,1-2,1-3)-iso-lacto-N-octa | 0.90 (0.40, 2.05) | 0.81 | . (., .) | . | 1.13 (0.68, 1.88) | 0.65 | . (., .) | . |
| Trifucosyllacto-N-hexaose I | 0.89 (0.51, 1.56) | 0.69 | . (., .) | . | 0.96 (0.61, 1.52) | 0.87 | . (., .) | . |
| Trifucosyllacto-N-hexaose-X1 | 1.21 (0.66, 2.21) | 0.54 | . (., .) | . | 0.90 (0.57, 1.42) | 0.64 | . (., .) | . |
| Trifucosyllacto-N-octaose-X1 | 1.28 (0.65, 2.51) | 0.47 | . (., .) | . | 0.97 (0.62, 1.51) | 0.89 | . (., .) | . |
| Trifucosyllacto-N-tetraose-X5 | 0.88 (0.52, 1.51) | 0.65 | . (., .) | . | 0.83 (0.56, 1.23) | 0.35 | . (., .) | . |
| a-Heptasaccharide | 1.01 (0.59, 1.74) | 0.96 | . (., .) | . | 0.96 (0.68, 1.37) | 0.83 | . (., .) | . |
| a-Pentasaccharide | 1.13 (0.64, 2.00) | 0.66 | . (., .) | . | 1.02 (0.72, 1.46) | 0.9 | . (., .) | . |
| a-Tetrasaccharide | 1.04 (0.56, 1.93) | 0.91 | . (., .) | . | 0.94 (0.65, 1.36) | 0.75 | . (., .) | . |

Associations determined by modified Poisson regression. Models adjusted for batch, child sex, duration of exclusive breastfeeding (weeks) and gestation age (weeks). Bold denotes statistically significant at nominal level (p<0.05). *Denotes statistically significant after correction for multiple testing (FDR<0.010). OM: Otitis media; HMO: Human milk oligosaccharides.

**Table S15**: Adjusted associations between human milk oligosaccharides measured at 6 weeks of lactation with lower respiratory tract infections (LRTI) in the first or second year of life stratified by secretor status in the Ulm SPATZ Health Study.

|  | **LRTI at 1 year** | | | | **LRTI at 2 years** | | | |
| --- | --- | --- | --- | --- | --- | --- | --- | --- |
|  | Secretor milk | | Non-secretor milk | | Secretor milk | | Non-secretor milk | |
| **HMO Structure** | RR (95% CI) | p | RR (95% CI) | p | RR (95% CI) | p | RR (95% CI) | p |
| **2,3 Difucosyllactose** | 0.75 (0.47, 1.20) | 0.23 | . (., .) | . | 0.65 (0.51, 0.84) | **0.001** | . (., .) | . |
| 2-Fucosyllactose | 0.42 (0.16, 1.10) | 0.08 | . (., .) | . | 0.82 (0.52, 1.28) | 0.38 | . (., .) | . |
| 3'-Sialyl-3-fucosyllactose | 1.24 (0.66, 2.36) | 0.5 | 0.85 (0.56, 1.29) | 0.44 | 0.88 (0.61, 1.27) | 0.5 | 1.10 (0.79, 1.53) | 0.58 |
| 3-Fucosyllactose | 0.76 (0.41, 1.44) | 0.41 | 0.75 (0.50, 1.13) | 0.17 | 0.70 (0.48, 1.03) | 0.07 | 0.92 (0.63, 1.34) | 0.66 |
| 3-Galactosyllactose | 0.97 (0.63, 1.51) | 0.91 | 0.99 (0.72, 1.35) | 0.93 | 0.96 (0.75, 1.22) | 0.74 | 0.97 (0.76, 1.23) | 0.78 |
| 3-sialyllactose | 1.41 (0.80, 2.49) | 0.24 | 1.04 (0.72, 1.53) | 0.82 | 1.17 (0.89, 1.53) | 0.26 | 1.17 (0.88, 1.54) | 0.28 |
| 6'-Sialyl-N-acetyllactosamine | 0.87 (0.54, 1.38) | 0.55 | 1.13 (0.78, 1.63) | 0.51 | 0.97 (0.73, 1.30) | 0.85 | 1.09 (0.83, 1.43) | 0.55 |
| **6-Galactosyllactose** | 0.66 (0.42, 1.01) | 0.06 | 0.96 (0.68, 1.35) | 0.82 | **0.65 (0.50, 0.85)** | **0.002** | 0.97 (0.72, 1.32) | 0.85 |
| 6-sialyllactose | 0.78 (0.48, 1.26) | 0.31 | 1.02 (0.70, 1.48) | 0.91 | 0.91 (0.68, 1.21) | 0.5 | 1.11 (0.82, 1.50) | 0.51 |
| Difucosyl-lacto-N-hexaose I | 0.81 (0.39, 1.69) | 0.58 | . (., .) | . | 1.14 (0.73, 1.77) | 0.57 | . (., .) | . |
| Difucosyl-lacto-N-hexaose II | 1.08 (0.45, 2.59) | 0.87 | 0.85 (0.52, 1.38) | 0.51 | 0.75 (0.48, 1.17) | 0.2 | 0.85 (0.58, 1.26) | 0.42 |
| Difucosyl-lacto-N-hexaose-X1 | 1.12 (0.55, 2.30) | 0.75 | . (., .) | . | 1.14 (0.76, 1.72) | 0.51 | . (., .) | . |
| Difucosyl-lacto-N-hexaose-X2 | 0.93 (0.50, 1.70) | 0.8 | . (., .) | . | 0.76 (0.53, 1.10) | 0.15 | . (., .) | . |
| **Difucosyl-lacto-N-hexaose-X3** | 0.69 (0.46, 1.04) | 0.08 | 0.82 (0.57, 1.16) | 0.26 | **0.73 (0.56, 0.94)** | **0.02** | 1.00 (0.77, 1.30) | 0.99 |
| **Difucosyl-lacto-N-neo-hexaose I** | 0.67 (0.42, 1.06) | 0.09 | 0.90 (0.58, 1.41) | 0.65 | **0.70 (0.55, 0.89)** | **0.004** | 0.85 (0.59, 1.21) | 0.36 |
| Difucosyl-para-lacto-N-hexaose I | 1.41 (0.71, 2.81) | 0.32 | 0.80 (0.57, 1.13) | 0.2 | 0.89 (0.59, 1.35) | 0.58 | 0.93 (0.68, 1.27) | 0.66 |
| Difucosyldisialyllacto-N-hexaose-X1 | 0.88 (0.39, 2.01) | 0.76 | . (., .) | . | 0.98 (0.61, 1.58) | 0.94 | 1.31 (0.17, 9.85) | 0.79 |
| Difucosyldisialyllacto-N-hexaose-X2 | 1.21 (0.52, 2.83) | 0.66 | 1.09 (0.67, 1.77) | 0.72 | 0.76 (0.50, 1.14) | 0.18 | 1.07 (0.72, 1.57) | 0.75 |
| Difucosylsialyllacto-N-hexaose-X1 | 0.48 (0.20, 1.19) | 0.11 | . (., .) | . | 0.72 (0.41, 1.26) | 0.25 | . (., .) | . |
| Difucosylsialyllacto-N-hexaose-X2 | 0.63 (0.34, 1.19) | 0.16 | . (., .) | . | 0.63 (0.43, 0.91) | 0.01 | . (., .) | . |
| **Disialyllacto-N-Hexaose-X1** | 1.33 (0.75, 2.36) | 0.32 | **1.56 (1.11, 2.19)** | **0.01** | 1.13 (0.81, 1.57) | 0.46 | 1.18 (0.88, 1.58) | 0.26 |
| Disialyllacto-N-Hexaose-X2 | 1.43 (0.75, 2.76) | 0.28 | 1.05 (0.72, 1.54) | 0.8 | 1.04 (0.73, 1.49) | 0.82 | 1.06 (0.79, 1.41) | 0.7 |
| Disialyllacto-N-Hexaose-X5 | 0.55 (0.29, 1.04) | 0.06 | 1.26 (0.90, 1.77) | 0.18 | 0.73 (0.51, 1.04) | 0.09 | 1.13 (0.88, 1.47) | 0.34 |
| **Disialyllacto-N-tetraose** | **1.60 (1.02, 2.51)** | **0.04** | 1.04 (0.70, 1.54) | 0.85 | 1.09 (0.83, 1.43) | 0.55 | 1.06 (0.83, 1.37) | 0.64 |
| **Fucosyl(1-3)-iso-lacto-N-octaose** | **2.61 (1.60, 4.28)** | **<0.001*** | 1.00 (0.67, 1.48) | 0.99 | **1.42 (1.11, 1.81)** | **0.005** | 0.93 (0.71, 1.23) | 0.61 |
| **Fucosyl-para-lacto-N-hexaose I** | **1.69 (1.10, 2.59)** | **0.02** | 1.02 (0.73, 1.43) | 0.91 | 1.22 (0.93, 1.60) | 0.14 | 1.02 (0.79, 1.32) | 0.86 |
| Fucosyldiasialyllacto-N-hexaose-X1 | 1.31 (0.73, 2.38) | 0.37 | 1.24 (0.90, 1.72) | 0.19 | 1.02 (0.76, 1.37) | 0.88 | 1.06 (0.79, 1.42) | 0.71 |
| Fucosyldiasialyllacto-N-hexaose-X2 | 1.20 (0.68, 2.13) | 0.53 | 0.99 (0.70, 1.39) | 0.93 | 0.95 (0.72, 1.26) | 0.72 | 1.06 (0.83, 1.36) | 0.63 |
| Fucosyldisialyllacto-N-tetraose-X2 | 1.57 (0.78, 3.18) | 0.21 | 0.93 (0.63, 1.38) | 0.73 | 0.92 (0.62, 1.38) | 0.7 | 1.07 (0.79, 1.45) | 0.67 |
| **Fucosyllacto-N-hexaose II** | **1.79 (1.04, 3.09)** | **0.04** | 1.09 (0.70, 1.71) | 0.7 | 1.17 (0.88, 1.57) | 0.28 | 0.88 (0.63, 1.23) | 0.46 |
| Fucosyllacto-N-hexaose-X1 | 1.45 (0.80, 2.62) | 0.22 | . (., .) | . | 1.22 (0.89, 1.68) | 0.22 | . (., .) | . |
| Fucosyllacto-N-hexaose-X2 | 1.02 (0.50, 2.06) | 0.96 | . (., .) | . | 1.11 (0.76, 1.61) | 0.59 | . (., .) | . |
| **Fucosyllacto-N-hexaose-X4** | 0.80 (0.44, 1.44) | 0.46 | 1.21 (0.93, 1.58) | 0.15 | **0.70 (0.51, 0.97)** | **0.03** | 1.06 (0.84, 1.35) | 0.62 |
| Fucosyllacto-N-octaose-X1 | 1.25 (0.73, 2.13) | 0.41 | . (., .) | . | 1.22 (0.90, 1.65) | 0.21 | . (., .) | . |
| Fucosyllacto-N-octaose-X2 | 1.43 (0.85, 2.40) | 0.18 | 1.18 (0.83, 1.69) | 0.36 | 0.98 (0.71, 1.37) | 0.93 | 1.01 (0.79, 1.30) | 0.92 |
| Fucosyllacto-N-sulfate-X1 | 1.04 (0.47, 2.33) | 0.91 | . (., .) | . | 1.02 (0.68, 1.53) | 0.94 | . (., .) | . |
| **Fucosylsialyllacto-N-Hexaose-X1** | **1.73 (1.06, 2.82)** | **0.03** | 0.94 (0.60, 1.48) | 0.8 | 1.15 (0.88, 1.52) | 0.3 | 0.89 (0.61, 1.29) | 0.53 |
| Fucosylsialyllacto-N-Hexaose-X2 | 1.15 (0.66, 2.00) | 0.61 | 1.12 (0.79, 1.58) | 0.52 | 1.05 (0.77, 1.45) | 0.75 | 0.90 (0.69, 1.19) | 0.47 |
| **Fucosylsialyllacto-N-Hexaose-X3** | 0.96 (0.51, 1.82) | 0.9 | **0.29 (0.09, 0.91)** | **0.03** | 1.16 (0.77, 1.76) | 0.48 | 0.55 (0.22, 1.37) | 0.2 |
| **Fucosylsialyllacto-N-Hexaose-X4** | **0.54 (0.33, 0.87)** | **0.01** | 1.09 (0.76, 1.55) | 0.65 | 0.71 (0.52, 0.97) | 0.03 | 1.07 (0.83, 1.38) | 0.61 |
| Fucosylsialyllacto-N-Hexaose-X5 | 1.15 (0.55, 2.41) | 0.71 | 0.97 (0.58, 1.62) | 0.89 | 0.80 (0.53, 1.21) | 0.29 | 0.95 (0.64, 1.42) | 0.81 |
| **Fucosylsialyllacto-N-Hexaose-X6** | 0.62 (0.38, 1.03) | 0.07 | 1.06 (0.75, 1.51) | 0.74 | **0.68 (0.50, 0.93)** | **0.01** | 1.14 (0.90, 1.43) | 0.28 |
| **Fucosylsialyllacto-N-neo-tetraose c** | 0.75 (0.42, 1.33) | 0.32 | 1.05 (0.68, 1.62) | 0.83 | **0.70 (0.49, 0.98)** | **0.04** | 1.13 (0.83, 1.54) | 0.43 |
| Fucosylsialyllacto-N-tetraose a | 1.46 (0.68, 3.14) | 0.34 | 0.95 (0.61, 1.47) | 0.81 | 0.89 (0.57, 1.39) | 0.61 | 0.94 (0.68, 1.29) | 0.69 |
| Fucosylsialyllacto-N-tetraose b | 1.75 (0.87, 3.50) | 0.11 | . (., .) | . | 1.20 (0.75, 1.89) | 0.45 | . (., .) | . |
| Fucosylsialyllacto-N-tetraose-X1 | 1.08 (0.54, 2.17) | 0.82 | 0.87 (0.60, 1.26) | 0.47 | 0.87 (0.61, 1.25) | 0.46 | 1.01 (0.76, 1.33) | 0.97 |
| **Lacto-N-decaose-X1** | **1.70 (1.03, 2.82)** | **0.04** | 1.22 (0.90, 1.67) | 0.21 | 1.25 (0.94, 1.66) | 0.13 | 1.00 (0.78, 1.28) | 0.99 |
| Lacto-N-difucohexaose I | 0.79 (0.44, 1.43) | 0.44 | . (., .) | . | 0.73 (0.50, 1.05) | 0.09 | . (., .) | . |
| Lacto-N-fucopentaose I | 1.70 (0.96, 3.02) | 0.07 | . (., .) | . | 1.39 (0.99, 1.95) | 0.06 | . (., .) | . |
| Lacto-N-fucopentaose II | 2.13 (0.84, 5.35) | 0.11 | 0.76 (0.52, 1.10) | 0.15 | 0.99 (0.61, 1.61) | 0.95 | 0.90 (0.65, 1.25) | 0.52 |
| **Lacto-N-fucopentaose III** | 0.75 (0.50, 1.12) | 0.15 | 0.76 (0.52, 1.10) | 0.15 | **0.75 (0.58, 0.96)** | **0.02** | 0.91 (0.68, 1.22) | 0.54 |
| **Lacto-N-fucopentaose V** | **2.87 (1.16, 7.10)** | **0.02** | **0.61 (0.39, 0.96)** | **0.03** | 1.07 (0.68, 1.69) | 0.78 | 0.80 (0.56, 1.14) | 0.21 |
| **Lacto-N-hexaose** | **1.69 (1.07, 2.66)** | **0.02** | 1.24 (0.95, 1.63) | 0.12 | **1.37 (1.02, 1.84)** | **0.04** | 1.00 (0.79, 1.26) | 0.98 |
| Lacto-N-hexaose neo | 0.90 (0.45, 1.81) | 0.77 | 1.35 (0.90, 2.02) | 0.15 | 0.90 (0.63, 1.29) | 0.57 | 1.04 (0.75, 1.45) | 0.8 |
| **Lacto-N-neo-difucohexaose I** | **0.40 (0.18, 0.90)** | **0.03** | . (., .) | . | 0.56 (0.34, 0.91) | 0.02 | . (., .) | . |
| Lacto-N-neo-difucohexaose II | 0.73 (0.38, 1.42) | 0.35 | 0.72 (0.46, 1.14) | 0.16 | 0.66 (0.45, 0.97) | 0.03 | 0.90 (0.60, 1.35) | 0.61 |
| Lacto-N-neo-octaose | 1.53 (0.85, 2.76) | 0.16 | 1.22 (0.84, 1.75) | 0.29 | 1.22 (0.87, 1.70) | 0.24 | 0.99 (0.73, 1.35) | 0.97 |
| **Lacto-N-tetraose** | **2.31 (1.57, 3.38)** | **<0.001*** | 0.88 (0.59, 1.32) | 0.54 | **1.39 (1.06, 1.82)** | **0.02** | 0.90 (0.68, 1.19) | 0.47 |
| **Lacto-N-triose II** | **1.67 (1.14, 2.46)** | **0.01** | 1.12 (0.74, 1.70) | 0.6 | 1.12 (0.84, 1.49) | 0.45 | 1.03 (0.76, 1.39) | 0.86 |
| Lactose-3'-Sulfate | 1.43 (0.87, 2.34) | 0.16 | 0.72 (0.48, 1.06) | 0.1 | 1.02 (0.75, 1.38) | 0.9 | 0.98 (0.73, 1.32) | 0.91 |
| Sialyllacto-N-hexaose-X1 | 1.46 (0.84, 2.52) | 0.18 | 1.17 (0.78, 1.77) | 0.45 | 1.12 (0.84, 1.50) | 0.44 | 1.00 (0.76, 1.33) | 0.98 |
| Sialyllacto-N-hexaose-X2 | 0.67 (0.25, 1.76) | 0.41 | 1.49 (0.97, 2.29) | 0.07 | 0.82 (0.54, 1.26) | 0.37 | 1.09 (0.78, 1.50) | 0.62 |
| **Sialyllacto-N-tetraose a** | **1.69 (1.05, 2.72)** | **0.03** | 1.07 (0.73, 1.55) | 0.73 | 1.38 (1.04, 1.83) | 0.02 | 0.95 (0.72, 1.26) | 0.73 |
| **Sialyllacto-N-tetraose b** | **2.17 (1.38, 3.40)** | **<0.001** | 0.84 (0.60, 1.17) | 0.29 | 1.24 (0.92, 1.68) | 0.16 | 0.98 (0.76, 1.26) | 0.86 |
| Sialyllacto-N-tetraose c | 0.73 (0.39, 1.36) | 0.32 | 1.20 (0.84, 1.72) | 0.32 | 0.80 (0.56, 1.13) | 0.2 | 1.15 (0.91, 1.46) | 0.23 |
| Trifucosyl(1-2,1-2,1-3)-iso-lacto-N-octa | 1.37 (0.72, 2.60) | 0.34 | . (., .) | . | 1.42 (0.96, 2.11) | 0.08 | . (., .) | . |
| Trifucosyllacto-N-hexaose I | 0.83 (0.40, 1.72) | 0.62 | . (., .) | . | 0.70 (0.46, 1.06) | 0.09 | . (., .) | . |
| **Trifucosyllacto-N-hexaose-X1** | 0.72 (0.38, 1.34) | 0.29 | . (., .) | . | **0.65 (0.45, 0.93)** | **0.02** | . (., .) | . |
| Trifucosyllacto-N-octaose-X1 | 1.68 (0.70, 4.05) | 0.25 | . (., .) | . | 1.13 (0.72, 1.76) | 0.6 | . (., .) | . |
| Trifucosyllacto-N-tetraose-X5 | 0.99 (0.57, 1.71) | 0.97 | . (., .) | . | 0.87 (0.62, 1.21) | 0.4 | . (., .) | . |
| a-Heptasaccharide | 1.04 (0.62, 1.73) | 0.89 | . (., .) | . | 1.05 (0.79, 1.40) | 0.72 | . (., .) | . |
| a-Pentasaccharide | 1.16 (0.71, 1.87) | 0.56 | . (., .) | . | 1.11 (0.84, 1.47) | 0.47 | . (., .) | . |
| a-Tetrasaccharide | 1.24 (0.73, 2.11) | 0.42 | . (., .) | . | 1.16 (0.87, 1.56) | 0.32 | . (., .) | . |

Associations determined by modified Poisson regression. Models adjusted for batch, child sex, duration of exclusive breastfeeding (weeks) and gestation age (weeks). Bold denotes statistically significant at nominal level (p<0.05). *Denotes statistically significant after correction for multiple testing (FDR<0.010). LRTI: Lower respiratory tract infections; HMO: Human milk oligosaccharides.

**Table S16**: Adjusted associations between human milk oligosaccharides measured at 6 weeks of lactation with upper respiratory tract infections (URTI) in the first or second year of life stratified by secretor status in the Ulm SPATZ Health Study

|  | **URTI at 1 year** | | | | **URTI at 2 years** | | | |
| --- | --- | --- | --- | --- | --- | --- | --- | --- |
|  | Secretor milk | | Non-secretor milk | | Secretor milk | | Non-secretor milk | |
| **HMO Structure** | RR (95% CI) | p | RR (95% CI) | p | RR (95% CI) | p | RR (95% CI) | p |
| 2,3 Difucosyllactose | 1.15 (0.93, 1.43) | 0.2 | . (., .) | . | 1.05 (0.88, 1.25) | 0.58 | . (., .) | . |
| 2-Fucosyllactose | 0.90 (0.66, 1.21) | 0.48 | . (., .) | . | 0.84 (0.69, 1.01) | 0.07 | . (., .) | . |
| 3'-Sialyl-3-fucosyllactose | 1.09 (0.86, 1.38) | 0.47 | 1.05 (0.85, 1.29) | 0.64 | 1.12 (0.99, 1.28) | 0.07 | 1.00 (0.86, 1.16) | 0.99 |
| 3-Fucosyllactose | 0.97 (0.74, 1.28) | 0.85 | 0.95 (0.78, 1.14) | 0.56 | 1.08 (0.91, 1.27) | 0.37 | 0.93 (0.82, 1.06) | 0.27 |
| 3-Galactosyllactose | 1.11 (0.95, 1.30) | 0.2 | 1.19 (0.99, 1.44) | 0.07 | 1.09 (0.99, 1.21) | 0.07 | 1.04 (0.94, 1.15) | 0.46 |
| 3-sialyllactose | 1.09 (0.91, 1.30) | 0.37 | 1.01 (0.83, 1.22) | 0.96 | 1.04 (0.93, 1.16) | 0.51 | 1.00 (0.89, 1.14) | 0.94 |
| 6'-Sialyl-N-acetyllactosamine | 1.06 (0.89, 1.26) | 0.5 | 1.01 (0.82, 1.23) | 0.95 | 0.98 (0.89, 1.09) | 0.72 | 1.08 (0.95, 1.24) | 0.25 |
| 6-Galactosyllactose | 1.00 (0.83, 1.20) | 0.98 | 1.03 (0.85, 1.24) | 0.76 | 1.00 (0.89, 1.13) | 0.96 | 1.08 (0.96, 1.21) | 0.19 |
| 6-sialyllactose | 1.01 (0.86, 1.18) | 0.9 | 1.00 (0.79, 1.25) | 0.99 | 0.96 (0.87, 1.05) | 0.35 | 1.08 (0.96, 1.21) | 0.21 |
| Difucosyl-lacto-N-hexaose I | 1.04 (0.80, 1.35) | 0.78 | . (., .) | . | 0.95 (0.81, 1.12) | 0.57 | . (., .) | . |
| Difucosyl-lacto-N-hexaose II | 1.02 (0.76, 1.37) | 0.88 | 0.86 (0.70, 1.06) | 0.17 | 1.13 (0.95, 1.35) | 0.17 | 0.94 (0.82, 1.08) | 0.4 |
| Difucosyl-lacto-N-hexaose-X1 | 1.14 (0.89, 1.47) | 0.3 | . (., .) | . | 0.99 (0.85, 1.15) | 0.86 | . (., .) | . |
| Difucosyl-lacto-N-hexaose-X2 | 1.09 (0.87, 1.37) | 0.44 | . (., .) | . | 0.97 (0.82, 1.16) | 0.76 | . (., .) | . |
| Difucosyl-lacto-N-hexaose-X3 | 1.16 (0.97, 1.39) | 0.09 | 1.10 (0.88, 1.38) | 0.4 | 1.15 (1.01, 1.32) | 0.03 | 1.07 (0.94, 1.21) | 0.31 |
| Difucosyl-lacto-N-neo-hexaose I | 1.03 (0.85, 1.25) | 0.75 | 0.78 (0.57, 1.06) | 0.11 | 1.10 (0.96, 1.27) | 0.18 | 0.96 (0.83, 1.12) | 0.63 |
| Difucosyl-para-lacto-N-hexaose I | 1.13 (0.87, 1.46) | 0.36 | 1.06 (0.86, 1.31) | 0.58 | 1.16 (0.99, 1.37) | 0.07 | 1.02 (0.91, 1.15) | 0.7 |
| Difucosyldisialyllacto-N-hexaose-X1 | 1.15 (0.90, 1.47) | 0.27 | 1.24 (0.35, 4.36) | 0.74 | 0.92 (0.77, 1.09) | 0.33 | 1.32 (0.86, 2.03) | 0.2 |
| Difucosyldisialyllacto-N-hexaose-X2 | 0.96 (0.74, 1.25) | 0.77 | 0.87 (0.72, 1.05) | 0.15 | 1.07 (0.93, 1.24) | 0.34 | 0.95 (0.83, 1.08) | 0.43 |
| Difucosylsialyllacto-N-hexaose-X1 | 1.13 (0.84, 1.51) | 0.43 | . (., .) | . | 0.97 (0.81, 1.15) | 0.71 | . (., .) | . |
| Difucosylsialyllacto-N-hexaose-X2 | 1.05 (0.83, 1.32) | 0.68 | . (., .) | . | 0.95 (0.80, 1.14) | 0.6 | . (., .) | . |
| Disialyllacto-N-Hexaose-X1 | 1.11 (0.92, 1.35) | 0.27 | 0.99 (0.81, 1.21) | 0.9 | 1.00 (0.89, 1.13) | 0.95 | 1.00 (0.88, 1.14) | 0.96 |
| Disialyllacto-N-Hexaose-X2 | 1.16 (0.94, 1.43) | 0.17 | 1.15 (0.91, 1.46) | 0.25 | 1.07 (0.94, 1.23) | 0.32 | 1.04 (0.94, 1.16) | 0.44 |
| Disialyllacto-N-Hexaose-X5 | 0.98 (0.80, 1.21) | 0.85 | 0.98 (0.79, 1.21) | 0.83 | 0.97 (0.84, 1.13) | 0.73 | 1.07 (0.96, 1.19) | 0.24 |
| Disialyllacto-N-tetraose | 1.03 (0.87, 1.22) | 0.74 | 1.01 (0.83, 1.24) | 0.9 | 1.04 (0.93, 1.15) | 0.52 | 1.04 (0.94, 1.15) | 0.46 |
| Fucosyl(1-3)-iso-lacto-N-octaose | 1.10 (0.91, 1.33) | 0.34 | 1.00 (0.85, 1.17) | 0.98 | 1.11 (1.01, 1.23) | 0.04 | 1.03 (0.94, 1.12) | 0.58 |
| Fucosyl-para-lacto-N-hexaose I | 1.11 (0.92, 1.33) | 0.29 | 1.11 (0.93, 1.32) | 0.25 | 1.12 (0.98, 1.28) | 0.11 | 1.09 (0.97, 1.21) | 0.14 |
| Fucosyldiasialyllacto-N-hexaose-X1 | 1.03 (0.84, 1.26) | 0.79 | 0.82 (0.67, 1.01) | 0.06 | 1.04 (0.95, 1.14) | 0.36 | 0.96 (0.85, 1.08) | 0.49 |
| Fucosyldiasialyllacto-N-hexaose-X2 | 1.09 (0.90, 1.34) | 0.38 | 1.01 (0.83, 1.23) | 0.91 | 1.10 (0.96, 1.25) | 0.16 | 1.07 (0.95, 1.20) | 0.27 |
| Fucosyldisialyllacto-N-tetraose-X2 | 1.11 (0.87, 1.42) | 0.42 | 1.04 (0.87, 1.25) | 0.67 | 1.12 (0.97, 1.30) | 0.12 | 1.02 (0.91, 1.15) | 0.69 |
| Fucosyllacto-N-hexaose II | 1.06 (0.84, 1.33) | 0.62 | 0.87 (0.69, 1.08) | 0.2 | 1.12 (1.00, 1.26) | 0.05 | 0.97 (0.87, 1.08) | 0.54 |
| Fucosyllacto-N-hexaose-X1 | 1.10 (0.89, 1.37) | 0.38 | . (., .) | . | 0.99 (0.89, 1.11) | 0.87 | . (., .) | . |
| Fucosyllacto-N-hexaose-X2 | 1.05 (0.80, 1.36) | 0.74 | . (., .) | . | 0.94 (0.81, 1.10) | 0.43 | . (., .) | . |
| Fucosyllacto-N-hexaose-X4 | 1.22 (0.96, 1.56) | 0.1 | 1.12 (0.92, 1.37) | 0.26 | 1.12 (0.96, 1.31) | 0.14 | 1.05 (0.92, 1.20) | 0.47 |
| Fucosyllacto-N-octaose-X1 | 1.11 (0.90, 1.37) | 0.33 | . (., .) | . | 1.03 (0.91, 1.16) | 0.65 | . (., .) | . |
| Fucosyllacto-N-octaose-X2 | 1.14 (0.95, 1.38) | 0.17 | 1.05 (0.83, 1.33) | 0.69 | 1.10 (0.98, 1.24) | 0.12 | 1.01 (0.88, 1.15) | 0.93 |
| Fucosyllacto-N-sulfate-X1 | 1.12 (0.90, 1.38) | 0.32 | . (., .) | . | 1.03 (0.93, 1.14) | 0.55 | . (., .) | . |
| Fucosylsialyllacto-N-Hexaose-X1 | 1.05 (0.83, 1.32) | 0.7 | 0.93 (0.73, 1.19) | 0.56 | 1.11 (1.00, 1.23) | 0.05 | 0.94 (0.82, 1.08) | 0.4 |
| Fucosylsialyllacto-N-Hexaose-X2 | 1.02 (0.83, 1.26) | 0.83 | 0.86 (0.71, 1.04) | 0.12 | 1.01 (0.92, 1.10) | 0.91 | 1.00 (0.91, 1.09) | 0.92 |
| Fucosylsialyllacto-N-Hexaose-X3 | 1.01 (0.78, 1.32) | 0.93 | 0.71 (0.41, 1.25) | 0.24 | 0.91 (0.78, 1.07) | 0.24 | 1.02 (0.79, 1.32) | 0.88 |
| Fucosylsialyllacto-N-Hexaose-X4 | 1.02 (0.84, 1.24) | 0.82 | 0.95 (0.76, 1.19) | 0.66 | 1.04 (0.92, 1.17) | 0.55 | 1.07 (0.95, 1.21) | 0.27 |
| Fucosylsialyllacto-N-Hexaose-X5 | 0.98 (0.75, 1.27) | 0.89 | 0.90 (0.74, 1.09) | 0.29 | 1.05 (0.90, 1.22) | 0.55 | 0.97 (0.87, 1.09) | 0.63 |
| Fucosylsialyllacto-N-Hexaose-X6 | 1.15 (0.94, 1.41) | 0.17 | 1.09 (0.89, 1.34) | 0.41 | 1.10 (0.96, 1.26) | 0.18 | 1.10 (0.96, 1.26) | 0.18 |
| Fucosylsialyllacto-N-neo-tetraose c | 1.09 (0.88, 1.36) | 0.42 | 1.11 (0.87, 1.41) | 0.42 | 1.09 (0.95, 1.24) | 0.23 | 1.11 (0.95, 1.29) | 0.19 |
| Fucosylsialyllacto-N-tetraose a | 0.96 (0.75, 1.23) | 0.77 | 0.92 (0.78, 1.09) | 0.34 | 1.06 (0.90, 1.23) | 0.49 | 1.02 (0.94, 1.10) | 0.69 |
| Fucosylsialyllacto-N-tetraose b | 1.16 (0.87, 1.53) | 0.31 | . (., .) | . | 1.03 (0.84, 1.25) | 0.8 | . (., .) | . |
| Fucosylsialyllacto-N-tetraose-X1 | 1.19 (0.96, 1.47) | 0.12 | 1.11 (0.91, 1.36) | 0.29 | 1.19 (1.02, 1.38) | 0.03 | 1.04 (0.91, 1.17) | 0.58 |
| Lacto-N-decaose-X1 | 1.12 (0.94, 1.34) | 0.21 | 1.00 (0.86, 1.15) | 0.95 | 1.08 (0.96, 1.21) | 0.2 | 1.02 (0.93, 1.11) | 0.71 |
| Lacto-N-difucohexaose I | 1.10 (0.89, 1.36) | 0.4 | . (., .) | . | 1.00 (0.85, 1.18) | 0.98 | . (., .) | . |
| Lacto-N-fucopentaose I | 1.08 (0.84, 1.39) | 0.55 | . (., .) | . | 0.96 (0.81, 1.12) | 0.58 | . (., .) | . |
| Lacto-N-fucopentaose II | 1.11 (0.83, 1.49) | 0.49 | 1.03 (0.85, 1.25) | 0.78 | 1.13 (0.94, 1.36) | 0.19 | 0.98 (0.87, 1.11) | 0.74 |
| Lacto-N-fucopentaose III | 1.07 (0.90, 1.28) | 0.41 | 1.05 (0.82, 1.35) | 0.71 | 1.13 (0.99, 1.29) | 0.06 | 1.03 (0.90, 1.19) | 0.67 |
| Lacto-N-fucopentaose V | 1.10 (0.82, 1.48) | 0.52 | 0.99 (0.74, 1.32) | 0.94 | 1.19 (0.98, 1.45) | 0.08 | 0.97 (0.80, 1.19) | 0.8 |
| Lacto-N-hexaose | 1.07 (0.88, 1.30) | 0.51 | 1.02 (0.89, 1.17) | 0.76 | 1.04 (0.92, 1.17) | 0.54 | 1.01 (0.93, 1.09) | 0.88 |
| Lacto-N-neo-difucohexaose I | 1.14 (0.83, 1.57) | 0.42 | . (., .) | . | 1.04 (0.89, 1.21) | 0.63 | . (., .) | . |
| Lacto-N-neo-difucohexaose II | 1.04 (0.80, 1.34) | 0.78 | 1.04 (0.82, 1.31) | 0.77 | 1.13 (0.96, 1.33) | 0.14 | 1.03 (0.90, 1.18) | 0.64 |
| Lacto-N-neo-hexaose | 1.14 (0.91, 1.44) | 0.26 | 1.02 (0.83, 1.26) | 0.85 | 1.08 (0.95, 1.21) | 0.23 | 1.00 (0.88, 1.14) | 0.99 |
| Lacto-N-neo-octaose | 1.13 (0.96, 1.35) | 0.15 | 1.01 (0.84, 1.20) | 0.94 | 1.08 (0.98, 1.19) | 0.12 | 1.01 (0.91, 1.12) | 0.89 |
| Lacto-N-tetraose | 1.06 (0.88, 1.28) | 0.51 | 1.09 (0.88, 1.34) | 0.44 | 1.08 (0.94, 1.23) | 0.28 | 1.04 (0.92, 1.17) | 0.52 |
| Lacto-N-triose II | 1.09 (0.92, 1.30) | 0.33 | 1.15 (0.89, 1.47) | 0.29 | 1.10 (0.97, 1.24) | 0.13 | 1.03 (0.89, 1.18) | 0.72 |
| Lactose-3'-Sulfate | 1.02 (0.84, 1.25) | 0.82 | 1.09 (0.89, 1.33) | 0.42 | 1.01 (0.90, 1.13) | 0.89 | 1.04 (0.92, 1.19) | 0.53 |
| Sialyllacto-N-hexaose-X1 | 1.04 (0.85, 1.26) | 0.72 | 0.92 (0.77, 1.10) | 0.36 | 1.04 (0.93, 1.16) | 0.51 | 1.02 (0.94, 1.11) | 0.56 |
| Sialyllacto-N-hexaose-X2 | 1.13 (0.89, 1.43) | 0.33 | 1.10 (0.86, 1.42) | 0.44 | 0.97 (0.85, 1.12) | 0.71 | 1.08 (0.93, 1.25) | 0.3 |
| Sialyllacto-N-tetraose a | 1.05 (0.88, 1.26) | 0.59 | 0.95 (0.79, 1.14) | 0.6 | 1.04 (0.93, 1.17) | 0.49 | 1.05 (0.97, 1.14) | 0.23 |
| Sialyllacto-N-tetraose b | 1.07 (0.88, 1.29) | 0.49 | 1.18 (0.95, 1.47) | 0.14 | 1.07 (0.94, 1.22) | 0.3 | 1.01 (0.87, 1.17) | 0.9 |
| Sialyllacto-N-tetraose c | 1.09 (0.91, 1.32) | 0.33 | 1.14 (0.91, 1.43) | 0.24 | 1.00 (0.89, 1.13) | 0.97 | 1.10 (0.97, 1.25) | 0.14 |
| Trifucosyl(1-2,1-2,1-3)-iso-lacto-N-octa | 1.05 (0.81, 1.37) | 0.7 | . (., .) | . | 0.95 (0.81, 1.12) | 0.53 | . (., .) | . |
| Trifucosyllacto-N-hexaose I | 1.10 (0.89, 1.34) | 0.38 | . (., .) | . | 1.01 (0.87, 1.17) | 0.94 | . (., .) | . |
| Trifucosyllacto-N-hexaose-X1 | 1.14 (0.91, 1.43) | 0.25 | . (., .) | . | 1.09 (0.95, 1.25) | 0.23 | . (., .) | . |
| Trifucosyllacto-N-octaose-X1 | 1.19 (0.96, 1.47) | 0.12 | . (., .) | . | 1.05 (0.91, 1.23) | 0.49 | . (., .) | . |
| Trifucosyllacto-N-tetraose-X5 | 1.10 (0.89, 1.35) | 0.38 | . (., .) | . | 1.01 (0.90, 1.13) | 0.91 | . (., .) | . |
| a-Heptasaccharide | 1.13 (0.93, 1.37) | 0.22 | . (., .) | . | 1.10 (0.97, 1.25) | 0.14 | . (., .) | . |
| a-Pentasaccharide | 1.16 (0.94, 1.43) | 0.16 | . (., .) | . | 1.11 (0.96, 1.27) | 0.16 | . (., .) | . |
| a-Tetrasaccharide | 1.19 (0.99, 1.43) | 0.07 | . (., .) | . | 1.10 (0.97, 1.25) | 0.14 | . (., .) | . |

Associations determined by modified Poisson regression. Models adjusted for batch, child sex, duration of exclusive breastfeeding (weeks) and gestation age (weeks). Bold denotes statistically significant at nominal level (p<0.05). *Denotes statistically significant after correction for multiple testing (FDR<0.010). RR: Risk Ratio; CI: Confidence Interval; URTI: Upper respiratory tract infections; HMO: Human milk oligosaccharides.

**Table S17:** Adjusted associations between human milk oligosaccharides measured at 6 months of lactation with otitis media in the first or second year of life in the Ulm SPATZ Health Study.

|  | **OM at 1 year** | | **OM at 2 years** | |
| --- | --- | --- | --- | --- |
| **HMO Structure** | RR (95% CI) | p | RR (95% CI) | p |
| 2,3 Difucosyllactose | 1.24 (0.80, 1.92) | 0.34 | 1.15 (0.85, 1.56) | 0.37 |
| 2-Fucosyllactose | 1.28 (0.80, 2.03) | 0.30 | 1.29 (0.93, 1.78) | 0.13 |
| 3'-Sialyl-3-fucosyllactose | 1.04 (0.66, 1.66) | 0.85 | 0.87 (0.62, 1.21) | 0.41 |
| **3-Fucosyllactose** | 0.79 (0.53, 1.16) | 0.22 | **0.73 (0.53, 0.99)** | **0.05** |
| 3-Galactosyllactose | 0.98 (0.51, 1.89) | 0.96 | 1.08 (0.76, 1.53) | 0.67 |
| 3-sialyllactose | 1.36 (0.83, 2.22) | 0.22 | 1.09 (0.81, 1.48) | 0.56 |
| 6'-Sialyl-N-acetyllactosamine | 1.04 (0.71, 1.54) | 0.84 | 0.99 (0.74, 1.34) | 0.96 |
| 6-Galactosyllactose | 0.93 (0.58, 1.49) | 0.77 | 0.97 (0.69, 1.36) | 0.86 |
| 6-sialyllactose | 0.82 (0.53, 1.27) | 0.37 | 0.93 (0.69, 1.24) | 0.60 |
| Difucosyl-lacto-N-hexaose I | 1.42 (0.87, 2.31) | 0.16 | 1.20 (0.88, 1.65) | 0.25 |
| Difucosyl-lacto-N-hexaose II | 1.10 (0.68, 1.78) | 0.71 | 0.73 (0.51, 1.04) | 0.08 |
| **Difucosyl-lacto-N-hexaose-X1** | **1.68 (1.09, 2.57)** | **0.02** | **1.38 (1.03, 1.86)** | **0.03** |
| **Difucosyl-lacto-N-hexaose-X2** | **1.76 (1.02, 3.04)** | **0.04** | 1.08 (0.77, 1.52) | 0.65 |
| Difucosyl-lacto-N-hexaose-X3 | 1.06 (0.57, 1.95) | 0.86 | 0.94 (0.67, 1.33) | 0.73 |
| Difucosyl-lacto-N-neo-hexaose I | 0.95 (0.57, 1.58) | 0.84 | 0.89 (0.65, 1.22) | 0.47 |
| Difucosyl-para-lacto-N-hexaose I | 1.37 (0.79, 2.38) | 0.26 | 0.85 (0.59, 1.24) | 0.40 |
| Difucosyldisialyllacto-N-hexaose-X1 | 1.22 (0.75, 1.99) | 0.41 | 1.01 (0.65, 1.57) | 0.98 |
| **Difucosyldisialyllacto-N-hexaose-X2** | 0.90 (0.56, 1.45) | 0.67 | **0.70 (0.49, 1.00)** | **0.05** |
| Difucosylsialyllacto-N-hexaose-X1 | 1.23 (0.73, 2.05) | 0.44 | 1.12 (0.79, 1.59) | 0.54 |
| Difucosylsialyllacto-N-hexaose-X2 | 1.01 (0.55, 1.83) | 0.98 | 0.88 (0.60, 1.30) | 0.52 |
| Disialyllacto-N-Hexaose-X1 | 1.31 (0.72, 2.37) | 0.38 | 1.05 (0.77, 1.45) | 0.74 |
| Disialyllacto-N-Hexaose-X2 | 1.52 (0.94, 2.45) | 0.09 | 1.11 (0.82, 1.49) | 0.51 |
| Disialyllacto-N-Hexaose-X5 | 0.95 (0.57, 1.58) | 0.85 | 0.99 (0.72, 1.36) | 0.95 |
| Disialyllacto-N-tetraose | 1.38 (0.68, 2.78) | 0.38 | 1.05 (0.73, 1.52) | 0.79 |
| Fucosyl(1-3)-iso-lacto-N-octaose | 1.26 (0.62, 2.58) | 0.53 | 1.03 (0.73, 1.47) | 0.86 |
| Fucosyl-para-lacto-N-hexaose I | 1.60 (0.91, 2.79) | 0.10 | 1.20 (0.86, 1.67) | 0.28 |
| Fucosyldiasialyllacto-N-hexaose-X1 | 1.09 (0.50, 2.37) | 0.83 | 1.02 (0.69, 1.52) | 0.91 |
| Fucosyldiasialyllacto-N-hexaose-X2 | 1.33 (0.59, 3.03) | 0.49 | 1.14 (0.76, 1.69) | 0.53 |
| Fucosyldisialyllacto-N-tetraose-X2 | 1.26 (0.84, 1.91) | 0.27 | 0.90 (0.64, 1.25) | 0.52 |
| Fucosyllacto-N-hexaose II | 1.14 (0.53, 2.41) | 0.74 | 1.02 (0.70, 1.47) | 0.93 |
| **Fucosyllacto-N-hexaose-X1** | **1.53 (1.03, 2.27)** | **0.04** | 1.18 (0.85, 1.62) | 0.32 |
| Fucosyllacto-N-hexaose-X2 | 1.29 (0.69, 2.40) | 0.42 | 1.06 (0.73, 1.53) | 0.76 |
| Fucosyllacto-N-hexaose-X4 | 1.38 (0.92, 2.08) | 0.12 | 1.02 (0.76, 1.38) | 0.87 |
| Fucosyllacto-N-octaose-X1 | 1.19 (0.63, 2.27) | 0.60 | 1.03 (0.67, 1.57) | 0.90 |
| Fucosyllacto-N-octaose-X2 | 1.22 (0.74, 2.02) | 0.43 | 0.98 (0.68, 1.39) | 0.90 |
| **Fucosyllacto-N-sulfate-X1** | **1.72 (1.01, 2.91)** | **0.04** | **1.44 (1.04, 2.02)** | **0.03** |
| Fucosylsialyllacto-N-Hexaose-X1 | 1.44 (0.83, 2.51) | 0.19 | 0.92 (0.61, 1.36) | 0.66 |
| Fucosylsialyllacto-N-Hexaose-X2 | 1.51 (0.87, 2.60) | 0.14 | 1.28 (0.91, 1.78) | 0.15 |
| Fucosylsialyllacto-N-Hexaose-X3 | 1.09 (0.60, 2.00) | 0.78 | 1.19 (0.86, 1.64) | 0.30 |
| Fucosylsialyllacto-N-Hexaose-X4 | 0.95 (0.58, 1.56) | 0.85 | 0.97 (0.70, 1.34) | 0.85 |
| Fucosylsialyllacto-N-Hexaose-X5 | 0.56 (0.24, 1.31) | 0.18 | **0.52 (0.31, 0.88)** | **0.01** |
| Fucosylsialyllacto-N-Hexaose-X6 | 0.84 (0.44, 1.61) | 0.61 | 1.10 (0.77, 1.59) | 0.60 |
| Fucosylsialyllacto-N-neo-tetraose c | 1.01 (0.60, 1.71) | 0.96 | 0.80 (0.56, 1.14) | 0.21 |
| Fucosylsialyllacto-N-tetraose a | 0.99 (0.58, 1.69) | 0.97 | 0.80 (0.56, 1.15) | 0.23 |
| **Fucosylsialyllacto-N-tetraose b** | **1.87 (1.25, 2.79)** | **0.002** | **1.37 (1.05, 1.77)** | **0.02** |
| Fucosylsialyllacto-N-tetraose-X1 | 1.49 (0.87, 2.56) | 0.15 | 1.03 (0.71, 1.49) | 0.87 |
| Lacto-N-decaose-X1 | 1.54 (0.91, 2.60) | 0.11 | 1.19 (0.80, 1.77) | 0.39 |
| **Lacto-N-difucohexaose I** | **1.86 (1.23, 2.80)** | **0.003** | 1.22 (0.90, 1.66) | 0.20 |
| **Lacto-N-fucopentaose I** | **1.81 (1.28, 2.55)** | **0.001** | **1.39 (1.04, 1.86)** | **0.03** |
| Lacto-N-fucopentaose II | 1.16 (0.76, 1.76) | 0.49 | 0.79 (0.55, 1.12) | 0.18 |
| Lacto-N-fucopentaose III | 1.24 (0.70, 2.19) | 0.46 | 1.01 (0.71, 1.44) | 0.96 |
| Lacto-N-fucopentaose V | 1.19 (0.72, 1.96) | 0.50 | 0.80 (0.57, 1.11) | 0.17 |
| Lacto-N-hexaose | 1.29 (0.69, 2.41) | 0.43 | 1.10 (0.79, 1.52) | 0.58 |
| **Lacto-N-neo-difucohexaose I** | **1.62 (1.08, 2.43)** | **0.02** | 1.30 (0.97, 1.75) | 0.08 |
| Lacto-N-neo-difucohexaose II | 0.94 (0.63, 1.42) | 0.78 | 0.74 (0.55, 1.00) | 0.05 |
| **Lacto-N-neo-hexaose** | **1.64 (1.00, 2.70)** | **0.05** | 1.19 (0.87, 1.63) | 0.29 |
| Lacto-N-neo-octaose | 1.47 (0.95, 2.27) | 0.09 | 1.14 (0.82, 1.58) | 0.43 |
| Lacto-N-tetraose | 1.54 (0.80, 2.95) | 0.19 | 1.03 (0.73, 1.47) | 0.85 |
| Lacto-N-triose II | 1.26 (0.66, 2.38) | 0.48 | 1.07 (0.77, 1.49) | 0.68 |
| Lactose-3'-Sulfate | 1.22 (0.70, 2.14) | 0.48 | 1.09 (0.75, 1.57) | 0.66 |
| Sialyllacto-N-hexaose-X1 | 1.05 (0.50, 2.21) | 0.90 | 0.98 (0.67, 1.43) | 0.91 |
| Sialyllacto-N-hexaose-X2 | 1.07 (0.65, 1.75) | 0.80 | 0.99 (0.71, 1.38) | 0.94 |
| Sialyllacto-N-tetraose a | 1.09 (0.55, 2.16) | 0.81 | 1.19 (0.86, 1.65) | 0.29 |
| Sialyllacto-N-tetraose b | 1.68 (0.97, 2.90) | 0.06 | 1.01 (0.70, 1.45) | 0.98 |
| Sialyllacto-N-tetraose c | 1.09 (0.73, 1.63) | 0.66 | 1.04 (0.78, 1.40) | 0.78 |
| Trifucosyl(1-2,1-2,1-3)-iso-lacto-N-octa | 1.49 (0.89, 2.48) | 0.13 | 1.15 (0.83, 1.59) | 0.41 |
| **Trifucosyllacto-N-hexaose I** | **1.53 (1.01, 2.29)** | **0.04** | 1.13 (0.81, 1.57) | 0.47 |
| **Trifucosyllacto-N-hexaose-X1** | **2.03 (1.32, 3.14)** | **0.001** | 1.33 (0.99, 1.79) | 0.06 |
| Trifucosyllacto-N-octaose-X1 | 1.52 (0.92, 2.51) | 0.10 | 1.11 (0.80, 1.56) | 0.53 |
| **Trifucosyllacto-N-tetraose-X5** | **1.47 (1.01, 2.14)** | **0.04** | 1.20 (0.89, 1.60) | 0.23 |
| a-Heptasaccharide | 1.14 (0.64, 2.05) | 0.65 | 1.11 (0.82, 1.51) | 0.50 |
| a-Pentasaccharide | 1.32 (0.72, 2.40) | 0.37 | 1.14 (0.83, 1.56) | 0.43 |
| a-Tetrasaccharide | 1.16 (0.59, 2.28) | 0.67 | 1.05 (0.75, 1.48) | 0.77 |

Associations determined by modified Poisson regression. Models adjusted for batch, child sex, duration of exclusive breastfeeding (weeks) and gestation age (weeks). Bold denotes statistically significant at nominal level (p<0.05). *Denotes statistically significant after correction for multiple testing (FDR<0.010). RR: Risk Ratio; CI: Confidence Interval; OM: Otitis Media; HMO: Human milk oligosaccharides.

**Table S18:** Adjusted associations between human milk oligosaccharides measured at 6 months of lactation with lower respiratory tract infections in the first or second year of life in the Ulm SPATZ Health Study.

|  | **LRTI at 1 year** | | **LRTI at 2 years** | |
| --- | --- | --- | --- | --- |
| **HMO Structure** | RR (95% CI) | p | RR (95% CI) | p |
| 2,3 Difucosyllactose | 0.76 (0.50, 1.15) | 0.19 | 0.85 (0.65, 1.11) | 0.23 |
| 2-Fucosyllactose | 0.84 (0.50, 1.38) | 0.49 | 1.13 (0.85, 1.51) | 0.38 |
| 3'-Sialyl-3-fucosyllactose | 1.02 (0.65, 1.59) | 0.93 | 0.78 (0.57, 1.06) | 0.11 |
| **3-Fucosyllactose** | 0.83 (0.57, 1.23) | 0.36 | **0.71 (0.55, 0.93)** | **0.01** |
| 3-Galactosyllactose | 1.30 (0.87, 1.95) | 0.20 | 1.04 (0.77, 1.39) | 0.81 |
| 3-sialyllactose | 1.19 (0.77, 1.85) | 0.42 | 1.12 (0.84, 1.50) | 0.43 |
| 6'-Sialyl-N-acetyllactosamine | 1.23 (0.84, 1.79) | 0.29 | 1.12 (0.86, 1.45) | 0.41 |
| 6-Galactosyllactose | 0.81 (0.58, 1.12) | 0.20 | 0.81 (0.65, 1.01) | 0.06 |
| 6-sialyllactose | 1.04 (0.69, 1.56) | 0.86 | 1.08 (0.83, 1.41) | 0.55 |
| **Difucosyl-lacto-N-hexaose I** | 1.14 (0.77, 1.69) | 0.51 | **1.36 (1.06, 1.74)** | **0.02** |
| Difucosyl-lacto-N-hexaose II | 1.20 (0.79, 1.83) | 0.39 | 0.90 (0.66, 1.22) | 0.50 |
| **Difucosyl-lacto-N-hexaose-X1** | 1.21 (0.85, 1.74) | 0.30 | **1.30 (1.02, 1.65)** | **0.03** |
| Difucosyl-lacto-N-hexaose-X2 | 1.02 (0.69, 1.51) | 0.92 | 0.95 (0.72, 1.25) | 0.73 |
| Difucosyl-lacto-N-hexaose-X3 | 0.87 (0.61, 1.25) | 0.46 | 0.81 (0.64, 1.01) | 0.06 |
| **Difucosyl-lacto-N-neo-hexaose I** | 0.69 (0.45, 1.04) | 0.08 | **0.74 (0.59, 0.93)** | **0.01** |
| Difucosyl-para-lacto-N-hexaose I | 1.22 (0.78, 1.92) | 0.39 | 0.87 (0.64, 1.18) | 0.37 |
| Difucosyldisialyllacto-N-hexaose-X1 | 1.13 (0.77, 1.65) | 0.53 | 1.08 (0.83, 1.42) | 0.56 |
| Difucosyldisialyllacto-N-hexaose-X2 | 1.13 (0.78, 1.64) | 0.52 | 0.94 (0.72, 1.23) | 0.66 |
| Difucosylsialyllacto-N-hexaose-X1 | 0.90 (0.58, 1.37) | 0.61 | 0.99 (0.75, 1.31) | 0.94 |
| Difucosylsialyllacto-N-hexaose-X2 | 0.81 (0.51, 1.27) | 0.36 | 0.88 (0.65, 1.19) | 0.40 |
| Disialyllacto-N-Hexaose-X1 | 1.11 (0.77, 1.60) | 0.58 | 1.16 (0.90, 1.49) | 0.26 |
| Disialyllacto-N-Hexaose-X2 | 1.32 (0.96, 1.82) | 0.09 | 1.17 (0.94, 1.46) | 0.15 |
| Disialyllacto-N-Hexaose-X5 | 1.10 (0.76, 1.60) | 0.60 | 1.12 (0.86, 1.45) | 0.41 |
| Disialyllacto-N-tetraose | 1.39 (0.92, 2.08) | 0.11 | 1.22 (0.93, 1.60) | 0.15 |
| **Fucosyl(1-3)-iso-lacto-N-octaose** | **1.75 (1.26, 2.43)** | **0.001** | **1.41 (1.09, 1.83)** | **0.01** |
| **Fucosyl-para-lacto-N-hexaose I** | **1.55 (1.13, 2.11)** | **0.01** | 1.25 (0.99, 1.58) | 0.06 |
| Fucosyldiasialyllacto-N-hexaose-X1 | 1.13 (0.71, 1.80) | 0.61 | 1.17 (0.86, 1.59) | 0.31 |
| Fucosyldiasialyllacto-N-hexaose-X2 | 1.21 (0.80, 1.84) | 0.36 | 1.08 (0.81, 1.46) | 0.59 |
| Fucosyldisialyllacto-N-tetraose-X2 | 1.18 (0.78, 1.77) | 0.43 | 0.84 (0.63, 1.14) | 0.27 |
| **Fucosyllacto-N-hexaose II** | **1.51 (1.05, 2.19)** | **0.03** | 1.21 (0.91, 1.60) | 0.19 |
| Fucosyllacto-N-hexaose-X1 | 1.20 (0.87, 1.66) | 0.27 | 1.16 (0.92, 1.45) | 0.21 |
| Fucosyllacto-N-hexaose-X2 | 1.29 (0.90, 1.85) | 0.17 | 1.23 (0.96, 1.58) | 0.10 |
| Fucosyllacto-N-hexaose-X4 | 1.06 (0.76, 1.46) | 0.75 | 0.99 (0.79, 1.23) | 0.91 |
| Fucosyllacto-N-octaose-X1 | 1.02 (0.62, 1.67) | 0.94 | 1.10 (0.83, 1.47) | 0.49 |
| Fucosyllacto-N-octaose-X2 | 1.24 (0.87, 1.76) | 0.23 | 0.95 (0.71, 1.28) | 0.75 |
| Fucosyllacto-N-sulfate-X1 | 1.20 (0.81, 1.79) | 0.37 | 1.13 (0.86, 1.48) | 0.38 |
| **Fucosylsialyllacto-N-Hexaose-X1** | **1.58 (1.01, 2.47)** | **0.04** | 1.13 (0.82, 1.57) | 0.45 |
| Fucosylsialyllacto-N-Hexaose-X2 | 1.11 (0.70, 1.76) | 0.66 | 1.27 (0.96, 1.68) | 0.10 |
| Fucosylsialyllacto-N-Hexaose-X3 | 1.00 (0.63, 1.56) | 0.99 | 1.27 (0.98, 1.65) | 0.07 |
| Fucosylsialyllacto-N-Hexaose-X4 | 0.84 (0.60, 1.17) | 0.30 | 0.91 (0.71, 1.18) | 0.50 |
| Fucosylsialyllacto-N-Hexaose-X5 | 1.41 (0.87, 2.27) | 0.16 | 1.06 (0.76, 1.48) | 0.74 |
| Fucosylsialyllacto-N-Hexaose-X6 | 0.81 (0.52, 1.27) | 0.35 | 0.85 (0.60, 1.19) | 0.35 |
| Fucosylsialyllacto-N-neo-tetraose c | 1.08 (0.71, 1.66) | 0.71 | 0.80 (0.61, 1.03) | 0.09 |
| Fucosylsialyllacto-N-tetraose a | 1.06 (0.65, 1.74) | 0.80 | 0.86 (0.62, 1.20) | 0.37 |
| Fucosylsialyllacto-N-tetraose b | 1.19 (0.85, 1.66) | 0.30 | 1.15 (0.94, 1.42) | 0.18 |
| Fucosylsialyllacto-N-tetraose-X1 | 1.27 (0.76, 2.11) | 0.36 | 0.97 (0.68, 1.38) | 0.86 |
| Lacto-N-decaose-X1 | 1.34 (0.85, 2.12) | 0.21 | 1.21 (0.89, 1.66) | 0.22 |
| Lacto-N-difucohexaose I | 1.02 (0.71, 1.47) | 0.92 | 0.99 (0.78, 1.25) | 0.90 |
| Lacto-N-fucopentaose I | 1.33 (0.93, 1.90) | 0.12 | **1.36 (1.09, 1.71)** | **0.01** |
| Lacto-N-fucopentaose II | 1.31 (0.86, 2.00) | 0.21 | 0.92 (0.67, 1.25) | 0.59 |
| Lacto-N-fucopentaose III | 0.93 (0.63, 1.37) | 0.70 | 0.85 (0.66, 1.08) | 0.18 |
| Lacto-N-fucopentaose V | 1.46 (0.96, 2.22) | 0.08 | 0.95 (0.71, 1.28) | 0.74 |
| **Lacto-N-hexaose** | **1.59 (1.15, 2.20)** | **0.01** | **1.35 (1.05, 1.72)** | **0.02** |
| Lacto-N-neo-difucohexaose I | 0.96 (0.65, 1.42) | 0.84 | 1.05 (0.83, 1.33) | 0.67 |
| Lacto-N-neo-difucohexaose II | 0.87 (0.57, 1.33) | 0.52 | **0.69 (0.54, 0.87)** | **0.002** |
| Lacto-N-neo-hexaose | 1.21 (0.90, 1.63) | 0.22 | 1.15 (0.92, 1.43) | 0.21 |
| Lacto-N-neo-octaose | 1.10 (0.74, 1.66) | 0.63 | 1.08 (0.83, 1.42) | 0.56 |
| Lacto-N-tetraose | 1.88 (1.40, 2.53) | **<0.001*** | **1.30 (1.01, 1.68)** | **0.04** |
| Lacto-N-triose II | 2.33 (1.53, 3.54) | **<0.001*** | **1.39 (1.08, 1.79)** | **0.01** |
| Lactose-3'-Sulfate | 1.01 (0.63, 1.63) | 0.96 | 0.96 (0.70, 1.31) | 0.80 |
| Sialyllacto-N-hexaose-X1 | 1.33 (0.88, 2.02) | 0.17 | 1.08 (0.79, 1.48) | 0.61 |
| Sialyllacto-N-hexaose-X2 | 0.97 (0.64, 1.46) | 0.88 | 1.02 (0.78, 1.34) | 0.89 |
| Sialyllacto-N-tetraose a | 1.46 (1.06, 1.99) | **0.02** | 1.46 (1.20, 1.78) | **<0.001** |
| **Sialyllacto-N-tetraose b** | **1.61 (1.14, 2.28)** | **0.01** | 1.12 (0.85, 1.47) | 0.41 |
| Sialyllacto-N-tetraose c | 1.12 (0.82, 1.52) | 0.49 | 1.07 (0.84, 1.35) | 0.59 |
| **Trifucosyl(1-2,1-2,1-3)-iso-lacto-N-octa** | 1.27 (0.89, 1.80) | 0.19 | **1.36 (1.07, 1.72)** | **0.01** |
| Trifucosyllacto-N-hexaose I | 0.92 (0.64, 1.33) | 0.67 | 0.94 (0.72, 1.22) | 0.64 |
| Trifucosyllacto-N-hexaose-X1 | 1.02 (0.72, 1.46) | 0.90 | 0.95 (0.74, 1.21) | 0.66 |
| **Trifucosyllacto-N-octaose-X1** | **1.48 (1.08, 2.03)** | **0.01** | **1.25 (1.00, 1.56)** | **0.05** |
| Trifucosyllacto-N-tetraose-X5 | 0.95 (0.67, 1.34) | 0.76 | 0.97 (0.75, 1.24) | 0.80 |
| a-Heptasaccharide | 1.05 (0.69, 1.59) | 0.83 | 1.03 (0.80, 1.33) | 0.82 |
| a-Pentasaccharide | 1.13 (0.77, 1.64) | 0.54 | 1.08 (0.84, 1.37) | 0.56 |
| a-Tetrasaccharide | 1.30 (0.85, 1.99) | 0.22 | 1.17 (0.90, 1.52) | 0.24 |

Associations determined by modified Poisson regression. Models adjusted for batch, child sex, duration of exclusive breastfeeding (weeks) and gestation age (weeks). **Bold** denotes statistically significant at nominal level (p<0.05). *****Denotes statistically significant after correction for multiple testing (FDR<0.010). RR: Risk Ratio; CI: Confidence Interval; LRTI: lower respiratory tract infections; HMO: Human milk oligosaccharides

**Table S19:** Adjusted associations between human milk oligosaccharides measured at 6 months of lactation with upper respiratory tract infections in the first or second year of life in the Ulm SPATZ Health Study.

|  | **URTI at 1 year** | | **URTI at 2 years** | |
| --- | --- | --- | --- | --- |
| **HMO Structure** | RR (95% CI) | p | RR (95% CI) | p |
| 2,3 Difucosyllactose | 1.00 (0.85, 1.18) | 0.97 | 0.95 (0.84, 1.07) | 0.39 |
| 2-Fucosyllactose | 0.93 (0.77, 1.14) | 0.49 | 0.90 (0.78, 1.04) | 0.16 |
| 3'-Sialyl-3-fucosyllactose | 1.15 (0.98, 1.35) | 0.09 | 1.09 (0.97, 1.23) | 0.15 |
| 3-Fucosyllactose | 1.03 (0.88, 1.20) | 0.74 | 1.04 (0.92, 1.18) | 0.50 |
| 3-Galactosyllactose | 1.12 (0.97, 1.30) | 0.12 | 1.05 (0.96, 1.16) | 0.29 |
| 3-sialyllactose | 1.09 (0.98, 1.22) | 0.12 | 1.01 (0.94, 1.09) | 0.72 |
| 6'-Sialyl-N-acetyllactosamine | 1.08 (0.93, 1.26) | 0.31 | 0.99 (0.89, 1.11) | 0.92 |
| 6-Galactosyllactose | 0.99 (0.83, 1.18) | 0.89 | 0.98 (0.88, 1.10) | 0.78 |
| 6-sialyllactose | 1.06 (0.92, 1.22) | 0.45 | 0.99 (0.89, 1.10) | 0.88 |
| Difucosyl-lacto-N-hexaose I | 0.98 (0.84, 1.15) | 0.82 | 0.94 (0.84, 1.06) | 0.35 |
| Difucosyl-lacto-N-hexaose II | 1.12 (0.93, 1.35) | 0.24 | 1.09 (0.95, 1.25) | 0.22 |
| Difucosyl-lacto-N-hexaose-X1 | 1.02 (0.85, 1.21) | 0.84 | 0.98 (0.86, 1.12) | 0.77 |
| Difucosyl-lacto-N-hexaose-X2 | 1.07 (0.92, 1.24) | 0.39 | 1.02 (0.89, 1.16) | 0.82 |
| **Difucosyl-lacto-N-hexaose-X3** | **1.25 (1.06, 1.48)** | **0.01** | 1.11 (0.99, 1.26) | 0.08 |
| Difucosyl-lacto-N-neo-hexaose I | 1.14 (0.94, 1.39) | 0.18 | 1.12 (0.97, 1.29) | 0.14 |
| **Difucosyl-para-lacto-N-hexaose I** | **1.22 (1.02, 1.46)** | **0.03** | 1.14 (1.00, 1.31) | 0.06 |
| Difucosyldisialyllacto-N-hexaose-X1 | 1.07 (0.87, 1.31) | 0.51 | 0.94 (0.78, 1.12) | 0.46 |
| Difucosyldisialyllacto-N-hexaose-X2 | 1.07 (0.90, 1.27) | 0.45 | 1.01 (0.89, 1.14) | 0.92 |
| Difucosylsialyllacto-N-hexaose-X1 | 1.05 (0.88, 1.26) | 0.57 | 0.94 (0.83, 1.06) | 0.30 |
| Difucosylsialyllacto-N-hexaose-X2 | 0.94 (0.78, 1.12) | 0.48 | 0.92 (0.79, 1.06) | 0.25 |
| Disialyllacto-N-Hexaose-X1 | 1.08 (0.93, 1.25) | 0.31 | 0.99 (0.88, 1.11) | 0.83 |
| Disialyllacto-N-Hexaose-X2 | 1.15 (0.99, 1.33) | 0.06 | 0.99 (0.90, 1.09) | 0.86 |
| Disialyllacto-N-Hexaose-X5 | 1.10 (0.94, 1.29) | 0.24 | 1.01 (0.90, 1.14) | 0.83 |
| Disialyllacto-N-tetraose | 1.10 (0.91, 1.33) | 0.30 | 0.98 (0.86, 1.11) | 0.70 |
| Fucosyl(1-3)-iso-lacto-N-octaose | 1.06 (0.93, 1.22) | 0.38 | 1.04 (0.95, 1.13) | 0.39 |
| Fucosyl-para-lacto-N-hexaose I | 1.14 (0.97, 1.34) | 0.11 | 1.07 (0.94, 1.22) | 0.28 |
| Fucosyldiasialyllacto-N-hexaose-X1 | 1.07 (0.89, 1.28) | 0.46 | 0.98 (0.87, 1.10) | 0.73 |
| **Fucosyldiasialyllacto-N-hexaose-X2** | **1.24 (1.01, 1.54)** | **0.04** | 1.04 (0.91, 1.19) | 0.58 |
| Fucosyldisialyllacto-N-tetraose-X2 | 1.18 (0.99, 1.40) | 0.06 | 1.08 (0.95, 1.24) | 0.24 |
| Fucosyllacto-N-hexaose II | 1.04 (0.88, 1.24) | 0.65 | 1.03 (0.93, 1.15) | 0.56 |
| Fucosyllacto-N-hexaose-X1 | 1.04 (0.86, 1.24) | 0.71 | 0.95 (0.84, 1.08) | 0.45 |
| Fucosyllacto-N-hexaose-X2 | 1.07 (0.90, 1.25) | 0.45 | 0.94 (0.83, 1.08) | 0.40 |
| **Fucosyllacto-N-hexaose-X4** | **1.19 (1.04, 1.35)** | **0.01** | 1.05 (0.98, 1.13) | 0.15 |
| Fucosyllacto-N-octaose-X1 | 1.04 (0.88, 1.24) | 0.63 | 0.94 (0.80, 1.10) | 0.42 |
| Fucosyllacto-N-octaose-X2 | 1.10 (0.97, 1.25) | 0.13 | 1.06 (0.97, 1.15) | 0.21 |
| Fucosyllacto-N-sulfate-X1 | 1.08 (0.93, 1.25) | 0.34 | 1.01 (0.93, 1.10) | 0.83 |
| Fucosylsialyllacto-N-Hexaose-X1 | 1.10 (0.91, 1.33) | 0.32 | 1.01 (0.88, 1.15) | 0.89 |
| Fucosylsialyllacto-N-Hexaose-X2 | 0.96 (0.79, 1.17) | 0.69 | 0.93 (0.80, 1.08) | 0.33 |
| Fucosylsialyllacto-N-Hexaose-X3 | 0.93 (0.79, 1.09) | 0.38 | 0.91 (0.80, 1.02) | 0.12 |
| Fucosylsialyllacto-N-Hexaose-X4 | 1.16 (1.00, 1.35) | 0.06 | 1.03 (0.93, 1.15) | 0.52 |
| Fucosylsialyllacto-N-Hexaose-X5 | 1.12 (0.94, 1.34) | 0.21 | 1.06 (0.93, 1.20) | 0.39 |
| **Fucosylsialyllacto-N-Hexaose-X6** | **1.15 (1.01, 1.33)** | **0.04** | 1.06 (0.97, 1.16) | 0.17 |
| **Fucosylsialyllacto-N-neo-tetraose c** | **1.34 (1.13, 1.60)** | **0.001** | **1.14 (1.01, 1.28)** | **0.04** |
| Fucosylsialyllacto-N-tetraose a | 1.06 (0.87, 1.29) | 0.59 | 1.07 (0.93, 1.24) | 0.34 |
| Fucosylsialyllacto-N-tetraose b | 1.04 (0.88, 1.23) | 0.64 | 0.95 (0.85, 1.07) | 0.41 |
| **Fucosylsialyllacto-N-tetraose-X1** | **1.33 (1.13, 1.56)** | **0.001** | **1.15 (1.02, 1.30)** | **0.02** |
| Lacto-N-decaose-X1 | 1.06 (0.90, 1.24) | 0.49 | 0.98 (0.84, 1.13) | 0.75 |
| Lacto-N-difucohexaose I | 1.07 (0.92, 1.24) | 0.40 | 1.01 (0.90, 1.13) | 0.88 |
| Lacto-N-fucopentaose I | 0.98 (0.81, 1.19) | 0.86 | 0.91 (0.79, 1.06) | 0.22 |
| Lacto-N-fucopentaose II | 1.14 (0.95, 1.37) | 0.15 | 1.11 (0.96, 1.27) | 0.16 |
| **Lacto-N-fucopentaose III** | **1.23 (1.03, 1.46)** | **0.02** | 1.11 (0.97, 1.27) | 0.12 |
| Lacto-N-fucopentaose V | 1.15 (0.95, 1.39) | 0.16 | 1.13 (0.97, 1.31) | 0.12 |
| Lacto-N-hexaose | 1.08 (0.93, 1.24) | 0.32 | 1.00 (0.90, 1.11) | 0.97 |
| Lacto-N-neo-difucohexaose I | 1.10 (0.94, 1.28) | 0.23 | 1.00 (0.90, 1.10) | 0.92 |
| Lacto-N-neo-difucohexaose II | 1.19 (1.00, 1.42) | 0.06 | 1.13 (0.98, 1.29) | 0.09 |
| Lacto-N-neo-hexaose | 1.08 (0.94, 1.24) | 0.27 | 1.02 (0.92, 1.13) | 0.67 |
| Lacto-N-neo-octaose | 1.12 (0.99, 1.27) | 0.07 | 1.05 (0.96, 1.14) | 0.28 |
| Lacto-N-tetraose | 1.11 (0.94, 1.30) | 0.22 | 1.07 (0.94, 1.21) | 0.34 |
| Lacto-N-triose II | 1.14 (0.97, 1.35) | 0.11 | 1.02 (0.92, 1.13) | 0.70 |
| Lactose-3'-Sulfate | 1.12 (0.92, 1.37) | 0.25 | 1.03 (0.90, 1.18) | 0.70 |
| Sialyllacto-N-hexaose-X1 | 1.08 (0.93, 1.26) | 0.33 | 1.02 (0.90, 1.14) | 0.79 |
| Sialyllacto-N-hexaose-X2 | 1.04 (0.88, 1.22) | 0.67 | 0.97 (0.86, 1.10) | 0.67 |
| Sialyllacto-N-tetraose a | 0.98 (0.85, 1.14) | 0.83 | 1.00 (0.90, 1.11) | 0.99 |
| Sialyllacto-N-tetraose b | 1.14 (0.96, 1.37) | 0.13 | 1.04 (0.92, 1.18) | 0.53 |
| **Sialyllacto-N-tetraose c** | **1.20 (1.05, 1.36)** | **0.01** | 1.04 (0.97, 1.12) | 0.29 |
| Trifucosyl(1-2,1-2,1-3)-iso-lacto-N-octa | 1.03 (0.87, 1.21) | 0.74 | 0.92 (0.81, 1.05) | 0.20 |
| Trifucosyllacto-N-hexaose I | 1.03 (0.88, 1.21) | 0.71 | 0.98 (0.86, 1.13) | 0.79 |
| Trifucosyllacto-N-hexaose-X1 | 1.10 (0.96, 1.27) | 0.18 | 1.04 (0.95, 1.15) | 0.36 |
| Trifucosyllacto-N-octaose-X1 | 1.11 (0.97, 1.26) | 0.12 | 1.03 (0.93, 1.14) | 0.62 |
| Trifucosyllacto-N-tetraose-X5 | 1.06 (0.93, 1.22) | 0.38 | 1.00 (0.91, 1.09) | 0.94 |
| a-Heptasaccharide | 1.11 (0.95, 1.30) | 0.19 | 1.08 (0.97, 1.21) | 0.14 |
| a-Pentasaccharide | 1.12 (0.96, 1.32) | 0.15 | 1.08 (0.96, 1.20) | 0.20 |
| a-Tetrasaccharide | 1.12 (0.96, 1.30) | 0.14 | 1.07 (0.97, 1.19) | 0.17 |

Associations determined by modified Poisson regression. Models adjusted for batch, child sex, duration of exclusive breastfeeding (weeks) and gestation age (weeks). Bold denotes statistically significant at nominal level (p<0.05). *Denotes statistically significant after correction for multiple testing (FDR<0.010). RR: Risk Ratio; CI: Confidence Interval; URTI: Upper respiratory tract infections; HMO: Human milk oligosaccharides

**Table S20**: Adjusted associations between human milk oligosaccharides measured at 6 months of lactation with otitis media (OM) in the first or second year of life stratified by secretor status in the Ulm SPATZ Health Study.

|  | **OM at 1 year** | | | | **OM at 2 years** | | | |
| --- | --- | --- | --- | --- | --- | --- | --- | --- |
|  | Secretor milk | | Non-secretor milk | | Secretor milk | | Non-secretor milk | |
| **HMO Structure** | RR (95% CI) | p | RR (95% CI) | p | RR (95% CI) | p1 | RR (95% CI) | p1 |
| 2,3 Difucosyllactose | 0.84 (0.31, 2.25) | 0.73 | . (., .) | . | 0.86 (0.47, 1.58) | 0.64 | . (., .) | . |
| 2-Fucosyllactose | 0.86 (0.38, 1.98) | 0.73 | . (., .) | . | 1.16 (0.69, 1.97) | 0.57 | . (., .) | . |
| 3'-Sialyl-3-fucosyllactose | 1.44 (0.71, 2.95) | 0.32 | 2.34 (0.86, 6.34) | 0.10 | 1.00 (0.61, 1.66) | 0.99 | 0.96 (0.42, 2.17) | 0.92 |
| **3-Fucosyllactose** | 0.91 (0.52, 1.59) | 0.74 | 1.61 (0.54, 4.75) | 0.39 | 0.81 (0.51, 1.28) | 0.37 | **0.62 (0.40, 0.96)** | **0.03** |
| **3-Galactosyllactose** | 0.89 (0.43, 1.84) | 0.75 | **3.82 (1.79, 8.16)** | **0.001** | 1.02 (0.69, 1.53) | 0.91 | **2.46 (1.32, 4.59)** | **0.01** |
| 3-sialyllactose | 1.35 (0.83, 2.19) | 0.23 | 2.53 (0.84, 7.60) | 0.10 | 1.07 (0.78, 1.48) | 0.67 | 1.59 (0.87, 2.92) | 0.1 |
| 6'-Sialyl-N-acetyllactosamine | 0.93 (0.58, 1.50) | 0.78 | 1.24 (0.62, 2.49) | 0.55 | 0.92 (0.65, 1.31) | 0.65 | 1.38 (0.86, 2.19) | 0.2 |
| **6-Galactosyllactose** | 0.83 (0.46, 1.48) | 0.52 | **2.92 (1.48, 5.73)** | **0.002** | 0.96 (0.63, 1.45) | 0.84 | 1.73 (0.91, 3.27) | 0.1 |
| 6-sialyllactose | 0.80 (0.49, 1.33) | 0.39 | 0.76 (0.32, 1.80) | 0.53 | 0.91 (0.66, 1.26) | 0.56 | 1.06 (0.59, 1.93) | 0.8 |
| Difucosyl-lacto-N-hexaose I | 1.18 (0.57, 2.41) | 0.66 | . (., .) | . | 1.09 (0.72, 1.65) | 0.69 | . (., .) | . |
| **Difucosyl-lacto-N-hexaose II** | 1.98 (0.70, 5.57) | 0.20 | 0.98 (0.49, 1.94) | 0.95 | 0.91 (0.50, 1.66) | 0.76 | **0.56 (0.40, 0.79)** | **0.001** |
| Difucosyl-lacto-N-hexaose-X1 | 1.57 (0.78, 3.15) | 0.20 | . (., .) | . | 1.34 (0.86, 2.09) | 0.19 | . (., .) | . |
| Difucosyl-lacto-N-hexaose-X2 | 1.75 (0.78, 3.93) | 0.17 | . (., .) | . | 0.90 (0.55, 1.45) | 0.65 | . (., .) | . |
| Difucosyl-lacto-N-hexaose-X3 | 1.09 (0.53, 2.24) | 0.81 | 1.41 (0.72, 2.75) | 0.32 | 0.92 (0.62, 1.37) | 0.70 | 1.26 (0.84, 1.90) | 0.27 |
| Difucosyl-lacto-N-neo-hexaose I | 1.02 (0.58, 1.79) | 0.94 | 0.96 (0.44, 2.09) | 0.91 | 0.91 (0.64, 1.31) | 0.62 | 0.85 (0.50, 1.46) | 0.56 |
| Difucosyl-para-lacto-N-hexaose I | 2.22 (0.81, 6.13) | 0.12 | 2.05 (0.52, 8.07) | 0.31 | 0.95 (0.56, 1.60) | 0.84 | 0.88 (0.44, 1.74) | 0.71 |
| Difucosyldisialyllacto-N-hexaose-X1 | 1.12 (0.70, 1.78) | 0.64 | . (., .) | . | 0.98 (0.65, 1.47) | 0.91 | . (., .) | . |
| **Difucosyldisialyllacto-N-hexaose-X2** | 1.49 (0.60, 3.72) | 0.40 | 0.78 (0.31, 1.98) | 0.61 | 0.90 (0.50, 1.64) | 0.73 | **0.48 (0.30, 0.78)** | **0.003** |
| Difucosylsialyllacto-N-hexaose-X1 | 0.96 (0.49, 1.88) | 0.91 | . (., .) | . | 0.99 (0.63, 1.55) | 0.95 | . (., .) | . |
| Difucosylsialyllacto-N-hexaose-X2 | 0.85 (0.41, 1.80) | 0.68 | . (., .) | . | 0.78 (0.52, 1.18) | 0.24 | . (., .) | . |
| Disialyllacto-N-Hexaose-X1 | 1.25 (0.57, 2.75) | 0.58 | 0.45 (0.20, 1.01) | 0.05 | 0.99 (0.67, 1.46) | 0.95 | 0.72 (0.33, 1.56) | 0.40 |
| Disialyllacto-N-Hexaose-X2 | 1.44 (0.81, 2.55) | 0.22 | 1.16 (0.43, 3.11) | 0.76 | 1.04 (0.74, 1.46) | 0.83 | 1.02 (0.57, 1.83) | 0.94 |
| Disialyllacto-N-Hexaose-X5 | 0.88 (0.43, 1.80) | 0.72 | 0.80 (0.46, 1.38) | 0.42 | 0.90 (0.59, 1.36) | 0.61 | 1.34 (0.81, 2.20) | 0.26 |
| Disialyllacto-N-tetraose | 1.57 (0.78, 3.17) | 0.21 | 1.29 (0.20, 8.46) | 0.79 | 1.09 (0.76, 1.57) | 0.64 | 1.34 (0.57, 3.18) | 0.51 |
| Fucosyl(1-3)-iso-lacto-N-octaose | 1.34 (0.62, 2.90) | 0.46 | 0.87 (0.33, 2.26) | 0.78 | 0.99 (0.67, 1.47) | 0.96 | 1.40 (0.75, 2.62) | 0.29 |
| Fucosyl-para-lacto-N-hexaose I | 1.64 (0.87, 3.11) | 0.13 | 0.96 (0.38, 2.42) | 0.92 | 1.10 (0.76, 1.61) | 0.62 | 1.58 (0.90, 2.78) | 0.11 |
| Fucosyldiasialyllacto-N-hexaose-X1 | 1.21 (0.53, 2.75) | 0.65 | 0.53 (0.14, 2.00) | 0.35 | 1.09 (0.71, 1.68) | 0.69 | 0.74 (0.39, 1.43) | 0.37 |
| Fucosyldiasialyllacto-N-hexaose-X2 | 1.48 (0.61, 3.58) | 0.38 | 1.13 (0.32, 4.00) | 0.85 | 1.14 (0.76, 1.71) | 0.53 | 1.67 (0.86, 3.26) | 0.13 |
| Fucosyldisialyllacto-N-tetraose-X2 | 2.17 (1.03, 4.59) | 0.04 | 1.83 (0.74, 4.55) | 0.19 | 1.05 (0.64, 1.74) | 0.84 | 0.96 (0.45, 2.07) | 0.92 |
| Fucosyllacto-N-hexaose II | 1.34 (0.56, 3.17) | 0.51 | 0.66 (0.24, 1.80) | 0.41 | 1.07 (0.69, 1.64) | 0.77 | 1.07 (0.58, 1.98) | 0.83 |
| Fucosyllacto-N-hexaose-X1 | 1.36 (0.79, 2.32) | 0.27 | . (., .) | . | 1.07 (0.72, 1.59) | 0.74 | . (., .) | . |
| Fucosyllacto-N-hexaose-X2 | 1.13 (0.56, 2.26) | 0.73 | . (., .) | . | 0.98 (0.65, 1.48) | 0.94 | . (., .) | . |
| Fucosyllacto-N-hexaose-X4 | 1.31 (0.79, 2.16) | 0.30 | 0.89 (0.35, 2.26) | 0.81 | 0.93 (0.65, 1.33) | 0.69 | 0.89 (0.50, 1.59) | 0.70 |
| Fucosyllacto-N-octaose-X1 | 1.07 (0.55, 2.07) | 0.84 | . (., .) | . | 0.97 (0.63, 1.50) | 0.91 | . (., .) | . |
| Fucosyllacto-N-octaose-X2 | 1.18 (0.70, 2.01) | 0.54 | 0.59 (0.16, 2.19) | 0.43 | 0.93 (0.63, 1.37) | 0.70 | 0.75 (0.35, 1.62) | 0.46 |
| Fucosyllacto-N-sulfate-X1 | 1.63 (0.64, 4.17) | 0.30 | . (., .) | . | 1.44 (0.81, 2.56) | 0.22 | . (., .) | . |
| Fucosylsialyllacto-N-Hexaose-X1 | 2.11 (1.01, 4.43) | 0.05 | 0.47 (0.21, 1.07) | 0.07 | 0.99 (0.60, 1.64) | 0.97 | 0.98 (0.56, 1.69) | 0.94 |
| Fucosylsialyllacto-N-Hexaose-X2 | 1.49 (0.79, 2.84) | 0.22 | 1.01 (0.41, 2.49) | 0.97 | 1.29 (0.87, 1.90) | 0.21 | 0.95 (0.52, 1.75) | 0.87 |
| **Fucosylsialyllacto-N-Hexaose-X3** | 0.95 (0.48, 1.89) | 0.88 | 1.68 (0.48, 5.81) | 0.42 | 1.08 (0.74, 1.57) | 0.70 | **3.23 (1.47, 7.09)** | **0.003** |
| Fucosylsialyllacto-N-Hexaose-X4 | 0.93 (0.52, 1.65) | 0.80 | 0.89 (0.39, 1.99) | 0.77 | 0.91 (0.62, 1.34) | 0.63 | 1.26 (0.73, 2.20) | 0.41 |
| Fucosylsialyllacto-N-Hexaose-X5 | 0.63 (0.13, 3.08) | 0.57 | 0.55 (0.23, 1.29) | 0.17 | 0.49 (0.19, 1.27) | 0.14 | 0.59 (0.35, 1.00) | 0.05 |
| Fucosylsialyllacto-N-Hexaose-X6 | 0.71 (0.30, 1.68) | 0.44 | 1.31 (0.62, 2.74) | 0.48 | 0.98 (0.63, 1.53) | 0.93 | 1.50 (0.97, 2.33) | 0.07 |
| Fucosylsialyllacto-N-neo-tetraose c | 1.20 (0.62, 2.29) | 0.59 | 1.15 (0.40, 3.30) | 0.80 | 0.82 (0.55, 1.24) | 0.35 | 0.99 (0.51, 1.91) | 0.97 |
| Fucosylsialyllacto-N-tetraose a | 1.22 (0.54, 2.73) | 0.64 | 1.89 (0.47, 7.59) | 0.37 | 0.92 (0.55, 1.53) | 0.75 | 0.76 (0.42, 1.38) | 0.37 |
| Fucosylsialyllacto-N-tetraose b | 2.17 (0.99, 4.75) | 0.05 | . (., .) | . | 1.36 (0.85, 2.17) | 0.20 | . (., .) | . |
| Fucosylsialyllacto-N-tetraose-X1 | 1.75 (0.99, 3.09) | 0.05 | 1.61 (0.70, 3.70) | 0.26 | 1.11 (0.73, 1.71) | 0.62 | 1.28 (0.73, 2.26) | 0.39 |
| Lacto-N-decaose-X1 | 1.43 (0.79, 2.57) | 0.24 | 1.52 (0.66, 3.50) | 0.33 | 1.12 (0.70, 1.78) | 0.64 | 1.38 (0.77, 2.45) | 0.28 |
| Lacto-N-difucohexaose I | 2.24 (0.97, 5.16) | 0.06 | . (., .) | . | 1.05 (0.61, 1.79) | 0.87 | . (., .) | . |
| Lacto-N-fucopentaose I | 1.82 (1.04, 3.17) | 0.04 | . (., .) | . | 1.38 (0.86, 2.21) | 0.18 | . (., .) | . |
| Lacto-N-fucopentaose II | 2.39 (0.89, 6.44) | 0.09 | 1.82 (0.43, 7.76) | 0.42 | 0.92 (0.53, 1.61) | 0.78 | 0.74 (0.43, 1.27) | 0.28 |
| Lacto-N-fucopentaose III | 1.29 (0.69, 2.41) | 0.43 | 1.32 (0.64, 2.71) | 0.45 | 1.00 (0.67, 1.47) | 0.99 | 1.29 (0.78, 2.14) | 0.32 |
| Lacto-N-fucopentaose V | 2.47 (0.74, 8.20) | 0.14 | 2.04 (0.40, 10.56) | 0.39 | 0.83 (0.53, 1.31) | 0.43 | 1.27 (0.47, 3.42) | 0.64 |
| Lacto-N-hexaose | 1.20 (0.54, 2.65) | 0.65 | 1.10 (0.61, 1.98) | 0.76 | 0.99 (0.67, 1.48) | 0.98 | 1.33 (0.83, 2.13) | 0.23 |
| Lacto-N-neo-difucohexaose I | 1.53 (0.70, 3.35) | 0.29 | . (., .) | . | 1.19 (0.70, 2.04) | 0.52 | . (., .) | . |
| Lacto-N-neo-difucohexaose II | 1.26 (0.68, 2.32) | 0.46 | 2.18 (0.92, 5.17) | 0.08 | 0.81 (0.52, 1.26) | 0.36 | 0.69 (0.38, 1.26) | 0.23 |
| Lacto-N-neo-hexaose | 1.58 (0.75, 3.36) | 0.23 | 1.18 (0.61, 2.31) | 0.62 | 1.03 (0.65, 1.63) | 0.90 | 1.45 (0.85, 2.48) | 0.18 |
| Lacto-N-neo-octaose | 1.34 (0.80, 2.25) | 0.27 | 1.28 (0.31, 5.27) | 0.73 | 1.04 (0.73, 1.50) | 0.81 | 1.35 (0.59, 3.06) | 0.48 |
| Lacto-N-tetraose | 1.77 (0.91, 3.46) | 0.09 | 0.99 (0.25, 3.86) | 0.99 | 1.04 (0.72, 1.50) | 0.83 | 1.50 (0.70, 3.19) | 0.29 |
| **Lacto-N-triose II** | 1.31 (0.59, 2.89) | 0.50 | 1.10 (0.60, 2.01) | 0.76 | 1.00 (0.69, 1.43) | 0.98 | **1.63 (1.12, 2.37)** | **0.01** |
| Lactose-3'-Sulfate | 1.29 (0.76, 2.18) | 0.35 | 0.72 (0.16, 3.32) | 0.68 | 1.12 (0.76, 1.65) | 0.57 | 1.08 (0.55, 2.12) | 0.83 |
| Sialyllacto-N-hexaose-X1 | 1.15 (0.41, 3.17) | 0.79 | 0.69 (0.33, 1.43) | 0.32 | 0.94 (0.56, 1.59) | 0.83 | 1.20 (0.71, 2.04) | 0.49 |
| Sialyllacto-N-hexaose-X2 | 0.91 (0.51, 1.62) | 0.75 | . (., .) | . | 0.88 (0.61, 1.27) | 0.50 | 1.51 (0.68, 3.37) | 0.31 |
| Sialyllacto-N-tetraose a | 1.07 (0.52, 2.19) | 0.86 | 1.11 (0.26, 4.69) | 0.88 | 1.18 (0.84, 1.67) | 0.34 | 1.43 (0.67, 3.08) | 0.36 |
| Sialyllacto-N-tetraose b | 2.13 (1.09, 4.14) | 0.03 | 1.30 (0.51, 3.33) | 0.58 | 1.04 (0.69, 1.57) | 0.84 | 1.38 (0.78, 2.42) | 0.27 |
| Sialyllacto-N-tetraose c | 1.03 (0.63, 1.69) | 0.90 | 0.76 (0.36, 1.61) | 0.474 | 0.96 (0.66, 1.39) | 0.82 | 1.15 (0.69, 1.94) | 0.59 |
| Trifucosyl(1-2,1-2,1-3)-iso-lacto-N-octa | 1.33 (0.71, 2.51) | 0.37 | . (., .) | . | 1.06 (0.73, 1.55) | 0.75 | . (., .) | . |
| Trifucosyllacto-N-hexaose I | 1.41 (0.73, 2.71) | 0.31 | . (., .) | . | 0.94 (0.59, 1.49) | 0.78 | . (., .) | . |
| Trifucosyllacto-N-hexaose-X1 | 2.61 (1.16, 5.86) | 0.02 | . (., .) | . | 1.25 (0.74, 2.13) | 0.40 | . (., .) | . |
| Trifucosyllacto-N-octaose-X1 | 1.40 (0.71, 2.73) | 0.33 | . (., .) | . | 0.97 (0.63, 1.49) | 0.90 | . (., .) | . |
| Trifucosyllacto-N-tetraose-X5 | 1.36 (0.80, 2.30) | 0.26 | . (., .) | . | 1.07 (0.73, 1.56) | 0.74 | . (., .) | . |
| a-Heptasaccharide | 1.05 (0.58, 1.89) | 0.87 | . (., .) | . | 1.06 (0.76, 1.48) | 0.74 | . (., .) | . |
| a-Pentasaccharide | 1.21 (0.65, 2.25) | 0.55 | . (., .) | . | 1.08 (0.76, 1.53) | 0.68 | . (., .) | . |
| a-Tetrasaccharide | 1.06 (0.53, 2.16) | 0.86 | . (., .) | . | 1.00 (0.69, 1.46) | 1.00 | . (., .) | . |

Associations determined by modified Poisson regression. Models adjusted for batch, child sex, duration of exclusive breastfeeding (weeks) and gestation age (weeks). Bold denotes statistically significant at nominal level (p<0.05). *Denotes statistically significant after correction for multiple testing (FDR<0.010). RR: Risk Ratio; CI: Confidence Interval; OM: Otitis Media; HMO: Human milk oligosaccharides

**Table S21**: Adjusted associations between human milk oligosaccharides measured at 6 months of lactation with lower respiratory tract infections (LRTI) in the first or second year of life stratified by secretor status in the Ulm SPATZ Health Study.

|  | **LRTI at 1 year** | | | | **LRTI at 2 years** | | | |
| --- | --- | --- | --- | --- | --- | --- | --- | --- |
|  | Secretor milk | | Non-secretor milk | | Secretor milk | | Non-secretor milk | |
| **HMO Structure** | RR (95% CI) | p | RR (95% CI) | p | RR (95% CI) | p | RR (95% CI) | p |
| 2,3 Difucosyllactose | 0.53 (0.17, 1.60) | 0.26 | . (., .) | . | 0.52 (0.25, 1.06) | 0.07 | . (., .) | . |
| 2-Fucosyllactose | 0.78 (0.27, 2.20) | 0.63 | . (., .) | . | 1.26 (0.75, 2.12) | 0.39 | . (., .) | . |
| **3'-Sialyl-3-fucosyllactose** | 0.84 (0.38, 1.87) | 0.68 | 1.22 (0.70, 2.13) | 0.48 | **0.57 (0.35, 0.92)** | **0.02** | 1.24 (0.86, 1.78) | 0.24 |
| **3-Fucosyllactose** | 0.62 (0.32, 1.21) | 0.16 | 1.03 (0.62, 1.73) | 0.91 | **0.52 (0.34, 0.81)** | **0.003** | 1.04 (0.71, 1.54) | 0.83 |
| 3-Galactosyllactose | 1.46 (0.86, 2.49) | 0.17 | 0.94 (0.64, 1.39) | 0.76 | 1.08 (0.74, 1.57) | 0.68 | 0.90 (0.67, 1.20) | 0.46 |
| 3-sialyllactose | 1.19 (0.72, 1.96) | 0.50 | 1.20 (0.73, 1.97) | 0.46 | 1.09 (0.76, 1.57) | 0.64 | 1.28 (0.93, 1.77) | 0.12 |
| 6'-Sialyl-N-acetyllactosamine | 1.30 (0.76, 2.22) | 0.34 | 1.18 (0.86, 1.64) | 0.31 | 1.12 (0.78, 1.62) | 0.53 | 1.08 (0.84, 1.40) | 0.55 |
| 6-Galactosyllactose | 0.82 (0.51, 1.32) | 0.41 | 0.78 (0.57, 1.08) | 0.14 | 0.78 (0.57, 1.07) | 0.13 | 0.82 (0.63, 1.06) | 0.13 |
| 6-sialyllactose | 1.00 (0.60, 1.67) | 0.99 | 1.20 (0.83, 1.72) | 0.33 | 1.11 (0.80, 1.52) | 0.54 | 1.04 (0.79, 1.38) | 0.77 |
| **Difucosyl-lacto-N-hexaose I** | 1.46 (0.76, 2.78) | 0.25 | . (., .) | . | **1.61 (1.07, 2.43)** | **0.02** | . (., .) | . |
| Difucosyl-lacto-N-hexaose II | 1.41 (0.57, 3.46) | 0.46 | 0.96 (0.62, 1.50) | 0.87 | 0.82 (0.47, 1.41) | 0.47 | 1.03 (0.73, 1.46) | 0.85 |
| Difucosyl-lacto-N-hexaose-X1 | 1.69 (0.92, 3.12) | 0.09 | . (., .) | . | 1.57 (1.06, 2.32) | 0.02 | . (., .) | . |
| Difucosyl-lacto-N-hexaose-X2 | 1.20 (0.59, 2.45) | 0.61 | . (., .) | . | 0.87 (0.56, 1.35) | 0.52 | . (., .) | . |
| Difucosyl-lacto-N-hexaose-X3 | 0.81 (0.50, 1.31) | 0.38 | 0.91 (0.62, 1.31) | 0.60 | 0.75 (0.56, 1.02) | 0.06 | 1.01 (0.76, 1.34) | 0.96 |
| **Difucosyl-lacto-N-neo-hexaose I** | 0.62 (0.37, 1.04) | 0.07 | 0.80 (0.54, 1.18) | 0.26 | **0.69 (0.51, 0.93)** | **0.02** | 0.95 (0.69, 1.30) | 0.73 |
| Difucosyl-para-lacto-N-hexaose I | 1.42 (0.60, 3.33) | 0.43 | 0.94 (0.62, 1.41) | 0.75 | 0.79 (0.47, 1.31) | 0.35 | 1.01 (0.73, 1.41) | 0.94 |
| Difucosyldisialyllacto-N-hexaose-X1 | 1.18 (0.75, 1.85) | 0.48 | . (., .) | . | 1.08 (0.82, 1.42) | 0.59 | . (., .) | . |
| Difucosyldisialyllacto-N-hexaose-X2 | 1.12 (0.49, 2.53) | 0.79 | 1.22 (0.75, 1.99) | 0.42 | 0.81 (0.47, 1.39) | 0.44 | 1.23 (0.84, 1.80) | 0.28 |
| Difucosylsialyllacto-N-hexaose-X1 | 0.94 (0.50, 1.75) | 0.84 | . (., .) | . | 0.92 (0.60, 1.41) | 0.71 | . (., .) | . |
| Difucosylsialyllacto-N-hexaose-X2 | 0.82 (0.47, 1.42) | 0.47 | . (., .) | . | 0.84 (0.59, 1.20) | 0.34 | . (., .) | . |
| Disialyllacto-N-Hexaose-X1 | 1.28 (0.75, 2.20) | 0.36 | 0.95 (0.60, 1.49) | 0.83 | 1.21 (0.84, 1.73) | 0.31 | 1.07 (0.73, 1.56) | 0.73 |
| Disialyllacto-N-Hexaose-X2 | 1.51 (1.00, 2.29) | 0.05 | 1.02 (0.69, 1.51) | 0.90 | 1.21 (0.91, 1.61) | 0.18 | 1.01 (0.74, 1.39) | 0.94 |
| Disialyllacto-N-Hexaose-X5 | 1.03 (0.59, 1.81) | 0.91 | 1.33 (1.00, 1.77) | 0.05 | 1.08 (0.74, 1.59) | 0.68 | 1.24 (0.98, 1.56) | 0.07 |
| Disialyllacto-N-tetraose | 1.44 (0.91, 2.28) | 0.12 | 1.04 (0.64, 1.69) | 0.87 | 1.31 (0.97, 1.76) | 0.08 | 0.90 (0.61, 1.33) | 0.60 |
| **Fucosyl(1-3)-iso-lacto-N-octaose** | **2.15 (1.43, 3.25)** | **<0.001*** | 0.92 (0.64, 1.33) | 0.66 | **1.59 (1.15, 2.20)** | **0.01** | 0.95 (0.70, 1.28) | 0.72 |
| **Fucosyl-para-lacto-N-hexaose I** | **1.80 (1.20, 2.71)** | **0.01** | 1.05 (0.72, 1.54) | 0.79 | 1.31 (0.98, 1.76) | 0.06 | 1.05 (0.78, 1.41) | 0.75 |
| Fucosyldiasialyllacto-N-hexaose-X1 | 1.12 (0.62, 2.02) | 0.70 | 1.10 (0.71, 1.69) | 0.67 | 1.21 (0.84, 1.74) | 0.31 | 1.00 (0.68, 1.46) | 0.99 |
| Fucosyldiasialyllacto-N-hexaose-X2 | 1.27 (0.77, 2.08) | 0.35 | 0.90 (0.58, 1.39) | 0.63 | 1.13 (0.81, 1.59) | 0.47 | 0.92 (0.65, 1.32) | 0.65 |
| Fucosyldisialyllacto-N-tetraose-X2 | 1.22 (0.55, 2.71) | 0.63 | 1.17 (0.73, 1.89) | 0.51 | 0.73 (0.45, 1.19) | 0.21 | 1.07 (0.74, 1.54) | 0.73 |
| **Fucosyllacto-N-hexaose II** | **1.78 (1.02, 3.11)** | **0.04** | 1.01 (0.68, 1.50) | 0.95 | 1.33 (0.92, 1.92) | 0.13 | 0.96 (0.70, 1.33) | 0.82 |
| Fucosyllacto-N-hexaose-X1 | 1.50 (0.94, 2.39) | 0.09 | . (., .) | . | 1.22 (0.88, 1.69) | 0.24 | . (., .) | . |
| Fucosyllacto-N-hexaose-X2 | 1.50 (0.93, 2.39) | 0.09 | . (., .) | . | 1.28 (0.94, 1.74) | 0.11 | . (., .) | . |
| Fucosyllacto-N-hexaose-X4 | 1.18 (0.74, 1.87) | 0.48 | 0.90 (0.60, 1.37) | 0.64 | 0.98 (0.73, 1.30) | 0.87 | 0.98 (0.71, 1.35) | 0.89 |
| Fucosyllacto-N-octaose-X1 | 1.06 (0.61, 1.85) | 0.82 | . (., .) | . | 1.10 (0.82, 1.48) | 0.52 | . (., .) | . |
| Fucosyllacto-N-octaose-X2 | 1.34 (0.85, 2.12) | 0.20 | 0.96 (0.60, 1.55) | 0.88 | 0.95 (0.67, 1.34) | 0.77 | 0.88 (0.58, 1.33) | 0.53 |
| Fucosyllacto-N-sulfate-X1 | 1.79 (0.93, 3.44) | 0.08 | . (., .) | . | 1.22 (0.74, 2.02) | 0.43 | . (., .) | . |
| Fucosylsialyllacto-N-Hexaose-X1 | 1.88 (0.94, 3.75) | 0.07 | 1.08 (0.67, 1.74) | 0.76 | 1.21 (0.75, 1.97) | 0.44 | 1.07 (0.77, 1.49) | 0.67 |
| Fucosylsialyllacto-N-Hexaose-X2 | 1.24 (0.67, 2.27) | 0.49 | 0.86 (0.55, 1.35) | 0.53 | 1.41 (0.98, 2.02) | 0.06 | 0.77 (0.53, 1.12) | 0.18 |
| **Fucosylsialyllacto-N-Hexaose-X3** | 1.08 (0.62, 1.87) | 0.79 | 0.85 (0.46, 1.59) | 0.62 | **1.38 (1.00, 1.92)** | **0.05** | 0.85 (0.50, 1.43) | 0.54 |
| Fucosylsialyllacto-N-Hexaose-X4 | 0.73 (0.46, 1.15) | 0.18 | 1.22 (0.88, 1.69) | 0.22 | 0.87 (0.62, 1.20) | 0.40 | 1.12 (0.86, 1.46) | 0.40 |
| Fucosylsialyllacto-N-Hexaose-X5 | 1.65 (0.63, 4.30) | 0.30 | 1.22 (0.82, 1.83) | 0.33 | 1.07 (0.50, 2.31) | 0.86 | 1.15 (0.84, 1.57) | 0.37 |
| Fucosylsialyllacto-N-Hexaose-X6 | 0.76 (0.40, 1.43) | 0.40 | 0.93 (0.65, 1.33) | 0.71 | 0.80 (0.50, 1.27) | 0.34 | 0.96 (0.73, 1.26) | 0.76 |
| Fucosylsialyllacto-N-neo-tetraose c | 0.97 (0.55, 1.72) | 0.92 | 1.31 (0.87, 1.99) | 0.20 | 0.71 (0.50, 1.02) | 0.06 | 1.15 (0.84, 1.58) | 0.39 |
| Fucosylsialyllacto-N-tetraose a | 1.09 (0.45, 2.63) | 0.86 | 0.92 (0.63, 1.34) | 0.66 | 0.84 (0.47, 1.49) | 0.55 | 0.87 (0.64, 1.20) | 0.40 |
| **Fucosylsialyllacto-N-tetraose b** | **2.13 (1.05, 4.31)** | **0.04** | . (., .) | . | 1.36 (0.84, 2.21) | 0.22 | . (., .) | . |
| Fucosylsialyllacto-N-tetraose-X1 | 1.34 (0.67, 2.69) | 0.41 | 1.01 (0.60, 1.69) | 0.97 | 0.97 (0.59, 1.60) | 0.92 | 0.96 (0.66, 1.41) | 0.84 |
| Lacto-N-decaose-X1 | 1.48 (0.84, 2.59) | 0.17 | 1.13 (0.76, 1.69) | 0.55 | 1.27 (0.85, 1.91) | 0.25 | 1.02 (0.71, 1.46) | 0.92 |
| Lacto-N-difucohexaose I | 1.38 (0.63, 3.05) | 0.42 | . (., .) | . | 0.85 (0.51, 1.41) | 0.53 | . (., .) | . |
| **Lacto-N-fucopentaose I** | **2.46 (1.33, 4.55)** | **0.004** | . (., .) | . | **1.90 (1.24, 2.93)** | **0.003** | . (., .) | . |
| Lacto-N-fucopentaose II | 1.98 (0.67, 5.87) | 0.22 | 0.89 (0.59, 1.36) | 0.60 | 0.91 (0.49, 1.69) | 0.76 | 0.93 (0.66, 1.30) | 0.66 |
| Lacto-N-fucopentaose III | 0.91 (0.54, 1.53) | 0.72 | 0.86 (0.60, 1.24) | 0.42 | 0.82 (0.60, 1.12) | 0.22 | 0.95 (0.71, 1.29) | 0.75 |
| Lacto-N-fucopentaose V | 2.25 (0.78, 6.51) | 0.13 | 0.97 (0.58, 1.64) | 0.92 | 0.96 (0.56, 1.63) | 0.88 | 0.94 (0.60, 1.47) | 0.79 |
| **Lacto-N-hexaose** | **2.03 (1.25, 3.29)** | **0.004** | 1.13 (0.79, 1.60) | 0.51 | **1.51 (1.06, 2.15)** | **0.02** | 0.99 (0.74, 1.32) | 0.94 |
| Lacto-N-hexaose neo | 1.58 (0.98, 2.55) | 0.06 | 1.05 (0.66, 1.65) | 0.85 | 1.23 (0.87, 1.73) | 0.24 | 1.05 (0.73, 1.52) | 0.79 |
| Lacto-N-neo-difucohexaose I | 1.11 (0.56, 2.17) | 0.77 | . (., .) | . | 1.04 (0.67, 1.61) | 0.87 | . (., .) | . |
| **Lacto-N-neo-difucohexaose II** | 0.67 (0.34, 1.35) | 0.27 | 0.96 (0.55, 1.66) | 0.88 | 0.51 (0.35, 0.72) | **<0.001*** | 1.05 (0.71, 1.57) | 0.80 |
| Lacto-N-neo-octaose | 1.25 (0.75, 2.07) | 0.39 | 0.64 (0.29, 1.43) | 0.27 | 1.10 (0.78, 1.54) | 0.588 | 0.76 (0.41, 1.43) | 0.40 |
| Lacto-N-tetraose | 2.30 (1.59, 3.31) | **<0.001*** | 0.92 (0.62, 1.36) | 0.67 | **1.46 (1.09, 1.96)** | **0.011** | 0.87 (0.64, 1.20) | 0.40 |
| **Lacto-N-triose II** | **3.28 (1.25, 8.61)** | **0.02** | 1.47 (1.06, 2.03) | 0.02 | 1.52 (1.08, 2.16) | 0.017 | 1.14 (0.89, 1.46) | 0.30 |
| Lactose-3'-Sulfate | 1.13 (0.62, 2.06) | 0.70 | 0.73 (0.49, 1.10) | 0.13 | 1.02 (0.69, 1.50) | 0.934 | 0.81 (0.59, 1.11) | 0.19 |
| Sialyllacto-N-hexaose-X1 | 1.41 (0.76, 2.62) | 0.27 | 1.13 (0.79, 1.64) | 0.50 | 1.13 (0.70, 1.82) | 0.624 | 1.03 (0.78, 1.37) | 0.83 |
| Sialyllacto-N-hexaose-X2 | 1.08 (0.62, 1.87) | 0.79 | . (., .) | . | 1.04 (0.75, 1.45) | 0.807 | . (., .) | . |
| **Sialyllacto-N-tetraose a** | **1.62 (1.15, 2.27)** | **0.01** | 0.92 (0.60, 1.41) | 0.70 | 1.67 (1.33, 2.09) | **<0.001*** | 0.78 (0.59, 1.04) | 0.09 |
| **Sialyllacto-N-tetraose b** | **1.99 (1.25, 3.18)** | **0.004** | 0.97 (0.64, 1.48) | 0.89 | 1.21 (0.85, 1.71) | 0.29 | 0.96 (0.69, 1.33) | 0.81 |
| Sialyllacto-N-tetraose c | 1.13 (0.74, 1.72) | 0.57 | 1.17 (0.85, 1.62) | 0.33 | 1.07 (0.78, 1.46) | 0.67 | 1.07 (0.83, 1.39) | 0.60 |
| **Trifucosyl(1-2,1-2,1-3)-iso-lacto-N-octa** | 1.58 (0.97, 2.59) | 0.07 | . (., .) | . | **1.50 (1.06, 2.13)** | **0.02** | . (., .) | . |
| Trifucosyllacto-N-hexaose I | 0.98 (0.53, 1.83) | 0.96 | . (., .) | . | 0.82 (0.53, 1.27) | 0.37 | . (., .) | . |
| Trifucosyllacto-N-hexaose-X1 | 1.29 (0.66, 2.50) | 0.46 | . (., .) | . | 0.81 (0.52, 1.26) | 0.35 | . (., .) | . |
| **Trifucosyllacto-N-octaose-X1** | **2.22 (1.25, 3.94)** | **0.01** | . (., .) | . | 1.35 (0.94, 1.92) | 0.10 | . (., .) | . |
| Trifucosyllacto-N-tetraose-X5 | 1.02 (0.62, 1.68) | 0.93 | . (., .) | . | 0.88 (0.61, 1.29) | 0.53 | . (., .) | . |
| a-Heptasaccharide | 1.08 (0.68, 1.72) | 0.73 | . (., .) | . | 1.04 (0.78, 1.40) | 0.77 | . (., .) | . |
| a-Pentasaccharide | 1.19 (0.77, 1.84) | 0.43 | . (., .) | . | 1.10 (0.83, 1.46) | 0.51 | . (., .) | . |
| a-Tetrasaccharide | 1.41 (0.87, 2.29) | 0.16 | . (., .) | . | 1.22 (0.90, 1.66) | 0.21 | . (., .) | . |

Associations determined by modified Poisson regression. Models adjusted for batch, child sex, duration of exclusive breastfeeding (weeks) and gestation age (weeks). Bold denotes statistically significant at nominal level (p<0.05). *Denotes statistically significant after correction for multiple testing (FDR<0.010). RR: Risk Ratio; CI: Confidence Interval; LRTI: Lower respiratory tract infections; HMO: Human milk oligosaccharides

**Table S22**: Adjusted associations between human milk oligosaccharides measured at 6 months of lactation with upper respiratory tract infections (URTI) in the first or second year of life stratified by secretor status in the Ulm SPATZ Health Study.

|  | **URTI at 1 year** | | | | **URTI at 2 years** | | | |
| --- | --- | --- | --- | --- | --- | --- | --- | --- |
|  | Secretor milk | | Non-secretor milk | | Secretor milk | | Non-secretor milk | |
| **HMO Structure** | RR (95% CI) | p | RR (95% CI) | p | RR (95% CI) | p | RR (95% CI) | p |
| 2,3 Difucosyllactose | 0.98 (0.72, 1.32) | 0.87 | . (., .) | . | 0.94 (0.76, 1.18) | 0.62 | . (., .) | . |
| 2-Fucosyllactose | 0.85 (0.62, 1.17) | 0.31 | . (., .) | . | 0.84 (0.67, 1.07) | 0.16 | . (., .) | . |
| **3'-Sialyl-3-fucosyllactose** | 1.24 (0.94, 1.63) | 0.14 | **1.27 (1.01, 1.61)** | **0.04** | 1.12 (0.90, 1.39) | 0.30 | 1.08 (0.96, 1.23) | 0.20 |
| 3-Fucosyllactose | 1.08 (0.84, 1.39) | 0.55 | 0.97 (0.80, 1.17) | 0.75 | 1.07 (0.86, 1.32) | 0.54 | 0.96 (0.86, 1.08) | 0.52 |
| 3-Galactosyllactose | 1.12 (0.94, 1.33) | 0.19 | 1.17 (1.00, 1.37) | 0.05 | 1.06 (0.95, 1.19) | 0.31 | 1.01 (0.94, 1.07) | 0.87 |
| **3-sialyllactose** | 1.07 (0.95, 1.22) | 0.28 | **1.32 (1.07, 1.62)** | **0.01** | 0.99 (0.92, 1.08) | 0.89 | 1.14 (0.99, 1.32) | 0.06 |
| **6'-Sialyl-N-acetyllactosamine** | 1.06 (0.87, 1.28) | 0.57 | **1.23 (1.05, 1.44)** | **0.01** | 0.98 (0.86, 1.12) | 0.77 | 1.09 (0.98, 1.21) | 0.12 |
| 6-Galactosyllactose | 0.96 (0.77, 1.20) | 0.74 | 1.09 (0.92, 1.30) | 0.31 | 0.97 (0.83, 1.12) | 0.64 | 1.05 (0.93, 1.18) | 0.44 |
| **6-sialyllactose** | 1.04 (0.88, 1.22) | 0.66 | 1.26 (0.99, 1.62) | 0.06 | 0.96 (0.85, 1.09) | 0.52 | **1.25 (1.08, 1.46)** | **0.004** |
| Difucosyl-lacto-N-hexaose I | 0.97 (0.79, 1.18) | 0.73 | . (., .) | . | 0.95 (0.81, 1.10) | 0.47 | . (., .) | . |
| Difucosyl-lacto-N-hexaose II | 1.31 (0.92, 1.86) | 0.13 | 0.98 (0.83, 1.15) | 0.77 | 1.15 (0.90, 1.48) | 0.26 | 1.01 (0.94, 1.10) | 0.73 |
| Difucosyl-lacto-N-hexaose-X1 | 1.02 (0.79, 1.30) | 0.91 | . (., .) | . | 1.00 (0.82, 1.22) | 0.98 | . (., .) | . |
| Difucosyl-lacto-N-hexaose-X2 | 1.10 (0.88, 1.38) | 0.40 | . (., .) | . | 1.06 (0.87, 1.30) | 0.54 | . (., .) | . |
| **Difucosyl-lacto-N-hexaose-X3** | **1.27 (1.04, 1.54)** | **0.02** | **1.27 (1.03, 1.57)** | **0.03** | 1.11 (0.97, 1.28) | 0.13 | 1.10 (0.97, 1.24) | 0.14 |
| Difucosyl-lacto-N-neo-hexaose I | 1.17 (0.92, 1.50) | 0.21 | 1.08 (0.87, 1.34) | 0.49 | 1.14 (0.95, 1.37) | 0.17 | 1.04 (0.91, 1.18) | 0.60 |
| Difucosyl-para-lacto-N-hexaose I | 1.40 (1.05, 1.87) | 0.02 | 1.16 (0.92, 1.46) | 0.21 | 1.22 (0.99, 1.51) | 0.07 | 1.07 (0.95, 1.20) | 0.30 |
| Difucosyldisialyllacto-N-hexaose-X1 | 1.08 (0.87, 1.33) | 0.51 | . (., .) | . | 0.94 (0.78, 1.13) | 0.50 | . (., .) | . |
| Difucosyldisialyllacto-N-hexaose-X2 | 1.18 (0.83, 1.70) | 0.36 | 1.03 (0.84, 1.26) | 0.80 | 0.96 (0.74, 1.24) | 0.75 | 1.03 (0.92, 1.14) | 0.64 |
| Difucosylsialyllacto-N-hexaose-X1 | 1.08 (0.84, 1.41) | 0.54 | . (., .) | . | 0.94 (0.81, 1.09) | 0.42 | . (., .) | . |
| Difucosylsialyllacto-N-hexaose-X2 | 0.92 (0.75, 1.12) | 0.40 | . (., .) | . | 0.92 (0.78, 1.07) | 0.27 | . (., .) | . |
| Disialyllacto-N-Hexaose-X1 | 1.09 (0.90, 1.32) | 0.36 | 1.05 (0.80, 1.37) | 0.75 | 0.99 (0.86, 1.14) | 0.88 | 1.04 (0.84, 1.29) | 0.72 |
| **Disialyllacto-N-Hexaose-X2** | 1.15 (0.95, 1.38) | 0.15 | **1.22 (1.01, 1.47)** | **0.04** | 0.98 (0.86, 1.11) | 0.74 | 1.08 (0.97, 1.20) | 0.16 |
| Disialyllacto-N-Hexaose-X5 | 1.08 (0.88, 1.34) | 0.46 | 1.22 (1.00, 1.49) | 0.05 | 0.99 (0.85, 1.15) | 0.87 | **1.14 (1.03, 1.26)** | **0.02** |
| **Disialyllacto-N-tetraose** | 1.09 (0.87, 1.37) | 0.45 | **1.31 (1.03, 1.66)** | **0.03** | 0.92 (0.79, 1.08) | 0.32 | **1.19 (1.01, 1.40)** | **0.04** |
| Fucosyl(1-3)-iso-lacto-N-octaose | 1.07 (0.91, 1.26) | 0.39 | 1.04 (0.84, 1.28) | 0.72 | 1.03 (0.93, 1.14) | 0.63 | 1.08 (0.94, 1.24) | 0.26 |
| Fucosyl-para-lacto-N-hexaose I | 1.15 (0.94, 1.41) | 0.18 | 1.13 (0.95, 1.35) | 0.17 | 1.07 (0.91, 1.25) | 0.44 | 1.09 (0.97, 1.24) | 0.15 |
| Fucosyldiasialyllacto-N-hexaose-X1 | 1.10 (0.90, 1.35) | 0.37 | 0.93 (0.69, 1.24) | 0.62 | 0.96 (0.83, 1.11) | 0.61 | 1.04 (0.92, 1.18) | 0.56 |
| **Fucosyldiasialyllacto-N-hexaose-X2** | 1.24 (0.96, 1.61) | 0.09 | **1.36 (1.10, 1.68)** | **0.004** | 1.00 (0.85, 1.18) | 0.99 | **1.19 (1.03, 1.38)** | **0.02** |
| **Fucosyldisialyllacto-N-tetraose-X2** | 1.30 (0.96, 1.77) | **0.09** | **1.31 (1.05, 1.63)** | **0.02** | 1.11 (0.88, 1.39) | 0.40 | 1.08 (0.96, 1.22) | 0.18 |
| Fucosyllacto-N-hexaose II | 1.07 (0.87, 1.33) | 0.51 | 0.95 (0.76, 1.18) | 0.61 | 1.02 (0.89, 1.17) | 0.74 | 1.03 (0.92, 1.15) | 0.57 |
| Fucosyllacto-N-hexaose-X1 | 1.04 (0.82, 1.32) | 0.76 | . (., .) | . | 0.95 (0.80, 1.13) | 0.58 | . (., .) | . |
| Fucosyllacto-N-hexaose-X2 | 1.07 (0.89, 1.29) | 0.48 | . (., .) | . | 0.95 (0.82, 1.09) | 0.45 | . (., .) | . |
| **Fucosyllacto-N-hexaose-X4** | **1.21 (1.01, 1.45)** | **0.04** | **1.26 (1.01, 1.57)** | **0.04** | 1.08 (0.97, 1.21) | 0.16 | 1.04 (0.90, 1.22) | 0.58 |
| Fucosyllacto-N-octaose-X1 | 1.04 (0.87, 1.25) | 0.65 | . (., .) | . | 0.94 (0.79, 1.11) | 0.46 | . (., .) | . |
| Fucosyllacto-N-octaose-X2 | 1.12 (0.97, 1.30) | 0.13 | 1.00 (0.76, 1.31) | 0.99 | 1.08 (0.97, 1.19) | 0.14 | 0.94 (0.79, 1.14) | 0.55 |
| Fucosyllacto-N-sulfate-X1 | 1.14 (0.90, 1.44) | 0.29 | . (., .) | . | 1.08 (0.95, 1.23) | 0.25 | . (., .) | . |
| Fucosylsialyllacto-N-Hexaose-X1 | 1.12 (0.88, 1.44) | 0.36 | 1.10 (0.88, 1.38) | 0.40 | 0.96 (0.79, 1.16) | 0.66 | 1.11 (0.98, 1.25) | 0.09 |
| Fucosylsialyllacto-N-Hexaose-X2 | 1.01 (0.82, 1.26) | 0.90 | 0.67 (0.44, 1.01) | 0.05 | 0.94 (0.79, 1.12) | 0.49 | 0.90 (0.76, 1.08) | 0.26 |
| **Fucosylsialyllacto-N-Hexaose-X3** | 0.90 (0.75, 1.07) | 0.22 | 1.23 (0.99, 1.54) | 0.07 | 0.88 (0.78, 1.01) | 0.06 | **1.16 (1.03, 1.30)** | **0.02** |
| **Fucosylsialyllacto-N-Hexaose-X4** | 1.16 (0.96, 1.39) | 0.12 | **1.27 (1.01, 1.60)** | **0.04** | 1.01 (0.89, 1.15) | 0.83 | **1.19 (1.04, 1.35)** | **0.01** |
| **Fucosylsialyllacto-N-Hexaose-X5** | 1.21 (0.92, 1.60) | 0.17 | 1.12 (0.90, 1.39) | 0.32 | 0.99 (0.77, 1.26) | 0.92 | **1.15 (1.02, 1.30)** | **0.02** |
| **Fucosylsialyllacto-N-Hexaose-X6** | 1.13 (0.94, 1.36) | 0.20 | **1.31 (1.10, 1.57)** | **0.003** | 1.06 (0.93, 1.19) | 0.38 | **1.15 (1.04, 1.27)** | **0.01** |
| **Fucosylsialyllacto-N-neo-tetraose c** | **1.44 (1.16, 1.77)** | **0.001** | 1.27 (1.00, 1.62) | 0.05 | 1.14 (0.98, 1.32) | 0.08 | **1.16 (1.03, 1.31)** | **0.02** |
| Fucosylsialyllacto-N-tetraose a | 1.12 (0.83, 1.53) | 0.46 | 0.98 (0.83, 1.16) | 0.80 | 1.08 (0.86, 1.37) | 0.51 | 1.03 (0.95, 1.11) | 0.50 |
| Fucosylsialyllacto-N-tetraose b | 1.08 (0.77, 1.51) | 0.66 | . (., .) | . | 0.95 (0.74, 1.21) | 0.67 | . (., .) | . |
| **Fucosylsialyllacto-N-tetraose-X1** | 1.39 (1.12, 1.73) | 0.00 | **1.43 (1.17, 1.74)** | **<0.001** | **1.17 (1.01, 1.36)** | **0.04** | 1.13 (0.99, 1.29) | 0.07 |
| Lacto-N-decaose-X1 | 1.09 (0.91, 1.31) | 0.35 | 0.88 (0.68, 1.13) | 0.32 | 0.99 (0.84, 1.18) | 0.95 | 0.93 (0.80, 1.08) | 0.36 |
| Lacto-N-difucohexaose I | 1.14 (0.87, 1.50) | 0.35 | . (., .) | . | 1.10 (0.88, 1.37) | 0.41 | . (., .) | . |
| Lacto-N-fucopentaose I | 0.94 (0.68, 1.29) | 0.71 | . (., .) | . | 0.87 (0.67, 1.12) | 0.27 | . (., .) | . |
| Lacto-N-fucopentaose II | 1.35 (0.96, 1.92) | 0.09 | 1.08 (0.86, 1.35) | 0.51 | 1.19 (0.93, 1.53) | 0.17 | 1.03 (0.90, 1.18) | 0.65 |
| **Lacto-N-fucopentaose III** | **1.24 (1.01, 1.52)** | **0.04** | 1.18 (0.95, 1.46) | 0.14 | 1.12 (0.96, 1.30) | 0.17 | 1.07 (0.94, 1.21) | 0.30 |
| Lacto-N-fucopentaose V | 1.31 (0.93, 1.85) | 0.12 | 1.17 (0.88, 1.56) | 0.27 | 1.20 (0.92, 1.55) | 0.18 | 1.12 (0.90, 1.40) | 0.33 |
| Lacto-N-hexaose | 1.09 (0.91, 1.31) | 0.34 | 1.01 (0.82, 1.24) | 0.93 | 1.01 (0.89, 1.15) | 0.92 | 0.99 (0.85, 1.16) | 0.94 |
| Lacto-N-neo-difucohexaose I | 1.23 (0.90, 1.66) | 0.19 | . (., .) | . | 1.06 (0.88, 1.29) | 0.53 | . (., .) | . |
| **Lacto-N-neo-difucohexaose II** | **1.36 (1.01, 1.83)** | **0.04** | 1.16 (0.90, 1.51) | 0.25 | 1.20 (0.96, 1.51) | 0.11 | 1.04 (0.93, 1.15) | 0.52 |
| Lacto-N-neo-hexaose | 1.10 (0.89, 1.36) | 0.39 | 1.16 (0.90, 1.50) | 0.25 | 1.07 (0.91, 1.25) | 0.41 | 1.02 (0.85, 1.23) | 0.83 |
| Lacto-N-neo-octaose | 1.14 (0.98, 1.33) | 0.09 | 1.08 (0.86, 1.35) | 0.53 | 1.07 (0.96, 1.19) | 0.23 | 1.09 (0.99, 1.19) | 0.08 |
| Lacto-N-tetraose | 1.12 (0.92, 1.36) | 0.25 | 1.12 (0.90, 1.41) | 0.31 | 1.05 (0.90, 1.23) | 0.54 | 1.11 (0.94, 1.31) | 0.24 |
| Lacto-N-triose II | 1.15 (0.93, 1.43) | 0.19 | 1.13 (0.96, 1.33) | 0.15 | 1.00 (0.88, 1.14) | 0.98 | 1.07 (0.96, 1.20) | 0.23 |
| Lactose-3'-Sulfate | 1.10 (0.87, 1.40) | 0.43 | 1.24 (0.98, 1.56) | 0.07 | 1.01 (0.86, 1.20) | 0.89 | 1.07 (0.96, 1.19) | 0.23 |
| **Sialyllacto-N-hexaose-X1** | 1.10 (0.90, 1.33) | 0.35 | 1.05 (0.85, 1.29) | 0.66 | 0.97 (0.83, 1.13) | 0.72 | **1.15 (1.02, 1.29)** | **0.02** |
| Sialyllacto-N-hexaose-X2 | 1.04 (0.86, 1.26) | 0.71 | 1.05 (0.78, 1.42) | 0.76 | 0.98 (0.85, 1.12) | 0.76 | 1.07 (0.95, 1.21) | 0.27 |
| Sialyllacto-N-tetraose a | 0.98 (0.83, 1.17) | 0.85 | 0.99 (0.77, 1.26) | 0.92 | 0.99 (0.87, 1.12) | 0.89 | 1.04 (0.90, 1.21) | 0.58 |
| **Sialyllacto-N-tetraose b** | 1.15 (0.91, 1.45) | 0.24 | **1.32 (1.07, 1.62)** | **0.01** | 1.00 (0.85, 1.18) | 0.97 | 1.15 (0.98, 1.35) | 0.09 |
| **Sialyllacto-N-tetraose c** | 1.22 (1.04, 1.43) | 0.01 | 1.22 (1.00, 1.50) | 0.05 | 1.03 (0.94, 1.13) | 0.54 | **1.17 (1.04, 1.31)** | **0.01** |
| Trifucosyl(1-2,1-2,1-3)-iso-lacto-N-octa | 1.03 (0.85, 1.24) | 0.77 | . (., .) | . | 0.92 (0.80, 1.06) | 0.24 | . (., .) | . |
| Trifucosyllacto-N-hexaose I | 1.05 (0.81, 1.36) | 0.71 | . (., .) | . | 1.02 (0.81, 1.28) | 0.89 | . (., .) | . |
| Trifucosyllacto-N-hexaose-X1 | 1.20 (0.93, 1.56) | 0.16 | . (., .) | . | 1.16 (0.96, 1.40) | 0.12 | . (., .) | . |
| Trifucosyllacto-N-octaose-X1 | 1.14 (0.96, 1.37) | 0.14 | . (., .) | . | 1.06 (0.92, 1.22) | 0.42 | . (., .) | . |
| Trifucosyllacto-N-tetraose-X5 | 1.11 (0.89, 1.38) | 0.36 | . (., .) | . | 1.04 (0.91, 1.19) | 0.57 | . (., .) | . |
| a-Heptasaccharide | 1.12 (0.93, 1.35) | 0.24 | . (., .) | . | 1.10 (0.97, 1.26) | 0.14 | . (., .) | . |
| a-Pentasaccharide | 1.14 (0.93, 1.39) | 0.21 | . (., .) | . | 1.10 (0.95, 1.26) | 0.19 | . (., .) | . |
| a-Tetrasaccharide | 1.13 (0.94, 1.34) | 0.19 | . (., .) | . | 1.09 (0.96, 1.23) | 0.19 | . (., .) | . |

Associations determined by modified Poisson regression. Models adjusted for batch, child sex, duration of exclusive breastfeeding (weeks) and gestation age (weeks). Bold denotes statistically significant at nominal level (p<0.05). *Denotes statistically significant after correction for multiple testing (FDR<0.010). RR: Risk Ratio; CI: Confidence Interval; URTI: Upper respiratory tract infections; HMO: Human milk oligosaccharides

**Table S23**: Adjusted associations between human milk oligosaccharides measured at 6 weeks of lactation with otitis media (OM) in the first or second year of life stratified by secretor status in the Ulm SPATZ Health Study

|  | **OM at 1 year** | | | | **OM at 2 years** | | | |
| --- | --- | --- | --- | --- | --- | --- | --- | --- |
|  | Secretor milk | | Non-secretor milk | | Secretor milk | | Non-secretor milk | |
| **HMO Structure** | RR (95% CI) | p | RR (95% CI) | p | RR (95% CI) | p | RR (95% CI) | p |
| 2,3 Difucosyllactose | 0.81 (0.50, 1.32) | 0.40 | . (., .) | . | 0.77 (0.55, 1.08) | 0.13 | . (., .) | . |
| **2-Fucosyllactose** | **0.41 (0.23, 0.73)** | **0.002** | . (., .) | . | 0.80 (0.57, 1.12) | 0.20 | . (., .) | . |
| 3'-Sialyl-3-fucosyllactose | 1.13 (0.73, 1.74) | 0.59 | 0.54 (0.27, 1.08) | 0.08 | 0.93 (0.66, 1.29) | 0.65 | 0.65 (0.41, 1.03) | 0.07 |
| **3-Fucosyllactose** | 1.03 (0.61, 1.75) | 0.91 | 0.70 (0.35, 1.42) | 0.33 | 0.85 (0.61, 1.16) | 0.30 | **0.58 (0.34, 1.00)** | **0.05** |
| **3-Galactosyllactose** | 0.74 (0.45, 1.23) | 0.25 | 1.20 (0.60, 2.41) | 0.60 | 0.95 (0.67, 1.36) | 0.79 | **1.75 (1.11, 2.78)** | **0.02** |
| 3-sialyllactose | 0.98 (0.50, 1.91) | 0.94 | 0.64 (0.29, 1.39) | 0.26 | 1.05 (0.72, 1.51) | 0.81 | 0.82 (0.55, 1.23) | 0.34 |
| 6'-Sialyl-N-acetyllactosamine | 0.56 (0.33, 0.95) | 0.03 | 0.86 (0.37, 2.02) | 0.73 | 0.80 (0.55, 1.17) | 0.26 | 1.08 (0.58, 2.01) | 0.82 |
| **6-Galactosyllactose** | 0.58 (0.34, 0.99) | 0.05 | 1.74 (0.92, 3.29) | 0.09 | 0.91 (0.64, 1.30) | 0.60 | **1.99 (1.30, 3.05)** | **0.001** |
| 6-sialyllactose | 0.75 (0.43, 1.32) | 0.32 | 0.96 (0.46, 2.01) | 0.92 | 0.82 (0.58, 1.17) | 0.27 | 0.92 (0.54, 1.59) | 0.78 |
| Difucosyl-lacto-N-hexaose I | 0.70 (0.40, 1.23) | 0.22 | . (., .) | . | 1.06 (0.74, 1.51) | 0.76 | . (., .) | . |
| **Difucosyl-lacto-N-hexaose II** | 1.13 (0.67, 1.91) | 0.65 | 1.26 (0.57, 2.79) | 0.57 | 0.97 (0.72, 1.31) | 0.85 | **0.56 (0.33, 0.96)** | **0.036** |
| Difucosyl-lacto-N-hexaose-X1 | 1.20 (0.59, 2.44) | 0.62 | . (., .) | . | 1.14 (0.79, 1.64) | 0.50 | . (., .) | . |
| Difucosyl-lacto-N-hexaose-X2 | 0.84 (0.52, 1.38) | 0.50 | . (., .) | . | 0.79 (0.58, 1.08) | 0.14 | . (., .) | . |
| Difucosyl-lacto-N-hexaose-X3 | 1.05 (0.66, 1.67) | 0.84 | 0.65 (0.35, 1.20) | 0.17 | 0.82 (0.59, 1.14) | 0.23 | 1.04 (0.65, 1.67) | 0.86 |
| Difucosyl-lacto-N-neo-hexaose I | 0.94 (0.62, 1.43) | 0.78 | 1.24 (0.51, 3.05) | 0.63 | 0.96 (0.72, 1.29) | 0.79 | 0.85 (0.53, 1.37) | 0.50 |
| Difucosyl-para-lacto-N-hexaose I | 1.51 (0.85, 2.70) | 0.16 | 0.99 (0.45, 2.14) | 0.97 | 0.94 (0.69, 1.28) | 0.71 | 0.83 (0.50, 1.38) | 0.47 |
| **Difucosyldisialyllacto-N-hexaose-X1** | **0.49 (0.26, 0.93)** | **0.03** | . (., .) | . | 0.85 (0.53, 1.35) | 0.49 | . (., .) | . |
| **Difucosyldisialyllacto-N-hexaose-X2** | 1.17 (0.70, 1.95) | 0.56 | 0.51 (0.27, 0.97) | 0.04 | 1.00 (0.73, 1.35) | 0.98 | **0.45 (0.29, 0.69)** | **<0.001** |
| Difucosylsialyllacto-N-hexaose-X1 | 0.59 (0.33, 1.05) | 0.07 | . (., .) | . | 0.77 (0.50, 1.19) | 0.24 | . (., .) | . |
| Difucosylsialyllacto-N-hexaose-X2 | 0.72 (0.46, 1.13) | 0.15 | . (., .) | . | 0.80 (0.58, 1.10) | 0.17 | . (., .) | . |
| Disialyllacto-N-Hexaose-X1 | 0.81 (0.41, 1.60) | 0.55 | 1.07 (0.48, 2.36) | 0.87 | 0.94 (0.66, 1.35) | 0.75 | 1.13 (0.68, 1.88) | 0.63 |
| Disialyllacto-N-Hexaose-X2 | 1.23 (0.56, 2.70) | 0.61 | 0.80 (0.46, 1.40) | 0.44 | 0.96 (0.65, 1.40) | 0.82 | 1.04 (0.68, 1.60) | 0.86 |
| Disialyllacto-N-Hexaose-X5 | 0.57 (0.32, 1.02) | 0.06 | 1.09 (0.42, 2.83) | 0.87 | 0.71 (0.50, 1.02) | 0.06 | 1.13 (0.63, 2.01) | 0.68 |
| Disialyllacto-N-tetraose | 1.24 (0.55, 2.79) | 0.60 | 0.57 (0.26, 1.25) | 0.16 | 1.00 (0.69, 1.46) | 0.99 | 0.89 (0.53, 1.47) | 0.64 |
| Fucosyl(1-3)-iso-lacto-N-octaose | 1.33 (0.65, 2.73) | 0.44 | 1.45 (0.75, 2.80) | 0.27 | 1.19 (0.81, 1.73) | 0.37 | 1.74 (0.95, 3.19) | 0.07 |
| **Fucosyl-para-lacto-N-hexaose I** | 1.65 (0.78, 3.50) | 0.19 | 1.11 (0.76, 1.62) | 0.60 | 1.12 (0.79, 1.58) | 0.54 | **1.87 (1.21, 2.87)** | **0.005** |
| **Fucosyldiasialyllacto-N-hexaose-X1** | 0.81 (0.39, 1.65) | 0.56 | **0.51 (0.26, 0.97)** | **0.04** | 1.04 (0.71, 1.52) | 0.83 | 0.77 (0.46, 1.28) | 0.31 |
| Fucosyldiasialyllacto-N-hexaose-X2 | 1.12 (0.46, 2.69) | 0.80 | 0.52 (0.26, 1.03) | 0.06 | 0.94 (0.61, 1.45) | 0.78 | 1.06 (0.63, 1.80) | 0.82 |
| Fucosyldisialyllacto-N-tetraose-X2 | 1.25 (0.71, 2.19) | 0.44 | 0.55 (0.24, 1.26) | 0.16 | 0.94 (0.68, 1.30) | 0.72 | 0.66 (0.43, 1.03) | 0.07 |
| Fucosyllacto-N-hexaose II | 1.12 (0.57, 2.20) | 0.74 | 1.50 (0.57, 3.94) | 0.41 | 1.12 (0.77, 1.63) | 0.54 | 1.39 (0.79, 2.45) | 0.25 |
| Fucosyllacto-N-hexaose-X1 | 1.13 (0.61, 2.10) | 0.70 | . (., .) | . | 0.96 (0.68, 1.36) | 0.81 | . (., .) | . |
| Fucosyllacto-N-hexaose-X2 | 0.72 (0.39, 1.31) | 0.28 | . (., .) | . | 0.93 (0.66, 1.33) | 0.71 | . (., .) | . |
| Fucosyllacto-N-hexaose-X4 | 1.09 (0.71, 1.67) | 0.70 | 1.66 (0.88, 3.12) | 0.12 | 0.78 (0.58, 1.04) | 0.09 | 1.01 (0.56, 1.81) | 0.99 |
| Fucosyllacto-N-octaose-X1 | 0.86 (0.51, 1.47) | 0.59 | . (., .) | . | 0.94 (0.66, 1.32) | 0.72 | . (., .) | . |
| Fucosyllacto-N-octaose-X2 | 1.15 (0.68, 1.95) | 0.59 | 1.78 (0.87, 3.66) | 0.12 | 0.89 (0.67, 1.18) | 0.41 | 1.38 (0.76, 2.51) | 0.29 |
| Fucosyllacto-N-sulfate-X1 | 0.78 (0.45, 1.35) | 0.37 | . (., .) | . | 0.91 (0.64, 1.28) | 0.58 | . (., .) | . |
| Fucosylsialyllacto-N-Hexaose-X1 | 1.02 (0.50, 2.10) | 0.95 | 1.04 (0.48, 2.26) | 0.91 | 1.03 (0.71, 1.51) | 0.87 | 1.03 (0.68, 1.56) | 0.90 |
| Fucosylsialyllacto-N-Hexaose-X2 | 0.95 (0.55, 1.63) | 0.85 | 1.39 (0.76, 2.55) | 0.28 | 1.20 (0.87, 1.65) | 0.27 | 1.27 (0.72, 2.23) | 0.41 |
| Fucosylsialyllacto-N-Hexaose-X3 | 0.70 (0.39, 1.26) | 0.24 | 0.68 (0.19, 2.50) | 0.56 | 0.96 (0.67, 1.37) | 0.81 | 0.90 (0.52, 1.55) | 0.70 |
| Fucosylsialyllacto-N-Hexaose-X4 | 0.76 (0.45, 1.27) | 0.29 | 1.10 (0.52, 2.33) | 0.80 | 0.72 (0.51, 1.03) | 0.07 | 1.18 (0.74, 1.89) | 0.49 |
| Fucosylsialyllacto-N-Hexaose-X5 | 0.99 (0.56, 1.72) | 0.96 | 1.71 (0.72, 4.05) | 0.22 | 0.82 (0.61, 1.12) | 0.22 | 0.64 (0.35, 1.19) | 0.16 |
| Fucosylsialyllacto-N-Hexaose-X6 | 0.94 (0.59, 1.48) | 0.78 | 0.57 (0.28, 1.13) | 0.11 | 0.74 (0.50, 1.10) | 0.13 | 1.12 (0.68, 1.87) | 0.65 |
| Fucosylsialyllacto-N-neo-tetraose c | 0.92 (0.59, 1.44) | 0.72 | 0.83 (0.44, 1.56) | 0.56 | 0.72 (0.53, 0.98) | 0.04 | 0.76 (0.50, 1.17) | 0.21 |
| Fucosylsialyllacto-N-tetraose a | 1.31 (0.73, 2.36) | 0.36 | 1.11 (0.62, 2.01) | 0.72 | 0.98 (0.72, 1.34) | 0.91 | 0.69 (0.41, 1.17) | 0.17 |
| Fucosylsialyllacto-N-tetraose b | 1.16 (0.60, 2.22) | 0.66 | . (., .) | . | 1.02 (0.69, 1.49) | 0.94 | . (., .) | . |
| Fucosylsialyllacto-N-tetraose-X1 | 1.08 (0.56, 2.08) | 0.82 | 0.76 (0.40, 1.46) | 0.42 | 0.90 (0.63, 1.28) | 0.56 | 0.84 (0.54, 1.30) | 0.43 |
| Lacto-N-decaose-X1 | 1.08 (0.54, 2.14) | 0.83 | 1.57 (0.59, 4.17) | 0.37 | 1.01 (0.72, 1.43) | 0.94 | 1.57 (0.98, 2.52) | 0.06 |
| Lacto-N-difucohexaose I | 0.81 (0.52, 1.28) | 0.37 | . (., .) | . | 0.81 (0.58, 1.12) | 0.21 | . (., .) | . |
| Lacto-N-fucopentaose I | 1.15 (0.63, 2.11) | 0.65 | . (., .) | . | 1.13 (0.82, 1.56) | 0.45 | . (., .) | . |
| Lacto-N-fucopentaose II | 1.41 (0.76, 2.61) | 0.28 | 1.09 (0.47, 2.53) | 0.84 | 0.94 (0.69, 1.29) | 0.71 | 0.71 (0.44, 1.14) | 0.16 |
| Lacto-N-fucopentaose III | 1.00 (0.64, 1.57) | 1.00 | 0.85 (0.48, 1.52) | 0.59 | 0.89 (0.65, 1.23) | 0.48 | 1.11 (0.68, 1.83) | 0.67 |
| Lacto-N-fucopentaose V | 1.75 (0.87, 3.53) | 0.12 | 1.06 (0.53, 2.13) | 0.87 | 1.03 (0.74, 1.43) | 0.88 | 0.88 (0.55, 1.40) | 0.58 |
| Lacto-N-hexaose | 0.99 (0.58, 1.69) | 0.97 | 1.58 (0.60, 4.16) | 0.36 | 1.05 (0.76, 1.45) | 0.79 | 1.25 (0.68, 2.27) | 0.47 |
| Lacto-N-neo-difucohexaose I | 0.63 (0.38, 1.06) | 0.09 | . (., .) | . | 0.68 (0.44, 1.04) | 0.08 | . (., .) | . |
| Lacto-N-neo-difucohexaose II | 1.11 (0.71, 1.71) | 0.65 | 0.66 (0.30, 1.45) | 0.30 | 0.88 (0.66, 1.18) | 0.40 | 0.63 (0.40, 1.01) | 0.05 |
| Lacto-N-neo-hexaose | 0.88 (0.55, 1.41) | 0.59 | 1.56 (0.66, 3.66) | 0.31 | 0.81 (0.60, 1.11) | 0.19 | 1.13 (0.60, 2.13) | 0.70 |
| Lacto-N-neo-octaose | 1.08 (0.58, 2.01) | 0.81 | 1.48 (0.64, 3.42) | 0.36 | 0.98 (0.70, 1.38) | 0.91 | 1.49 (0.94, 2.39) | 0.09 |
| Lacto-N-tetraose | 1.73 (0.79, 3.79) | 0.17 | 1.14 (0.71, 1.84) | 0.58 | 1.15 (0.82, 1.61) | 0.42 | 1.46 (0.90, 2.37) | 0.13 |
| Lacto-N-triose II | 1.32 (0.71, 2.46) | 0.38 | 1.28 (0.84, 1.95) | 0.25 | 1.02 (0.68, 1.51) | 0.93 | 1.54 (0.83, 2.86) | 0.17 |
| Lactose-3'-Sulfate | 0.72 (0.39, 1.32) | 0.28 | 0.28 (0.13, 0.57) | 0.00 | 1.03 (0.70, 1.51) | 0.89 | 0.69 (0.40, 1.18) | 0.18 |
| Sialyllacto-N-hexaose-X1 | 1.01 (0.53, 1.89) | 0.99 | 1.61 (0.58, 4.43) | 0.36 | 0.97 (0.68, 1.37) | 0.86 | 1.50 (0.82, 2.73) | 0.19 |
| Sialyllacto-N-hexaose-X2 | 0.77 (0.46, 1.29) | 0.32 | 1.88 (0.86, 4.13) | 0.11 | 0.74 (0.52, 1.04) | 0.08 | 1.23 (0.69, 2.19) | 0.48 |
| Sialyllacto-N-tetraose a | 1.30 (0.59, 2.86) | 0.51 | 1.18 (0.66, 2.09) | 0.58 | 1.22 (0.81, 1.86) | 0.34 | 1.31 (0.75, 2.27) | 0.34 |
| Sialyllacto-N-tetraose b | 1.52 (0.76, 3.03) | 0.24 | 0.81 (0.40, 1.61) | 0.55 | 1.04 (0.75, 1.45) | 0.79 | 1.14 (0.74, 1.75) | 0.56 |
| Sialyllacto-N-tetraose c | 0.79 (0.50, 1.24) | 0.31 | 0.93 (0.41, 2.12) | 0.87 | 0.72 (0.48, 1.09) | 0.13 | 1.25 (0.70, 2.23) | 0.46 |
| Trifucosyl(1-2,1-2,1-3)-iso-lacto-N-octa | 0.91 (0.51, 1.62) | 0.74 | . (., .) | . | 1.08 (0.75, 1.54) | 0.69 | . (., .) | . |
| Trifucosyllacto-N-hexaose I | 0.83 (0.53, 1.30) | 0.41 | . (., .) | . | 0.94 (0.66, 1.32) | 0.70 | . (., .) | . |
| Trifucosyllacto-N-hexaose-X1 | 1.08 (0.65, 1.79) | 0.76 | . (., .) | . | 0.90 (0.64, 1.28) | 0.56 | . (., .) | . |
| Trifucosyllacto-N-octaose-X1 | 1.09 (0.62, 1.91) | 0.76 | . (., .) | . | 0.95 (0.68, 1.34) | 0.78 | . (., .) | . |
| Trifucosyllacto-N-tetraose-X5 | 0.88 (0.57, 1.37) | 0.57 | . (., .) | . | 0.87 (0.63, 1.21) | 0.41 | . (., .) | . |
| a-Heptasaccharide | 0.95 (0.55, 1.64) | 0.85 | . (., .) | . | 0.95 (0.66, 1.36) | 0.78 | . (., .) | . |
| a-Pentasaccharide | 1.06 (0.61, 1.86) | 0.83 | . (., .) | . | 1.02 (0.71, 1.46) | 0.91 | . (., .) | . |
| a-Tetrasaccharide | 0.99 (0.53, 1.85) | 0.98 | . (., .) | . | 0.94 (0.65, 1.36) | 0.74 | . (., .) | . |

Human milk oligosaccharide levels were normalised following stratification by secretor status. Associations determined by modified Poisson regression. Models adjusted for batch, child sex, duration of exclusive breastfeeding (weeks) and gestation age (weeks). Bold denotes statistically significant at nominal level (p<0.05). *Denotes statistically significant after correction for multiple testing (FDR<0.010). RR: Risk Ratio; CI: Confidence Interval; OM: Otitis Media; HMO: Human milk oligosaccharides

**Table S24**: Adjusted associations between human milk oligosaccharides measured at 6 weeks of lactation with lower respiratory tract infections (LRTI) in the first or second year of life stratified by secretor status in the Ulm SPATZ Health Study.

|  | **LRTI at 1 year** | | | | **LRTI at 2 years** | | | |
| --- | --- | --- | --- | --- | --- | --- | --- | --- |
|  | Secretor milk | | Non-secretor milk | | Secretor milk | | Non-secretor milk | |
| **HMO Structure** | RR (95% CI) | p | RR (95% CI) | p | RR (95% CI) | p | RR (95% CI) | p |
| **2,3 Difucosyllactose** | 0.72 (0.46, 1.10) | 0.13 | . (., .) | . | **0.68 (0.52, 0.90)** | **0.008** | . (., .) | . |
| 2-Fucosyllactose | 0.56 (0.32, 0.97) | 0.04 | . (., .) | . | 0.86 (0.64, 1.14) | 0.29 | . (., .) | . |
| 3'-Sialyl-3-fucosyllactose | 1.19 (0.71, 1.98) | 0.51 | 0.93 (0.66, 1.30) | 0.66 | 0.89 (0.67, 1.19) | 0.45 | 1.12 (0.88, 1.43) | 0.36 |
| 3-Fucosyllactose | 0.85 (0.53, 1.37) | 0.51 | 0.79 (0.56, 1.12) | 0.19 | 0.77 (0.59, 1.01) | 0.06 | 0.95 (0.71, 1.26) | 0.71 |
| 3-Galactosyllactose | 0.96 (0.60, 1.52) | 0.85 | 1.01 (0.75, 1.35) | 0.97 | 0.95 (0.73, 1.22) | 0.68 | 0.97 (0.78, 1.22) | 0.82 |
| 3-sialyllactose | 1.45 (0.80, 2.63) | 0.22 | 1.03 (0.74, 1.46) | 0.85 | 1.18 (0.89, 1.58) | 0.25 | 1.15 (0.89, 1.47) | 0.29 |
| 6'-Sialyl-N-acetyllactosamine | 0.86 (0.54, 1.35) | 0.50 | 1.15 (0.80, 1.64) | 0.46 | 0.96 (0.73, 1.28) | 0.79 | 1.10 (0.84, 1.44) | 0.48 |
| 6-Galactosyllactose | 0.68 (0.45, 1.04) | 0.08 | 0.98 (0.69, 1.39) | 0.91 | 0.68 (0.53, 0.87) | 0.00 | 0.99 (0.73, 1.35) | 0.96 |
| 6-sialyllactose | 0.79 (0.49, 1.25) | 0.31 | 0.98 (0.67, 1.44) | 0.93 | 0.90 (0.68, 1.19) | 0.48 | 1.07 (0.79, 1.46) | 0.65 |
| Difucosyl-lacto-N-hexaose I | 0.85 (0.52, 1.40) | 0.53 | . (., .) | . | 1.09 (0.81, 1.45) | 0.58 | . (., .) | . |
| Difucosyl-lacto-N-hexaose II | 1.07 (0.63, 1.82) | 0.80 | 0.90 (0.59, 1.38) | 0.64 | 0.85 (0.64, 1.13) | 0.26 | 0.87 (0.62, 1.23) | 0.44 |
| Difucosyl-lacto-N-hexaose-X1 | 1.08 (0.65, 1.78) | 0.77 | . (., .) | . | 1.14 (0.86, 1.50) | 0.36 | . (., .) | . |
| Difucosyl-lacto-N-hexaose-X2 | 0.99 (0.63, 1.55) | 0.95 | . (., .) | . | 0.84 (0.63, 1.11) | 0.22 | . (., .) | . |
| **Difucosyl-lacto-N-hexaose-X3** | 0.70 (0.45, 1.08) | 0.11 | 0.83 (0.59, 1.15) | 0.26 | **0.72 (0.55, 0.95)** | **0.02** | 1.01 (0.78, 1.29) | 0.97 |
| **Difucosyl-lacto-N-neo-hexaose I** | 0.66 (0.41, 1.05) | 0.08 | 0.92 (0.64, 1.33) | 0.67 | **0.69 (0.55, 0.88)** | **0.002** | 0.88 (0.66, 1.18) | 0.40 |
| Difucosyl-para-lacto-N-hexaose I | 1.29 (0.81, 2.05) | 0.28 | 0.82 (0.58, 1.15) | 0.25 | 0.94 (0.71, 1.26) | 0.70 | 0.96 (0.73, 1.27) | 0.79 |
| Difucosyldisialyllacto-N-hexaose-X1 | 0.91 (0.52, 1.62) | 0.75 | . (., .) | . | 0.95 (0.68, 1.32) | 0.75 | 1.17 (0.58, 2.38) | 0.66 |
| Difucosyldisialyllacto-N-hexaose-X2 | 1.16 (0.65, 2.07) | 0.61 | 1.11 (0.77, 1.60) | 0.58 | 0.84 (0.63, 1.12) | 0.22 | 1.07 (0.78, 1.45) | 0.68 |
| Difucosylsialyllacto-N-hexaose-X1 | 0.64 (0.37, 1.11) | 0.11 | . (., .) | . | 0.76 (0.54, 1.07) | 0.12 | . (., .) | . |
| **Difucosylsialyllacto-N-hexaose-X2** | 0.73 (0.47, 1.14) | 0.16 | . (., .) | . | **0.72 (0.55, 0.95)** | **0.02** | . (., .) | . |
| Disialyllacto-N-Hexaose-X1 | 1.25 (0.77, 2.02) | 0.36 | 1.48 (1.09, 2.00) | 0.01 | 1.12 (0.86, 1.46) | 0.41 | 1.14 (0.89, 1.47) | 0.31 |
| Disialyllacto-N-Hexaose-X2 | 1.40 (0.80, 2.46) | 0.24 | 1.05 (0.76, 1.45) | 0.79 | 1.04 (0.77, 1.41) | 0.78 | 1.04 (0.81, 1.33) | 0.76 |
| Disialyllacto-N-Hexaose-X5 | 0.57 (0.33, 1.01) | 0.05 | 1.27 (0.90, 1.79) | 0.17 | 0.75 (0.54, 1.04) | 0.08 | 1.14 (0.88, 1.49) | 0.31 |
| **Disialyllacto-N-tetraose** | **1.70 (1.02, 2.83)** | **0.04** | 0.99 (0.69, 1.41) | 0.95 | 1.10 (0.81, 1.47) | 0.55 | 1.02 (0.81, 1.28) | 0.88 |
| **Fucosyl(1-3)-iso-lacto-N-octaose** | **2.58 (1.59, 4.19)** | **<0.001*** | 1.00 (0.67, 1.47) | 0.98 | 1.40 (1.11, 1.78) | 0.01 | 0.93 (0.70, 1.23) | 0.61 |
| **Fucosyl-para-lacto-N-hexaose I** | **1.63 (1.08, 2.47)** | **0.02** | 1.01 (0.71, 1.43) | 0.96 | 1.20 (0.93, 1.56) | 0.16 | 1.01 (0.78, 1.33) | 0.92 |
| Fucosyldiasialyllacto-N-hexaose-X1 | 1.28 (0.72, 2.28) | 0.41 | 1.24 (0.89, 1.73) | 0.20 | 1.02 (0.77, 1.35) | 0.90 | 1.05 (0.78, 1.41) | 0.75 |
| Fucosyldiasialyllacto-N-hexaose-X2 | 1.19 (0.66, 2.15) | 0.56 | 0.97 (0.70, 1.36) | 0.88 | 0.95 (0.70, 1.28) | 0.72 | 1.05 (0.82, 1.34) | 0.70 |
| Fucosyldisialyllacto-N-tetraose-X2 | 1.40 (0.84, 2.34) | 0.20 | 0.99 (0.72, 1.35) | 0.93 | 0.95 (0.70, 1.28) | 0.72 | 1.09 (0.87, 1.36) | 0.47 |
| Fucosyllacto-N-hexaose II | 1.60 (0.94, 2.70) | 0.08 | 1.06 (0.73, 1.55) | 0.75 | 1.14 (0.87, 1.50) | 0.32 | 0.89 (0.67, 1.19) | 0.43 |
| Fucosyllacto-N-hexaose-X1 | 1.24 (0.76, 2.02) | 0.40 | . (., .) | . | 1.13 (0.88, 1.46) | 0.33 | . (., .) | . |
| Fucosyllacto-N-hexaose-X2 | 1.03 (0.63, 1.69) | 0.90 | . (., .) | . | 1.12 (0.86, 1.45) | 0.41 | . (., .) | . |
| **Fucosyllacto-N-hexaose-X4** | 0.87 (0.53, 1.41) | 0.57 | 1.23 (0.91, 1.67) | 0.18 | **0.75 (0.56, 0.99)** | **0.04** | 1.05 (0.81, 1.36) | 0.70 |
| Fucosyllacto-N-octaose-X1 | 1.15 (0.73, 1.81) | 0.55 | . (., .) | . | 1.12 (0.87, 1.45) | 0.39 | . (., .) | . |
| Fucosyllacto-N-octaose-X2 | 1.44 (0.86, 2.41) | 0.17 | 1.19 (0.84, 1.69) | 0.32 | 0.99 (0.72, 1.37) | 0.97 | 1.02 (0.80, 1.29) | 0.88 |
| Fucosyllacto-N-sulfate-X1 | 0.97 (0.58, 1.65) | 0.92 | . (., .) | . | 0.97 (0.74, 1.27) | 0.84 | . (., .) | . |
| Fucosylsialyllacto-N-Hexaose-X1 | 1.65 (0.90, 3.01) | 0.11 | 0.95 (0.67, 1.35) | 0.78 | 1.13 (0.85, 1.52) | 0.40 | 0.91 (0.68, 1.21) | 0.52 |
| Fucosylsialyllacto-N-Hexaose-X2 | 1.10 (0.69, 1.77) | 0.68 | 1.13 (0.75, 1.69) | 0.56 | 1.04 (0.79, 1.38) | 0.76 | 0.89 (0.65, 1.21) | 0.45 |
| **Fucosylsialyllacto-N-Hexaose-X3** | 0.96 (0.60, 1.53) | 0.85 | **0.50 (0.28, 0.91)** | **0.02** | 1.12 (0.85, 1.48) | 0.43 | 0.78 (0.47, 1.27) | 0.31 |
| **Fucosylsialyllacto-N-Hexaose-X4** | **0.50 (0.31, 0.83)** | **0.01** | 1.11 (0.79, 1.57) | 0.53 | 0.69 (0.50, 0.96) | 0.03 | 1.08 (0.84, 1.38) | 0.56 |
| Fucosylsialyllacto-N-Hexaose-X5 | 1.10 (0.67, 1.81) | 0.71 | 1.00 (0.66, 1.52) | 0.99 | 0.85 (0.64, 1.13) | 0.26 | 0.97 (0.69, 1.34) | 0.84 |
| Fucosylsialyllacto-N-Hexaose-X6 | 0.65 (0.41, 1.03) | 0.07 | 1.07 (0.75, 1.52) | 0.72 | 0.70 (0.53, 0.93) | 0.02 | 1.15 (0.90, 1.46) | 0.26 |
| Fucosylsialyllacto-N-neo-tetraose c | 0.76 (0.46, 1.27) | 0.30 | 1.05 (0.72, 1.51) | 0.80 | 0.71 (0.53, 0.95) | 0.02 | 1.11 (0.86, 1.45) | 0.42 |
| Fucosylsialyllacto-N-tetraose a | 1.34 (0.77, 2.34) | 0.31 | 1.01 (0.70, 1.45) | 0.97 | 0.93 (0.68, 1.28) | 0.65 | 0.94 (0.72, 1.23) | 0.65 |
| Fucosylsialyllacto-N-tetraose b | 1.44 (0.87, 2.38) | 0.15 | . (., .) | . | 1.11 (0.82, 1.50) | 0.49 | . (., .) | . |
| Fucosylsialyllacto-N-tetraose-X1 | 1.07 (0.60, 1.91) | 0.81 | 0.88 (0.62, 1.25) | 0.47 | 0.89 (0.65, 1.21) | 0.46 | 1.01 (0.78, 1.29) | 0.96 |
| **Lacto-N-decaose-X1** | **1.63 (1.05, 2.52)** | **0.03** | 1.23 (0.87, 1.73) | 0.25 | 1.24 (0.96, 1.61) | 0.10 | 0.99 (0.76, 1.30) | 0.94 |
| Lacto-N-difucohexaose I | 0.90 (0.59, 1.38) | 0.64 | . (., .) | . | 0.84 (0.64, 1.10) | 0.21 | . (., .) | . |
| Lacto-N-fucopentaose I | 1.32 (0.82, 2.12) | 0.25 | . (., .) | . | 1.25 (0.98, 1.60) | 0.07 | . (., .) | . |
| Lacto-N-fucopentaose II | 1.57 (0.97, 2.56) | 0.07 | 0.80 (0.57, 1.14) | 0.22 | 1.04 (0.78, 1.40) | 0.77 | 0.94 (0.72, 1.23) | 0.67 |
| **Lacto-N-fucopentaose III** | 0.74 (0.48, 1.14) | 0.17 | 0.80 (0.59, 1.08) | 0.15 | **0.73 (0.55, 0.95)** | **0.02** | 0.93 (0.73, 1.19) | 0.58 |
| **Lacto-N-fucopentaose V** | **1.83 (1.14, 2.95)** | **0.01** | 0.77 (0.58, 1.01) | 0.06 | 1.08 (0.81, 1.44) | 0.60 | 0.89 (0.72, 1.11) | 0.30 |
| Lacto-N-hexaose | 1.46 (1.02, 2.08) | 0.04 | 1.29 (0.92, 1.82) | 0.15 | 1.26 (0.99, 1.59) | 0.06 | 0.99 (0.75, 1.32) | 0.96 |
| Lacto-N-hexaose neo | 0.94 (0.55, 1.59) | 0.81 | 1.28 (0.90, 1.81) | 0.17 | 0.95 (0.72, 1.23) | 0.68 | 1.02 (0.78, 1.35) | 0.87 |
| **Lacto-N-neo-difucohexaose I** | **0.56 (0.32, 0.98)** | **0.04** | . (., .) | . | **0.66 (0.47, 0.93)** | **0.02** | . (., .) | . |
| **Lacto-N-neo-difucohexaose II** | 0.81 (0.49, 1.36) | 0.43 | 0.83 (0.57, 1.20) | 0.32 | **0.73 (0.55, 0.96)** | **0.03** | 1.00 (0.74, 1.36) | 1.00 |
| Lacto-N-neo-octaose | 1.41 (0.84, 2.39) | 0.197 | 1.18 (0.84, 1.65) | 0.35 | 1.16 (0.87, 1.55) | 0.32 | 0.98 (0.75, 1.29) | 0.89 |
| **Lacto-N-tetraose** | **2.14 (1.49, 3.08)** | **<0.001*** | 0.89 (0.64, 1.25) | 0.52 | **1.35 (1.04, 1.75)** | **0.02** | 0.92 (0.72, 1.16) | 0.48 |
| **Lacto-N-triose II** | **1.67 (1.08, 2.57)** | **0.02** | 1.13 (0.78, 1.63) | 0.53 | 1.11 (0.83, 1.47) | 0.49 | 1.03 (0.79, 1.36) | 0.80 |
| Lactose-3'-Sulfate | 1.39 (0.86, 2.26) | 0.182 | 0.75 (0.52, 1.08) | 0.12 | 1.01 (0.75, 1.38) | 0.93 | 0.99 (0.76, 1.30) | 0.96 |
| Sialyllacto-N-hexaose-X1 | 1.43 (0.90, 2.28) | 0.132 | 1.17 (0.77, 1.79) | 0.46 | 1.11 (0.87, 1.43) | 0.40 | 1.00 (0.75, 1.33) | 1.00 |
| Sialyllacto-N-hexaose-X2 | 0.78 (0.41, 1.49) | 0.458 | 1.32 (0.94, 1.85) | 0.11 | 0.89 (0.65, 1.22) | 0.46 | 1.04 (0.81, 1.34) | 0.75 |
| Sialyllacto-N-tetraose a | 1.60 (0.97, 2.63) | 0.064 | 1.02 (0.70, 1.50) | 0.91 | 1.34 (1.02, 1.77) | 0.04 | 0.94 (0.71, 1.25) | 0.66 |
| **Sialyllacto-N-tetraose b** | **2.16 (1.41, 3.29)** | **<0.001*** | 0.86 (0.63, 1.17) | 0.35 | 1.24 (0.93, 1.65) | 0.15 | 0.98 (0.78, 1.24) | 0.89 |
| Sialyllacto-N-tetraose c | 0.76 (0.46, 1.28) | 0.304 | 1.23 (0.85, 1.76) | 0.27 | 0.83 (0.62, 1.12) | 0.22 | 1.18 (0.92, 1.52) | 0.20 |
| Trifucosyl(1-2,1-2,1-3)-iso-lacto-N-octa | 1.22 (0.76, 1.94) | 0.41 | . (., .) | . | 1.27 (0.96, 1.69) | 0.09 | . (., .) | . |
| Trifucosyllacto-N-hexaose I | 0.84 (0.50, 1.40) | 0.499 | . (., .) | . | 0.76 (0.56, 1.03) | 0.08 | . (., .) | . |
| Trifucosyllacto-N-hexaose-X1 | 0.82 (0.51, 1.30) | 0.396 | . (., .) | . | 0.75 (0.57, 0.99) | 0.04 | . (., .) | . |
| Trifucosyllacto-N-octaose-X1 | 1.53 (0.83, 2.83) | 0.175 | . (., .) | . | 1.11 (0.81, 1.53) | 0.52 | . (., .) | . |
| Trifucosyllacto-N-tetraose-X5 | 1.01 (0.65, 1.57) | 0.972 | . (., .) | . | 0.90 (0.68, 1.19) | 0.46 | . (., .) | . |
| a-Heptasaccharide | 1.05 (0.62, 1.77) | 0.861 | . (., .) | . | 1.07 (0.80, 1.43) | 0.67 | . (., .) | . |
| a-Pentasaccharide | 1.13 (0.71, 1.80) | 0.615 | . (., .) | . | 1.10 (0.83, 1.46) | 0.49 | . (., .) | . |
| a-Tetrasaccharide | 1.27 (0.75, 2.12) | 0.372 | . (., .) | . | 1.18 (0.88, 1.58) | 0.27 | . (., .) | . |

Human milk oligosaccharides were normalised after stratification by secretor status. Associations determined by modified Poisson regression. Models adjusted for batch, child sex, duration of exclusive breastfeeding (weeks) and gestation age (weeks). Bold denotes statistically significant at nominal level (p<0.05). *Denotes statistically significant after correction for multiple testing (FDR<0.010). RR: Risk Ratio; CI: Confidence Interval; LRTI: Lower respiratory tract infections; HMO: Human milk oligosaccharides

**Table S25**: Adjusted associations between human milk oligosaccharides measured at 6 weeks of lactation with upper respiratory tract infections (URTI) in the first or second year of life stratified by secretor status in the Ulm SPATZ Health Study.

|  | **URTI at 1 year** | | | | **URTI at 2 years** | | | |
| --- | --- | --- | --- | --- | --- | --- | --- | --- |
|  | Secretor milk | | Non-secretor milk | | Secretor milk | | Non-secretor milk | |
| **HMO Structure** | RR (95% CI) | p | RR (95% CI) | p | RR (95% CI) | p | RR (95% CI) | p |
| 2,3 Difucosyllactose | 1.06 (0.90, 1.24) | 0.49 | . (., .) | . | 1.00 (0.89, 1.11) | 0.94 | . (., .) | . |
| 2-Fucosyllactose | 0.92 (0.75, 1.12) | 0.41 | . (., .) | . | 0.89 (0.79, 1.00) | 0.05 | . (., .) | . |
| 3'-Sialyl-3-fucosyllactose | 1.07 (0.89, 1.29) | 0.44 | 1.05 (0.89, 1.24) | 0.57 | 1.09 (0.99, 1.20) | 0.08 | 1.02 (0.90, 1.15) | 0.81 |
| 3-Fucosyllactose | 0.99 (0.82, 1.19) | 0.89 | 0.97 (0.82, 1.15) | 0.73 | 1.04 (0.93, 1.17) | 0.47 | 0.95 (0.86, 1.06) | 0.39 |
| 3-Galactosyllactose | 1.11 (0.94, 1.32) | 0.22 | 1.17 (0.98, 1.40) | 0.09 | 1.10 (0.98, 1.22) | 0.10 | 1.04 (0.95, 1.15) | 0.39 |
| 3-sialyllactose | 1.09 (0.90, 1.32) | 0.35 | 1.01 (0.85, 1.21) | 0.89 | 1.04 (0.92, 1.17) | 0.51 | 1.01 (0.90, 1.13) | 0.88 |
| 6'-Sialyl-N-acetyllactosamine | 1.06 (0.89, 1.25) | 0.54 | 1.02 (0.83, 1.24) | 0.87 | 0.98 (0.89, 1.09) | 0.74 | 1.08 (0.95, 1.23) | 0.22 |
| 6-Galactosyllactose | 1.00 (0.83, 1.19) | 0.97 | 1.03 (0.85, 1.25) | 0.76 | 1.00 (0.88, 1.12) | 0.94 | 1.09 (0.97, 1.22) | 0.16 |
| 6-sialyllactose | 1.01 (0.86, 1.18) | 0.94 | 1.00 (0.80, 1.26) | 0.97 | 0.95 (0.86, 1.05) | 0.31 | 1.09 (0.97, 1.23) | 0.15 |
| Difucosyl-lacto-N-hexaose I | 1.03 (0.86, 1.23) | 0.78 | . (., .) | . | 0.97 (0.87, 1.08) | 0.56 | . (., .) | . |
| Difucosyl-lacto-N-hexaose II | 1.01 (0.84, 1.22) | 0.88 | 0.86 (0.69, 1.08) | 0.20 | 1.08 (0.97, 1.20) | 0.14 | 0.95 (0.83, 1.09) | 0.47 |
| Difucosyl-lacto-N-hexaose-X1 | 1.06 (0.89, 1.26) | 0.52 | . (., .) | . | 0.99 (0.89, 1.09) | 0.83 | . (., .) | . |
| Difucosyl-lacto-N-hexaose-X2 | 1.07 (0.91, 1.26) | 0.43 | . (., .) | . | 0.98 (0.86, 1.12) | 0.78 | . (., .) | . |
| Difucosyl-lacto-N-hexaose-X3 | 1.17 (0.98, 1.40) | 0.09 | 1.12 (0.90, 1.39) | 0.31 | 1.16 (1.02, 1.32) | 0.03 | 1.08 (0.95, 1.22) | 0.23 |
| Difucosyl-lacto-N-neo-hexaose I | 1.05 (0.86, 1.27) | 0.65 | 0.80 (0.63, 1.01) | 0.06 | 1.11 (0.96, 1.28) | 0.16 | 0.95 (0.84, 1.08) | 0.47 |
| Difucosyl-para-lacto-N-hexaose I | 1.09 (0.91, 1.31) | 0.33 | 1.08 (0.89, 1.32) | 0.44 | 1.11 (0.99, 1.24) | 0.07 | 1.05 (0.93, 1.19) | 0.40 |
| Difucosyldisialyllacto-N-hexaose-X1 | 1.09 (0.91, 1.30) | 0.36 | 1.05 (0.63, 1.74) | 0.86 | 0.94 (0.83, 1.07) | 0.37 | 1.11 (0.95, 1.30) | 0.20 |
| Difucosyldisialyllacto-N-hexaose-X2 | 0.97 (0.81, 1.16) | 0.74 | 0.89 (0.72, 1.09) | 0.25 | 1.05 (0.95, 1.16) | 0.36 | 0.96 (0.85, 1.10) | 0.59 |
| Difucosylsialyllacto-N-hexaose-X1 | 1.10 (0.90, 1.35) | 0.37 | . (., .) | . | 0.98 (0.87, 1.11) | 0.76 | . (., .) | . |
| Difucosylsialyllacto-N-hexaose-X2 | 1.03 (0.87, 1.23) | 0.69 | . (., .) | . | 0.96 (0.84, 1.09) | 0.53 | . (., .) | . |
| Disialyllacto-N-Hexaose-X1 | 1.08 (0.93, 1.27) | 0.31 | 0.99 (0.84, 1.18) | 0.92 | 1.01 (0.91, 1.11) | 0.92 | 1.00 (0.90, 1.12) | 0.94 |
| Disialyllacto-N-Hexaose-X2 | 1.13 (0.94, 1.36) | 0.20 | 1.11 (0.90, 1.36) | 0.33 | 1.06 (0.94, 1.19) | 0.33 | 1.03 (0.94, 1.13) | 0.52 |
| Disialyllacto-N-Hexaose-X5 | 0.98 (0.81, 1.19) | 0.88 | 0.98 (0.79, 1.21) | 0.84 | 0.98 (0.85, 1.12) | 0.73 | 1.07 (0.96, 1.19) | 0.23 |
| Disialyllacto-N-tetraose | 1.03 (0.85, 1.25) | 0.77 | 1.00 (0.84, 1.18) | 0.99 | 1.04 (0.93, 1.17) | 0.50 | 1.03 (0.94, 1.12) | 0.56 |
| Fucosyl(1-3)-iso-lacto-N-octaose | 1.09 (0.91, 1.32) | 0.34 | 1.00 (0.85, 1.17) | 0.99 | 1.11 (1.01, 1.23) | 0.03 | 1.03 (0.95, 1.13) | 0.47 |
| Fucosyl-para-lacto-N-hexaose I | 1.11 (0.93, 1.32) | 0.25 | 1.11 (0.92, 1.33) | 0.27 | 1.12 (0.99, 1.27) | 0.08 | 1.08 (0.97, 1.22) | 0.17 |
| Fucosyldiasialyllacto-N-hexaose-X1 | 1.03 (0.85, 1.25) | 0.75 | 0.82 (0.67, 1.01) | 0.07 | 1.04 (0.95, 1.13) | 0.40 | 0.96 (0.85, 1.08) | 0.50 |
| Fucosyldiasialyllacto-N-hexaose-X2 | 1.11 (0.90, 1.37) | 0.32 | 1.01 (0.84, 1.21) | 0.94 | 1.11 (0.97, 1.26) | 0.13 | 1.06 (0.95, 1.18) | 0.33 |
| Fucosyldisialyllacto-N-tetraose-X2 | 1.08 (0.90, 1.29) | 0.43 | 1.04 (0.89, 1.23) | 0.61 | 1.09 (0.98, 1.21) | 0.12 | 1.03 (0.93, 1.15) | 0.57 |
| Fucosyllacto-N-hexaose II | 1.07 (0.89, 1.29) | 0.47 | 0.89 (0.74, 1.07) | 0.23 | 1.11 (1.00, 1.23) | 0.05 | 0.98 (0.89, 1.07) | 0.63 |
| Fucosyllacto-N-hexaose-X1 | 1.05 (0.89, 1.24) | 0.55 | . (., .) | . | 0.99 (0.91, 1.08) | 0.77 | . (., .) | . |
| Fucosyllacto-N-hexaose-X2 | 1.03 (0.85, 1.23) | 0.79 | . (., .) | . | 0.96 (0.87, 1.07) | 0.51 | . (., .) | . |
| Fucosyllacto-N-hexaose-X4 | 1.18 (0.96, 1.45) | 0.11 | 1.15 (0.93, 1.44) | 0.20 | 1.11 (0.97, 1.28) | 0.13 | 1.06 (0.93, 1.22) | 0.39 |
| Fucosyllacto-N-octaose-X1 | 1.09 (0.92, 1.29) | 0.34 | . (., .) | . | 1.02 (0.93, 1.13) | 0.64 | . (., .) | . |
| Fucosyllacto-N-octaose-X2 | 1.16 (0.96, 1.40) | 0.12 | 1.05 (0.84, 1.32) | 0.66 | 1.12 (0.99, 1.27) | 0.08 | 1.00 (0.88, 1.14) | 0.96 |
| Fucosyllacto-N-sulfate-X1 | 1.07 (0.92, 1.25) | 0.37 | . (., .) | . | 1.01 (0.94, 1.09) | 0.76 | . (., .) | . |
| Fucosylsialyllacto-N-Hexaose-X1 | 1.07 (0.88, 1.31) | 0.49 | 0.94 (0.78, 1.14) | 0.54 | 1.11 (1.00, 1.23) | 0.06 | 0.96 (0.86, 1.07) | 0.43 |
| Fucosylsialyllacto-N-Hexaose-X2 | 1.01 (0.85, 1.21) | 0.88 | 0.84 (0.68, 1.04) | 0.12 | 1.01 (0.93, 1.09) | 0.88 | 1.00 (0.90, 1.10) | 0.94 |
| Fucosylsialyllacto-N-Hexaose-X3 | 1.00 (0.84, 1.20) | 0.99 | 0.88 (0.68, 1.15) | 0.36 | 0.94 (0.84, 1.05) | 0.25 | 1.00 (0.88, 1.14) | 0.94 |
| Fucosylsialyllacto-N-Hexaose-X4 | 1.01 (0.83, 1.23) | 0.91 | 0.96 (0.78, 1.18) | 0.69 | 1.03 (0.92, 1.17) | 0.59 | 1.07 (0.95, 1.20) | 0.27 |
| Fucosylsialyllacto-N-Hexaose-X5 | 1.00 (0.83, 1.19) | 0.96 | 0.91 (0.73, 1.12) | 0.35 | 1.03 (0.93, 1.14) | 0.57 | 0.98 (0.88, 1.09) | 0.75 |
| Fucosylsialyllacto-N-Hexaose-X6 | 1.14 (0.95, 1.37) | 0.15 | 1.09 (0.89, 1.35) | 0.40 | 1.10 (0.97, 1.24) | 0.14 | 1.11 (0.97, 1.26) | 0.14 |
| Fucosylsialyllacto-N-neo-tetraose c | 1.09 (0.90, 1.32) | 0.37 | 1.09 (0.89, 1.35) | 0.40 | 1.07 (0.95, 1.22) | 0.25 | 1.10 (0.96, 1.25) | 0.16 |
| Fucosylsialyllacto-N-tetraose a | 0.97 (0.82, 1.16) | 0.76 | 0.92 (0.77, 1.11) | 0.40 | 1.04 (0.92, 1.16) | 0.53 | 1.02 (0.94, 1.09) | 0.67 |
| Fucosylsialyllacto-N-tetraose b | 1.12 (0.92, 1.35) | 0.27 | . (., .) | . | 1.02 (0.90, 1.16) | 0.75 | . (., .) | . |
| Fucosylsialyllacto-N-tetraose-X1 | 1.15 (0.96, 1.37) | 0.12 | 1.11 (0.92, 1.33) | 0.28 | 1.16 (1.02, 1.32) | 0.02 | 1.03 (0.92, 1.16) | 0.59 |
| Lacto-N-decaose-X1 | 1.11 (0.95, 1.30) | 0.19 | 0.98 (0.84, 1.16) | 0.84 | 1.07 (0.97, 1.19) | 0.20 | 1.02 (0.93, 1.12) | 0.74 |
| Lacto-N-difucohexaose I | 1.07 (0.91, 1.25) | 0.42 | . (., .) | . | 0.99 (0.89, 1.11) | 0.92 | . (., .) | . |
| Lacto-N-fucopentaose I | 1.04 (0.87, 1.23) | 0.67 | . (., .) | . | 0.96 (0.87, 1.07) | 0.48 | . (., .) | . |
| Lacto-N-fucopentaose II | 1.05 (0.87, 1.26) | 0.61 | 1.07 (0.88, 1.30) | 0.47 | 1.08 (0.98, 1.20) | 0.14 | 1.01 (0.89, 1.13) | 0.93 |
| Lacto-N-fucopentaose III | 1.09 (0.90, 1.32) | 0.37 | 1.06 (0.86, 1.31) | 0.59 | 1.15 (0.99, 1.33) | 0.06 | 1.03 (0.92, 1.16) | 0.58 |
| Lacto-N-fucopentaose V | 1.05 (0.87, 1.27) | 0.59 | 1.02 (0.84, 1.23) | 0.85 | 1.10 (0.98, 1.24) | 0.10 | 0.99 (0.89, 1.11) | 0.92 |
| Lacto-N-hexaose | 1.05 (0.88, 1.24) | 0.61 | 1.03 (0.87, 1.21) | 0.75 | 1.02 (0.92, 1.14) | 0.68 | 1.01 (0.92, 1.11) | 0.79 |
| Lacto-N-neo-difucohexaose I | 1.10 (0.88, 1.38) | 0.39 | . (., .) | . | 1.04 (0.93, 1.17) | 0.49 | . (., .) | . |
| Lacto-N-neo-difucohexaose II | 1.03 (0.86, 1.25) | 0.74 | 1.06 (0.88, 1.27) | 0.55 | 1.09 (0.96, 1.24) | 0.16 | 1.04 (0.94, 1.15) | 0.42 |
| Lacto-N-neo-hexaose | 1.11 (0.93, 1.34) | 0.25 | 1.02 (0.86, 1.21) | 0.85 | 1.05 (0.96, 1.16) | 0.27 | 1.00 (0.91, 1.11) | 0.95 |
| Lacto-N-neo-octaose | 1.11 (0.95, 1.28) | 0.19 | 1.00 (0.85, 1.18) | 0.99 | 1.06 (0.98, 1.15) | 0.17 | 1.01 (0.91, 1.11) | 0.89 |
| Lacto-N-tetraose | 1.06 (0.89, 1.26) | 0.51 | 1.07 (0.89, 1.29) | 0.45 | 1.07 (0.95, 1.21) | 0.27 | 1.03 (0.93, 1.14) | 0.61 |
| Lacto-N-triose II | 1.10 (0.92, 1.31) | 0.30 | 1.13 (0.91, 1.40) | 0.27 | 1.10 (0.97, 1.25) | 0.12 | 1.03 (0.91, 1.16) | 0.66 |
| Lactose-3'-Sulfate | 1.03 (0.85, 1.25) | 0.77 | 1.09 (0.91, 1.31) | 0.33 | 1.01 (0.91, 1.13) | 0.84 | 1.05 (0.94, 1.18) | 0.41 |
| Sialyllacto-N-hexaose-X1 | 1.03 (0.87, 1.22) | 0.77 | 0.92 (0.77, 1.11) | 0.40 | 1.03 (0.93, 1.14) | 0.56 | 1.03 (0.95, 1.13) | 0.46 |
| Sialyllacto-N-hexaose-X2 | 1.07 (0.89, 1.28) | 0.48 | 1.06 (0.87, 1.30) | 0.57 | 0.98 (0.88, 1.09) | 0.73 | 1.05 (0.94, 1.18) | 0.36 |
| Sialyllacto-N-tetraose a | 1.03 (0.87, 1.23) | 0.72 | 0.94 (0.79, 1.12) | 0.49 | 1.03 (0.93, 1.15) | 0.54 | 1.04 (0.96, 1.13) | 0.31 |
| Sialyllacto-N-tetraose b | 1.07 (0.89, 1.28) | 0.49 | 1.17 (0.96, 1.43) | 0.13 | 1.07 (0.94, 1.21) | 0.29 | 1.01 (0.89, 1.16) | 0.86 |
| Sialyllacto-N-tetraose c | 1.08 (0.92, 1.27) | 0.34 | 1.14 (0.92, 1.42) | 0.23 | 1.00 (0.90, 1.11) | 1.00 | 1.10 (0.96, 1.25) | 0.16 |
| Trifucosyl(1-2,1-2,1-3)-iso-lacto-N-octa | 1.02 (0.85, 1.21) | 0.84 | . (., .) | . | 0.97 (0.87, 1.07) | 0.51 | . (., .) | . |
| Trifucosyllacto-N-hexaose I | 1.06 (0.90, 1.23) | 0.49 | . (., .) | . | 0.99 (0.89, 1.11) | 0.88 | . (., .) | . |
| Trifucosyllacto-N-hexaose-X1 | 1.11 (0.93, 1.31) | 0.24 | . (., .) | . | 1.06 (0.96, 1.18) | 0.26 | . (., .) | . |
| Trifucosyllacto-N-octaose-X1 | 1.13 (0.97, 1.33) | 0.12 | . (., .) | . | 1.03 (0.93, 1.15) | 0.55 | . (., .) | . |
| Trifucosyllacto-N-tetraose-X5 | 1.07 (0.91, 1.25) | 0.44 | . (., .) | . | 1.01 (0.92, 1.11) | 0.88 | . (., .) | . |
| a-Heptasaccharide | 1.12 (0.92, 1.37) | 0.25 | . (., .) | . | 1.10 (0.97, 1.26) | 0.14 | . (., .) | . |
| a-Pentasaccharide | 1.14 (0.93, 1.40) | 0.21 | . (., .) | . | 1.10 (0.96, 1.27) | 0.18 | . (., .) | . |
| a-Tetrasaccharide | 1.18 (0.98, 1.42) | 0.07 | . (., .) | . | 1.10 (0.97, 1.25) | 0.15 | . (., .) | . |

Human milk oligosaccharide levels were normalised after stratification. Associations determined by modified Poisson regression. Models adjusted for batch, child sex, duration of exclusive breastfeeding (weeks) and gestation age (weeks). Bold denotes statistically significant at nominal level (p<0.05). *Denotes statistically significant after correction for multiple testing (FDR<0.010). RR: Risk Ratio; CI: Confidence Interval; URTI: Upper respiratory tract infections; HMO: Human milk oligosaccharides

**Table S26**: Adjusted associations between human milk oligosaccharides measured at 6 months of lactation with otitis media (OM) in the first or second year of life stratified by secretor status in the Ulm SPATZ Health Study.

|  | OM at 1 year | | | | OM at 2 years | | | |
| --- | --- | --- | --- | --- | --- | --- | --- | --- |
|  | Secretor milk | | Non-secretor milk | | Secretor milk | | Non-secretor milk | |
| **HMO Structure** | RR (95% CI) | p | RR (95% CI) | p | RR (95% CI) | p | RR (95% CI) | p |
| 2,3 Difucosyllactose | 1.00 (0.63, 1.60) | 0.99 | . (., .) | **.** | 0.93 (0.67, 1.29) | 0.67 | . (., .) | **.** |
| 2-Fucosyllactose | 0.91 (0.53, 1.58) | 0.74 | . (., .) | **.** | 1.05 (0.75, 1.49) | 0.76 | . (., .) | **.** |
| 3'-Sialyl-3-fucosyllactose | 1.20 (0.76, 1.90) | 0.43 | 1.87 (0.82, 4.28) | 0.14 | 1.02 (0.74, 1.40) | 0.9 | 1.03 (0.58, 1.83) | 0.91 |
| 3-Fucosyllactose | 0.93 (0.59, 1.48) | 0.77 | 1.37 (0.59, 3.20) | 0.46 | 0.86 (0.62, 1.20) | 0.38 | 0.67 (0.42, 1.08) | 0.1 |
| **3-Galactosyllactose** | 0.91 (0.46, 1.82) | 0.79 | **3.30 (1.79, 6.07)** | **<0.001*** | 1.05 (0.71, 1.56) | 0.8 | 2.23 (1.30, 3.83) | **<0.001** |
| 3-sialyllactose | 1.33 (0.71, 2.48) | 0.37 | 2.09 (0.88, 4.97) | 0.10 | 1.05 (0.73, 1.51) | 0.81 | 1.45 (0.89, 2.37) | 0.14 |
| 6'-Sialyl-N-acetyllactosamine | 0.89 (0.55, 1.42) | 0.62 | 1.29 (0.64, 2.62) | 0.48 | 0.91 (0.64, 1.30) | 0.6 | 1.40 (0.86, 2.29) | 0.18 |
| **6-Galactosyllactose** | 0.82 (0.47, 1.44) | 0.5 | **2.48 (1.42, 4.36)** | **<0.001** | 0.97 (0.65, 1.44) | 0.88 | 1.58 (0.89, 2.81) | 0.12 |
| 6-sialyllactose | 0.82 (0.47, 1.41) | 0.46 | 0.86 (0.41, 1.78) | 0.68 | 0.90 (0.62, 1.30) | 0.57 | 1.05 (0.64, 1.74) | 0.84 |
| Difucosyl-lacto-N-hexaose I | 1.13 (0.62, 2.04) | 0.69 | . (., .) | **.** | 1.09 (0.77, 1.55) | 0.61 | . (., .) | **.** |
| **Difucosyl-lacto-N-hexaose II** | 1.41 (0.80, 2.49) | 0.24 | 0.93 (0.45, 1.95) | 0.86 | 0.95 (0.65, 1.38) | 0.79 | **0.56 (0.37, 0.85)** | **0.01** |
| Difucosyl-lacto-N-hexaose-X1 | 1.50 (0.90, 2.49) | 0.12 | . (., .) | **.** | 1.26 (0.90, 1.75) | 0.18 | . (., .) | **.** |
| Difucosyl-lacto-N-hexaose-X2 | 1.53 (0.83, 2.81) | 0.18 | . (., .) | **.** | 0.94 (0.65, 1.35) | 0.72 | . (., .) | **.** |
| Difucosyl-lacto-N-hexaose-X3 | 1.05 (0.56, 1.98) | 0.87 | 1.40 (0.74, 2.65) | 0.31 | 0.92 (0.64, 1.33) | 0.66 | 1.25 (0.84, 1.86) | 0.26 |
| Difucosyl-lacto-N-neo-hexaose I | 1.03 (0.64, 1.67) | 0.9 | 0.96 (0.45, 2.06) | 0.92 | 0.93 (0.68, 1.28) | 0.66 | 0.86 (0.51, 1.44) | 0.57 |
| Difucosyl-para-lacto-N-hexaose I | 1.61 (0.89, 2.93) | 0.12 | 1.62 (0.54, 4.91) | 0.39 | 0.95 (0.68, 1.34) | 0.79 | 0.98 (0.51, 1.90) | 0.95 |
| Difucosyldisialyllacto-N-hexaose-X1 | 1.10 (0.65, 1.87) | 0.72 | . (., .) | **.** | 0.97 (0.60, 1.57) | 0.9 | . (., .) | **.** |
| **Difucosyldisialyllacto-N-hexaose-X2** | 1.18 (0.70, 2.00) | 0.54 | 0.92 (0.44, 1.94) | 0.83 | 0.95 (0.65, 1.40) | 0.8 | **0.56 (0.35, 0.89)** | **0.01** |
| Difucosylsialyllacto-N-hexaose-X1 | 1.04 (0.57, 1.91) | 0.89 | . (., .) | **.** | 1.07 (0.71, 1.62) | 0.75 | . (., .) | **.** |
| Difucosylsialyllacto-N-hexaose-X2 | 0.83 (0.38, 1.80) | 0.64 | . (., .) | **.** | 0.79 (0.51, 1.23) | 0.3 | . (., .) | **.** |
| **Disialyllacto-N-Hexaose-X1** | 1.23 (0.61, 2.50) | 0.56 | **0.47 (0.23, 0.95)** | **0.04** | 0.98 (0.68, 1.42) | 0.92 | 0.70 (0.36, 1.34) | 0.28 |
| Disialyllacto-N-Hexaose-X2 | 1.47 (0.81, 2.69) | 0.21 | 1.19 (0.45, 3.14) | 0.73 | 1.05 (0.74, 1.47) | 0.8 | 1.04 (0.58, 1.86) | 0.88 |
| Disialyllacto-N-Hexaose-X5 | 0.87 (0.43, 1.73) | 0.69 | 0.75 (0.42, 1.36) | 0.35 | 0.89 (0.60, 1.33) | 0.57 | 1.32 (0.78, 2.22) | 0.3 |
| Disialyllacto-N-tetraose | 1.44 (0.71, 2.91) | 0.32 | 1.17 (0.25, 5.52) | 0.84 | 1.04 (0.73, 1.47) | 0.83 | 1.23 (0.60, 2.54) | 0.57 |
| Fucosyl(1-3)-iso-lacto-N-octaose | 1.29 (0.60, 2.77) | 0.51 | 0.89 (0.34, 2.34) | 0.81 | 0.99 (0.68, 1.44) | 0.97 | 1.40 (0.76, 2.59) | 0.28 |
| Fucosyl-para-lacto-N-hexaose I | 1.55 (0.82, 2.92) | 0.17 | 0.94 (0.35, 2.55) | 0.9 | 1.05 (0.73, 1.50) | 0.79 | 1.64 (0.90, 2.98) | 0.1 |
| Fucosyldiasialyllacto-N-hexaose-X1 | 1.22 (0.55, 2.69) | 0.63 | 0.61 (0.20, 1.84) | 0.38 | 1.06 (0.71, 1.57) | 0.78 | 0.80 (0.45, 1.41) | 0.44 |
| Fucosyldiasialyllacto-N-hexaose-X2 | 1.40 (0.62, 3.17) | 0.41 | 1.08 (0.35, 3.30) | 0.89 | 1.10 (0.76, 1.60) | 0.61 | 1.54 (0.85, 2.80) | 0.15 |
| Fucosyldisialyllacto-N-tetraose-X2 | 1.54 (0.95, 2.50) | 0.08 | 1.58 (0.78, 3.19) | 0.2 | 1.04 (0.75, 1.45) | 0.81 | 0.96 (0.56, 1.65) | 0.89 |
| Fucosyllacto-N-hexaose II | 1.30 (0.66, 2.56) | 0.45 | 0.66 (0.25, 1.71) | 0.39 | 1.06 (0.74, 1.53) | 0.74 | 1.07 (0.60, 1.89) | 0.83 |
| Fucosyllacto-N-hexaose-X1 | 1.27 (0.82, 1.97) | 0.28 | . (., .) | **.** | 1.06 (0.75, 1.50) | 0.74 | . (., .) | **.** |
| Fucosyllacto-N-hexaose-X2 | 1.14 (0.55, 2.38) | 0.73 | . (., .) | **.** | 0.98 (0.63, 1.53) | 0.94 | . (., .) | **.** |
| Fucosyllacto-N-hexaose-X4 | 1.32 (0.81, 2.13) | 0.26 | 0.87 (0.37, 2.07) | 0.76 | 0.94 (0.67, 1.32) | 0.73 | 0.90 (0.52, 1.56) | 0.7 |
| Fucosyllacto-N-octaose-X1 | 1.13 (0.56, 2.28) | 0.72 | . (., .) | **.** | 0.99 (0.61, 1.60) | 0.96 | . (., .) | **.** |
| Fucosyllacto-N-octaose-X2 | 1.19 (0.67, 2.10) | 0.55 | 0.58 (0.17, 2.00) | 0.39 | 0.92 (0.61, 1.40) | 0.71 | 0.81 (0.38, 1.73) | 0.58 |
| Fucosyllacto-N-sulfate-X1 | 1.37 (0.75, 2.48) | 0.3 | . (., .) | **.** | 1.27 (0.86, 1.87) | 0.23 | . (., .) | **.** |
| Fucosylsialyllacto-N-Hexaose-X1 | 1.72 (1.00, 2.98) | 0.05 | 0.49 (0.23, 1.04) | 0.06 | 0.98 (0.67, 1.44) | 0.92 | 0.97 (0.59, 1.58) | 0.9 |
| Fucosylsialyllacto-N-Hexaose-X2 | 1.45 (0.78, 2.70) | 0.24 | 1.00 (0.38, 2.59) | 1 | 1.27 (0.88, 1.85) | 0.21 | 0.95 (0.50, 1.81) | 0.88 |
| **Fucosylsialyllacto-N-Hexaose-X3** | 0.95 (0.43, 2.09) | 0.89 | 1.48 (0.63, 3.51) | 0.37 | 1.11 (0.72, 1.71) | 0.62 | **2.21 (1.30, 3.75)** | **<0.001** |
| Fucosylsialyllacto-N-Hexaose-X4 | 0.95 (0.54, 1.67) | 0.85 | 0.88 (0.39, 1.97) | 0.76 | 0.90 (0.61, 1.31) | 0.57 | 1.24 (0.72, 2.13) | 0.43 |
| Fucosylsialyllacto-N-Hexaose-X5 | 0.73 (0.25, 2.14) | 0.56 | 0.54 (0.23, 1.24) | 0.15 | 0.61 (0.31, 1.17) | 0.13 | 0.59 (0.35, 1.00) | 0.05 |
| Fucosylsialyllacto-N-Hexaose-X6 | 0.70 (0.30, 1.63) | 0.41 | 1.35 (0.62, 2.95) | 0.45 | 0.96 (0.62, 1.49) | 0.86 | 1.51 (0.95, 2.41) | 0.08 |
| Fucosylsialyllacto-N-neo-tetraose c | 1.14 (0.66, 1.97) | 0.63 | 1.21 (0.50, 2.93) | 0.68 | 0.86 (0.61, 1.21) | 0.38 | 1.04 (0.60, 1.81) | 0.9 |
| Fucosylsialyllacto-N-tetraose a | 1.12 (0.64, 1.95) | 0.69 | 1.60 (0.46, 5.53) | 0.46 | 0.94 (0.67, 1.33) | 0.73 | 0.83 (0.42, 1.63) | 0.58 |
| Fucosylsialyllacto-N-tetraose b | 1.56 (0.80, 3.06) | 0.19 | . (., .) | **.** | 1.11 (0.76, 1.61) | 0.59 | . (., .) | **.** |
| Fucosylsialyllacto-N-tetraose-X1 | 1.52 (0.88, 2.62) | 0.14 | 1.53 (0.80, 2.93) | 0.2 | 1.05 (0.73, 1.53) | 0.78 | 1.24 (0.79, 1.94) | 0.36 |
| Lacto-N-decaose-X1 | 1.46 (0.79, 2.72) | 0.23 | 1.45 (0.64, 3.28) | 0.37 | 1.13 (0.69, 1.84) | 0.63 | 1.35 (0.76, 2.37) | 0.31 |
| Lacto-N-difucohexaose I | 1.75 (0.89, 3.44) | 0.1 | . (., .) | **.** | 1.01 (0.69, 1.46) | 0.98 | . (., .) | **.** |
| Lacto-N-fucopentaose I | 1.51 (0.93, 2.44) | 0.09 | . (., .) | **.** | 1.17 (0.84, 1.64) | 0.34 | . (., .) | **.** |
| Lacto-N-fucopentaose II | 1.59 (0.96, 2.61) | 0.07 | 1.45 (0.49, 4.26) | 0.5 | 0.99 (0.72, 1.36) | 0.96 | 0.85 (0.47, 1.54) | 0.58 |
| Lacto-N-fucopentaose III | 1.27 (0.71, 2.26) | 0.43 | 1.31 (0.66, 2.58) | 0.43 | 0.98 (0.68, 1.41) | 0.91 | 1.30 (0.81, 2.07) | 0.28 |
| Lacto-N-fucopentaose V | 1.72 (0.88, 3.39) | 0.12 | 1.54 (0.45, 5.30) | 0.49 | 0.93 (0.67, 1.27) | 0.64 | 1.07 (0.54, 2.12) | 0.85 |
| Lacto-N-hexaose | 1.24 (0.56, 2.78) | 0.6 | 1.14 (0.64, 2.01) | 0.66 | 1.01 (0.67, 1.53) | 0.94 | 1.31 (0.83, 2.07) | 0.24 |
| Lacto-N-neo-difucohexaose I | 1.28 (0.69, 2.37) | 0.43 | . (., .) | **.** | 1.06 (0.71, 1.58) | 0.77 | . (., .) | **.** |
| Lacto-N-neo-difucohexaose II | 1.05 (0.71, 1.56) | 0.8 | 1.84 (0.97, 3.51) | 0.06 | 0.88 (0.65, 1.18) | 0.39 | 0.84 (0.50, 1.41) | 0.5 |
| Lacto-N-neo-hexaose | 1.34 (0.74, 2.43) | 0.33 | 1.18 (0.67, 2.09) | 0.57 | 1.01 (0.69, 1.46) | 0.98 | 1.29 (0.81, 2.08) | 0.29 |
| Lacto-N-neo-octaose | 1.35 (0.79, 2.31) | 0.27 | 1.25 (0.36, 4.31) | 0.73 | 1.04 (0.71, 1.53) | 0.82 | 1.26 (0.60, 2.65) | 0.54 |
| Lacto-N-tetraose | 1.71 (0.89, 3.30) | 0.11 | 1.06 (0.31, 3.66) | 0.92 | 1.02 (0.73, 1.42) | 0.93 | 1.52 (0.79, 2.94) | 0.21 |
| **Lacto-N-triose II** | 1.35 (0.64, 2.85) | 0.44 | 1.18 (0.63, 2.19) | 0.61 | 1.00 (0.70, 1.42) | 0.99 | **1.74 (1.15, 2.62)** | **0.01** |
| Lactose-3'-Sulfate | 1.19 (0.70, 2.00) | 0.52 | 0.74 (0.17, 3.25) | 0.69 | 1.08 (0.73, 1.60) | 0.69 | 1.08 (0.57, 2.05) | 0.81 |
| Sialyllacto-N-hexaose-X1 | 1.14 (0.45, 2.90) | 0.79 | 0.68 (0.31, 1.45) | 0.32 | 0.95 (0.59, 1.53) | 0.82 | 1.21 (0.70, 2.09) | 0.49 |
| Sialyllacto-N-hexaose-X2 | 0.86 (0.47, 1.59) | 0.64 | . (., .) | **.** | 0.87 (0.59, 1.28) | 0.47 | 1.19 (0.51, 2.76) | 0.69 |
| Sialyllacto-N-tetraose a | 1.06 (0.50, 2.24) | 0.87 | 1.16 (0.31, 4.34) | 0.83 | 1.18 (0.83, 1.68) | 0.37 | 1.40 (0.70, 2.80) | 0.34 |
| Sialyllacto-N-tetraose b | 1.93 (0.97, 3.86) | 0.06 | 1.20 (0.57, 2.55) | 0.63 | 1.02 (0.72, 1.44) | 0.91 | 1.29 (0.81, 2.05) | 0.28 |
| Sialyllacto-N-tetraose c | 1.05 (0.64, 1.71) | 0.86 | 0.77 (0.37, 1.58) | 0.48 | 0.96 (0.66, 1.39) | 0.82 | 1.14 (0.68, 1.90) | 0.63 |
| Trifucosyl(1-2,1-2,1-3)-iso-lacto-N-octa | 1.30 (0.71, 2.37) | 0.4 | . (., .) | **.** | 1.09 (0.75, 1.58) | 0.66 | . (., .) | **.** |
| Trifucosyllacto-N-hexaose I | 1.28 (0.76, 2.17) | 0.35 | . (., .) | **.** | 0.95 (0.67, 1.36) | 0.79 | . (., .) | **.** |
| Trifucosyllacto-N-hexaose-X1 | 1.90 (0.97, 3.74) | 0.06 | . (., .) | **.** | 1.15 (0.78, 1.68) | 0.49 | . (., .) | **.** |
| Trifucosyllacto-N-octaose-X1 | 1.45 (0.80, 2.61) | 0.22 | . (., .) | **.** | 0.96 (0.65, 1.42) | 0.84 | . (., .) | **.** |
| Trifucosyllacto-N-tetraose-X5 | 1.22 (0.78, 1.90) | 0.38 | . (., .) | **.** | 1.05 (0.76, 1.45) | 0.76 | . (., .) | **.** |
| a-Heptasaccharide | 1.02 (0.56, 1.86) | 0.94 | . (., .) | **.** | 1.06 (0.76, 1.48) | 0.73 | . (., .) | **.** |
| a-Pentasaccharide | 1.19 (0.65, 2.18) | 0.57 | . (., .) | **.** | 1.10 (0.78, 1.54) | 0.6 | . (., .) | **.** |
| a-Tetrasaccharide | 1.07 (0.53, 2.17) | 0.85 | . (., .) | **.** | 1.02 (0.71, 1.49) | 0.9 | . (., .) | **.** |

Human milk oligosaccharide levels were normalised following stratification by secretor status. Associations determined by modified Poisson regression. Models adjusted for batch, child sex, duration of exclusive breastfeeding (weeks) and gestation age (weeks). Bold denotes statistically significant at nominal level (p<0.05). *Denotes statistically significant after correction for multiple testing (FDR<0.010). RR: Risk Ratio; CI: Confidence Interval; OM: Otitis Media; HMO: Human milk oligosaccharides

**Table S27**: Adjusted associations between human milk oligosaccharides measured at 6 months of lactation with lower respiratory tract infections (LRTI) in the first or second year of life stratified by secretor status in the Ulm SPATZ Health Study.

|  | **LRTI at 1 year** | | | | **LRTI at 2 years** | | | |
| --- | --- | --- | --- | --- | --- | --- | --- | --- |
|  | Secretor milk | | Non-secretor milk | | Secretor milk | | Non-secretor milk | |
| **HMO Structure** | RR (95% CI) | p | RR (95% CI) | p | RR (95% CI) | p | RR (95% CI) | p |
| 2,3 Difucosyllactose | 0.72 (0.44, 1.18) | 0.2 | . (., .) | **.** | 0.71 (0.50, 1.02) | 0.06 | . (., .) | **.** |
| 2-Fucosyllactose | 0.80 (0.46, 1.40) | 0.44 | . (., .) | **.** | 1.09 (0.77, 1.53) | 0.63 | . (., .) | **.** |
| **3'-Sialyl-3-fucosyllactose** | 0.90 (0.55, 1.47) | 0.66 | 1.18 (0.80, 1.74) | 0.41 | **0.69 (0.49, 0.97)** | **0.03** | 1.19 (0.93, 1.53) | 0.18 |
| **3-Fucosyllactose** | 0.74 (0.46, 1.18) | 0.2 | 0.99 (0.65, 1.50) | 0.96 | **0.66 (0.48, 0.91)** | **0.01** | 1.00 (0.73, 1.37) | 0.99 |
| 3-Galactosyllactose | 1.38 (0.81, 2.37) | 0.24 | 0.94 (0.65, 1.35) | 0.73 | 1.05 (0.74, 1.49) | 0.8 | 0.90 (0.68, 1.18) | 0.44 |
| 3-sialyllactose | 1.14 (0.63, 2.06) | 0.67 | 1.17 (0.78, 1.75) | 0.45 | 1.07 (0.72, 1.60) | 0.73 | 1.23 (0.95, 1.60) | 0.11 |
| 6'-Sialyl-N-acetyllactosamine | 1.29 (0.76, 2.21) | 0.35 | 1.20 (0.86, 1.67) | 0.29 | 1.13 (0.78, 1.63) | 0.52 | 1.09 (0.83, 1.41) | 0.54 |
| 6-Galactosyllactose | 0.80 (0.53, 1.23) | 0.31 | 0.78 (0.55, 1.12) | 0.17 | 0.80 (0.61, 1.06) | 0.12 | 0.83 (0.63, 1.09) | 0.18 |
| 6-sialyllactose | 1.03 (0.60, 1.76) | 0.91 | 1.16 (0.83, 1.60) | 0.38 | 1.13 (0.80, 1.61) | 0.48 | 1.02 (0.79, 1.31) | 0.9 |
| **Difucosyl-lacto-N-hexaose I** | 1.36 (0.80, 2.28) | 0.25 | . (., .) | **.** | **1.49 (1.08, 2.06)** | **0.02** | . (., .) | **.** |
| Difucosyl-lacto-N-hexaose II | 1.21 (0.74, 2.00) | 0.45 | 0.98 (0.67, 1.44) | 0.91 | 0.91 (0.65, 1.26) | 0.56 | 1.02 (0.77, 1.37) | 0.87 |
| **Difucosyl-lacto-N-hexaose-X1** | 1.50 (0.96, 2.35) | 0.07 | . (., .) | **.** | **1.41 (1.05, 1.88)** | **0.02** | . (., .) | **.** |
| Difucosyl-lacto-N-hexaose-X2 | 1.25 (0.74, 2.12) | 0.41 | . (., .) | **.** | 0.96 (0.68, 1.35) | 0.82 | . (., .) | **.** |
| Difucosyl-lacto-N-hexaose-X3 | 0.82 (0.54, 1.26) | 0.37 | 0.92 (0.64, 1.32) | 0.65 | 0.77 (0.59, 1.00) | 0.05 | 1.02 (0.78, 1.35) | 0.87 |
| **Difucosyl-lacto-N-neo-hexaose I** | 0.65 (0.41, 1.03) | 0.07 | 0.82 (0.57, 1.18) | 0.29 | **0.72 (0.55, 0.93)** | **0.01** | 0.97 (0.72, 1.30) | 0.85 |
| Difucosyl-para-lacto-N-hexaose I | 1.28 (0.74, 2.20) | 0.38 | 0.92 (0.64, 1.33) | 0.67 | 0.88 (0.63, 1.22) | 0.44 | 1.01 (0.76, 1.35) | 0.93 |
| Difucosyldisialyllacto-N-hexaose-X1 | 1.21 (0.71, 2.05) | 0.49 | . (., .) | **.** | 1.09 (0.80, 1.50) | 0.58 | . (., .) | **.** |
| Difucosyldisialyllacto-N-hexaose-X2 | 1.03 (0.63, 1.68) | 0.92 | 1.21 (0.83, 1.76) | 0.33 | 0.90 (0.64, 1.26) | 0.53 | 1.19 (0.89, 1.60) | 0.24 |
| Difucosylsialyllacto-N-hexaose-X1 | 1.00 (0.56, 1.81) | 0.99 | . (., .) | **.** | 0.97 (0.65, 1.45) | 0.88 | . (., .) | **.** |
| Difucosylsialyllacto-N-hexaose-X2 | 0.78 (0.43, 1.42) | 0.42 | . (., .) | **.** | 0.82 (0.56, 1.20) | 0.3 | . (., .) | **.** |
| Disialyllacto-N-Hexaose-X1 | 1.24 (0.75, 2.05) | 0.4 | 0.97 (0.66, 1.43) | 0.87 | 1.17 (0.84, 1.64) | 0.35 | 1.08 (0.80, 1.48) | 0.61 |
| Disialyllacto-N-Hexaose-X2 | 1.50 (0.98, 2.31) | 0.06 | 1.05 (0.72, 1.53) | 0.81 | 1.20 (0.90, 1.61) | 0.21 | 1.03 (0.75, 1.40) | 0.87 |
| Disialyllacto-N-Hexaose-X5 | 1.02 (0.59, 1.76) | 0.94 | 1.32 (0.98, 1.76) | 0.06 | 1.08 (0.74, 1.57) | 0.68 | 1.24 (0.97, 1.57) | 0.08 |
| Disialyllacto-N-tetraose | 1.27 (0.77, 2.08) | 0.35 | 1.04 (0.70, 1.52) | 0.86 | 1.23 (0.91, 1.68) | 0.18 | 0.93 (0.67, 1.28) | 0.64 |
| **Fucosyl(1-3)-iso-lacto-N-octaose** | **2.09 (1.40, 3.11)** | **<0.001** | 0.92 (0.63, 1.34) | 0.66 | **1.57 (1.15, 2.14)** | **0.004** | 0.94 (0.69, 1.28) | 0.7 |
| **Fucosyl-para-lacto-N-hexaose I** | **1.75 (1.17, 2.62)** | **0.01** | 1.06 (0.70, 1.61) | 0.78 | 1.28 (0.97, 1.69) | 0.08 | 1.06 (0.77, 1.45) | 0.74 |
| Fucosyldiasialyllacto-N-hexaose-X1 | 1.12 (0.64, 1.94) | 0.7 | 1.11 (0.77, 1.62) | 0.57 | 1.18 (0.83, 1.68) | 0.35 | 1.01 (0.73, 1.41) | 0.93 |
| Fucosyldiasialyllacto-N-hexaose-X2 | 1.21 (0.76, 1.93) | 0.42 | 0.93 (0.64, 1.36) | 0.71 | 1.12 (0.82, 1.52) | 0.49 | 0.95 (0.69, 1.30) | 0.75 |
| Fucosyldisialyllacto-N-tetraose-X2 | 1.16 (0.69, 1.96) | 0.58 | 1.13 (0.80, 1.61) | 0.5 | 0.83 (0.58, 1.17) | 0.28 | 1.06 (0.82, 1.38) | 0.65 |
| **Fucosyllacto-N-hexaose II** | **1.59 (1.01, 2.50)** | **0.04** | 1.01 (0.70, 1.46) | 0.96 | 1.28 (0.94, 1.73) | 0.12 | 0.96 (0.71, 1.29) | 0.78 |
| Fucosyllacto-N-hexaose-X1 | 1.39 (0.93, 2.07) | 0.11 | . (., .) | **.** | 1.15 (0.87, 1.53) | 0.32 | . (., .) | **.** |
| Fucosyllacto-N-hexaose-X2 | 1.52 (0.94, 2.44) | 0.09 | . (., .) | **.** | 1.30 (0.95, 1.78) | 0.1 | . (., .) | **.** |
| Fucosyllacto-N-hexaose-X4 | 1.15 (0.73, 1.82) | 0.54 | 0.92 (0.62, 1.37) | 0.69 | 0.97 (0.74, 1.28) | 0.84 | 0.99 (0.73, 1.34) | 0.95 |
| Fucosyllacto-N-octaose-X1 | 1.09 (0.59, 1.99) | 0.79 | . (., .) | **.** | 1.10 (0.80, 1.52) | 0.56 | . (., .) | **.** |
| Fucosyllacto-N-octaose-X2 | 1.36 (0.84, 2.21) | 0.21 | 0.95 (0.60, 1.48) | 0.81 | 0.93 (0.65, 1.35) | 0.72 | 0.87 (0.58, 1.29) | 0.49 |
| Fucosyllacto-N-sulfate-X1 | 1.45 (0.92, 2.26) | 0.11 | . (., .) | **.** | 1.15 (0.84, 1.56) | 0.38 | . (., .) | **.** |
| Fucosylsialyllacto-N-Hexaose-X1 | 1.58 (0.94, 2.64) | 0.08 | 1.11 (0.74, 1.67) | 0.61 | 1.16 (0.80, 1.68) | 0.44 | 1.10 (0.83, 1.46) | 0.51 |
| Fucosylsialyllacto-N-Hexaose-X2 | 1.24 (0.69, 2.23) | 0.47 | 0.85 (0.53, 1.37) | 0.51 | 1.40 (0.99, 1.98) | 0.06 | 0.76 (0.51, 1.13) | 0.17 |
| **Fucosylsialyllacto-N-Hexaose-X3** | 1.13 (0.60, 2.13) | 0.7 | 0.89 (0.57, 1.40) | 0.62 | **1.47 (1.02, 2.12)** | **0.04** | 0.89 (0.61, 1.30) | 0.54 |
| Fucosylsialyllacto-N-Hexaose-X4 | 0.73 (0.46, 1.17) | 0.19 | 1.21 (0.88, 1.68) | 0.24 | 0.88 (0.63, 1.22) | 0.44 | 1.12 (0.86, 1.46) | 0.39 |
| Fucosylsialyllacto-N-Hexaose-X5 | 1.41 (0.74, 2.67) | 0.29 | 1.20 (0.83, 1.74) | 0.34 | 1.05 (0.62, 1.78) | 0.85 | 1.14 (0.85, 1.52) | 0.39 |
| Fucosylsialyllacto-N-Hexaose-X6 | 0.76 (0.41, 1.40) | 0.38 | 0.93 (0.64, 1.35) | 0.72 | 0.79 (0.50, 1.24) | 0.31 | 0.96 (0.72, 1.28) | 0.76 |
| Fucosylsialyllacto-N-neo-tetraose c | 1.00 (0.61, 1.64) | 1 | 1.27 (0.89, 1.80) | 0.19 | 0.76 (0.56, 1.03) | 0.08 | 1.14 (0.87, 1.49) | 0.35 |
| Fucosylsialyllacto-N-tetraose a | 1.06 (0.59, 1.91) | 0.84 | 0.91 (0.65, 1.27) | 0.58 | 0.90 (0.61, 1.32) | 0.6 | 0.85 (0.63, 1.13) | 0.26 |
| **Fucosylsialyllacto-N-tetraose b** | **1.64 (1.00, 2.67)** | **0.05** | . (., .) | **.** | 1.26 (0.92, 1.72) | 0.16 | . (., .) | **.** |
| Fucosylsialyllacto-N-tetraose-X1 | 1.25 (0.67, 2.34) | 0.48 | 1.02 (0.68, 1.52) | 0.93 | 0.97 (0.65, 1.46) | 0.89 | 0.99 (0.74, 1.32) | 0.94 |
| Lacto-N-decaose-X1 | 1.50 (0.82, 2.76) | 0.19 | 1.12 (0.75, 1.66) | 0.58 | 1.29 (0.85, 1.98) | 0.24 | 1.01 (0.70, 1.45) | 0.95 |
| Lacto-N-difucohexaose I | 1.31 (0.77, 2.24) | 0.32 | . (., .) | **.** | 0.97 (0.68, 1.39) | 0.86 | . (., .) | **.** |
| **Lacto-N-fucopentaose I** | **1.82 (1.18, 2.81)** | **0.01** | . (., .) | **.** | **1.58 (1.19, 2.10)** | **0.002** | . (., .) | **.** |
| Lacto-N-fucopentaose II | 1.59 (0.91, 2.78) | 0.1 | 0.86 (0.59, 1.25) | 0.44 | 1.03 (0.72, 1.46) | 0.88 | 0.89 (0.67, 1.19) | 0.43 |
| Lacto-N-fucopentaose III | 0.93 (0.57, 1.50) | 0.75 | 0.91 (0.64, 1.29) | 0.58 | 0.83 (0.63, 1.10) | 0.19 | 1.00 (0.75, 1.33) | 0.99 |
| Lacto-N-fucopentaose V | 1.72 (1.00, 2.96) | 0.05 | 0.95 (0.67, 1.34) | 0.76 | 1.05 (0.74, 1.48) | 0.79 | 0.94 (0.70, 1.25) | 0.65 |
| **Lacto-N-hexaose** | **1.95 (1.23, 3.10)** | **0.005** | 1.11 (0.79, 1.57) | 0.56 | **1.50 (1.06, 2.12)** | **0.02** | 0.98 (0.75, 1.29) | 0.91 |
| Lacto-N-hexaose neo | 1.40 (0.93, 2.10) | 0.1 | 1.06 (0.73, 1.53) | 0.76 | 1.12 (0.85, 1.49) | 0.42 | 1.05 (0.78, 1.41) | 0.74 |
| Lacto-N-neo-difucohexaose I | 1.06 (0.69, 1.63) | 0.78 | . (., .) | **.** | 1.04 (0.78, 1.39) | 0.78 | . (., .) | **.** |
| Lacto-N-neo-difucohexaose II | 0.76 (0.48, 1.21) | 0.25 | 0.99 (0.67, 1.46) | 0.95 | **0.63 (0.49, 0.83)** | **0.001** | 1.05 (0.79, 1.40) | 0.73 |
| Lacto-N-neo-octaose | 1.28 (0.76, 2.18) | 0.36 | 0.68 (0.34, 1.36) | 0.27 | 1.11 (0.78, 1.58) | 0.56 | 0.79 (0.46, 1.37) | 0.41 |
| **Lacto-N-tetraose** | **2.18 (1.49, 3.18)** | **<0.001*** | 0.95 (0.65, 1.38) | 0.79 | **1.39 (1.04, 1.85)** | **0.02** | 0.91 (0.67, 1.24) | 0.55 |
| **Lacto-N-triose II** | **3.26 (1.47, 7.22)** | **0.004** | 1.54 (1.08, 2.20) | **0.02** | **1.52 (1.11, 2.10)** | **0.01** | 1.15 (0.89, 1.49) | 0.29 |
| Lactose-3'-Sulfate | 1.05 (0.61, 1.80) | 0.87 | 0.74 (0.50, 1.10) | 0.13 | 0.99 (0.68, 1.45) | 0.97 | 0.80 (0.59, 1.09) | 0.16 |
| Sialyllacto-N-hexaose-X1 | 1.38 (0.78, 2.42) | 0.27 | 1.14 (0.78, 1.66) | 0.5 | 1.12 (0.72, 1.74) | 0.61 | 1.03 (0.77, 1.39) | 0.83 |
| Sialyllacto-N-hexaose-X2 | 1.03 (0.59, 1.79) | 0.92 | . (., .) | **.** | 1.01 (0.72, 1.42) | 0.94 | . (., .) | **.** |
| **Sialyllacto-N-tetraose a** | **1.63 (1.15, 2.33)** | **0.01** | 0.95 (0.63, 1.43) | 0.8 | **1.72 (1.35, 2.19)** | **<0.001*** | 0.80 (0.61, 1.06) | 0.12 |
| **Sialyllacto-N-tetraose b** | **1.75 (1.12, 2.73)** | **0.01** | 0.98 (0.69, 1.37) | 0.89 | 1.15 (0.85, 1.54) | 0.37 | 0.99 (0.76, 1.29) | 0.95 |
| Sialyllacto-N-tetraose c | 1.11 (0.72, 1.71) | 0.63 | 1.18 (0.85, 1.62) | 0.32 | 1.04 (0.76, 1.43) | 0.8 | 1.07 (0.83, 1.39) | 0.59 |
| Trifucosyl(1-2,1-2,1-3)-iso-lacto-N-octa | 1.57 (0.98, 2.51) | 0.06 | . (., .) | **.** | **1.49 (1.08, 2.07)** | **0.02** | . (., .) | **.** |
| Trifucosyllacto-N-hexaose I | 1.06 (0.68, 1.67) | 0.79 | . (., .) | **.** | 0.91 (0.66, 1.27) | 0.59 | . (., .) | **.** |
| Trifucosyllacto-N-hexaose-X1 | 1.20 (0.75, 1.91) | 0.45 | . (., .) | **.** | 0.89 (0.64, 1.24) | 0.5 | . (., .) | **.** |
| **Trifucosyllacto-N-octaose-X1** | **1.97 (1.23, 3.16)** | **<0.001** | . (., .) | **.** | 1.32 (0.96, 1.81) | 0.08 | . (., .) | **.** |
| Trifucosyllacto-N-tetraose-X5 | 1.00 (0.65, 1.53) | 0.98 | . (., .) | **.** | 0.91 (0.65, 1.27) | 0.58 | . (., .) | **.** |
| a-Heptasaccharide | 1.05 (0.65, 1.70) | 0.84 | . (., .) | **.** | 1.03 (0.76, 1.39) | 0.84 | . (., .) | **.** |
| a-Pentasaccharide | 1.15 (0.75, 1.75) | 0.53 | . (., .) | **.** | 1.08 (0.81, 1.42) | 0.61 | . (., .) | **.** |
| a-Tetrasaccharide | 1.43 (0.88, 2.32) | 0.15 | . (., .) | **.** | 1.22 (0.90, 1.66) | 0.2 | . (., .) | **.** |

Human milk oligosaccharide levels were normalised following stratification by secretor status. Associations determined by modified Poisson regression. Models adjusted for batch, child sex, duration of exclusive breastfeeding (weeks) and gestation age (weeks). Bold denotes statistically significant at nominal level (p<0.05). *Denotes statistically significant after correction for multiple testing (FDR<0.010). RR: Risk Ratio; CI: Confidence Interval; LRTI: Lower respiratory tract infections; HMO: Human milk oligosaccharides

**Table S28**: Adjusted associations between human milk oligosaccharides measured at 6 months of lactation with upper respiratory tract infections (URTI) in the first or second year of life stratified by secretor status in the Ulm SPATZ Health Study.

|  | **URTI at 1 year** | | | | **URTI at 2 years** | | | |
| --- | --- | --- | --- | --- | --- | --- | --- | --- |
|  | Secretor milk | | Non-secretor milk | | Secretor milk | | Non-secretor milk | |
| **HMO Structure** | RR (95% CI) | p | RR (95% CI) | p | RR (95% CI) | p | RR (95% CI) | p |
| 2,3 Difucosyllactose | 0.99 (0.84, 1.17) | 0.95 | . (., .) | **.** | 0.99 (0.87, 1.12) | 0.84 | . (., .) | **.** |
| 2-Fucosyllactose | 0.90 (0.74, 1.09) | 0.29 | . (., .) | **.** | 0.90 (0.79, 1.02) | 0.10 | . (., .) | **.** |
| **3'-Sialyl-3-fucosyllactose** | 1.15 (0.96, 1.38) | 0.13 | **1.23 (1.04, 1.47)** | **0.02** | 1.07 (0.93, 1.22) | 0.38 | 1.08 (0.98, 1.19) | 0.14 |
| 3-Fucosyllactose | 1.07 (0.91, 1.26) | 0.42 | 1.03 (0.85, 1.24) | 0.79 | 1.05 (0.92, 1.20) | 0.44 | 0.99 (0.88, 1.11) | 0.84 |
| **3-Galactosyllactose** | 1.12 (0.94, 1.34) | 0.21 | **1.17 (1.01, 1.34)** | **0.03** | 1.06 (0.95, 1.20) | 0.30 | 1.01 (0.95, 1.07) | 0.81 |
| **3-sialyllactose** | 1.08 (0.93, 1.25) | 0.32 | **1.24 (1.05, 1.47)** | **0.01** | 0.99 (0.90, 1.08) | 0.77 | 1.11 (0.99, 1.24) | 0.07 |
| **6'-Sialyl-N-acetyllactosamine** | 1.04 (0.87, 1.25) | 0.66 | **1.24 (1.05, 1.46)** | **0.01** | 0.98 (0.86, 1.12) | 0.76 | 1.09 (0.98, 1.22) | 0.12 |
| 6-Galactosyllactose | 0.99 (0.79, 1.23) | 0.92 | 1.08 (0.92, 1.28) | 0.36 | 0.98 (0.85, 1.13) | 0.75 | 1.03 (0.92, 1.15) | 0.60 |
| 6-sialyllactose | 1.04 (0.87, 1.25) | 0.66 | **1.25 (1.01, 1.55)** | **0.04** | 0.96 (0.84, 1.10) | 0.56 | **1.23 (1.08, 1.41)** | **0.002** |
| Difucosyl-lacto-N-hexaose I | 0.97 (0.82, 1.15) | 0.71 | . (., .) | **.** | 0.94 (0.83, 1.07) | 0.35 | . (., .) | **.** |
| Difucosyl-lacto-N-hexaose II | 1.17 (0.94, 1.45) | 0.16 | 1.01 (0.85, 1.19) | 0.92 | 1.08 (0.92, 1.26) | 0.35 | 1.02 (0.94, 1.11) | 0.56 |
| Difucosyl-lacto-N-hexaose-X1 | 1.04 (0.86, 1.25) | 0.72 | . (., .) | **.** | 1.00 (0.86, 1.16) | 0.97 | . (., .) | **.** |
| Difucosyl-lacto-N-hexaose-X2 | 1.08 (0.91, 1.28) | 0.40 | . (., .) | **.** | 1.04 (0.89, 1.21) | 0.61 | . (., .) | **.** |
| **Difucosyl-lacto-N-hexaose-X3** | **1.23 (1.03, 1.47)** | **0.02** | **1.25 (1.03, 1.53)** | **0.03** | 1.10 (0.97, 1.25) | 0.12 | 1.09 (0.97, 1.24) | 0.14 |
| Difucosyl-lacto-N-neo-hexaose I | 1.12 (0.90, 1.40) | 0.32 | 1.07 (0.87, 1.31) | 0.52 | 1.10 (0.94, 1.29) | 0.24 | 1.04 (0.92, 1.17) | 0.55 |
| **Difucosyl-para-lacto-N-hexaose I** | **1.25 (1.04, 1.51)** | **0.02** | 1.20 (0.99, 1.47) | 0.06 | 1.14 (0.99, 1.30) | 0.07 | 1.08 (0.96, 1.22) | 0.20 |
| Difucosyldisialyllacto-N-hexaose-X1 | 1.07 (0.84, 1.38) | 0.57 | . (., .) | **.** | 0.92 (0.75, 1.14) | 0.46 | . (., .) | **.** |
| Difucosyldisialyllacto-N-hexaose-X2 | 1.10 (0.89, 1.36) | 0.38 | 1.05 (0.88, 1.26) | 0.58 | 0.97 (0.82, 1.14) | 0.70 | 1.04 (0.94, 1.14) | 0.48 |
| Difucosylsialyllacto-N-hexaose-X1 | 1.07 (0.85, 1.35) | 0.56 | . (., .) | **.** | 0.96 (0.83, 1.11) | 0.55 | . (., .) | **.** |
| Difucosylsialyllacto-N-hexaose-X2 | 0.90 (0.73, 1.11) | 0.33 | . (., .) | **.** | 0.90 (0.76, 1.06) | 0.20 | . (., .) | **.** |
| Disialyllacto-N-Hexaose-X1 | 1.09 (0.92, 1.30) | 0.32 | 1.05 (0.84, 1.31) | 0.65 | 0.99 (0.86, 1.13) | 0.85 | 1.03 (0.87, 1.23) | 0.71 |
| **Disialyllacto-N-Hexaose-X2** | 1.15 (0.96, 1.39) | 0.14 | **1.22 (1.02, 1.45)** | **0.03** | 0.98 (0.87, 1.11) | 0.79 | 1.08 (0.97, 1.19) | 0.16 |
| **Disialyllacto-N-Hexaose-X5** | 1.07 (0.87, 1.31) | 0.55 | **1.23 (1.00, 1.50)** | **0.05** | 0.98 (0.85, 1.14) | 0.83 | **1.15 (1.03, 1.28)** | **0.01** |
| **Disialyllacto-N-tetraose** | 1.08 (0.89, 1.32) | 0.44 | **1.23 (1.01, 1.51)** | **0.04** | 0.93 (0.82, 1.05) | 0.24 | **1.15 (1.01, 1.31)** | **0.04** |
| Fucosyl(1-3)-iso-lacto-N-octaose | 1.07 (0.92, 1.24) | 0.40 | 1.04 (0.84, 1.28) | 0.74 | 1.02 (0.93, 1.13) | 0.64 | 1.08 (0.95, 1.24) | 0.25 |
| Fucosyl-para-lacto-N-hexaose I | 1.13 (0.94, 1.37) | 0.20 | 1.14 (0.94, 1.37) | 0.18 | 1.06 (0.92, 1.23) | 0.43 | 1.10 (0.97, 1.26) | 0.14 |
| Fucosyldiasialyllacto-N-hexaose-X1 | 1.10 (0.91, 1.33) | 0.33 | 0.96 (0.75, 1.23) | 0.74 | 0.97 (0.85, 1.10) | 0.65 | 1.04 (0.93, 1.17) | 0.46 |
| **Fucosyldiasialyllacto-N-hexaose-X2** | 1.22 (0.98, 1.53) | 0.08 | **1.30 (1.08, 1.56)** | **0.01** | 1.00 (0.87, 1.15) | 0.98 | **1.16 (1.02, 1.32)** | **0.02** |
| **Fucosyldisialyllacto-N-tetraose-X2** | 1.20 (0.99, 1.45) | 0.07 | **1.22 (1.03, 1.44)** | **0.02** | 1.06 (0.91, 1.23) | 0.44 | 1.07 (0.98, 1.17) | 0.16 |
| Fucosyllacto-N-hexaose II | 1.07 (0.89, 1.28) | 0.49 | 0.95 (0.78, 1.17) | 0.66 | 1.02 (0.90, 1.15) | 0.73 | 1.03 (0.93, 1.14) | 0.56 |
| Fucosyllacto-N-hexaose-X1 | 1.03 (0.84, 1.27) | 0.76 | . (., .) | **.** | 0.96 (0.83, 1.12) | 0.61 | . (., .) | **.** |
| Fucosyllacto-N-hexaose-X2 | 1.06 (0.88, 1.29) | 0.55 | . (., .) | **.** | 0.93 (0.80, 1.09) | 0.39 | . (., .) | **.** |
| **Fucosyllacto-N-hexaose-X4** | **1.21 (1.01, 1.44)** | **0.04** | 1.23 (0.99, 1.53) | 0.06 | 1.08 (0.97, 1.20) | 0.16 | 1.03 (0.89, 1.20) | 0.66 |
| Fucosyllacto-N-octaose-X1 | 1.05 (0.85, 1.28) | 0.66 | . (., .) | **.** | 0.93 (0.77, 1.13) | 0.47 | . (., .) | **.** |
| Fucosyllacto-N-octaose-X2 | 1.12 (0.95, 1.32) | 0.16 | 1.00 (0.78, 1.28) | 1.00 | 1.08 (0.97, 1.21) | 0.17 | 0.95 (0.80, 1.13) | 0.56 |
| Fucosyllacto-N-sulfate-X1 | 1.11 (0.94, 1.31) | 0.21 | . (., .) | **.** | 1.04 (0.95, 1.14) | 0.40 | . (., .) | **.** |
| Fucosylsialyllacto-N-Hexaose-X1 | 1.09 (0.90, 1.32) | 0.36 | 1.10 (0.90, 1.34) | 0.36 | 0.97 (0.83, 1.12) | 0.64 | 1.10 (0.99, 1.21) | 0.07 |
| Fucosylsialyllacto-N-Hexaose-X2 | 1.01 (0.83, 1.25) | 0.89 | 0.65 (0.42, 1.01) | 0.06 | 0.94 (0.80, 1.12) | 0.50 | 0.90 (0.75, 1.08) | 0.27 |
| **Fucosylsialyllacto-N-Hexaose-X3** | 0.89 (0.73, 1.09) | 0.27 | 1.17 (1.01, 1.36) | **0.04** | 0.87 (0.75, 1.01) | 0.07 | **1.11 (1.02, 1.20)** | **0.02** |
| **Fucosylsialyllacto-N-Hexaose-X4** | 1.16 (0.97, 1.39) | 0.10 | **1.27 (1.01, 1.60)** | **0.04** | 1.02 (0.90, 1.16) | 0.75 | **1.19 (1.04, 1.35)** | **0.01** |
| **Fucosylsialyllacto-N-Hexaose-X5** | 1.14 (0.95, 1.36) | 0.16 | 1.11 (0.91, 1.37) | 0.31 | 0.99 (0.85, 1.17) | 0.94 | **1.15 (1.03, 1.28)** | **0.01** |
| **Fucosylsialyllacto-N-Hexaose-X6** | 1.12 (0.94, 1.33) | 0.20 | **1.33 (1.10, 1.60)** | **0.003** | 1.05 (0.93, 1.18) | 0.42 | **1.16 (1.04, 1.29)** | **0.01** |
| **Fucosylsialyllacto-N-neo-tetraose c** | **1.33 (1.11, 1.59)** | **0.002** | 1.21 (0.99, 1.48) | 0.06 | 1.11 (0.97, 1.26) | 0.12 | **1.13 (1.03, 1.24)** | **0.01** |
| Fucosylsialyllacto-N-tetraose a | 1.06 (0.86, 1.31) | 0.60 | 1.00 (0.83, 1.20) | 0.99 | 1.04 (0.88, 1.22) | 0.67 | 1.03 (0.95, 1.13) | 0.46 |
| Fucosylsialyllacto-N-tetraose b | 1.07 (0.86, 1.32) | 0.56 | . (., .) | **.** | 0.97 (0.84, 1.11) | 0.63 | . (., .) | **.** |
| **Fucosylsialyllacto-N-tetraose-X1** | **1.33 (1.10, 1.61)** | **0.004** | **1.32 (1.12, 1.55)** | **0.001** | **1.14 (1.00, 1.30)** | **0.05** | 1.10 (0.99, 1.23) | 0.09 |
| Lacto-N-decaose-X1 | 1.10 (0.91, 1.34) | 0.34 | 0.88 (0.68, 1.15) | 0.35 | 1.00 (0.83, 1.20) | 0.97 | 0.94 (0.80, 1.09) | 0.39 |
| Lacto-N-difucohexaose I | 1.09 (0.89, 1.33) | 0.40 | . (., .) | **.** | 1.04 (0.89, 1.21) | 0.60 | . (., .) | **.** |
| Lacto-N-fucopentaose I | 0.97 (0.80, 1.17) | 0.73 | . (., .) | **.** | 0.93 (0.80, 1.08) | 0.34 | . (., .) | **.** |
| Lacto-N-fucopentaose II | 1.19 (0.99, 1.44) | 0.07 | 1.17 (0.94, 1.46) | 0.16 | 1.10 (0.96, 1.26) | 0.17 | 1.08 (0.93, 1.25) | 0.33 |
| **Lacto-N-fucopentaose III** | **1.23 (1.01, 1.49)** | **0.04** | 1.18 (0.97, 1.43) | 0.10 | 1.11 (0.96, 1.28) | 0.17 | 1.07 (0.95, 1.21) | 0.24 |
| Lacto-N-fucopentaose V | 1.19 (0.97, 1.46) | 0.10 | 1.14 (0.93, 1.39) | 0.21 | 1.11 (0.95, 1.30) | 0.20 | 1.10 (0.94, 1.28) | 0.23 |
| Lacto-N-hexaose | 1.10 (0.91, 1.31) | 0.32 | 1.02 (0.84, 1.23) | 0.87 | 1.01 (0.88, 1.14) | 0.94 | 1.00 (0.88, 1.15) | 0.95 |
| Lacto-N-neo-difucohexaose I | 1.15 (0.94, 1.40) | 0.18 | . (., .) | **.** | 1.02 (0.91, 1.15) | 0.71 | . (., .) | **.** |
| **Lacto-N-neo-difucohexaose II** | **1.22 (1.01, 1.46)** | **0.04** | 1.17 (0.95, 1.43) | 0.13 | 1.11 (0.97, 1.29) | 0.14 | 1.04 (0.95, 1.13) | 0.44 |
| Lacto-N-neo-hexaose | 1.10 (0.92, 1.30) | 0.29 | 1.15 (0.95, 1.39) | 0.16 | 1.05 (0.93, 1.19) | 0.41 | 1.04 (0.91, 1.18) | 0.61 |
| Lacto-N-neo-octaose | 1.15 (0.98, 1.35) | 0.08 | 1.06 (0.86, 1.31) | 0.59 | 1.07 (0.96, 1.20) | 0.22 | 1.08 (0.99, 1.17) | 0.08 |
| Lacto-N-tetraose | 1.11 (0.93, 1.33) | 0.24 | 1.11 (0.91, 1.37) | 0.30 | 1.05 (0.91, 1.21) | 0.50 | 1.10 (0.94, 1.28) | 0.23 |
| Lacto-N-triose II | 1.16 (0.95, 1.41) | 0.14 | 1.14 (0.96, 1.36) | 0.14 | 1.01 (0.89, 1.14) | 0.92 | 1.08 (0.96, 1.21) | 0.23 |
| **Lactose-3'-Sulfate** | 1.08 (0.86, 1.35) | 0.50 | **1.25 (1.01, 1.54)** | **0.04** | 0.99 (0.86, 1.16) | 0.95 | 1.09 (0.98, 1.21) | 0.12 |
| **Sialyllacto-N-hexaose-X1** | 1.09 (0.91, 1.30) | 0.36 | 1.05 (0.84, 1.30) | 0.68 | 0.97 (0.84, 1.12) | 0.70 | **1.16 (1.02, 1.31)** | **0.02** |
| Sialyllacto-N-hexaose-X2 | 1.05 (0.87, 1.26) | 0.64 | 1.01 (0.74, 1.37) | 0.96 | 0.98 (0.86, 1.13) | 0.81 | 1.06 (0.97, 1.16) | 0.21 |
| Sialyllacto-N-tetraose a | 0.98 (0.82, 1.17) | 0.79 | 0.97 (0.77, 1.23) | 0.80 | 0.99 (0.86, 1.13) | 0.85 | 1.04 (0.90, 1.19) | 0.61 |
| Sialyllacto-N-tetraose b | 1.12 (0.92, 1.36) | 0.27 | 1.25 (1.05, 1.49) | **0.01** | 1.00 (0.87, 1.15) | 0.99 | 1.13 (0.99, 1.28) | 0.07 |
| **Sialyllacto-N-tetraose c** | **1.22 (1.04, 1.42)** | **0.01** | 1.22 (0.99, 1.50) | 0.06 | 1.03 (0.94, 1.14) | 0.49 | **1.16 (1.04, 1.31)** | **0.01** |
| Trifucosyl(1-2,1-2,1-3)-iso-lacto-N-octa | 1.02 (0.85, 1.24) | 0.82 | . (., .) | **.** | 0.92 (0.79, 1.06) | 0.26 | . (., .) | **.** |
| Trifucosyllacto-N-hexaose I | 1.04 (0.85, 1.26) | 0.72 | . (., .) | **.** | 0.99 (0.84, 1.16) | 0.92 | . (., .) | **.** |
| Trifucosyllacto-N-hexaose-X1 | 1.16 (0.96, 1.40) | 0.12 | . (., .) | **.** | 1.10 (0.96, 1.26) | 0.16 | . (., .) | **.** |
| Trifucosyllacto-N-octaose-X1 | 1.14 (0.97, 1.34) | 0.12 | . (., .) | **.** | 1.05 (0.92, 1.20) | 0.44 | . (., .) | **.** |
| Trifucosyllacto-N-tetraose-X5 | 1.09 (0.91, 1.30) | 0.34 | . (., .) | **.** | 1.03 (0.92, 1.16) | 0.57 | . (., .) | **.** |
| a-Heptasaccharide | 1.12 (0.92, 1.36) | 0.26 | . (., .) | **.** | 1.10 (0.96, 1.26) | 0.16 | . (., .) | **.** |
| a-Pentasaccharide | 1.12 (0.92, 1.37) | 0.24 | . (., .) | **.** | 1.09 (0.95, 1.25) | 0.21 | . (., .) | **.** |
| a-Tetrasaccharide | 1.12 (0.94, 1.34) | 0.19 | . (., .) | **.** | 1.09 (0.96, 1.23) | 0.17 | . (., .) | **.** |

Human milk oligosaccharide levels were normalised following stratification by secretor status. Associations determined by modified Poisson regression. Models adjusted for batch, child sex, duration of exclusive breastfeeding (weeks) and gestation age (weeks). Bold denotes statistically significant at nominal level (p<0.05). *Denotes statistically significant after correction for multiple testing (FDR<0.010). RR: Risk Ratio; CI: Confidence Interval; URTI: Upper respiratory tract infections; HMO: Human milk oligosaccharides


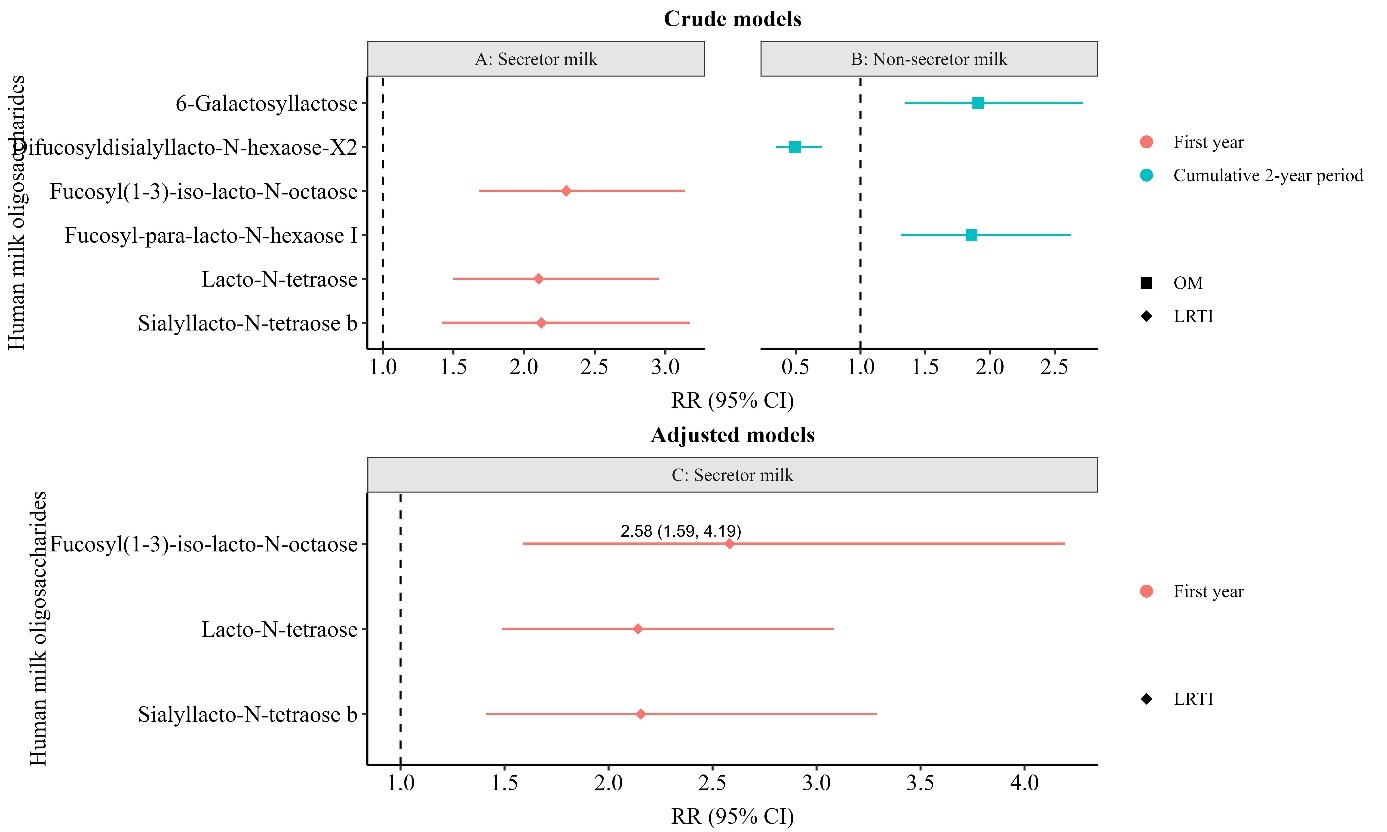


**Figure S2**: Crude (**A** and **B**) and adjusted (**C**) associations between human milk oligosaccharides measured at 6 weeks of lactation with infections in the first or second year of life in the Ulm SPATZ Health Study. All associations shown reached level of statistical significance following corrections for multiple testing (FDR<0.010). Human milk oligosaccharide levels were normalised after stratification by secretor status. Associations determined by modified Poisson regression. Models adjusted for batch, child sex, duration of exclusive breastfeeding (weeks) and gestation age (weeks). OM: Otitis media; LRTI: Lower respiratory tract infections; RR: Risk Ratio determined using weighted secretor status for panel A only; CI: Confidence intervals.

**
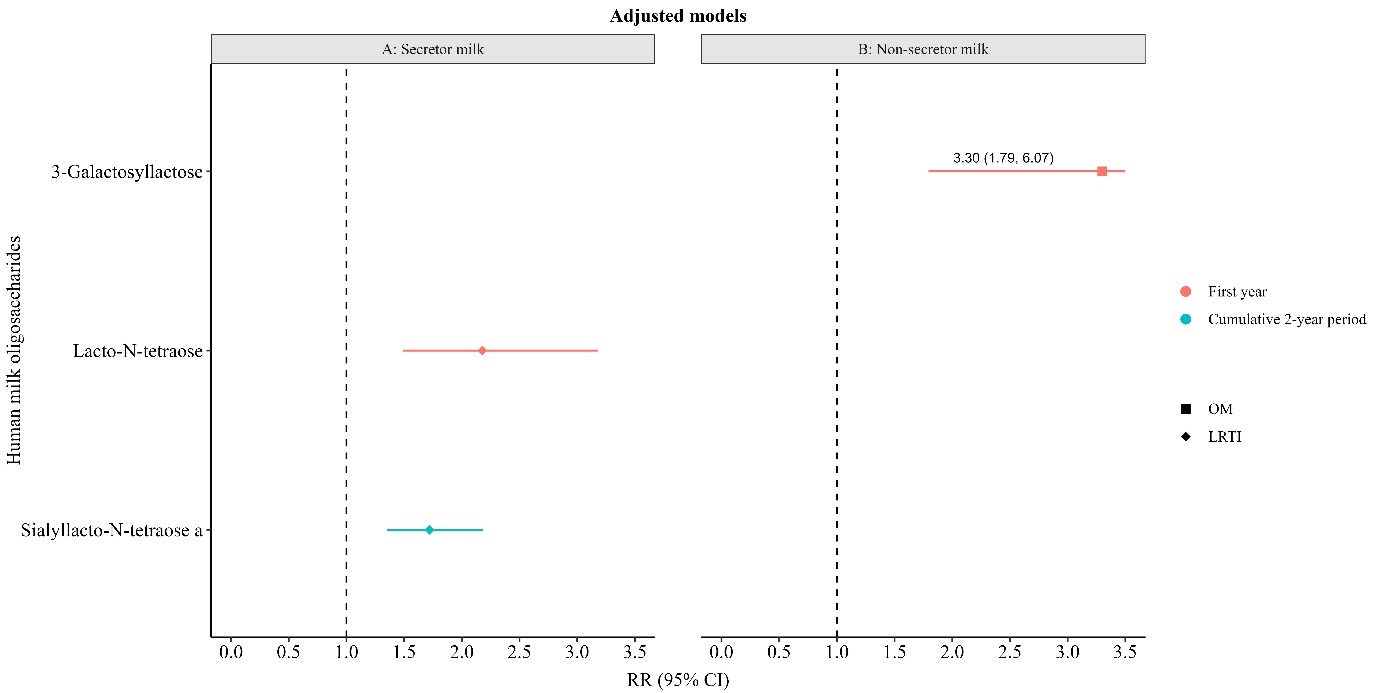
**

**Figure S3:** Adjusted associations between human milk oligosaccharides in secretor and non-secretor milk measured at 6 months of lactation with infections in the first or second year of life in the Ulm SPATZ Health Study. All associations shown reached level of statistical significance following corrections for multiple testing (FDR<0.010). Human milk oligosaccharide levels were normalised after stratification by secretor status. Associations determined by modified Poisson regression. Models adjusted for batch, child sex, duration of exclusive breastfeeding (weeks) and gestation age (weeks). OM: Otitis media; LRTI: Lower respiratory tract infections; RR: Risk Ratio determined using weighted secretor status for panel A only; CI: Confidence intervals.
